# Supplementary material for: Association between vitamin D supplementation or serum vitamin D level and susceptibility to SARS-CoV-2 infection or COVID-19 including clinical course, morbidity and mortality outcomes? A systematic review
Source: BMJ Open. 2021 May 28;11(5):e043737. doi: 10.1136/bmjopen-2020-043737 (PMC8166456; doi:10.1136/bmjopen-2020-043737)
Supplement: Supplementary data [file bmjopen-2020-043737supp001.pdf]

Supplemental file

Contents

1. Full record of search
2. Full details of the study eligibility criteria
3. List of studies excluded at full text review
4. Articles included at full text, but later excluded at time of narrative synthesis
5. Quality assessment of included studies

1. <u>Full record of search</u>

Medline (Ovid)

Search date: 06/05/2020
Database: Ovid MEDLINE(R) ALL <1946 to May 05, 2020>
Search Strategy:
----
1 exp Vitamin D/ (58492)
2 Vitamin D Deficiency/ (15552)
3 (vitamin D? or vit D? or D vitamin? or ergocalciferol or cholecalciferol or colecalciferol or calciferol or dihydroergocalciferol or sitocalciferol or dihydrotachysterol or dihydroxycholecalciferol? or hydroxycholecalciferol? or "25(OH)D?" or 25OHD? or calcidiol or hydroxyergocalciferol or alfacalcidol or alphacalcidol or "1,25(OH)2D" or hydroxyvitamin D? or dihydroxyvitamin D?).ab,kf,ti. (78232)
4 (paricalcitol or doxercalciferol or doxerocalciferol or calcifediol or calcitriol).ab,kf,ti. (5577)
5 hypovitaminosis D?.ab,kf,ti. (1775)
6 ((D or D2 or D3) adj3 supplement*).ab,kf,ti. (12158)
7 1 or 2 or 3 or 4 or 5 or 6 (92560)
8 coronavirus/ or betacoronavirus/ or betacoronavirus 1/ or coronavirus oc43, human/ or middle east respiratory syndrome coronavirus/ or sars virus/ (7431)
9 coronavirus infections/ or severe acute respiratory syndrome/ (10675)
10 (((corona* or corono*) adj1 (virus* or viral* or virinae*)) or coronavirus* or coronovirus* or coronavirinae* or Coronavirus* or Coronovirus* or Wuhan* or Hubei* or Huanan or "2019-nCoV" or 2019nCoV or nCoV2019 or "nCoV-2019" or "COVID-19" or COVID19 or "CORVID-19" or CORVID19 or "WN-CoV" or WNCoV or "HCoV-19" or HCoV19 or CoV or "2019 novel*" or Ncov or "n-cov" or "SARS-CoV-2" or "SARSCoV-2" or "SARSCoV2" or "SARS-CoV2" or SARSCov19 or "SARS-Cov19" or "SARSCov-19" or "SARS-Cov-19" or Ncovor or Ncorona* or Ncorono* or NcovWuhan* or NcovHubei* or NcovChina* or NcovChinese*).ab,kf,ti. (26891)
11 (severe acute respiratory syndrome or SARS or Middle East respiratory syndrome or MERS).ab,kf,ti. (16560)
12 (betacoronavirus* or betacoronavirinae*).ab,kf,ti. (280)
13 8 or 9 or 10 or 11 or 12 (37180)
14 7 and 13 (32)
15 exp Animals/ (23144176)

Grove A, *et al. BMJ Open* 2021; 11:e043737. doi: 10.1136/bmjopen-2020-043737

16 exp Humans/ (18448248)
17 15 not 16 (4695928)
18 14 not 17 (30)
19 limit 18 to yr="2002 -Current" (30)

Update
Search date: 10/6/2020
Actual databases searched: Ovid MEDLINE All <1946 to June 09, 2020>
Search strategy:
Re-ran search above plus…
20 limit 19 to ed=20200506-20200610 (8)
21 limit 19 to ep=20200506-20200610 (39)
22 limit 19 to dt=20200506-20200610 (43)
23 limit 19 to ez=20200506-20200610 (27)
24 20 or 21 or 22 or 23 (46)

Embase (Ovid)

Search date: 06/05/2020
Database: Embase <1974 to 2020 May 05>
Search Strategy:
----
1 exp vitamin D/ (139781)
2 vitamin D deficiency/ (29333)
3 (vitamin D? or vit D? or D vitamin? or ergocalciferol or cholecalciferol or colecalciferol or calciferol or dihydroergocalciferol or sitocalciferol or dihydrotachysterol or dihydroxycholecalciferol? or hydroxycholecalciferol? or "25(OH)D?" or 25OHD? or calcidiol or hydroxyergocalciferol or alfacalcidol or alphacalcidol or "1,25(OH)2D" or hydroxyvitamin D? or dihydroxyvitamin D?).ab,kw,ti. (112459)
4 (paricalcitol or doxercalciferol or doxerocalciferol or calcifediol or calcitriol).ab,kw,ti. (8478)
5 hypovitaminosis D?.ab,kw,ti. (3012)
6 ((D or D2 or D3) adj3 supplement*).ab,kw,ti. (19177)
7 1 or 2 or 3 or 4 or 5 or 6 (163395)
8 betacoronavirus 1/ or betacoronavirus/ or human coronavirus oc43/ (696)
9 Middle East respiratory syndrome coronavirus/ (2028)
10 sars-related coronavirus/ or sars coronavirus/ (6354)
11 Coronavirinae/ (2231)
12 coronavirus infection/ or middle east respiratory syndrome/ or severe acute respiratory syndrome/ (11950)
13 (((corona* or corono*) adj1 (virus* or viral* or virinae*)) or coronavirus* or coronovirus* or coronavirinae* or Coronavirus* or Coronovirus* or Wuhan* or Hubei* or Huanan or "2019-nCoV" or 2019nCoV or nCoV2019 or "nCoV-2019" or "COVID-19" or COVID19 or "CORVID-19" or CORVID19 or "WN-CoV" or WNCoV or "HCoV-19" or HCoV19 or CoV or "2019 novel*" or Ncov or "n-cov" or "SARS-CoV-2" or "SARSCoV-2" or "SARSCoV2" or "SARS-CoV2" or SARSCov19 or "SARS-Cov19" or "SARSCov-19" or "SARS-Cov-19" or Ncovor or Ncorona* or Ncorono* or NcovWuhan* or NcovHubei* or NcovChina* or NcovChinese*).ab,kw,ti. (27686)
14 (severe acute respiratory syndrome or SARS or Middle East respiratory syndrome or MERS).ab,kw,ti. (17146)
15 (betacoronavirus* or betacoronavirinae*).ab,kw,ti. (275)
16 8 or 9 or 10 or 11 or 12 or 13 or 14 or 15 (40716)

Grove A, *et al. BMJ Open* 2021; 11:e043737. doi: 10.1136/bmjopen-2020-043737

17 7 and 16 (61)
18 exp animal/ (25459151)
19 exp human/ (20834835)
20 18 not 19 (4624316)
21 17 not 20 (58)
22 limit 21 to yr="2002 -Current" (58)

Update
Search date: 10/6/2020
Actual databases searched: Ovid Embase <1974 to 2020 June 09>
Search strategy:
Re-ran search above plus…
22 limit 21 to yr="2002 -Current" (123)
23 limit 22 to dd=20200506-20200610 (39)
24 limit 22 to em=202005-202006 (0)
25 limit 22 to dc=20200506-20200610 (62)
26 23 or 24 or 25 (62)

MedrXiv (searched via Medrxivr https://mcguinlu.shinyapps.io/medrxivr/)

Search date: 07/05/2020
Search Strategy:

Topic 1:

[Vv]itamin D
[Vv]itamin D2
[Vv]itamin D3
calciferol
25OHD
25OHD3
[Hh]ypovitaminosis D

Topic 2:

[Cc]oronavirus
[Cc]orona(\\s)([[:graph:]]+\\s){0,1}virus
[Cc]oronavirinae
[Cc]ovid
COVID
nCoV
NCOV
Ncov
[Nn]-cov
N-COV
2019ncov
2019-ncov
ncov2019
ncov-2019

SARS
[Ss]evere [Aa]cute [Rr]espiratory [Ss]yndrome
[Mm]iddle [Ee]ast [Rr]espiratory [Ss]yndrome
MERS

Earliest record date
20190101
Latest record date
20200507
Remove older versions of the same record

6 results

<u>Update</u>
Search date: 10/6/2020
Re-ran search above changing record dates as follows:
Earliest record date
20200507
Latest record date
20200610
Remove older versions of the same record

11 results

BioRxiv
https://www.biorxiv.org/

Search date: 07/05/2020

65 Results
for abstract or title "vitamin D" (match phrase words)

22 Results
for full text or abstract or title "ergocalciferol cholecalciferol colecalciferol" (match whole any)

41 Results
for full text or abstract or title "25OHD 25OHD3" (match whole any)

Imported into EndNote and de-duplicated
92 results after deduplication

Searched in Endnote using the following search strategy:
coronavirus or corona or covid or SARS or MERS or betacoronavirus or ncov
Any Field

5 results

<u>Update</u>
Search date: 10/6/2020

1 Results
for abstract or title "vitamin D" (match phrase words) and posted between "07 May, 2020 and 10 Jun, 2020" – *animal study (also in both results sets below) so not exported to EndNote*

3 Results
for full text or abstract or title "ergocalciferol cholecalciferol colecalciferol" (match whole any) and posted between "07 May, 2020 and 10 Jun, 2020" *- 2 animal studies and 1 on sertraline in TB*

2 Results
for full text or abstract or title "25OHD 25OHD3" (match whole any) and posted between "07 May, 2020 and 10 Jun, 2020" *- 1 animal study, 1 non-clinical / non-coronavirus*

0 results relevant to coronaviruses

Cochrane Library

Search date: 08/05/2020

ID Search Hits
#1 MeSH descriptor: [Vitamin D] explode all trees 5224
#2 MeSH descriptor: [Vitamin D Deficiency] this term only 1226
#3 ((vitamin NEXT D?) or (vit NEXT D?) or (D NEXT vitamin?) or ergocalciferol or cholecalciferol or colecalciferol or calciferol or dihydroergocalciferol or sitocalciferol or dihydrotachysterol or dihydroxycholecalciferol? or hydroxycholecalciferol? or "25(OH)D" or "25(OH)D3" or 25OHD? or calcidiol or hydroxyergocalciferol or alfacalcidol or alphacalcidol or "1,25(OH)2D" or (hydroxyvitamin NEXT D?) or (dihydroxyvitamin NEXT D?)):ti,ab,kw 12959
#4 (paricalcitol or doxercalciferol or doxerocalciferol or calcifediol or calcitriol):ti,ab,kw 2417
#5 hypovitaminosis NEXT D? 303
#6 ((D OR D2 OR D3) NEAR/3 supplement*):ti,ab,kw 5633
#7 #1 or #2 or #3 or #4 or #5 or #6 14461
#8 MeSH descriptor: [Coronavirus] this term only 2
#9 MeSH descriptor: [Betacoronavirus] this term only 2
#10 MeSH descriptor: [Betacoronavirus 1] this term only 0
#11 MeSH descriptor: [Coronavirus OC43, Human] this term only 0
#12 MeSH descriptor: [Middle East Respiratory Syndrome Coronavirus] explode all trees 1
#13 MeSH descriptor: [SARS Virus] this term only 9
#14 MeSH descriptor: [Coronavirus Infections] this term only 137
#15 MeSH descriptor: [Severe Acute Respiratory Syndrome] this term only 107
#16 (((corona* or corono*) near/1 (virus* or viral* or virinae*)) or coronavirus* or coronovirus* or coronavirinae* or Coronavirus* or Coronovirus* or Wuhan* or Hubei* or Huanan or "2019-nCoV" or 2019nCoV or nCoV2019 or "nCoV-2019" or "COVID-19" or COVID19 or "CORVID-19" or CORVID19 or "WN-CoV" or WNCoV or "HCoV-19" or HCoV19 or CoV or "2019 novel*" or Ncov or "n-cov" or "SARS-CoV-2" or "SARSCoV-2" or "SARSCoV2" or "SARS-CoV2" or SARSCov19 or "SARS-Cov19" or "SARSCov-19" or "SARS-Cov-19" or Ncovor or Ncorona* or Ncorono* or NcovWuhan* or NcovHubei* or NcovChina* or NcovChinese*):ti,ab,kw 614
#17 ("severe acute respiratory syndrome" or SARS or "Middle East respiratory syndrome" or MERS):ti,ab,kw 350
#18 (betacoronavirus* or betacoronavirinae*):ti,ab,kw 4

Grove A, *et al. BMJ Open* 2021; 11:e043737. doi: 10.1136/bmjopen-2020-043737

#19 #8 or #9 or #10 or #11 or #12 or #13 or #14 or #15 or #16 or #17 or #18 798
#20 #7 and #19 3

[all 3 results were from CENTRAL]

<u>Update</u>
Search date: 10/06/2020
Re-ran search exactly as above and retrieved **5** results, all from CENTRAL. All 5 results exported to EndNote for deduplication.

Database of publications (living map of evidence) on coronavirus disease (COVID-19) developed by the University of Bern

Living Evidence on COVID-19
Contributors: Michel Counotte, Hira Imeri, Mert Ipekci, Nicola Low

https://zika.ispm.unibe.ch/assets/data/pub/ncov/

Search date: 10/05/2020 (14,988 entries)

Search: Title, Abstract
Search:

vitamin D 13
vitamin D2 0
vitamin D3 0
ergocalciferol 0
cholecalciferol 0
colecalciferol 0
25(OH)D 0
25OHD 0
25(OH)D3 0
25OHD3 0
hypovitaminosis D 1
Vitamin D Deficiency 1

Oxford COVID-19 Evidence Service
https://www.cebm.net/oxford-covid-19-evidence-service/
The Centre for Evidence-Based Medicine (CEBM) The University of Oxford

Search date: 10/05/2020 (142 articles)
vitamin D 1
vitamin D2 0
vitamin D3 0
ergocalciferol 0
cholecalciferol 0
colecalciferol 0
25(OH)D 0
25OHD 0

25(OH)D3 0
25OHD3 0
hypovitaminosis D 0
Vitamin D Deficiency 0

Database of publications on coronavirus disease (COVID-19) developed by WHO
https://www.who.int/emergencies/diseases/novel-coronavirus-2019/global-research-on-novel-coronavirus-2019-ncov

Search date: 10/05/2020 (15,253 entries)

Search: Title, Abstract, Subject

vitamin D 19
vitamin D2 0
vitamin D3 2
ergocalciferol 0
cholecalciferol 1
colecalciferol 0
25(OH)D 0
25OHD 0
25(OH)D3 0
25OHD3 0
hypovitaminosis D 1
Vitamin D Deficiency 2

Total: 25
After de-duplication: 20

Searches for systematic reviews, for reference checking

Medline

Search date: 19/05/2020
Database: Ovid MEDLINE(R) ALL <1946 to May 18, 2020>
Search Strategy:
----
1 exp Vitamin D/ (58577)
2 Vitamin D Deficiency/ (15588)
3 (vitamin D? or vit D? or D vitamin? or ergocalciferol or cholecalciferol or colecalciferol or calciferol or dihydroergocalciferol or sitocalciferol or dihydrotachysterol or dihydroxycholecalciferol? or hydroxycholecalciferol? or "25(OH)D?" or 25OHD? or calcidiol or hydroxyergocalciferol or alfacalcidol or alphacalcidol or "1,25(OH)2D" or hydroxyvitamin D? or dihydroxyvitamin D?).ab,kf,ti. (78395)
4 (paricalcitol or doxercalciferol or doxerocalciferol or calcifediol or calcitriol).ab,kf,ti. (5588)
5 hypovitaminosis D?.ab,kf,ti. (1780)
6 ((D or D2 or D3) adj3 supplement*).ab,kf,ti. (12198)
7 1 or 2 or 3 or 4 or 5 or 6 (92747)

8 coronavirus/ or betacoronavirus/ or betacoronavirus 1/ or coronavirus oc43, human/ or middle east respiratory syndrome coronavirus/ or sars virus/ (8161)
9 coronavirus infections/ or severe acute respiratory syndrome/ (11614)
10 (((corona* or corono*) adj1 (virus* or viral* or virinae*)) or coronavirus* or coronovirus* or coronavirinae* or Coronavirus* or Coronovirus* or Wuhan* or Hubei* or Huanan or "2019-nCoV" or 2019nCoV or nCoV2019 or "nCoV-2019" or "COVID-19" or COVID19 or "CORVID-19" or CORVID19 or "WN-CoV" or WNCoV or "HCoV-19" or HCoV19 or CoV or "2019 novel*" or Ncov or "n-cov" or "SARS-CoV-2" or "SARSCoV-2" or "SARSCoV2" or "SARS-CoV2" or SARSCov19 or "SARS-Cov19" or "SARSCov-19" or "SARS-Cov-19" or Ncovor or Ncorona* or Ncorono* or NcovWuhan* or NcovHubei* or NcovChina* or NcovChinese*).ab,kf,ti. (31115)
11 (severe acute respiratory syndrome or SARS or Middle East respiratory syndrome or MERS).ab,kf,ti. (17795)
12 (betacoronavirus* or betacoronavirinae*).ab,kf,ti. (294)
13 exp Respiratory Tract Infections/ (356696)
14 (acute respiratory infection* or severe respiratory infection* or acute respiratory tract infection* or severe respiratory tract infection* or influenza or common cold or pneumonia or bronchitis).ab,kf,ti. (234266)
15 8 or 9 or 10 or 11 or 12 or 13 or 14 (503079)
16 7 and 15 (1062)
17 (metaanalys* or "meta analys*" or "meta-analys*").tw. (169008)
18 (systematic* adj3 review*).mp. (200684)
19 meta analysis.pt. (114746)
20 17 or 18 or 19 (301767)
21 16 and 20 (55)

Embase

Search date: 19/05/2020

Database: Embase Classic+Embase <1947 to 2020 Week 20>
Search Strategy:
----
1 exp vitamin D/ (147053)
2 vitamin D deficiency/ (30106)
3 (vitamin D? or vit D? or D vitamin? or ergocalciferol or cholecalciferol or colecalciferol or calciferol or dihydroergocalciferol or sitocalciferol or dihydrotachysterol or dihydroxycholecalciferol? or hydroxycholecalciferol? or "25(OH)D?" or 25OHD? or calcidiol or hydroxyergocalciferol or alfacalcidol or alphacalcidol or "1,25(OH)2D" or hydroxyvitamin D? or dihydroxyvitamin D?).ab,kw,ti. (118981)
4 (paricalcitol or doxercalciferol or doxerocalciferol or calcifediol or calcitriol).ab,kw,ti. (8485)
5 hypovitaminosis D?.ab,kw,ti. (3033)
6 ((D or D2 or D3) adj3 supplement*).ab,kw,ti. (19335)
7 1 or 2 or 3 or 4 or 5 or 6 (172654)
8 betacoronavirus 1/ or betacoronavirus/ or human coronavirus oc43/ (1085)
9 Middle East respiratory syndrome coronavirus/ (2082)
10 sars-related coronavirus/ or sars coronavirus/ (6062)
11 Coronavirinae/ (2060)
12 coronavirus infection/ or middle east respiratory syndrome/ or severe acute respiratory syndrome/ (12565)

13 (((corona* or corono*) adj1 (virus* or viral* or virinae*)) or coronavirus* or coronovirus* or coronavirinae* or Coronavirus* or Coronovirus* or Wuhan* or Hubei* or Huanan or "2019-nCoV" or 2019nCoV or nCoV2019 or "nCoV-2019" or "COVID-19" or COVID19 or "CORVID-19" or CORVID19 or "WN-CoV" or WNCoV or "HCoV-19" or HCoV19 or CoV or "2019 novel*" or Ncov or "n-cov" or "SARS-CoV-2" or "SARSCoV-2" or "SARSCoV2" or "SARS-CoV2" or SARSCov19 or "SARS-Cov19" or "SARSCov-19" or "SARS-Cov-19" or Ncovor or Ncorona* or Ncorono* or NcovWuhan* or NcovHubei* or NcovChina* or NcovChinese*).ab,kw,ti. (30532)
14 (severe acute respiratory syndrome or SARS or Middle East respiratory syndrome or MERS).ab,kw,ti. (17954)
15 (betacoronavirus* or betacoronavirinae*).ab,kw,ti. (286)
16 exp respiratory tract infection/ (460049)
17 (acute respiratory infection* or severe respiratory infection* or acute respiratory tract infection* or severe respiratory tract infection* or influenza or common cold or pneumonia or bronchitis).ab,kw,ti. (329779)
18 8 or 9 or 10 or 11 or 12 or 13 or 14 or 15 or 16 or 17 (674800)
19 7 and 18 (3315)
20 (metaanalys* or "meta analys*" or "meta-analys*").mp. (294469)
21 (systematic* adj2 review*).mp. (330720)
22 20 or 21 (475492)
23 19 and 22 (219)
24 limit 19 to (meta analysis or "systematic review") (145)
25 23 or 24 (219)
26 limit 25 to (conference abstract or conference paper or "conference review" or editorial or letter) (41)
27 25 not 26 (178)

Cochrane Database of Systematic Reviews (Cochrane Library)

Search Name: Vitamin D Covid and Acute Respiratory Infections SRs
Date Run: 20/05/2020 18:30:28
Comment:

ID	Search	Hits
#1	MeSH descriptor: [Vitamin D] explode all trees	5224
#2	MeSH descriptor: [Vitamin D Deficiency] this term only	1226
#3	((vitamin NEXT D?) or (vit NEXT D?) or (D NEXT vitamin?) or ergocalciferol or cholecalciferol or colecalciferol or calciferol or dihydroergocalciferol or sitocalciferol or dihydrotachysterol or dihydroxycholecalciferol? or hydroxycholecalciferol? or "25(OH)D" or "25(OH)D3" or 25OHD? or calcidiol or hydroxyergocalciferol or alfacalcidol or alphacalcidol or "1,25(OH)2D" or (hydroxyvitamin NEXT D?) or (dihydroxyvitamin NEXT D?)):ti,ab,kw	12959
#4	(paricalcitol or doxercalciferol or doxerocalciferol or calcifediol or calcitriol):ti,ab,kw	2417
#5	hypovitaminosis NEXT D?	303
#6	((D OR D2 OR D3) NEAR/3 supplement*):ti,ab,kw	5632
#7	#1 or #2 or #3 or #4 or #5 or #6	14461
#8	MeSH descriptor: [Coronavirus] this term only	2
#9	MeSH descriptor: [Betacoronavirus] this term only	2
#10	MeSH descriptor: [Betacoronavirus 1] this term only	0
#11	MeSH descriptor: [Coronavirus OC43, Human] this term only	0
#12	MeSH descriptor: [Middle East Respiratory Syndrome Coronavirus] explode all trees	1

#13 MeSH descriptor: [SARS Virus] this term only 9
#14 MeSH descriptor: [Coronavirus Infections] this term only 137
#15 MeSH descriptor: [Severe Acute Respiratory Syndrome] this term only 107
#16 (((corona* or corono*) near/1 (virus* or viral* or virinae*)) or coronavirus* or coronovirus* or coronavirinae* or Coronavirus* or Coronovirus* or Wuhan* or Hubei* or Huanan or "2019-nCoV" or 2019nCoV or nCoV2019 or "nCoV-2019" or "COVID-19" or COVID19 or "CORVID-19" or CORVID19 or "WN-CoV" or WNCoV or "HCoV-19" or HCoV19 or CoV or "2019 novel*" or Ncov or "n-cov" or "SARS-CoV-2" or "SARSCoV-2" or "SARSCoV2" or "SARS-CoV2" or SARSCov19 or "SARS-Cov19" or "SARSCov-19" or "SARS-Cov-19" or Ncovor or Ncorona* or Ncorono* or NcovWuhan* or NcovHubei* or NcovChina* or NcovChinese*):ti,ab,kw 616
#17 ("severe acute respiratory syndrome" or SARS or "Middle East respiratory syndrome" or MERS):ti,ab,kw 351
#18 (betacoronavirus* or betacoronavirinae*):ti,ab,kw 4
#19 MeSH descriptor: [Respiratory Tract Infections] explode all trees 14360
#20 (("acute respiratory" NEXT infection*) or ("severe respiratory" NEXT infection*) or ("acute respiratory tract" NEXT infection*) or ("severe respiratory tract" NEXT infection*) or influenza or "common cold" or pneumonia or bronchitis):ti,ab,kw 25944
#21 #8 or #9 or #10 or #11 or #12 or #13 or #14 or #15 or #16 or #17 or #18 or #19 or #20 32554
#22 #7 and #21 329

CDSR: 3

Expert consultation

One additional study identified:

Martineau AR, Jolliffe DA, Hooper RL, et al., (2017) Vitamin D supplementation to prevent acute respiratory tract infections: systematic review and meta-analysis of individual participant data. BMJ. 2017;356:i6583. doi:10.1136/bmj.i6583

2. Full details of the study eligibility criteria

Include	Exclude
**P- Population**<br><br>1) Patients acutely ill with Betacoronavirus infection [SARS-CoV, MERS-CoV, SARS-CoV-2]<br>2) or at risk of acute illness with Betacoronavirus infection<br><br>**I – Intervention/exposure**<br><br>1) Vitamin D supplementation<br>2) Low Serum Vitamin D<br><br>**O - Outcomes**<br><br>1) Betacoronavirus infection (to include serological evidence of infection or clinically confirmed symptomatic infection);<br>2) severity of Betacoronavirus infection (to include patients admitted to hospital or admitted to intensive care); mortality due to Betacoronavirus.<br>3) Mortality due to Betacoronavirus<br><br>**C – Comparator**<br><br>1) No Vitamin D supplementation<br>2) high or normal Serum Vitamin D<br><br>**S** - **Study design**<br>Randomised controlled trials and non-randomized studies will be eligible for inclusion in the review including, non randomized controlled trials, interrupted time series, controlled before-and-after studies, cohort studies, ecological studies, case reports and case series.<br><br>**Subgroups**<br><br>1. Ethnicity characteristics (White British, All Other White, Mixed, Asian, Black, Other)<br>2. Age characteristics (population by five-year age groups)	Animals studies, modelling studies<br><br>Qualitative studies, Non-primary research- reviews, editorials etc, guidelines and non-systematic reviews.<br><br>Non-English language. Non peer reviewed publication.

3. List of studies excluded at full text review

Excluded studies		Reason<br><br>*Non-eligible study design- e.g. review*<br>*Non-eligible population*<br>*Non-eligible intervention*<br>*No relevant outcome*<br>*No comparator group*
1	Adams, K. K., et al. (2020). "Myth Busters: Dietary Supplements and COVID-19." Annals of Pharmacotherapy: 1060028020928052.	Study design – commentary
2	Ahmed, I., et al. (2020). "First Covid-19 maternal mortality in the UK associated with thrombotic complications." British Journal of Haematology. **18**.	Study design – letter
3	Alpalhao, M. and P. Filipe (2020). "SARS-CoV-2 pandemic and Vitamin D deficiency - a double trouble." Photodermatology, Photoimmunology & Photomedicine **01**: 01.	Study design – letter
4	Annweiler, C., et al. (2020). COvid-19 and Vitamin D Supplementation: a Multicenter Randomized Controlled Trial of High Dose Versus Standard Dose Vitamin D3 in High-risk COVID-19 Patients (CoVitTrial). https://clinicaltrials.gov/show/NCT04344041.	Study design – on-going RCT completion date. July 2020
5	Arya, A. and V. D. Dwivedi (2020). "Synergistic effect of vitamin D and remdesivir can fight COVID-19." Journal of Biomolecular Structure & Dynamics: 1-2	Study design – letter
6	Banerjee, D., et al. (2020). "COVID-19 infection in kidney transplant recipients." Kidney International **97**(6): 1076-1082.	Study design – commentary
7	Caccialanza, R., et al. (2020). Early nutritional supplementation in non-critically ill patients hospitalized for the 2019 novel coronavirus disease (COVID-19): Rationale and feasibility of a shared pragmatic protocol. Nutrition: 110835.	Study design - protocol
8	Calder, P. C., et al. (2020). Optimal Nutritional Status for a Well-Functioning Immune System Is an Important Factor to Protect against Viral Infections. Nutrients: 12(4), 1181.	Study design -narrative review
9	Cao, Z., et al. (2020). SARS-CoV-2 & Covid-19: Key-Roles of the 'Renin-Angiotensin' System / Vitamin D Impacting Drug and Vaccine Developments. Infectious Disorders - Drug Targets. *(E-pub Ahead of Print)* DOI : 10.2174/1871526520999200505174704	Study design – editorial
10	Carter, S. J., et al. (2020). Considerations for obesity, vitamin D, and physical activity amidst the COVID-19 pandemic. Obesity **16**: 16.	Study design -narrative review

Grove A, *et al. BMJ Open* 2021; 11:e043737. doi: 10.1136/bmjopen-2020-043737

11	Daneshkhah, A., et al. (2020). The Possible Role of Vitamin D in Suppressing Cytokine Storm and Associated Mortality in COVID-19 Patients. medRxiv. DOI: https://doi.org/10.1101/2020.04.08.20058578	Study design – modelling
12	Davies G, Garami AR, Byers JC. Evidence Supports a Causal Model for Vitamin D in COVID-19 Outcomes. medRxiv, 2020. DOR: https://doi.org/10.1101/2020.05.01.20087965v3	Study design – modelling
13	de Lucena, T. M. C., et al. (2020). "Mechanism of inflammatory response in associated comorbidities in COVID-19." Diabetes & Metabolic Syndrome **14**(4): 597-600.	Study design -narrative review
14	Eroglu, C., et al. (2019). The relation between serum vitamin D levels, viral infections and severity of attacks in children with recurrent wheezing. Allergologia et Immunopathologia **47**(6): 591-597.	Population - Not COVID-19/SARs/MERs
15	Faul, J. L., et al. (2020). "Vitamin d deficiency and ards after sars-cov-2 infection." Irish Medical Journal **113**(5).	Study design – letter
16	Ghasemian, R., et al. (2020). "The Role of Vitamin D in The Age of COVID-19: A Systematic Review and Meta-Analysis Along with an Ecological Approach." MedRxiv : the Preprint Server for Health Sciences. DOI: 10.1101/2020.06.05.20123554	Study design -narrative review
17	Grant, W. B., et al. (2020). Evidence that Vitamin D Supplementation Could Reduce Risk of Influenza and COVID-19 Infections and Deaths. Nutrients **12**(4): 02.	Study design -narrative review
18	Heiser, K., et al. (2020). Identification of potential treatments for COVID-19 through artificial intelligence-enabled phenomic analysis of human cells infected with SARS-CoV-2. bioRxiv: 2020.2004.2021.054387.	Study design – modelling
19	Hribar, C. A., et al. (2020). "Potential Role of Vitamin D in the Elderly to Resist COVID-19 and to Slow Progression of Parkinson's Disease." Brain Sciences **10**(5): 08.	Study design -narrative review
20	Jakovac, H. (2020). COVID-19 and vitamin D-Is there a link and an opportunity for intervention? American Journal of Physiology - Endocrinology & Metabolism **318**(5): E589-E589.	Study design – letter
21	Jamaati, H., et al. (2020). A fourteen-day experience with coronavirus disease 2019 (COVID-19) induced acute respiratory distress syndrome (ARDS): An Iranian treatment protocol. Iranian Journal of Pharmaceutical Research **19**(1): 31-36.	Study design – treatment protocol/recommendation
22	Kalippurayil Moozhipurath, R., et al. (2020). "Evidence of Protective Role of Ultraviolet-B (UVB) Radiation in	Exposure – not vitamin D(UVB)

	Reducing COVID-19 Deaths." MedRxiv : the Preprint Server for Health Sciences. DOI 10.1101/2020.05.06.20093419	
23	Kara, M., et al. (2020). "'Scientific Strabismus' or Two Related Pandemics: COVID-19 & Vitamin D Deficiency." British Journal of Nutrition: 1-20.	Study design -narrative review
24	Koivisto, O., et al. (2020). Key Vitamin D Target Genes with Functions in the Immune System. Nutrients, **12**(4):1140.	Population - Outcomes - target genes
25	Kow, C. S., et al. (2020). "Vitamin D Supplementation in Influenza and COVID-19 Infections Comment on: "Evidence that Vitamin D Supplementation Could Reduce Risk of Influenza and COVID-19 Infections and Deaths" Nutrients 2020, 12(4), 988." Nutrients **12**(6): 01.	Study design – commentary
26	Kumar, V. and A. Srivastava (2020). "Spurious Correlation? A review of the relationship between Vitamin D and Covid-19 infection and mortality." MedRxiv : the Preprint Server for Health Sciences. DOI: 10.1101/2020.05.25.20110338	Study design -narrative review
27	Laird, E., et al. (2020). "Vitamin D and inflammation: Potential implications for severity of Covid-19." Irish Medical Journal **113**(5).	Study design -narrative review
28	La Vignera, S., et al. (2020). Sex-Specific SARS-CoV-2 Mortality: Among Hormone-Modulated ACE2 Expression, Risk of Venous Thromboembolism and Hypovitaminosis D. International journal of molecular sciences **21**(8):2948.	Study design – editorial
29	Li, A. Y., et al. (2020). Multivariate Analysis of Factors Affecting COVID-19 Case and Death Rate in U.S. Counties: The Significant Effects of Black Race and Temperature. medRxiv. DOI: https://doi.org/10.1101/2020.04.17.20069708	Intervention – not Vitamin D
30	Maestri, E., et al. (2020). Vitamin D and coronavirus: a new field of use?. Recenti Progressi in Medicina **111**(4): 253-256.	Study design -narrative review, non-English
31	Mansbach, J. M. and C. A. Camargo Jr., (2009). Respiratory Viruses in Bronchiolitis and Their Link to Recurrent Wheezing and Asthma. Clinics in Laboratory Medicine **29**(4): 741-755.	Population - Not COVID-19/SARs/MERs
32	Marik, P. E., et al. (2020). Does vitamin D status impact mortality from SARS-CoV-2 infection? Medicine in Drug Discovery: 100041-100041.	Study design - commentary
33	McKenna, M. J. and M. A. T. Flynn (2020). "Covid-19, cocooning and vitamin d intake requirements." Irish Medical Journal **113**(5).	Study design -narrative review

34	Mitchell, F. (2020). "Vitamin-D and COVID-19: do deficient risk a poorer outcome?" The Lancet Diabetes & Endocrinology **20**: 20.	Study design -narrative review
35	Molloy, E. J. and N. Murphy (2020). Vitamin D, Covid-19 and Children. Irish Medical Journal **113**(4): 64.	Study design -narrative review
36	McCartney, D. M. and D. G. Byrne (2020). Optimisation of Vitamin D Status for Enhanced Immuno-protection Against Covid-19. Irish Medical Journal **113**(4): 58.	Study design -narrative review
37	Rabbitt, L. and E. Slattery (2020). "Vitamin d and covid-19: A note of caution." Irish Medical Journal **113**(5).	Study design - letter
38	Ribeiro, H., et al. (2020). "Does Vitamin D play a role in the management of Covid-19 in Brazil?" Revista de Saude Publica **54**: 53.	Study design -narrative review
39	Romano, L., et al. (2020). "Short Report - Medical nutrition therapy for critically ill patients with COVID-19." European Review for Medical and Pharmacological Sciences **24**(7): 4035-4039.	Study design -narrative review
40	Silberstein, M. (2020). Vitamin D: A simpler alternative to tocilizumab for trial in COVID-19? Medical Hypotheses **140**: 109767-109767.	Study design -narrative review
41	Speeckaert, M. M. and J. R. Delanghe (2020). "Association between low vitamin D and COVID-19: don't forget the vitamin D binding protein." Aging Clinical & Experimental Research **28**: 28.	Study design - letter
42	Skutsch, M., et al. (2020). "The association of UV with rates of COVID-19 transmission and deaths in Mexico: the possible mediating role of vitamin D." MedRxiv : the Preprint Server for Health Sciences. DOI: 10.1101/2020.05.25.20112805	Study design – modelling
43	Suresh, P. S. (2020). "Hypovitaminosis D and COVID-19: Matter of Concern in India?" Indian Journal of Clinical Biochemistry.	Study design - letter
44	Taghizadieh, A., et al. (2020). "Acute kidney injury in pregnant women following SARS-CoV-2 infection: A case report from Iran." Respiratory Medicine Case Reports 30	Exposure – not vitamin D
45	Tan, S. H. S., et al. (2020). "Medications in COVID-19 patients: summarizing the current literature from an orthopaedic perspective." International Orthopaedics **22**: 22.	Study design -narrative review
46	Yalaki, Z., et al. (2019). Comparison of viral agents and vitamin D levels in children with acute bronchiolitis infection. Cocuk Enfeksiyon Dergisi **13**(1): e14-e20.	Population - COVID-19/SARs/MERs not specified
47	Zabetakis, I., et al. (2020). "COVID-19: The Inflammation Link and the Role of Nutrition in Potential Mitigation." Nutrients **12**(5): 19.	Study design -narrative review

48	Zemb, P., et al. (2020). "Vitamin D deficiency and COVID-19 pandemic." Journal of Global Antimicrobial Resistance **28**: 28.	Study design - commentary

4. Articles included at full text, but later excluded at time of narrative synthesis

	Citation record	Exclusion reason	Update performed 8th October 2020
49	Darling, A. L., et al. (2020). Vitamin D status, body mass index, ethnicity and COVID-19: Initial analysis of the first-reported UK Biobank COVID-19 positive cases (n 580) compared with negative controls (n 723). medRxiv.<br><br>https://www.medrxiv.org/content/10.1101/2020.04.29.20084277v1?versioned=TRUE	Not peer reviewed publication at time of narrative synthesis	No update available
50	De Smet, D., et al. (2020). Vitamin D deficiency as risk factor for severe COVID-19: a convergence of two pandemics. medRxiv.<br><br>https://www.medrxiv.org/content/10.1101/2020.05.01.20079376v2	Not peer reviewed publication at time of narrative synthesis	No update available
51	Lau, F. H., et al. (2020). Vitamin D Insufficiency is Prevalent in Severe COVID-19. medRxiv.<br><br>https://www.medrxiv.org/content/10.1101/2020.04.24.20075838v1?versioned=TRUE	Not peer reviewed publication at time of narrative synthesis	No update available
52	Meltzer, D. O., et al. (2020). "Association of Vitamin D Deficiency and Treatment with COVID-19 Incidence." MedRxiv : the Preprint Server for Health Sciences **13**: 13.	Not peer reviewed public	An updated publication is available at https://jamanetwork.com/journals/jamanetworkopen/fullarticle/2770157<br><br>Citation

	https://www.medrxiv.org/content/10.1101/2020.05.08.20095893v1	ation at time of narrative synthesis	Meltzer DO, Best TJ, Zhang H, Vokes T, Arora V, Solway J. Association of Vitamin D Status and Other Clinical Characteristics With COVID-19 Test Results. *JAMA Netw Open.* 2020;3(9):e2019722. doi:10.1001/jamanetworkopen.2020.19722
53	Notari, A. and G. Torrieri (2020). "COVID-19 transmission risk factors." MedRxiv : the Preprint Server for Health Sciences.<br><br>https://www.medrxiv.org/content/10.1101/2020.05.08.20095083v1?versioned=TRUE	Not peer reviewed publication at time of narrative synthesis	No update available
54	Raisi-Estabragh, Z., et al. (2020). "Greater risk of severe COVID-19 in non-White ethnicities is not explained by cardiometabolic, socioeconomic, or behavioural factors, or by 25(OH)-vitamin D status: study of 1,326 cases from the UK Biobank." MedRxiv : the Preprint Server for Health Sciences.<br><br>https://www.medrxiv.org/content/10.1101/2020.06.01.20118943v1?versioned=TRUE	Not peer reviewed publication at time of narrative synthesis	An updated publication is available at https://academic.oup.com/jpubhealth/article/42/3/451/5859581<br><br>Citation<br>Zahra Raisi-Estabragh, Celeste McCracken, Mae S Bethell, Jackie Cooper, Cyrus Cooper, Mark J Caulfield, Patricia B Munroe, Nicholas C Harvey, Steffen E Petersen, Greater risk of severe COVID-19 in Black, Asian and Minority Ethnic populations is not explained by cardiometabolic, socioeconomic or behavioural factors, or by 25(OH)-vitamin D status: study of 1326 cases from the UK Biobank, *Journal of Public Health*, Volume 42, Issue 3, September 2020, Pages 451–460, https://doi.org/10.1093/pubmed/fdaa095
55	Tan, C. W., et al. (2020). "A cohort study to evaluate the effect of combination Vitamin D, Magnesium and Vitamin B12 (DMB) on progression to severe outcome in older COVID-19 patients." MedRxiv : the Preprint Server for Health Sciences.<br><br>https://www.medrxiv.org/content/10.1101/2020.06.01.20112334v2	Not peer reviewed publication at time of narrative synthesis	No update available

Study details of the seven articles included at full text, but excluded at time of narrative synthesis						
**Study**	**Design/ setting**	**Population**	**Exposure/Intervention**	**Outcomes**	**Results**	**Limitations**
Darling, A. L., et al. (2020)[1]	Retrospective cohort study<br><br>UK Biobank England cohort only	COVID-19 positive cases (n 580) Mean age 57.5 (SD 9.7)<br><br>COVID-19 negative controls (n 723) Mean age 57.9 (SD 8.7)	Serum 25(OH)D status Median (IQR) nmol/L<br><br>by gender (Male/Female), body mass index (Normal/underweight, overweight, obesity), ethnicity (Asian, Black, Mixed and Other, White)	COVID-19 test result	Serum 25(OH)D status similar in both groups:<br><br>COVID-19 positive cases (median IQR) = 43.3 (32.1) nmol/L)<br><br>COVID-19 negative controls (median (IQR) 44.1 (31.2) nmol/L) for COVID-19.<br><br>A logistic regression model suggests that being overweight (OR 1.51 CI 1.13-2.02) or obese (OR 1.67 CI 1.24-2.26); living in London (OR 1.45 CI 1.05-2.00); being male (OR 1.28 CI 1.01-1.61) and being of Asian, Black or Mixed ethnicity (OR 1.66 CI 1.08-2.54) is associated with a higher odds of testing positive for COVID-19	UK Biobank baseline samples collected in 2006-2010.
De Smet, D., et al. (2020)[2]	Retrospective observational study<br><br>Central network hospital, West Flanders, Belgium	186 SARS-CoV-2 infected patients hospitalised from March 1, 2020 to April 7, 2020<br><br>(109 males [median age 68 years, IQR 53-79] 77 females [median age 71 years, IQR 65-74])<br><br>25(OH)D in COVID-19 patients was compared a control group of 2717 patients with similar age distribution, sampled from March 1, 2019 to April 30, 2019. (999 males [median age 69 years, IQR 53-81] and 1718 females [median age 68 years, IQR 43-83]).	25(OH)D levels	SARS-CoV-2 infection	COVID-19 patients had a lower median 25(OH)D on admission (18.6 ng/mL, IQR 12.6-25.3) than controls (21.5 ng/mL, IQR 13.9-20.8, P=0.0016) and a higher percentage of vitamin D deficiency (defined as 25(OH)D < 20ng/mL): 58.6% versus 45.2% (P=0.0005).<br><br>In male COVID-19 patients, vitamin D deficiency was lower median 25(OH)D (17.6 ng/mL, IQR 12.7-24.0 versus 20.3 ng/mL, IQR 13.7-28.3, P=0.0234) and a higher deficiency rate (67.0% versus 49.2%, P=0.0006) than male controls.	The prevalence and age/sex/ seasonal-distribution of vitamin D status was derived from the general population sampled from 16274 consecutive, unselected and unique patients from January 1, 2019 to December 31, 2019.

Grove A, *et al. BMJ Open* 2021; 11:e043737. doi: 10.1136/bmjopen-2020-043737

Lau, F. H., et al. (2020)[3]	Retrospective observational study<br><br>A single, tertiary care academic (university) medical centre, Louisiana, New Orleans, USA	COVID-19 ICU patients (n 13) Mean age 61.5 (SD 15.7)<br><br>COVID-19 floor patients (n 7) Mean age 72.0 (SD 14.8)<br><br>Medical records of COVID-19 patients between March 27, 2020 and April 21, 2020<br><br>Vitamin D insufficiency (VDI) and COVID-19 metrics in ICU vs. floor patients	VDI: defined as serum 25(OH) D < 30 ng/mL)<br><br>Serum 25(OH) D status Mean (SD) ng/mL<br><br>by gender (Male/Female), body mass index (Normal, obesity), race (African American), age (elderly>65 years), hypertension	COVID-19 metrics	Overall, few significant differences were identified between ICU and floor patients: Lactate dehydrogenase was significantly higher among ICU patients (441.8 vs. 223.0, P=0.001). Also, body mass index was significantly higher among ICU patients (35.2 vs. 24.5, P=0.02).<br><br>Among ICU subjects, 11 (84.6%) had VDI, vs. 4 (57.1%) of floor subjects. 100% of ICU patients less than 75 years old had VDI (n=11). Among these, 64.6% (n=7) had critically low 25(OH) D (<20 ng/mL) and 3 had <10 ng/mL.<br><br>VDI is highly prevalent in dark-skinned people (82.1% of African Americans vs. 41.6% overall).<br><br>Male/Female ratio was 1.24 and 1.44 for COVID-19 and VDI respectively.	Statistical analysis was limited by the small number of subjects.
Meltzer, D. O., et al. (2020)[4]	Retrospective cohort study<br><br>University of Chicago Medicine, USA	4,314 patients tested for COVID-19 from 3/3/2020 to 4/10/2020. Among these, 499 had a vitamin D level in the year before testing.<br><br>COVID-19 positive cases with vitamin D deficient (n 178) Mean age 45.6<br><br>COVID-19 positive cases with not vitamin D deficient (n 321) Mean age 50.7	Vitamin D deficiency: defined by the most recent 25(OH) D <20ng/ml or 1,25-dihydroxycholecalciferol <18pg/ml within 1 year before COVID-19 testing. Treatment: defined by the most recent vitamin D type and dose, and treatment changes between the time of the most recent vitamin D level and time of COVID-19 testing<br><br>Vitamin D deficiency and treatment changes were combined to categorize vitamin D status at the time of COVID-19 testing as:<br>1)Likely deficient (last-level-deficient/treatment-not-increased)	Testing positive for COVID-19	In multivariable analysis, testing positive for COVID-19 was associated with increasing age (RR (age<50)=1.05, P<0.021; RR (age≥50)=1.02, P<0.064)), non-white race (RR=2.54, P<0.01) and being likely vitamin D deficient (deficient/treatment-not-increased: RR=1.77, P<0.02) as compared to likely vitamin D sufficient (not-deficient/treatment-not-decreased), with predicted COVID-19 rates in the vitamin D deficient group of 21.6% (95%CI [14.0%-29.2%]) vs 12.2% (95%CI [8.9%-15.4%]) in the vitamin D sufficient group.<br><br>Vitamin D deficiency declined with increasing vitamin D dose (especially of vitamin D3). Vitamin D dose was not significantly associated with testing positive for COVID-19 (P=0.18).	The associations observed might not reflect causal effects of vitamin D deficiency on COVID-19. This is because vitamin D deficiency can reflect a range of chronic health conditions or behavioural factors which plausibly decrease the likelihood of treatment of vitamin D

			2)Likely sufficient (last-level-not-deficient/treatment-not-decreased)<br>3)Uncertain deficiency (last-level-deficient/treatment-increased or last-level-not-deficient/treatment-decreased)<br><br>by age (<50, ≥50), gender (Male/Female), race (White, other than White), ethnicity (Hispanic, not Hispanic), body mass index, employee status, comorbidity indicators (e.g. hypertension)			deficiency and increase COVID-19 risk.
Notari, A. and G. Torrieri (2020)[5]	Correlational study<br><br>126 countries, Only 50 countries for vitamin D	The number of cases follows in its early stages an almost exponential expansion. A starting point in each country was chosen: the first day di with 30 cases and fitted for 12 days. Thus, capturing the early exponential growth.<br><br>Countries with too small total population (less than 300 thousands inhabitants) were excluded.	They analysed risk factors correlated with the initial transmission growth rate of COVID-19<br><br>Average annual level of serum Vitamin D and the seasonal level<br><br>The seasonal level is defined as: the amount during March or during winter for northern hemisphere, or during summer for southern hemisphere or the annual level for countries with little seasonal variation.	Growth rate of COVID-19	They looked for linear correlations of the exponents with other variables, for a sample of 126 countries.<br><br>They found a positive correlation, i.e. faster spread of COVID-19, with high confidence level with the following variables, with respective p-value: low Temperature (4.10^{-7}), high ratio of old vs. working-age people (3.10^{-6}), life expectancy (8.10^{-6}), number of international tourists (1.10^{-5}), earlier epidemic starting date di (2.10^{-5}), high level of physical contact in greeting habits (6.10^{-5}), lung cancer prevalence (6.10^{-5}), obesity in males (1.10^{-4}), share of population in urban areas (2.10^{-4}), cancer prevalence (3.10^{-4}), alcohol consumption (0.0019), daily smoking prevalence (0.0036), UV index (0.004, smaller sample, 73 countries), low Vitamin D serum levels (0.002-0.006, smaller sample, 50 countries). There is highly significant correlation also with blood type. Also, positive correlation with moderate CI (p-value of 0.02-0.03) with: CO2/SO emissions, type-1 diabetes in children, and low vaccination coverage for Tuberculosis (BCG).	The dataset for the annual vitamin D was built with the available literature, which is quite inhomogeneous.<br><br>The dataset for the seasonal levels is more restricted. This is because the relative literature is less complete. So, for this the authors have included only 42 countries.

Grove A, *et al. BMJ Open* 2021; 11:e043737. doi: 10.1136/bmjopen-2020-043737

					Vitamin D is not highly correlated with UV index due to different food consumption in different countries.	
Raisi-Estabragh, Z., et al. (2020)[6]	Retrospective cohort study<br><br>UK Biobank	4,510 UK participants tested for COVID-19.<br><br>Latest data release (29/05/2020) includes test results from 16/03/2020 to 18/05/2020.<br><br>COVID-19 positive cases (n 1,326) Mean age 68.11 (SD 9.23)<br><br>COVID-19 negative controls (n 3,184) Mean age 68.91 (SD 8.72)	Serum 25(OH) D levels nmol/L<br><br>Multivariate logistic regression models by age, gender (Male/Female), ethnicity (Caucasian (any White background) and non-Caucasian: Black, Asian, Chinese) to test whether addition of: 1)cardio metabolic factors (e.g. hypertension, body mass index); 2) 25(OH)-vitamin D; 3) poor diet; 4) Townsend deprivation score; 5) housing; or 6)behavioural factors attenuated sex/ethnicity associations with COVID-19 status	COVID-19 test result<br><br>Greater risk of severe COVID-19	Over-representation of men and non-White ethnicities in the COVID-19 positive group.<br><br>Non-Whites had, on average, poorer cardio metabolic profile, lower 25(OH)-vitamin D, greater material deprivation, and were more likely to live in larger households and flats.<br><br>Male sex, non-White ethnicity, higher body mass index, Townsend deprivation score, and household overcrowding were independently associated with significantly greater odds of COVID-19. The pattern of association was consistent for men and women; cardio metabolic, socio-demographic and behavioural factors did not attenuate sex/ethnicity associations.	UK Biobank baseline samples collected in 2006-2010.<br><br>Aggregating all Black and Minority Ethnic (BAME) populations into one cohort might overlook important differences between non-Caucasian ethnicities.<br><br>The current dataset does not allow assessment of specific COVID-19 health outcomes.
Tan, C. W., et al. (2020)[7]	Cohort observational study<br><br>A tertiary academic hospital, Singapore	All 43 consecutive hospitalized COVID-19 patients aged 50 and above.<br><br>Between 15 January and 15 April 2020.	DMB = a single daily oral dose of vitamin D3 1000 IU, magnesium 150mg and vitamin B12 500mcg for up to 14 days<br><br>Adjusted for age, gender and comorbidities	Deterioration post-DMB administration leading to any form of oxygen therapy and/or intensive care	Duration of therapy: days, Median 5 (IQR 4-7)<br><br>Significantly fewer DMB patients than controls required initiation of oxygen therapy subsequently throughout their hospitalization (17.6% vs 61.5%, P=0.006).<br><br>On univariate analysis, increasing age and presence of comorbidities were associated	Small sample size, and the lack of systematic biologic measures to support their findings.

		17 patients received Vitamin D, Magnesium, Vitamin B12 (DMB): Mean age 58.4 (SD 7.0)<br><br>26 patients did not: Mean age 64.1 (SD 7.9)		support for COVID-19 patients	with significantly higher OR for oxygen therapy, while exposure to DMB therapy was associated with a significantly improved OR 0.13 (95% CI: 0.03 – 0.59, P=0.008) .<br><br>On multivariate analysis, increasing age was associated with significantly higher OR for oxygen therapy, while exposure to DMB therapy was associated with a significantly improved OR 0.15 (95% CI: 0.025 – 0.93, P=0.041).	

Grove A, *et al. BMJ Open* 2021; 11:e043737. doi: 10.1136/bmjopen-2020-043737

5. Risk of bias of included studies

Risk of bias assessment using the Downs and Black Checklist[8]

Study	Quality score	Reviewer notes
Hastie et al., 2020[9]	14/20<br><br>Seven domains were not applicable and therefore not assessed, 2 reporting, 1 external validity 3 internal validity (bias) and 1 internal validity (confounding).	The study could not be scored for 3 questions as we were unable to determine; 1) the representativeness of the subjects who were prepared to participate from entire population from which they were recruited, 2) whether losses to follow-up were taken into account as patients lost to follow-up were not reported and 3) whether the study had sufficient power to detect a clinically important effect.<br><br>The study did not score a point for 3 questions; 1) providing the number and a description of the characteristics of patients lost to follow-up, 2) stating whether study subjects in different intervention groups we recruited over the same period of time and 3) for assignment concealment as it was a non-randomised study. The study scored partially (only 1 point not two) for clearly described distributions of principal confounders in each group of subjects to be compared.
D'Avolio et al, 2020[10]	13/15<br><br>Twelve domains were not applicable therefore not assessed, 3 reporting, 1 external validity, 4 internal validity (bias) and 4 internal validity (confounding).	The study could not be scored for the 'power' domain as we were unable to determine from the article whether the study had sufficient power to detect a clinically important effect where the probability value for a difference being due to chance is less than 5%.<br><br>The study did not score 1 point in the 'external validity' domain as those subjects who were prepared to participate were not representative of the entire population from which they were recruited.
Fasano et al., 2020[11]	12/17<br>Ten domains were not applicable therefore not assessed, 3 reporting, 1 external validity, 4 internal validity (bias) and 2 internal validity (confounding).	The study could not be scored for 4 items, the 'power' domain and one question in the 'Internal validity - confounding (selection bias)' as the study did not specify the time period over which patients were recruited. It could also not be scored for 2 questions in the 'External validity domain', 1) the representativeness of the subjects asked to participate in the study compared to the entire population from which they were recruited and 2) the representativeness of those subjects who were prepared to participate compared to the entire population from which they were recruited.<br><br>The study did not score 1 point as the effect of the main confounders was not investigated or confounding was demonstrated but no adjustment was made in the final analyses. The study scored two points for presentation of potential confounders.

Grove A, *et al. BMJ Open* 2021; 11:e043737. doi: 10.1136/bmjopen-2020-043737

Ilie et al., 2020[12]	4/20<br>Seven domains were not applicable and therefore not assessed, 1 reporting, 1 external validity, 3 internal validity (bias), 1 internal validity (confounding) and 1 for power.	The study could not be scored for 9 questions. Two in the 'reporting' domain, 1) interventions of interest not clearly described, 2) the main findings of the study are not clearly described. Two 'External validity' questions 1) the representativeness of the subjects asked to participate in the study compared to the entire population from which they were recruited and 2) the representativeness of those subjects who were prepared to participate compared to the entire population from which they were recruited. Two 'Internal validity – bias' domain questions 1) all analyses that had not been planned at the outset of the study were not clearly indicated (results of the study based on "data dredging", were not made clear), and 2) it was not clear is the statistical techniques used were appropriate to the data. Three 'Internal validity - confounding (selection bias)' domain questions, 1) no information provided concerning the source of patients included in the study 2) does not specify the time period over which patients were recruited, and 3) the numbers of patients lost to follow-up are not reported.<br><br>The study did not score 7 points for the following; 3 reporting issues 1) no description of the characteristics of participants included in the study 2) no description of the distributions of principal confounders in each group of subjects to be compared, and 3) no description of the characteristics of patients lost to follow-up. Two internal validity bias issues 1) differences in follow-up were ignored and 2) no evidence that the main measure used were accurate (valid and reliable).
**Note:** For each included study, the maximum possible quality score was dependent on which domains could be assessed based on the study design. The higher the score assigned to a study, the lower the risk of bias. For example, Hastie et al. 2020[9] was assigned a score of 14 out of a maximum possible score of 20, suggesting good quality and therefore low risk of bias compared to the other studies.		

References

1. Darling AL, Ahmadi KR, Ward KA, et al. Vitamin D status, body mass index, ethnicity and COVID-19: initial analysis of the first-reported UK Biobank COVID-19 positive cases (n 580) compared with negative controls (n 723). *medRxiv* 2020. doi: 10.1101/2020.04.29.20084277
2. De Smet D, De Smet K, Herroelen P, Gryspeerdt S, Martens GA. Vitamin D deficiency as risk factor for severe COVID-19: a convergence of two pandemics. *medRxiv* 2020. doi: 10.1101/2020.05.01.20079376
3. Lau FH, Majumder R, Torabi R, et al. Vitamin D insufficiency is prevalent in severe COVID-19. *medRxiv* 2020. doi: 10.1101/2020.04.24.20075838

4. Meltzer DO, Best TJ, Zhang H, Vokes T, Arora V, Solway J. Association of vitamin D deficiency and treatment with COVID-19 incidence. *medRxiv* 2020. doi: 10.1101/2020.05.08.20095893
5. Notari A, Torrieri G. COVID-19 transmission risk factors. *medRxiv* 2020. doi: 10.1101/2020.05.08.20095083
6. Raisi-Estabragh Z, McCracken C, Bethell MS, et al. Greater risk of severe COVID-19 in non-White ethnicities is not explained by cardiometabolic, socioeconomic, or behavioural factors, or by 25(OH)-vitamin D status: study of 1,326 cases from the UK Biobank. *medRxiv* 2020. doi: 10.1101/2020.06.01.20118943
7. Tan CW, Ho LP, Kalimuddin S, et al. A cohort study to evaluate the effect of combination vitamin D, magnesium and vitamin B12 (DMB) on progression to severe outcome in older COVID-19 patients. *medRxiv* 2020. doi: 10.1101/2020.06.01.20112334
8. Downs SH, Black N. The feasibility of creating a checklist for the assessment of the methodological quality both of randomised and non-randomised studies of health care interventions. *J Epidemiol Community Health* 1998;52(6):377-84. doi: 10.1136/jech.52.6.377
9. Hastie CE, Mackay DF, Ho F, et al. Vitamin D concentrations and COVID-19 infection in UK Biobank. *Diabetes Metab Syndr* 2020;14(4):561-65. doi: 10.1016/j.dsx.2020.04.050
10. D'Avolio A, Avataneo V, Manca A, et al. 25-hydroxyvitamin D concentrations are lower in patients with positive PCR for SARS-CoV-2. *Nutrients* 2020;12(5):1359. doi: 10.3390/nu12051359
11. Fasano A, Cereda E, Barichella M, et al. COVID-19 in Parkinson's Disease Patients Living in Lombardy, Italy. *Mov Disord* 2020. doi: 10.1002/mds.28176
12. Ilie PC, Stefanescu S, Smith L. The role of vitamin D in the prevention of coronavirus disease 2019 infection and mortality. *Aging Clin Exp Res* 2020. doi: 10.1007/s40520-020-01570-8
