# Supplementary material for: Association between vitamin D supplementation or serum vitamin D level and susceptibility to SARS-CoV-2 infection or COVID-19 including clinical course, morbidity and mortality outcomes? A systematic review
Source: BMJ Open. 2021 May 28;11(5):e043737. doi: 10.1136/bmjopen-2020-043737 (PMC8166456; doi:10.1136/bmjopen-2020-043737)
Supplement: Author's manuscript [file bmjopen-2020-043737.draft_revisions.pdf]

BMJ Open is committed to open peer review. As part of this commitment we make the peer review history of every article we publish publicly available.

When an article is published we post the peer reviewers' comments and the authors' responses online. We also post the versions of the paper that were used during peer review. These are the versions that the peer review comments apply to.

The versions of the paper that follow are the versions that were submitted during the peer review process. They are not the versions of record or the final published versions. They should not be cited or distributed as the published version of this manuscript.

BMJ Open is an open access journal and the full, final, typeset and author-corrected version of record of the manuscript is available on our site with no access controls, subscription charges or pay-per-view fees (http://bmjopen.bmj.com).

If you have any questions on BMJ Open's open peer review process please email info.bmjopen@bmj.com

BMJ Open

Association between vitamin D supplementation or level and susceptibility to COVID-19 infection including clinical course, morbidity and mortality outcomes? A systematic review.

Journal:	*BMJ Open*
Manuscript ID	bmjopen-2020-043737
Article Type:	Original research
Date Submitted by the Author:	13-Aug-2020
Complete List of Authors:	Grove, Amy; Warwick Life Sciences, Division of Health Sciences, Warwick Medical School<br>Osokogu, Osemeke; University of Warwick, Warwick Medical School<br>Al-Khudairy, Lena; University of Warwick Warwick Medical School, Population, evidence and technology<br>Mehrabin, Amin; Warwick University , Warwick Medical School<br>Zanganeh, Mandana; University of Warwick, Warwick Medical School<br>Brown, Anna; University of Warwick, Warwick Medical School<br>Court, Rachel; University of Warwick, Health Sciences<br>Taylor-Phillips, Sian; University of Warwick, Warwick Medical School<br>Uthman, Olalekan; University of Warwick, Warwick-Centre for Applied Health Research (WCAHRD)<br>McCarthy, Noel; University of Warwick, Warwick Medical School<br>Kumar, Sudhesh; University of Warwick, Medical School<br>Clarke, Aileen; University of Warwick, Division of Health Sciences
Keywords:	COVID-19, Public health < INFECTIOUS DISEASES, NUTRITION & DIETETICS

SCHOLARONE™
Manuscripts

I, the Submitting Author has the right to grant and does grant on behalf of all authors of the Work (as defined in the below author licence), an exclusive licence and/or a non-exclusive licence for contributions from authors who are: i) UK Crown employees; ii) where BMJ has agreed a CC-BY licence shall apply, and/or iii) in accordance with the terms applicable for US Federal Government officers or employees acting as part of their official duties; on a worldwide, perpetual, irrevocable, royalty-free basis to BMJ Publishing Group Ltd ("BMJ") its licensees and where the relevant Journal is co-owned by BMJ to the co-owners of the Journal, to publish the Work in this journal and any other BMJ products and to exploit all rights, as set out in our licence.

The Submitting Author accepts and understands that any supply made under these terms is made by BMJ to the Submitting Author unless you are acting as an employee on behalf of your employer or a postgraduate student of an affiliated institution which is paying any applicable article publishing charge ("APC") for Open Access articles. Where the Submitting Author wishes to make the Work available on an Open Access basis (and intends to pay the relevant APC), the terms of reuse of such Open Access shall be governed by a Creative Commons licence – details of these licences and which Creative Commons licence will apply to this Work are set out in our licence referred to above.

Other than as permitted in any relevant BMJ Author's Self Archiving Policies, I confirm this Work has not been accepted for publication elsewhere, is not being considered for publication elsewhere and does not duplicate material already published. I confirm all authors consent to publication of this Work and authorise the granting of this licence.

For peer review only

Title page

Title

Association between vitamin D supplementation or level and susceptibility to COVID-19 infection including clinical course, morbidity and mortality outcomes? A systematic review.

Author's names

Amy Grove (0000-0002-8027-7274), Osemeke Osokogu, Lena Al-Khudairy, Amin Mehrabian, Mandana Zanganeh, Anna Brown, Rachel Court, Sian Taylor-Phillips, Olalekan Uthman, Noel McCarthy, Sudhesh Kumar, Aileen Clarke (0000-0001-8299-3146).[1]

Address for each author
Author's names and positions

1) Warwick Medical School, University of Warwick, Coventry, CV4 7AL, UK

Dr Amy Grove
Associate Professor of Health Technology Assessment and Implementation Science

Dr Osemeke Osokogu
Research Fellow

Dr Lena Al-Khudairy
Senior Research Fellow

Dr Mandana Zanganeh
Research Fellow

Mrs Anna Brown
Information Specialist

Mrs Rachel Court
Information Specialist

Professor Sian Taylor-Phillips
Professor of Population Health

Professor Olalekan Uthman
Professor of Evidence Synthesis

For peer review only - http://bmjopen.bmj.com/site/about/guidelines.xhtml

Professor Noel McCarthy
Professor of Public Health

Professor Sudhesh Kumar
Dean of Medicine

Professor Aileen Clarke
Professor of Public Health and Health Services Research

2) Department of Pharmaceutical Nanotechnology, School of Pharmacy, Mashhad University of Medical Sciences, Mashhad, Iran

Mr Amin Mehrabian
Research Fellow

Corresponding Author

Dr Amy Grove, Room A101, Division of Health Sciences, Warwick Medical School, University of Warwick, Coventry, CV4 7AL
Email: A.L.Grove@warwick.ac.uk

For peer review only

Abstract

Objective: To systemically review and critically appraise published studies of the association between vitamin D supplementation or level and susceptibility to COVID-19 infection, including clinical course, morbidity and mortality outcomes.

Design: Systematic review.

Data sources: MEDLINE (OVID), Embase (OVID), Cochrane Central Register of Controlled Trials, MedRxiv and BioRxiv preprint databases. COVID-19 databases of the WHO, Cochrane, CEBM Oxford, and Bern University up to 10 June 2020.

Study selection: Studies which assessed Vitamin D supplementation and/or Low Serum Vitamin D in patients acutely ill with, or at risk of severe betacoronavirus infection [SARS-CoV, MERS-CoV, SARS-CoV-2].

Data extraction: Two authors independently extracted data using a predefined critical appraisal and data extraction form; risk of bias was assessed using the Downs and Black Quality Assessment Checklist.

Results: Searches elicited 449 papers, 59 studies were included at full text. There was very limited evidence on the association between vitamin D supplementation or level and susceptibility to COVID-19 infection. Four articles were included in a narrative synthesis including a retrospective cohort study (348,598 participants, 449 cases) in which univariable analysis showed that vitamin D protects against COVID-19; a cross-sectional study (n=107) suggesting an inverse association between serum vitamin D and COVID-19, a case-control survey (n=1486) showing cases with confirmed/probable COVID-19 reported lower vitamin D supplementation, and an ecological country level study demonstrating a negative correlation between vitamin D and COVID-19 case numbers and mortality. All studies were at high/unclear risk of bias.

Conclusion: There is limited evidence of a negative association between vitamin D and COVID-19 infection. There is no peer reviewed published evidence of association between Vitamin D levels and severity of symptoms or mortality due to COVID-19. Guideline producers should acknowledge that benefits of vitamin D supplementation in COVID-19 infection are yet unproven despite increasing interest from the media and academic community.

Strengths and limitations of this study

For peer review only - http://bmjopen.bmj.com/site/about/guidelines.xhtml

- Speculation exists on the relationship between vitamin D and susceptibility to COVID-19 or disease/treatment outcomes but this has not been proven.
- This systematic review idenitified four relevant studies which were sythesised in a narrative synthesis; 1) a retrospective cohort study in which univariable analysis showed that vitamin D protects against COVID-19; 2) a cross-sectional evaluation which suggested an inverse association between serum vitamin D and COVID-19, 3) a survey where cases with confirmed/probable COVID-19 reported lower vitamin D supplementation, and 4) an ecological study which demonstrated a negative correlation between vitamin D and COVID-19.
- Results demonstrate that age and ethnicity were associated with COVID-19 infection even after multivariable adjustment. Black and South Asian people had a much higher risk of confirmed COVID-19 compared to white people. However, there was no interaction between ethnicity and vitamin D deficiency. Overall, positive COVID-19 cases had lower serum vitamin D and reported lower vitamin D supplementation in comparison to negative cases.
- Due to limitations of the evidence, it is not possible to conclude that vitamin D supplementation or level is associated with susceptibility to COVID-19, or its clinical course, morbidity or mortality.
- The limitations of the study relate to the small amount of evidence available which was, moreover, at risk of bias. This limits the inferences that can be drawn. The review was restricted to English language only, therefore non English language papers may have been missed.

For peer review only

For peer review only - http://bmjopen.bmj.com/site/about/guidelines.xhtml

Introduction

COVID-19, a novel viral infection caused by Severe Acute Respiratory Syndrome Coronavirus two (SARS-CoV-2) was declared a pandemic by The World Health Organization (WHO) on 11 March 2020.[1] Interest is mounting regarding the association of vitamin D supplementation or level with susceptibility to COVID-19 infection due to the recognised modulating effects of vitamin D on the immune system and immune response.

Vitamin D can modulate the immune system through highly expressed receptors in most non-skeletal tissues.[2 3] Two of the most common analogues of vitamin D in the human body are D_2 (ergocalciferol) and D_3 (cholecalciferol).[4] Both D_2 and D_3 can be hydroxylated by liver enzymes CYP2R1 and CYP27A1 to form calcidiol (25(OH)D). The active metabolite of vitamin D, calcitriol (1α,25(OH)2D), results from the action of CYP27B enzyme on calcidiol. CYP27B is found in several tissues including the kidney, skin, bones, and immune system.[5 6] Tumour necrosis factor α (TNF α) and interferon (IFNγ) are examples of inflammatory cytokines that stimulate the CYP27B enzymes of the immune system.[7-17] Vitamin D can interact with both the innate and cellular immune systems through these mechanisms.

Current Public Health England (PHE),[18] National Institutes of Health[19] and European Food Safety Authority[20] recommendations highlight the importance of vitamin D to population health. Vitamin D deficiency is defined as less than 25 nmol/L (10ng/ml) measured in blood serum.[18] Guideline recommendations suggest that people take a supplement of 10 micrograms of vitamin D per day during the winter months or throughout the year if they do not spend time outdoors or if they cover the majority of their skin when outside.[18] Published editorials, commentaries [21-26] and news media reports [27-29] suggest that individuals with low blood serum concentrations of vitamin D might be at higher risk of infection with COVID-19, or upon infection have worse outcomes than individuals with normal/high serum vitamin D.[30]

Several observational studies have reported associations between low serum vitamin D and chronic[31] and acute conditions such as susceptibility to acute respiratory tract infections (RTI).[32-34] Most recently, Martineau and colleagues (2017) conducted a systematic review and meta-analysis of individual participant data from randomised controlled trials to assess the overall effect of vitamin D supplementation on risk of acute RTI.[35] They reported vitamin D supplementation to be safe while protecting against acute RTI overall (adjusted odds ratio 0.88, 95% confidence interval 0.81 to 0.96; P for heterogeneity <0.001). Patients very deficient in vitamin D benefited the most (adjusted odds ratio 0.75, 0.60 to 0.95; p for interaction=0.006.[35] Recent rapid reviews of vitamin D for treatment or prevention in COVID-19 reported no evidence that vitamin D deficiency predisposes to COVID-19, or that vitamin D supplementation is effective in prevention or treatment of COVID-19.[36 37] However, data sources included in the rapid review were limited.[38]

For peer review only - http://bmjopen.bmj.com/site/about/guidelines.xhtml

Mild COVID-19 infection may manifest as high temperature, a continuous cough and a loss of or change in sense of smell or taste.[39 40] However, more severe and critical cases can result in inflammation of the lungs, low oxygen levels and acute respiratory distress syndrome.[41] If vitamin D supplementation could influence the susceptibility of people to COVID-19 infection, the intervention could be cost-effective with few associated safety risks.[35] Therefore, it is timely to systematically review and critically appraise all peer reviewed published evidence to assess the association of vitamin D supplementation or level with susceptibility to COVID-19 infection including clinical course, morbidity and mortality outcomes.

Methods

Protocol registration

The methods were prespecified in a protocol that was registered with the PROSPERO International Prospective Register of Systematic Reviews (https://www.crd.york.ac.uk/prospero/display_record.php?ID=CRD42020182876). Research ethics committee approval was not required for this study.

We undertook a systematic review to answer the following question: Is vitamin D supplementation or level associated with susceptibility to severe betacoronavirus infection (Severe Acute Respiratory Syndrome [SARS-CoV], Middle East Respiratory Syndrome [MERSCoV], Severe Acute Respiratory Syndrome two [SARS-CoV-2]) including clinical course, morbidity and mortality outcomes?

Our review was conceptualised and written in accordance with the PRISMA statement.[42]

Data sources and search

The search strategy was developed by the information specialists in collaboration with the research team and clinical advisors. We searched MEDLINE (OVID interface), Embase (OVID interface), Cochrane Central Register of Controlled Trials, MedRxiv and BioRxiv preprint databases on 6th-8th May 2020. We searched the global research on COVID-19 developed by the WHO,[43] CEBM Oxford,[44] and the living systematic review developed by Bern University[45] on 10 May 2020. We updated the database searches on 10th June 2020 to capture articles which may have been published since the initial search was conducted.

We also searched for relevant systematic reviews in MEDLINE (OVID interface), Embase (OVID interface) and Cochrane Database of Systematic Reviews (19th May 2020), and assessed the reference lists of two systematic reviews identified as potentially relevant. In addition, we assessed the reference lists of 17 narrative reviews.[24 25 30 46-59] Finally, we identified one additional article through consultation with clinical experts.[35] Our full search record is included in the supplementary information.

For peer review only - http://bmjopen.bmj.com/site/about/guidelines.xhtml

Study eligibility

We developed pre-defined study eligibility criteria aligned to the research question (Table 1). We imposed a date restriction of January 2002, to capture all published articles since SARS-CoV was first discovered in Asia in February 2003.[60] We limited to English language only.

Table 1. Study eligibility criteria

Study eligibility criteria
P - Population<br><br>1) Patients acutely ill with betacoronavirus infection [SARS-CoV, MERS-CoV, SARS-CoV-2]<br>2) or at risk of acute illness with betacoronavirus infection<br><br>I - Intervention/exposure<br><br>1) Vitamin D supplementation<br>2) Low Serum vitamin D<br><br>O - Outcomes<br><br>1) Betacoronavirus infection (to include serological evidence of infection or clinically confirmed symptomatic infection);<br>2) Severe betacoronavirus infection (to include patients admitted to hospital or admitted to intensive care); mortality due to betacoronavirus infection<br>3) Mortality due to betacoronavirus infection<br><br>C – Comparators<br><br>1) No vitamin D supplementation<br>2) High or normal serum vitamin D<br><br>S - Study design<br><br>Peer reviewed publications of randomised controlled trials and non-randomised studies were eligible for inclusion; including, non randomised controlled trials, interrupted time series analyses, controlled before-and-after studies, cohort studies, ecological studies, case reports and case series.<br><br>Subgroups<br><br>1. Ethnicity characteristics (White British, all other White, Mixed, Asian, Black, Other)<br>2. Age characteristics (population by five-year age groups)

Article selection

Following the article search, we systematically identified and removed any duplicate citations using EndNote X9 software. Using titles and abstracts, de-duplicated citations were screened by two independent reviewers and checked by a third. All articles deemed ineligible were excluded at this stage. We identified and obtained all remaining articles for full text screening, which was performed independently by at least two reviewers against the pre-specified eligibility criteria (Table 1). Where disagreements regarding the inclusion of articles arose, a third reviewer was consulted to reach a final decision.

For peer review only - http://bmjopen.bmj.com/site/about/guidelines.xhtml

Data extraction

Two reviewers independently extracted data from eligible full-text papers using a prespecified data extraction form. The accuracy of all the data extraction was independently assessed by a third reviewer. Where reported, we sought to extract data from each article relevant to the research question, including details of population, intervention/exposure, comparator, outcomes and any detail related to the two pre-specified subgroups: ethnicity characteristics and age characteristics. Disagreements between reviewers were resolved by discussion and agreement, or via consultation with a third reviewer.

Risk of bias

The included studies had observational study designs aimed at answering a specific question. Therefore, risk of bias of included full-text papers was assessed using the Downs and Black Quality Assessment Checklist.[61] Two reviewers independently assessed the risk of bias of the included studies and the accuracy of the assessment was evaluated by a third reviewer.

Data analysis

We anticipated that identified studies would be too heterogeneous to facilitate pooling of study data and planned a narrative synthesis. Nevertheless, we intended to consider pooling outcomes data in a meta-analysis using a random-effects model if appropriate.

Patient and public involvement

It was not possible to involve patients or the public in the design, conduct, or reporting of our research.

Results

After searching databases and other sources, we identified 499 citations. Following removal of duplicates and screening of titles and abstracts, we retrieved 59 full-text papers of which four met the full eligibility criteria (see Figure 1). The electronic supplement includes characteristics of included studies, and a list of reasons for excluding studies at full text review. Seven articles met the eligibility criteria but were excluded as they were not available as peer reviewed publications at the time of our narrative synthesis, details of these seven studies[62-68] is provided in the electronic supplement.

<Insert Figure 1 here>

Figure 1. PRISMA flow diagram for the selection of studies

Risk of bias assessments demonstrate that all studies scored poorly across several domains of the Downs and Black Quality Assessment Checklist,[61] including external validity, internal validity and power. A prominent issue amongst the included studies was that the authors did

For peer review only - http://bmjopen.bmj.com/site/about/guidelines.xhtml

not perfom adequate multivariable adjustment to correct for confounding.[71 72 73] Ecological bias was present in Ilie et al., 2020[70] which may result from spatial and temporal scale differences between country level mean levels of vitamin D. However, several domains in each risk of bias assessment were not applicable or not reported and therefore, could not be scored using the Downs and Black Quality Assessment Checklist.[61] The risk of bias scores are detailed in the electronic supplement. All four included studies were conducted in Europe and published in April or May 2020. One study was based on data from UK residents exclusively,[69] another included data on residents in 20 European countries, including the UK.[70]

Hastie et al., 2020 is a retrospective cohort study that utilised data from the UK Biobank.[71] UK Biobank is a long-term follow-up study of about 500,000 participants originally recruited between 2006 and 2010 when they were between 37 and 73 years old.[69] For this study 348,598 people with complete data on vitamin D and covariates were included; of which 449 tested positive for COVID-19 infection. Of these 385 (85.8%) were White compared to 64 (14.2%) non-white (Black, South Asian and others). Conversely, the 345,140 COVID-19 negative participants included 331,464 (95.2%) White compared to 16,685 (4.8%) non-white. COVID-19 positive participants were older (Median = 49 years; Interquartile Range [IQR] = 40-58) than COVID-19 negative participants (Median = 49 years; IQR = 38 - 57) with p-value of <0.05. Multivariable analysis showed that age at assessment (OR = 1.02; 95% CI = 1.00 - 1.03; P= 0.016) and non-white ethnicity (Black OR = 4.30, 95% CI = 2.92 - 6.31, P= < 0.001; South Asian OR = 2.42, 95% CI = 1.50 - 3.93, P= <0.001) were associated with confirmed COVID-19 infection. There was no significant interaction between ethnicity and vitamin D deficiency (OR = 0.90; 95% CI = 0.66 - 1.23; P= 0.515).

Median vitamin D concentration at recruitment was lower in patients with subsequent confirmed COVID-19 infection (28.7 [IQR 10.0-43.8] nmol/l) than other participants (32.7 [IQR 10.0-47.2) nmol/l) (P= <0.01). Although univariable analysis suggested an association between vitamin D and COVID-19 (OR=0.99; 95% CI 0.99 – 0.999; P=0.013), this association became insignificant (OR=1.00; 95% CI = 0.998-1.01; P=0.208) after adjustment for covariates.[69]

D'Avolio et al, 2020[72] is a cross-sectional study in which 25(OH)D levels were compared among three groups (the third group is not relevant for this review and therefore not presented) of participants from the Canton of Tessin, Switzerland. Data from participants that had a nasopharyngeal swab PCR analysis for COVID-19 and a 25(OH)D measurement were retrospectively evaluated. Group 1 comprised 27 patients with positive polymerase chain reaction (PCR) test results for COVID-19 while group 2 comprised 80 patients with a negative PCR result for COVID-19. Both groups had their 25(OH) D and COVID-19 status assessed between 1st March and 14th April 2020. Group 1 participants (median age = 74 years [IQR 65–81];male = 70.4%;) had significantly (P= 0.004) lower serum 25(OH)D levels (median = 11.1 ng/mL [IQR 8.2–21.0]) than group 2 participants (median age = 73 years [IQR 61–82]; male =

For peer review only - http://bmjopen.bmj.com/site/about/guidelines.xhtml

48.8%; median 25(OH)D = 24.6 ng/mL [IQR 8.9–30.5]). Although gender and age stratified analysis showed no statistically overall significant differences, older (>70 years) group 1 (COVID-19 test positive, n =18) participants had significantly (p = 0.037) lower median serum 25 (0H) D levels (9.3 ng/mL [IQR 8.1–19.9] than older group 2 (COVID-19 negative, n = 43) (23.1 ng/mL [IQR 8.5–31.7]).

Fasano et al., 2020[73] is a case-control phone survey that investigated patients from one of the largest tertiary centres for Parkinson Disease in Lombardy, Italy. COVID-19 diagnosis was either “confirmed” using a nasopharyngeal swab or “probable” based on the following criteria: a) presence of persistent COVID-19-related symptoms (≥3 including fever or ≥5 without fever); or b) ≥1 symptom in presence of suggestive chest radiologic signs; and/or c) living with a family member with a confirmed diagnosis of COVID-19. A total of 1486 participants were included in the survey (77.2% response rate, 32 confirmed COVID-19, 73 probable COVID-19 and 1381 unaffected). Confirmed/probable COVID-19 cases (mean age = 70.5 [Standard Deviation [SD] = 10.1]; male = 53%) reported a significantly lower intake of vitamin D supplementation (12.4%) compared to unaffected cases (22.9%; mean age = 73.0 [SD = 9.5], male = 57%). The age-adjusted OR (OR 0.56 [95% CI = 0.32-0.99], P= 0.048) suggested a protective effect of vitamin D intake.

Ilie et al., 2020[70] is an ecological study which used data reported from 20 European countries as at 8th April 2020; the data pertains to mean levels of vitamin D, cases of COVID-19 infection per million population and deaths from COVID-19 per million population. The authors performed Pearson Correlation Coefficient Calculations and reported a negative correlation between mean levels of vitamin D (Mean 56.79 nmol/l, SD 10.61) and number of cases of COVID-19 infection per million population in each country (Mean 1393.4, SD 1129.984, $r(20) = -0.44$; P = 0.05). Ilie et al., 2020 also reported a negative correlation between mean vitamin D levels and the number of deaths caused by COVID-19 per million population in each country (Mean 80.42, SD 94.61, $r(20) = -0.4378$; P = 0.05). Sweden had the highest mean level of vitamin D (73.5 nmol/l) compared to Spain which had a mean level of 42.5 nmol/l). The number of cases of COVID-19 per million population was 834 in Sweden and 3,137 in Spain. Likewise, at the time of the study, there were 68 deaths from COVID-19 per million population in Sweden and 314 in Spain.

We planned to perform subgroup analyses by age and ethnicity. According to Hastie et al., 2020[69] multivariable analysis showed that age at assessment (OR = 1.02; 95% CI = 1.00 - 1.03; P= 0.016) and non-white ethnicity (Black OR = 4.30, 95% CI = 2.92 - 6.31, P= < 0.001; South Asians OR = 2.42, 95% CI = 1.50 - 3.93, P= <0.001) were associated with confirmed COVID-19 infection. However, Hastie et al found no significant interaction between ethnicity and vitamin D deficiency (OR = 0.90; 95% CI = 0.66 - 1.23; P= 0.515).

Discussion

For peer review only - http://bmjopen.bmj.com/site/about/guidelines.xhtml

This systematic review of non-randomised studies has shown limited evidence of an association between vitamin D and COVID-19 infection. We identified four studies for inclusion in a narrative synthesis. A univariable analysis of data from the UK Biobank database revealed an association between vitamin D and COVID-19 infection (OR=0.99; 95% CI 0.99 – 0.999; P=0.013). However, this association became insignificant (OR=1.00; 95% CI=0.998-1.01; P=0.208) after adjustment for 13 other covariates. The second small evaluation suggested a significant inverse association between a positive PCR test and serum vitamin D levels. Older (>70 years) positive cases had lower serum levels in comparison to negative cases but this did not reach statistical significance. However, the findings are limited as the authors did not perform any multivariable adjustment to correct for confounding. The third study was a survey that suggested that patients with Parkinson Disease and a confirmed/probable COVID-19 diagnosis reported lower vitamin D supplementation in comparison to negative COVID-19 cases. In this study, the authors adjusted for only age which is a limitation. A fourth study using a design subject to ecological bias, reported a negative correlation between mean levels of vitamin D (mean 56.8 nmol/l, SD 10.6), and number of COVID-19 cases and number of COVID-19 deaths per 1 million persons across 20 European countries.

The fact that the initially identified association between vitamin D and COVID-19 infection found by Hastie et al., 2020 became insignificant after adjustment for covariates, suggests that the initial association was due to one or more confounding variables.[69] This view is further strengthened by the demonstration of highly significant associations between age and ethnic characteristics as predictor variables, and COVID-19 infection as the outcome variable. However, it should be noted that the UK Biobank data included only one measurement of Vitamin D levels for participants which was taken between 10 and 14 years prior to the outbreak of COVID-19.

D'Avolio results suggested that older (>70 years) positive cases had lower serum levels in comparison to negative cases, however this did not reach statistical significance. Liu et al., 2020 concluded that patients over 60 years experienced more severe manifestations and had longer disease courses of COVID-19 compared to patients below 60 years.[74] Other studies have shown that older (rather than younger) people are more likely to die from COVID-19 infection.[75-78] Although older persons have an increased likelihood of being vitamin D deficient and vitamin D is thought to protect against COVID-19 infection possibly via an immune-mediated pathway,[79] our review shows that age remained an independent predictor of COVID-19 infection even after adjusting for vitamin D levels. Further research is essential to better understand all the associated issues. Non-white people are known to be more susceptible to COVID-19 infection and tend to develop worse outcomes,[80] a finding that our review has further substantiated.[69]

For peer review only - http://bmjopen.bmj.com/site/about/guidelines.xhtml

Nevertheless, Hastie et al., 2020 did not find any interaction between ethnicity and vitamin D deficiency and although Ilie et al 2020[70] identified a relationship, the study is subject to ecological bias. Ethnicity is a multi-faceted construct that includes genetic make-up, socio-cultural identity and behavioural patterns.[81] Many studies have demonstrated significant differences in disease manifestations, based on complex ethnicity-related factors. An example is the impact of ethnic disparities on treatment outcomes in patients with tuberculosis.[82 83] Bime et al., 2016 reported that '*blacks, hispanics, and other racial minorities in the US were observed to exhibit significantly higher in-hospital sepsis-related respiratory failure associated mortality when compared with non-Hispanic whites'*.[84] Given the findings so far from our review and research to date we consider that a relationship between ethnicity, vitamin D (serum levels or supplementation) and susceptibility to or severity of COVID-19 infection cannot yet be ruled out. In particular, there is paucity of data on vitamin D levels and morbidity and mortality from COVID-19 and there is no evidence from randomised clinical trials on outcomes of vitamin D supplementation on severity of symptoms or mortality so far.

Risk of bias assessments demonstrate that all studies scored poorly across several domains of the Downs and Black Quality Assessment Checklist.[61] All studies were observational designs and therefore subject to confounding. Of the four included studies, Hastie et al., 2020[69] performed multivariable adjustment for many potential confounders whereas Fasano et al., 2020[73] only adjusted for only age and D'Avolio et al, 2020[72] did not report using multivariable adjustment to correct for confounders. Until more robust scientific evidence for vitamin D supplementation is available, there will be limited evidence to support greater supplementation (upper limit of 4000 IU/day (100 µg/day)) of vitamin D to reduce susceptibility to COVID-19 infection. More robust prognostic studies could be combined in a systematic review where a prognostic factor research question is phrased and considerations of participation, attrition, prognostic factor measurement, confounding measurement and account, outcome measurement, and analysis and reporting are evaluated. Thereby, fully assessing validity and bias of the included studies.

The persistent calls for high-dose vitamin D supplementation[85] arise from speculation about presumed mechanisms. We have identified one study involving an '*unbiased screen of repurposed drugs for treatment of avian influenza A H5N1 virus using appropriate cell lines and mice, which highlighted calcitriol (the active hormone of vitamin D) as a potential therapy.*'[86] A second study involved '*a recent analysis of vitamin D and viral infections.*'[87] Publication of peer reviewed research specific to COVID-19 infection is required to identify the exact mechanisms involved in the human population. We are aware of two ongoing randomised clinical trials investigating the effects of vitamin D on COVID-19, the ZnD3-CoVici study, France (NCT04351490)[88] and the CoVitTrial, France (NCT04344041).[89] Both trials have an estimated study completion date of July 2020. Inclusion of data from these studies in future systematic review and meta-analyses may enable us to potentially draw better conclusions even for subgroup effects. Results from the ongoing international VITDALIZE

For peer review only - http://bmjopen.bmj.com/site/about/guidelines.xhtml

Study (NCT03188796) may also contribute to our understanding of the effect of high dose vitamin D3 on mortality.[90]

Study limitations

We performed a full systematic review of the published evidence available, and simultaneous independent screening, data extraction and risk of bias assessments. However, our study is limited by the small amount of evidence available which was, moreover, at risk of bias. This limits the inferences that can be drawn. Seven eligible studies were excluded because they are not available as peer reviewed publications.[62-68] If published, these seven studies would be included. A final limitation is that the review was restricted to English language only. Therefore, articles published in other languages may have been excluded.

Implications for practice

Our review does not provide evidence for or against additional or high dose vitamin D supplementation specifically in relation to COVID-19. Treatment as standard practice for people who are deficient is pre-existing practice. The European Food Safety Authority revised dietary reference values for vitamin D for the EU population in 2016.[20] They recommend that all population groups aged one year and more achieve an adequate intake of 15 µg/day with an assumed minimal sunshine exposure and considered there to be increase health risks at serum 25(OH)D concentrations below 50 nmol/L.[91] An Institute of Medicine review concluded that persons are at risk of vitamin D deficiency at serum 25(OH)D concentrations <30 nmol/L (<12 ng/mL) and 50 nmol/L is the serum 25(OH)D level that covers the needs of 97.5% of the US population. The current guidelines from PHE suggest that the entire UK population should take vitamin D supplements to prevent vitamin D deficiency in winter or with inadequate sunlight exposure to sun in summer.[18] This review does not give evidence to drive a change in this current advice. Treatment recommendations for patients should be updated following the publication of results from the ongoing randomised clinical trials.

Conclusion

This systematic review identified very limited evidence to enable us to assess an association between vitamin D supplementation or level with susceptibility to COVID-19 infection including clinical course, morbidity and mortality outcomes. Narrative synthesis of the four included studies found a retrospective cohort study in which univariable (but not multivariable) analysis showed that vitamin D protects against COVID-19; a cross-sectional study (107 participants) that suggested an inverse association between serum vitamin D and COVID-19; a case-control survey (n = 1486) that showed cases with confirmed/probable COVID-19 reported lower vitamin D supplementation and finally an ecological country level study that demonstrated a negative correlation between vitamin D and COVID-19 case numbers and mortality. All studies were at high or unclear risk of bias. The results provide very limited evidence of an association between vitamin D and COVID-19 infection. Both age and ethnicity were associated with vitamin D even after multivariable adjustment. Black and

For peer review only - http://bmjopen.bmj.com/site/about/guidelines.xhtml

South Asian people had a much higher risk of confirmed COVID-19 compared to white people. However, there was no interaction between the association of ethnicity and vitamin D deficiency with COVID-19. There were no papers reporting association of vitamin D with severity of symptoms or mortality due to COVID-19.

What is already known on this topic

- Much speculation exists on the relationship between vitamin D and susceptibility to COVID-19 or disease/treatment outcomes but this has not been proven.
- Since low serum vitamin D predisposes to acute respiratory tract infections (RTI), and COVID-19 can manifest as an acute RTI, it is plausible that vitamin D supplementation could reduce the risk of contracting COVID-19 or reduce its severity. Such an intervention would be low cost and with few safety risks.
- A recent rapid review could not demonstrate any evidence of vitamin D deficiency predisposing to COVID-19 or of the successful use of vitamin D supplementation for preventing or treating COVID-19. However, this review was limited in its searches.

What this study adds

- Four relevant studies were identified: a retrospective cohort study in which univariable (but not multivariable) analysis showed that vitamin D protects against COVID-19; a cross-sectional evaluation which suggested an inverse association between serum vitamin D and COVID-19, a survey where cases with confirmed/probable COVID-19 reported lower vitamin D supplementation, and an ecological study which demonstrated a negative correlation between vitamin D and COVID-19.
- Both age and ethnicity were associated with COVID-19 infection even after multivariable adjustment. Black and South Asian people had a much higher risk of confirmed COVID-19 compared to white people. However, there was no interaction between ethnicity and vitamin D deficiency.
- Positive COVID-19 cases had lower serum vitamin D and reported lower vitamin D supplementation in comparison to negative cases.
- Due to limited evidence, we cannot conclude that vitamin D supplementation or level is associated with susceptibility to COVID-19, or its clinical course, morbidity or mortality. Meanwhile clinicians and policy makers should ensure that patients with vitamin D deficiency are appropriately treated, regardless of COVID-19 status, and follow pre-existing population level public health advice.

For peer review only

Acknowledgements:

We thank Mrs Kath Charters, Welsh Ambulance Services NHS Trust for comments on the study design and drafts of this manuscript and for valuable advice and helpful discussions.

Contributorship statement:

SK, AG and AC conceived the study. AG, AC, NMcC, SK, STP and OU designed the study. AG, AC, AM screened titles and abstracts for inclusion. AG, OO, AM, MZ, LAK, AC screened at full text and extracted and analysed data. OO, AM, MZ performed risk of bias assessments. AC, SK and NMcC assisted in the interpretation from a clinical perspective. STP, LAK, OU offered technical and methodological support. AG and OO wrote the first draft, all authors revised content. All authors approved the final manuscript. AG and AC are the guarantors. The corresponding author attests that all listed authors meet authorship criteria and that no others meeting the criteria have been omitted.

Copyright/license for publication:

The Corresponding Author has the right to grant on behalf of all authors and does grant on behalf of all authors, a worldwide licence to the Publishers and its licensees in perpetuity, in all forms, formats and media (whether known now or created in the future), to i) publish, reproduce, distribute, display and store the Contribution, ii) translate the Contribution into other languages, create adaptations, reprints, include within collections and create summaries, extracts and/or, abstracts of the Contribution, iii) create any other derivative work(s) based on the Contribution, iv) to exploit all subsidiary rights in the Contribution, v) the inclusion of electronic links from the Contribution to third party material where-ever it may be located; and, vi) licence any third party to do any or all of the above.

Funding:

Dr Amy Grove is supported by the National Institute for Health Research (NIHR) Advanced Fellowship Programme Reference (AF-300060). Dr Taylor-Phillips is supported by an NIHR Career Development Fellowship (CDF-2016-09-018). Prof Clarke and Dr Al-Khudairy are supported by the NIHR Applied Research Centre (ARC) West Midlands. All other authors, with the exception of Professor Uthman, Professor Kumar and Professor McCarthy, are supported by the NIHR, Evidence Synthesis Programme. The views expressed are those of the authors and not necessarily those of the NIHR or the Department of Health and Social Care.

Competing interests:

All authors have completed the ICMJE uniform disclosure form at www.icmje.org/coi_disclosure.pdf and declare: no support from any organisation for the submitted work; no competing interests with regards to the submitted work.

Ethical statement:

For peer review only - http://bmjopen.bmj.com/site/about/guidelines.xhtml

Not required

Data sharing statement:

The study protocol is available

Systematic review protocol registration: CRD42020182876 available online via PROSPERO at https://www.crd.york.ac.uk/prospero/display_record.php?ID=CRD42020182876. All included studies are publicly available. Additional data are available upon reasonable request by emailing the corresponding author.

For peer review only

For peer review only - http://bmjopen.bmj.com/site/about/guidelines.xhtml

References

1. World Health Organization. Coronavirus disease (COVID-19) pandemic. 2020. https://www.who.int/emergencies/diseases/novel-coronavirus-2019
2. Gruber BM. Fenomen witaminy D [The phenomenon of vitamin D]. *Postepy Hig Med Dosw (Online)* 2015;69:127-39
3. Gruber-Bzura BM. Vitamin D and influenza: prevention or therapy? *Int J Mol Sci* 2018;19(8):2419. doi: 10.3390/ijms19082419
4. Holick MF. High prevalence of vitamin D inadequacy and implications for health. *Mayo Clin Proc* 2006;81(3):353-73. doi: 10.4065/81.3.353
5. Sigmundsdottir H, Pan J, Debes GF, et al. DCs metabolize sunlight-induced vitamin D3 to 'program' T cell attraction to the epidermal chemokine CCL27. *Nat Immunol* 2007;8(3):285-93. doi: 10.1038/ni1433
6. Kogawa M, Findlay DM, Anderson PH, et al. Osteoclastic metabolism of 25(OH)-vitamin D3: a potential mechanism for optimization of bone resorption. *Endocrinology* 2010;151(10):4613-25. doi: 10.1210/en.2010-0334
7. Jones G. Vitamin D safety: its mechanisms and application. *Standardy Medyczne, Pediatria* 2012;9:605-09
8. Kim D. The role of vitamin D in thyroid diseases. *Int J Mol Sci* 2017;18(9):1949. doi: 10.3390/ijms18091949
9. Beard JA, Bearden A, Striker R. Vitamin D and the anti-viral state. *J Clin Virol* 2011;50(3):194-200. doi: 10.1016/j.jcv.2010.12.006
10. Liu PT, Stenger S, Li H, et al. Toll-like receptor triggering of a vitamin D-mediated human antimicrobial response. *Science* 2006;311(5768):1770-3. doi: 10.1126/science.1123933
11. Adams JS, Ren S, Liu PT, et al. Vitamin D-directed rheostatic regulation of monocyte antibacterial responses. *J Immunol* 2009;182(7):4289-95. doi: 10.4049/jimmunol.0803736
12. Laaksi I. Vitamin D and respiratory infection in adults. *Proc Nutr Soc* 2012;71(1):90-7. doi: 10.1017/s0029665111003351
13. Sharifi A, Vahedi H, Nedjat S, Rafiei H, Hosseinzadeh-Attar MJ. Effect of single-dose injection of vitamin D on immune cytokines in ulcerative colitis patients: a randomized placebo-controlled trial. *APMIS* 2019;127(10):681-87. doi: 10.1111/apm.12982
14. Cantorna MT, Snyder L, Lin YD, Yang L. Vitamin D and 1,25(OH)2D regulation of T cells. *Nutrients* 2015;7(4):3011-21. doi: 10.3390/nu7043011
15. Jeffery LE, Burke F, Mura M, et al. 1,25-Dihydroxyvitamin D3 and IL-2 combine to inhibit T cell production of inflammatory cytokines and promote development of regulatory T cells expressing CTLA-4 and FoxP3. *J Immunol* 2009;183(9):5458-67. doi: 10.4049/jimmunol.0803217
16. Lei GS, Zhang C, Cheng BH, Lee CH. Mechanisms of action of vitamin D as supplemental therapy for pneumocystis pneumonia. *Antimicrob Agents Chemother* 2017;61(10):e01226-17. doi: 10.1128/aac.01226-17
17. Colunga Biancatelli RML, Berrill M, Marik PE. The antiviral properties of vitamin C. *Expert Rev Anti Infect Ther* 2020;18(2):99-101. doi: 10.1080/14787210.2020.1706483

For peer review only

For peer review only - http://bmjopen.bmj.com/site/about/guidelines.xhtml

18. Scientific Advisory Committee on Nutrition. SACN vitamin D and health report. 21 July 2016. https://www.gov.uk/government/publications/sacn-vitamin-d-and-health-report
19. National Institutes of Health, Office of Dietary Supplements. Vitamin D: fact sheet for health professionals. 24 March 2020. https://ods.od.nih.gov/factsheets/VitaminD-HealthProfessional/#h2
20. EFSA Panel on Dietetic Products, Nutrition and Allergies. Dietary reference values for vitamin D. *EFSA Journal* 2016;14(10):e04547. doi: 10.2903/j.efsa.2016.4547
21. Cao Z, Wu Y, Faucon E, Sabatier JM. SARS-CoV-2 & Covid-19: key-roles of the 'renin-angiotensin' system / vitamin D impacting drug and vaccine developments. *Infect Disord Drug Targets* 2020;20(1). doi: 10.2174/1871526520999200505174704
22. Jakovac H. COVID-19 and vitamin D: is there a link and an opportunity for intervention? *Am J Physiol Endocrinol Metab* 2020;318(5):E589. doi: 10.1152/ajpendo.00138.2020
23. Mitchell F. Vitamin-D and COVID-19: do deficient risk a poorer outcome? *Lancet Diabetes Endocrinol* 2020;8(7):570. doi: 10.1016/S2213-8587(20)30183-2
24. Silberstein M. Vitamin D: a simpler alternative to tocilizumab for trial in COVID-19? *Med Hypotheses* 2020;140:109767. doi: 10.1016/j.mehy.2020.109767
25. Calder PC, Carr AC, Gombart AF, Eggersdorfer M. Optimal nutritional status for a well-functioning immune system is an important factor to protect against viral infections. *Nutrients* 2020;12(4):1181. doi: https://dx.doi.org/10.3390/nu12041181
26. Tian Y, Rong L. Letter: Covid-19, and vitamin D. Authors' reply. *Aliment Pharmacol Ther* 2020;51(10):995-96. doi: 10.1111/apt.15764
27. Busby M. UK public health bodies reviewing vitamin D's effects on coronavirus. The Guardian; 17 June 2020. https://www.theguardian.com/world/2020/jun/17/uk-ministers-order-urgent-vitamin-d-coronavirus-review
28. Coronavirus: Should I start taking vitamin D? BBC News; 18 June 2020. https://www.bbc.co.uk/news/health-52371688
29. Torjesen I. Covid-19: Public health agencies review whether vitamin D supplements could reduce risk. *BMJ* 2020;369:m2475. doi: 10.1136/bmj.m2475
30. Grant WB, Lahore H, McDonnell SL, et al. Evidence that vitamin D supplementation could reduce risk of influenza and COVID-19 infections and deaths. *Nutrients* 2020;12(4):988. doi: 10.3390/nu12040988
31. Parker J, Hashmi O, Dutton D, et al. Levels of vitamin D and cardiometabolic disorders: systematic review and meta-analysis. *Maturitas* 2010;65(3):225-36. doi: 10.1016/j.maturitas.2009.12.013
32. Cannell JJ, Vieth R, Umhau JC, et al. Epidemic influenza and vitamin D. *Epidemiol Infect* 2006;134(6):1129-40. doi: 10.1017/s0950268806007175
33. Jolliffe DA, Griffiths CJ, Martineau AR. Vitamin D in the prevention of acute respiratory infection: systematic review of clinical studies. *J Steroid Biochem Mol Biol* 2013;136:321-9. doi: 10.1016/j.jsbmb.2012.11.017
34. Nnoaham KE, Clarke A. Low serum vitamin D levels and tuberculosis: a systematic review and meta-analysis. *Int J Epidemiol* 2008;37(1):113-19. doi: 10.1093/ije/dym247
35. Martineau AR, Jolliffe DA, Hooper RL, et al. Vitamin D supplementation to prevent acute respiratory tract infections: systematic review and meta-analysis of individual participant data. *BMJ* 2017;356:i6583. doi: 10.1136/bmj.i6583

For peer review only - http://bmjopen.bmj.com/site/about/guidelines.xhtml

36. National Institute for Health and Care Excellence. COVID-19 rapid evidence summary: vitamin D for COVID-19. 29 June 2020. https://www.nice.org.uk/advice/es28/chapter/Key-messages
37. Royal Society. Vitamin D and covid-19. 18 June 2020. https://royalsociety.org/-/media/policy/projects/set-c/set-c-vitamin-d-and-covid-19.pdf
38. Lee J, van Hecke O, Roberts N, on behalf of the Oxford COVID-19 Evidence Service Team. Vitamin D: a rapid review of the evidence for treatment or prevention in COVID-19. Centre for Evidence-Based Medicine, University of Oxford; 1 May 2020. https://www.cebm.net/covid-19/vitamin-d-a-rapid-review-of-the-evidence-for-treatment-or-prevention-in-covid-19/
39. NHS. Check if you or your child has coronavirus symptoms. 2020. https://www.nhs.uk/conditions/coronavirus-covid-19/symptoms/#symptoms
40. Argenziano MG, Bruce SL, Slater CL, et al. Characterization and clinical course of 1000 patients with coronavirus disease 2019 in New York: retrospective case series. *BMJ* 2020;369:m1996. doi: 10.1136/bmj.m1996
41. Matthay MA, Aldrich JM, Gotts JE. Treatment for severe acute respiratory distress syndrome from COVID-19. *Lancet Respir Med* 2020;8(5):433-34. doi: 10.1016/s2213-2600(20)30127-2
42. Moher D, Liberati A, Tetzlaff J, Altman DG. Preferred reporting items for systematic reviews and meta-analyses: the PRISMA statement. *BMJ* 2009;339:b2535. doi: 10.1136/bmj.b2535
43. World Health Organization. Global research on coronavirus disease (COVID-19) [online database]. 2020. https://www.who.int/emergencies/diseases/novel-coronavirus-2019/global-research-on-novel-coronavirus-2019-ncov
44. Oxford COVID-19 Evidence Service. Centre for Evidence-Based Medicine, University of Oxford; 2020. https://www.cebm.net/oxford-covid-19-evidence-service/
45. Counotte M, Imeri H, Ipekci M, Low N. Living evidence on COVID-19 [online database]. University of Bern Institute of Social and Preventive Medicine; 2020. https://zika.ispm.unibe.ch/assets/data/pub/ncov/
46. Carter SJ, Baranauskas MN, Fly AD. Considerations for obesity, vitamin D, and physical activity amidst the COVID-19 pandemic. *Obesity (Silver Spring)* 2020;28:1176-77. doi: 10.1002/oby.22838
47. Molloy EJ, Murphy N. Vitamin D, covid-19 and children. *Ir Med J* 2020;113(4):64
48. McCartney DM, Byrne DG. Optimisation of vitamin D status for enhanced immuno-protection against covid-19. *Ir Med J* 2020;113(4):P58
49. Adams KK, Baker WL, Sobieraj DM. Myth busters: dietary supplements and COVID-19. *Ann Pharmacother* 2020;54(8):820-26. doi: 10.1177/1060028020928052
50. de Lucena TMC, da Silva Santos AF, de Lima BR, de Albuquerque Borborema ME, de Azevedo Silva J. Mechanism of inflammatory response in associated comorbidities in COVID-19. *Diabetes Metab Syndr* 2020;14(4):597-600. doi: 10.1016/j.dsx.2020.05.025
51. Ghasemian R, Shamshirian A, Heydari K, et al. The role of vitamin D in the age of COVID-19: a systematic review and meta-analysis along with an ecological approach. *medRxiv* 2020. doi: 10.1101/2020.06.05.20123554
52. Hribar CA, Cobbold PH, Church FC. Potential role of vitamin D in the elderly to resist COVID-19 and to slow progression of Parkinson's disease. *Brain Sci* 2020;10(5):284. doi: 10.3390/brainsci10050284

For peer review only - http://bmjopen.bmj.com/site/about/guidelines.xhtml

53. Laird E, Rhodes J, Kenny RA. Vitamin D and inflammation: potential implications for severity of Covid-19. *Ir Med J* 2020;113(5):P81
54. McKenna MJ, Flynn MAT. Covid-19, cocooning and vitamin D intake requirements. *Ir Med J* 2020;113(5):P79
55. Ribeiro H, Santana KVS, Oliver SL, et al. Does vitamin D play a role in the management of Covid-19 in Brazil? *Rev Saude Publica* 2020;54:53
56. Speeckaert MM, Delanghe JR. Association between low vitamin D and COVID-19: don't forget the vitamin D binding protein. *Aging Clin Exp Res* 2020. doi: 10.1007/s40520-020-01607-y
57. Suresh PS. Hypovitaminosis D and COVID-19: matter of concern in India? *Indian J Clin Biochem* 2020. doi: 10.1007/s12291-020-00894-6
58. Tan SHS, Hong CC, Saha S, Murphy D, Hui JH. Medications in COVID-19 patients: summarizing the current literature from an orthopaedic perspective. *Int Orthop* 2020. doi: 10.1007/s00264-020-04643-5
59. Zabetakis I, Lordan R, Norton C, Tsoupras A. COVID-19: the inflammation link and the role of nutrition in potential mitigation. *Nutrients* 2020;12(5):1466. doi: 10.3390/nu12051466
60. Centers for Disease Control and Prevention. CDC SARS response timeline. 2013. https://www.cdc.gov/about/history/sars/timeline.htm
61. Downs SH, Black N. The feasibility of creating a checklist for the assessment of the methodological quality both of randomised and non-randomised studies of health care interventions. *J Epidemiol Community Health* 1998;52(6):377-84. doi: 10.1136/jech.52.6.377
62. Lau FH, Majumder R, Torabi R, et al. Vitamin D insufficiency is prevalent in severe COVID-19. *medRxiv* 2020. doi: 10.1101/2020.04.24.20075838
63. Darling AL, Ahmadi KR, Ward KA, et al. Vitamin D status, body mass index, ethnicity and COVID-19: initial analysis of the first-reported UK Biobank COVID-19 positive cases (n 580) compared with negative controls (n 723). *medRxiv* 2020. doi: 10.1101/2020.04.29.20084277
64. De Smet D, De Smet K, Herroelen P, Gryspeerdt S, Martens GA. Vitamin D deficiency as risk factor for severe COVID-19: a convergence of two pandemics. *medRxiv* 2020. doi: 10.1101/2020.05.01.20079376
65. Meltzer DO, Best TJ, Zhang H, Vokes T, Arora V, Solway J. Association of vitamin D deficiency and treatment with COVID-19 incidence. *medRxiv* 2020. doi: 10.1101/2020.05.08.20095893
66. Notari A, Torrieri G. COVID-19 transmission risk factors. *medRxiv* 2020. doi: 10.1101/2020.05.08.20095083
67. Raisi-Estabragh Z, McCracken C, Bethell MS, et al. Greater risk of severe COVID-19 in non-White ethnicities is not explained by cardiometabolic, socioeconomic, or behavioural factors, or by 25(OH)-vitamin D status: study of 1,326 cases from the UK Biobank. *medRxiv* 2020. doi: 10.1101/2020.06.01.20118943
68. Tan CW, Ho LP, Kalimuddin S, et al. A cohort study to evaluate the effect of combination vitamin D, magnesium and vitamin B12 (DMB) on progression to severe outcome in older COVID-19 patients. *medRxiv* 2020. doi: 10.1101/2020.06.01.20112334
69. Hastie CE, Mackay DF, Ho F, et al. Vitamin D concentrations and COVID-19 infection in UK Biobank. *Diabetes Metab Syndr* 2020;14(4):561-65. doi: 10.1016/j.dsx.2020.04.050

For peer review only

For peer review only - http://bmjopen.bmj.com/site/about/guidelines.xhtml

70. Ilie PC, Stefanescu S, Smith L. The role of vitamin D in the prevention of coronavirus disease 2019 infection and mortality. *Aging Clin Exp Res* 2020. doi: 10.1007/s40520-020-01570-8
71. UK Biobank. About UK Biobank. 17 June 2020. https://www.ukbiobank.ac.uk/about-biobank-uk/
72. D'Avolio A, Avataneo V, Manca A, et al. 25-hydroxyvitamin D concentrations are lower in patients with positive PCR for SARS-CoV-2. *Nutrients* 2020;12(5):1359. doi: 10.3390/nu12051359
73. Fasano A, Cereda E, Barichella M, et al. COVID-19 in Parkinson's Disease Patients Living in Lombardy, Italy. *Mov Disord* 2020. doi: 10.1002/mds.28176
74. Liu Y, Mao B, Liang S, et al. Association between age and clinical characteristics and outcomes of COVID-19. *Eur Respir J* 2020;55(5):2001112. doi: 10.1183/13993003.01112-2020
75. Verity R, Okell LC, Dorigatti I, et al. Estimates of the severity of coronavirus disease 2019: a model-based analysis. *Lancet Infect Dis* 2020;20(6):669-77. doi: 10.1016/s1473-3099(20)30243-7
76. Mahase E. Covid-19: death rate is 0.66% and increases with age, study estimates. *BMJ* 2020;369:m1327. doi: 10.1136/bmj.m1327
77. Fulzele S, Sahay B, Yusufu I, et al. COVID-19 virulence in aged patients might be impacted by the host cellular microRNAs abundance/profile. *Aging Dis* 2020;11(3):509-22. doi: 10.14336/ad.2020.0428
78. Koff WC, Williams MA. Covid-19 and immunity in aging populations: a new research agenda. *N Engl J Med* 2020. doi: 10.1056/NEJMp2006761
79. Watkins J. Preventing a covid-19 pandemic. *BMJ* 2020;368:m810. doi: 10.1136/bmj.m810
80. Pan D, Sze S, Minhas JS, et al. The impact of ethnicity on clinical outcomes in COVID-19: a systematic review. *EClinicalMedicine*. doi: 10.1016/j.eclinm.2020.100404
81. Lee C. "Race" and "ethnicity" in biomedical research: how do scientists construct and explain differences in health? *Soc Sci Med* 2009;68(6):1183-90. doi: 10.1016/j.socscimed.2008.12.036
82. Stead WW, Senner JW, Reddick WT, Lofgren JP. Racial differences in susceptibility to infection by Mycobacterium tuberculosis. *N Engl J Med* 1990;322(7):422-7. doi: 10.1056/nejm199002153220702
83. Nahid P, Horne DJ, Jarlsberg LG, et al. Racial differences in tuberculosis infection in United States communities: the coronary artery risk development in young adults study. *Clin Infect Dis* 2011;53(3):291-4. doi: 10.1093/cid/cir378
84. Bime C, Poongkunran C, Borgstrom M, et al. Racial differences in mortality from severe acute respiratory failure in the United States, 2008-2012. *Ann Am Thorac Soc* 2016;13(12):2184-89. doi: 10.1513/AnnalsATS.201605-359OC
85. Lanham-New SA, Webb AR, Cashman KD, et al. Vitamin D and SARS-CoV-2 virus/COVID-19 disease. *BMJ Nutrition, Prevention & Health* 2020:bmjnph-2020-000089. doi: 10.1136/bmjnph-2020-000089
86. Huang F, Zhang C, Liu Q, et al. Identification of amitriptyline HCl, flavin adenine dinucleotide, azacitidine and calcitriol as repurposing drugs for influenza A H5N1 virus-induced lung injury. *PLoS Pathog* 2020;16(3):e1008341. doi: 10.1371/journal.ppat.1008341

For peer review only - http://bmjopen.bmj.com/site/about/guidelines.xhtml

87. Lee C. Controversial effects of vitamin D and related genes on viral infections, pathogenesis, and treatment outcomes. *Nutrients* 2020;12(4):962. doi: 10.3390/nu12040962
88. Impact of zinc and vitamin D3 supplementation on the survival of aged patients infected with COVID-19. NCT04351490. ClinicalTrials.gov, U.S. National Library of Medicine; 2020. https://ClinicalTrials.gov/show/NCT04351490
89. COvid-19 and Vitamin D supplementation: a multicenter randomized controlled trial of high dose versus standard dose vitamin D3 in high-risk COVID-19 Patients (CoVitTrial). NCT04344041. ClinicalTrials.gov, U.S. National Library of Medicine; 2020. https://clinicaltrials.gov/show/NCT04344041
90. The VITDALIZE Study: effect of high-dose vitamin D3 on 28-day mortality in adult critically ill patients. NCT03188796. ClinicalTrials.gov, U.S. National Library of Medicine; 2017. https://ClinicalTrials.gov/show/NCT03188796
91. Scientific Advisory Committee on Nutrition, European Food Safety Authority. Joint explanatory note by the European Food Safety Authority and the UK Scientific Advisory Committee on Nutrition regarding dietary reference values for vitamin D. 2016. https://www.efsa.europa.eu/sites/default/files/documents/news/explanatory_note_EFSA_SACN_vitaminD.pdf

For peer review only

For peer review only - http://bmjopen.bmj.com/site/about/guidelines.xhtml

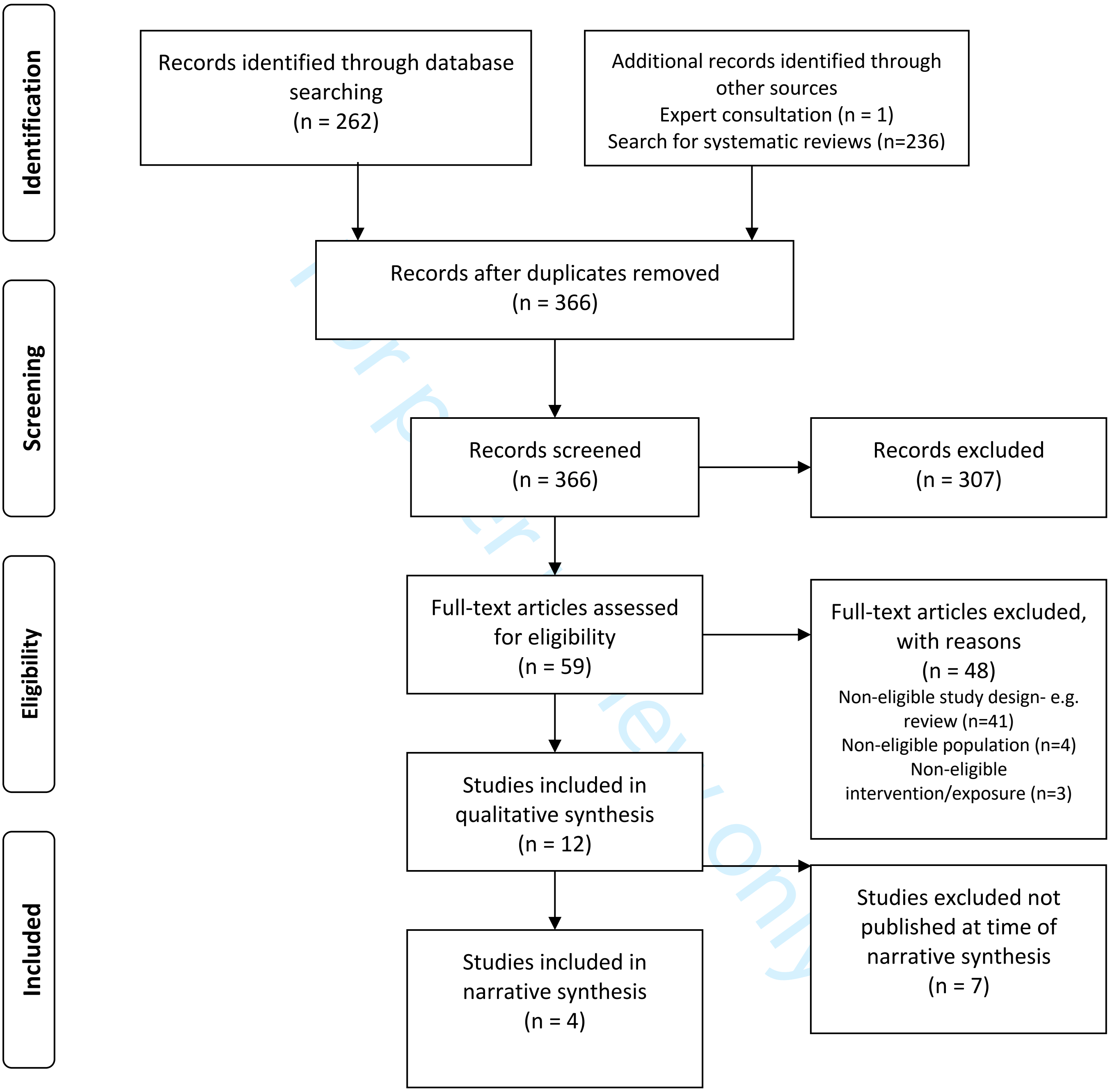

For peer review only - http://bmjopen.bmj.com/site/about/guidelines.xhtml

Supplemental file

Contents

1. Full record of search
2. Full details of the study eligibility criteria
3. List of studies excluded at full text review
4. Quality assessment of included studies

For peer review only

1. Full record of search

Medline (Ovid)

Search date: 06/05/2020
Database: Ovid MEDLINE(R) ALL <1946 to May 05, 2020>
Search Strategy:
----
1 exp Vitamin D/ (58492)
2 Vitamin D Deficiency/ (15552)
3 (vitamin D? or vit D? or D vitamin? or ergocalciferol or cholecalciferol or colecalciferol or calciferol or dihydroergocalciferol or sitocalciferol or dihydrotachysterol or dihydroxycholecalciferol? or hydroxycholecalciferol? or "25(OH)D?" or 25OHD? or calcidiol or hydroxyergocalciferol or alfacalcidol or alphacalcidol or "1,25(OH)2D" or hydroxyvitamin D? or dihydroxyvitamin D?).ab,kf,ti. (78232)
4 (paricalcitol or doxercalciferol or doxerocalciferol or calcifediol or calcitriol).ab,kf,ti. (5577)
5 hypovitaminosis D?.ab,kf,ti. (1775)
6 ((D or D2 or D3) adj3 supplement*).ab,kf,ti. (12158)
7 1 or 2 or 3 or 4 or 5 or 6 (92560)
8 coronavirus/ or betacoronavirus/ or betacoronavirus 1/ or coronavirus oc43, human/ or middle east respiratory syndrome coronavirus/ or sars virus/ (7431)
9 coronavirus infections/ or severe acute respiratory syndrome/ (10675)
10 (((corona* or corono*) adj1 (virus* or viral* or virinae*)) or coronavirus* or coronovirus* or coronavirinae* or Coronavirus* or Coronovirus* or Wuhan* or Hubei* or Huanan or "2019-nCoV" or 2019nCoV or nCoV2019 or "nCoV-2019" or "COVID-19" or COVID19 or "CORVID-19" or CORVID19 or "WN-CoV" or WNCoV or "HCoV-19" or HCoV19 or CoV or "2019 novel*" or Ncov or "n-cov" or "SARS-CoV-2" or "SARSCoV-2" or "SARSCoV2" or "SARS-CoV2" or SARSCov19 or "SARS-Cov19" or "SARSCov-19" or "SARS-Cov-19" or Ncovor or Ncorona* or Ncorono* or NcovWuhan* or NcovHubei* or NcovChina* or NcovChinese*).ab,kf,ti. (26891)
11 (severe acute respiratory syndrome or SARS or Middle East respiratory syndrome or MERS).ab,kf,ti. (16560)
12 (betacoronavirus* or betacoronavirinae*).ab,kf,ti. (280)
13 8 or 9 or 10 or 11 or 12 (37180)
14 7 and 13 (32)
15 exp Animals/ (23144176)
16 exp Humans/ (18448248)
17 15 not 16 (4695928)

18 14 not 17 (30)
19 limit 18 to yr="2002 -Current" (30)

Update
Search date: 10/6/2020
Actual databases searched: Ovid MEDLINE All <1946 to June 09, 2020>
Search strategy:
Re-ran search above plus…
20 limit 19 to ed=20200506-20200610 (8)
21 limit 19 to ep=20200506-20200610 (39)
22 limit 19 to dt=20200506-20200610 (43)
23 limit 19 to ez=20200506-20200610 (27)
24 20 or 21 or 22 or 23 (46)

Embase (Ovid)

Search date: 06/05/2020
Database: Embase <1974 to 2020 May 05>
Search Strategy:
----
1 exp vitamin D/ (139781)
2 vitamin D deficiency/ (29333)
3 (vitamin D? or vit D? or D vitamin? or ergocalciferol or cholecalciferol or colecalciferol or calciferol or dihydroergocalciferol or sitocalciferol or dihydrotachysterol or dihydroxycholecalciferol? or hydroxycholecalciferol? or "25(OH)D?" or 25OHD? or calcidiol or hydroxyergocalciferol or alfacalcidol or alphacalcidol or "1,25(OH)2D" or hydroxyvitamin D? or dihydroxyvitamin D?).ab,kw,ti. (112459)
4 (paricalcitol or doxercalciferol or doxerocalciferol or calcifediol or calcitriol).ab,kw,ti. (8478)
5 hypovitaminosis D?.ab,kw,ti. (3012)
6 ((D or D2 or D3) adj3 supplement*).ab,kw,ti. (19177)
7 1 or 2 or 3 or 4 or 5 or 6 (163395)
8 betacoronavirus 1/ or betacoronavirus/ or human coronavirus oc43/ (696)
9 Middle East respiratory syndrome coronavirus/ (2028)
10 sars-related coronavirus/ or sars coronavirus/ (6354)
11 Coronavirinae/ (2231)
12 coronavirus infection/ or middle east respiratory syndrome/ or severe acute respiratory syndrome/ (11950)
13 (((corona* or corono*) adj1 (virus* or viral* or virinae*)) or coronavirus* or coronovirus* or coronavirinae* or Coronavirus* or Coronovirus* or Wuhan* or Hubei* or Huanan or "2019-nCoV" or 2019nCoV or nCoV2019 or "nCoV-2019" or "COVID-19" or COVID19 or "CORVID-19" or CORVID19 or "WN-CoV" or WNCoV or "HCoV-19" or HCoV19 or CoV or "2019 novel*" or Ncov or "n-cov" or "SARS-CoV-2" or "SARSCoV-2" or "SARSCoV2" or "SARS-CoV2" or SARSCov19 or "SARS-Cov19" or "SARSCov-19" or "SARS-Cov-19" or Ncovor or Ncorona* or Ncorono* or NcovWuhan* or NcovHubei* or NcovChina* or NcovChinese*).ab,kw,ti. (27686)
14 (severe acute respiratory syndrome or SARS or Middle East respiratory syndrome or MERS).ab,kw,ti. (17146)
15 (betacoronavirus* or betacoronavirinae*).ab,kw,ti. (275)
16 8 or 9 or 10 or 11 or 12 or 13 or 14 or 15 (40716)
17 7 and 16 (61)
18 exp animal/ (25459151)

For peer review only - http://bmjopen.bmj.com/site/about/guidelines.xhtml

19 exp human/ (20834835)
20 18 not 19 (4624316)
21 17 not 20 (58)
22 limit 21 to yr="2002 -Current" (58)

Update
Search date: 10/6/2020
Actual databases searched: Ovid Embase <1974 to 2020 June 09>
Search strategy:
Re-ran search above plus…
22 limit 21 to yr="2002 -Current" (123)
23 limit 22 to dd=20200506-20200610 (39)
24 limit 22 to em=202005-202006 (0)
25 limit 22 to dc=20200506-20200610 (62)
26 23 or 24 or 25 (62)

MedrXiv (searched via Medrxivr https://mcguinlu.shinyapps.io/medrxivr/)

Search date: 07/05/2020
Search Strategy:

Topic 1:

[Vv]itamin D
[Vv]itamin D2
[Vv]itamin D3
calciferol
25OHD
25OHD3
[Hh]ypovitaminosis D

Topic 2:

[Cc]oronavirus
[Cc]orona(\\s)([[:graph:]]+\\s){0,1}virus
[Cc]oronavirinae
[Cc]ovid
COVID
nCoV
NCOV
Ncov
[Nn]-cov
N-COV
2019ncov
2019-ncov
ncov2019
ncov-2019
SARS
[Ss]evere [Aa]cute [Rr]espiratory [Ss]yndrome

For peer review only

[Mm]iddle [Ee]ast [Rr]espiratory [Ss]yndrome
MERS

Earliest record date
20190101
Latest record date
20200507
Remove older versions of the same record

6 results

Update
Search date: 10/6/2020
Re-ran search above changing record dates as follows:
Earliest record date
20200507
Latest record date
20200610
Remove older versions of the same record

11 results

BioRxiv
https://www.biorxiv.org/

Search date: 07/05/2020

65 Results
for abstract or title "vitamin D" (match phrase words)

22 Results
for full text or abstract or title "ergocalciferol cholecalciferol colecalciferol" (match whole any)

41 Results
for full text or abstract or title "25OHD 25OHD3" (match whole any)

Imported into EndNote and de-duplicated
92 results after deduplication

Searched in Endnote using the following search strategy:
coronavirus or corona or covid or SARS or MERS or betacoronavirus or ncov
Any Field

5 results

Update
Search date: 10/6/2020

1 Results

For peer review only

for abstract or title "vitamin D" (match phrase words) and posted between "07 May, 2020 and 10 Jun, 2020" – *animal study (also in both results sets below) so not exported to EndNote*

3 Results
for full text or abstract or title "ergocalciferol cholecalciferol colecalciferol" (match whole any) and posted between "07 May, 2020 and 10 Jun, 2020" - *2 animal studies and 1 on sertraline in TB*

2 Results
for full text or abstract or title "25OHD 25OHD3" (match whole any) and posted between "07 May, 2020 and 10 Jun, 2020" - *1 animal study, 1 non-clinical / non-coronavirus*

0 results relevant to coronaviruses

Cochrane Library

Search date: 08/05/2020

ID Search Hits
#1 MeSH descriptor: [Vitamin D] explode all trees 5224
#2 MeSH descriptor: [Vitamin D Deficiency] this term only 1226
#3 ((vitamin NEXT D?) or (vit NEXT D?) or (D NEXT vitamin?) or ergocalciferol or cholecalciferol or colecalciferol or calciferol or dihydroergocalciferol or sitocalciferol or dihydrotachysterol or dihydroxycholecalciferol? or hydroxycholecalciferol? or "25(OH)D" or "25(OH)D3" or 25OHD? or calcidiol or hydroxyergocalciferol or alfacalcidol or alphacalcidol or "1,25(OH)2D" or (hydroxyvitamin NEXT D?) or (dihydroxyvitamin NEXT D?)):ti,ab,kw 12959
#4 (paricalcitol or doxercalciferol or doxerocalciferol or calcifediol or calcitriol):ti,ab,kw 2417
#5 hypovitaminosis NEXT D? 303
#6 ((D OR D2 OR D3) NEAR/3 supplement*):ti,ab,kw 5633
#7 #1 or #2 or #3 or #4 or #5 or #6 14461
#8 MeSH descriptor: [Coronavirus] this term only 2
#9 MeSH descriptor: [Betacoronavirus] this term only 2
#10 MeSH descriptor: [Betacoronavirus 1] this term only 0
#11 MeSH descriptor: [Coronavirus OC43, Human] this term only 0
#12 MeSH descriptor: [Middle East Respiratory Syndrome Coronavirus] explode all trees 1
#13 MeSH descriptor: [SARS Virus] this term only 9
#14 MeSH descriptor: [Coronavirus Infections] this term only 137
#15 MeSH descriptor: [Severe Acute Respiratory Syndrome] this term only 107
#16 (((corona* or corono*) near/1 (virus* or viral* or virinae*)) or coronavirus* or coronovirus* or coronavirinae* or Coronavirus* or Coronovirus* or Wuhan* or Hubei* or Huanan or "2019-nCoV" or 2019nCoV or nCoV2019 or "nCoV-2019" or "COVID-19" or COVID19 or "CORVID-19" or CORVID19 or "WN-CoV" or WNCoV or "HCoV-19" or HCoV19 or CoV or "2019 novel*" or Ncov or "n-cov" or "SARS-CoV-2" or "SARSCoV-2" or "SARSCoV2" or "SARS-CoV2" or SARSCov19 or "SARS-Cov19" or "SARSCov-19" or "SARS-Cov-19" or Ncovor or Ncorona* or Ncorono* or NcovWuhan* or NcovHubei* or NcovChina* or NcovChinese*):ti,ab,kw 614
#17 ("severe acute respiratory syndrome" or SARS or "Middle East respiratory syndrome" or MERS):ti,ab,kw 350
#18 (betacoronavirus* or betacoronavirinae*):ti,ab,kw 4
#19 #8 or #9 or #10 or #11 or #12 or #13 or #14 or #15 or #16 or #17 or #18 798
#20 #7 and #19 3

For peer review only - http://bmjopen.bmj.com/site/about/guidelines.xhtml

[all 3 results were from CENTRAL]

Update
Search date: 10/06/2020
Re-ran search exactly as above and retrieved **5** results, all from CENTRAL. All 5 results exported to EndNote for deduplication.

Database of publications (living map of evidence) on coronavirus disease (COVID-19) developed by the University of Bern

Living Evidence on COVID-19
Contributors: Michel Counotte, Hira Imeri, Mert Ipekci, Nicola Low

https://zika.ispm.unibe.ch/assets/data/pub/ncov/

Search date: 10/05/2020 (14,988 entries)

Search: Title, Abstract
Search:

vitamin D 13
vitamin D2 0
vitamin D3 0
ergocalciferol 0
cholecalciferol 0
colecalciferol 0
25(OH)D 0
25OHD 0
25(OH)D3 0
25OHD3 0
hypovitaminosis D 1
Vitamin D Deficiency 1

Oxford COVID-19 Evidence Service
https://www.cebm.net/oxford-covid-19-evidence-service/
The Centre for Evidence-Based Medicine (CEBM) The University of Oxford

Search date: 10/05/2020 (142 articles)
vitamin D 1
vitamin D2 0
vitamin D3 0
ergocalciferol 0
cholecalciferol 0
colecalciferol 0
25(OH)D 0
25OHD 0
25(OH)D3 0
25OHD3 0

For peer review only - http://bmjopen.bmj.com/site/about/guidelines.xhtml

hypovitaminosis D 0
Vitamin D Deficiency 0

Database of publications on coronavirus disease (COVID-19) developed by WHO
https://www.who.int/emergencies/diseases/novel-coronavirus-2019/global-research-on-novel-coronavirus-2019-ncov

Search date: 10/05/2020 (15,253 entries)

Search: Title, Abstract, Subject

vitamin D 19
vitamin D2 0
vitamin D3 2
ergocalciferol 0
cholecalciferol 1
colecalciferol 0
25(OH)D 0
25OHD 0
25(OH)D3 0
25OHD3 0
hypovitaminosis D 1
Vitamin D Deficiency 2

Total: 25
After de-duplication: 20

Searches for systematic reviews, for reference checking

Medline

Search date: 19/05/2020
Database: Ovid MEDLINE(R) ALL <1946 to May 18, 2020>
Search Strategy:
----
1 exp Vitamin D/ (58577)
2 Vitamin D Deficiency/ (15588)
3 (vitamin D? or vit D? or D vitamin? or ergocalciferol or cholecalciferol or colecalciferol or calciferol or dihydroergocalciferol or sitocalciferol or dihydrotachysterol or dihydroxycholecalciferol? or hydroxycholecalciferol? or "25(OH)D?" or 25OHD? or calcidiol or hydroxyergocalciferol or alfacalcidol or alphacalcidol or "1,25(OH)2D" or hydroxyvitamin D? or dihydroxyvitamin D?).ab,kf,ti. (78395)
4 (paricalcitol or doxercalciferol or doxerocalciferol or calcifediol or calcitriol).ab,kf,ti. (5588)
5 hypovitaminosis D?.ab,kf,ti. (1780)
6 ((D or D2 or D3) adj3 supplement*).ab,kf,ti. (12198)
7 1 or 2 or 3 or 4 or 5 or 6 (92747)
8 coronavirus/ or betacoronavirus/ or betacoronavirus 1/ or coronavirus oc43, human/ or middle east respiratory syndrome coronavirus/ or sars virus/ (8161)
9 coronavirus infections/ or severe acute respiratory syndrome/ (11614)

For peer review only

10 (((corona* or corono*) adj1 (virus* or viral* or virinae*)) or coronavirus* or coronovirus* or coronavirinae* or Coronavirus* or Coronovirus* or Wuhan* or Hubei* or Huanan or "2019-nCoV" or 2019nCoV or nCoV2019 or "nCoV-2019" or "COVID-19" or COVID19 or "CORVID-19" or CORVID19 or "WN-CoV" or WNCoV or "HCoV-19" or HCoV19 or CoV or "2019 novel*" or Ncov or "n-cov" or "SARS-CoV-2" or "SARSCoV-2" or "SARSCoV2" or "SARS-CoV2" or SARSCov19 or "SARS-Cov19" or "SARSCov-19" or "SARS-Cov-19" or Ncovor or Ncorona* or Ncorono* or NcovWuhan* or NcovHubei* or NcovChina* or NcovChinese*).ab,kf,ti. (31115)
11 (severe acute respiratory syndrome or SARS or Middle East respiratory syndrome or MERS).ab,kf,ti. (17795)
12 (betacoronavirus* or betacoronavirinae*).ab,kf,ti. (294)
13 exp Respiratory Tract Infections/ (356696)
14 (acute respiratory infection* or severe respiratory infection* or acute respiratory tract infection* or severe respiratory tract infection* or influenza or common cold or pneumonia or bronchitis).ab,kf,ti. (234266)
15 8 or 9 or 10 or 11 or 12 or 13 or 14 (503079)
16 7 and 15 (1062)
17 (metaanalys* or "meta analys*" or "meta-analys*").tw. (169008)
18 (systematic* adj3 review*).mp. (200684)
19 meta analysis.pt. (114746)
20 17 or 18 or 19 (301767)
21 16 and 20 (55)

Embase

Search date: 19/05/2020

Database: Embase Classic+Embase <1947 to 2020 Week 20>
Search Strategy:
----
1 exp vitamin D/ (147053)
2 vitamin D deficiency/ (30106)
3 (vitamin D? or vit D? or D vitamin? or ergocalciferol or cholecalciferol or colecalciferol or calciferol or dihydroergocalciferol or sitocalciferol or dihydrotachysterol or dihydroxycholecalciferol? or hydroxycholecalciferol? or "25(OH)D?" or 25OHD? or calcidiol or hydroxyergocalciferol or alfacalcidol or alphacalcidol or "1,25(OH)2D" or hydroxyvitamin D? or dihydroxyvitamin D?).ab,kw,ti. (118981)
4 (paricalcitol or doxercalciferol or doxerocalciferol or calcifediol or calcitriol).ab,kw,ti. (8485)
5 hypovitaminosis D?.ab,kw,ti. (3033)
6 ((D or D2 or D3) adj3 supplement*).ab,kw,ti. (19335)
7 1 or 2 or 3 or 4 or 5 or 6 (172654)
8 betacoronavirus 1/ or betacoronavirus/ or human coronavirus oc43/ (1085)
9 Middle East respiratory syndrome coronavirus/ (2082)
10 sars-related coronavirus/ or sars coronavirus/ (6062)
11 Coronavirinae/ (2060)
12 coronavirus infection/ or middle east respiratory syndrome/ or severe acute respiratory syndrome/ (12565)
13 (((corona* or corono*) adj1 (virus* or viral* or virinae*)) or coronavirus* or coronovirus* or coronavirinae* or Coronavirus* or Coronovirus* or Wuhan* or Hubei* or Huanan or "2019-nCoV" or 2019nCoV or nCoV2019 or "nCoV-2019" or "COVID-19" or COVID19 or "CORVID-19" or CORVID19 or "WN-CoV" or WNCoV or "HCoV-19" or HCoV19 or CoV or "2019 novel*" or Ncov or "n-cov" or "SARS-

CoV-2" or "SARSCoV-2" or "SARSCoV2" or "SARS-CoV2" or SARSCov19 or "SARS-Cov19" or "SARSCov-19" or "SARS-Cov-19" or Ncovor or Ncorona* or Ncorono* or NcovWuhan* or NcovHubei* or NcovChina* or NcovChinese*).ab,kw,ti. (30532)
14 (severe acute respiratory syndrome or SARS or Middle East respiratory syndrome or MERS).ab,kw,ti. (17954)
15 (betacoronavirus* or betacoronavirinae*).ab,kw,ti. (286)
16 exp respiratory tract infection/ (460049)
17 (acute respiratory infection* or severe respiratory infection* or acute respiratory tract infection* or severe respiratory tract infection* or influenza or common cold or pneumonia or bronchitis).ab,kw,ti. (329779)
18 8 or 9 or 10 or 11 or 12 or 13 or 14 or 15 or 16 or 17 (674800)
19 7 and 18 (3315)
20 (metaanalys* or "meta analys*" or "meta-analys*").mp. (294469)
21 (systematic* adj2 review*).mp. (330720)
22 20 or 21 (475492)
23 19 and 22 (219)
24 limit 19 to (meta analysis or "systematic review") (145)
25 23 or 24 (219)
26 limit 25 to (conference abstract or conference paper or "conference review" or editorial or letter) (41)
27 25 not 26 (178)

Cochrane Database of Systematic Reviews (Cochrane Library)

Search Name: Vitamin D Covid and Acute Respiratory Infections SRs
Date Run: 20/05/2020 18:30:28
Comment:

ID	Search	Hits
#1	MeSH descriptor: [Vitamin D] explode all trees	5224
#2	MeSH descriptor: [Vitamin D Deficiency] this term only	1226
#3	((vitamin NEXT D?) or (vit NEXT D?) or (D NEXT vitamin?) or ergocalciferol or cholecalciferol or colecalciferol or calciferol or dihydroergocalciferol or sitocalciferol or dihydrotachysterol or dihydroxycholecalciferol? or hydroxycholecalciferol? or "25(OH)D" or "25(OH)D3" or 25OHD? or calcidiol or hydroxyergocalciferol or alfacalcidol or alphacalcidol or "1,25(OH)2D" or (hydroxyvitamin NEXT D?) or (dihydroxyvitamin NEXT D?)):ti,ab,kw	12959
#4	(paricalcitol or doxercalciferol or doxerocalciferol or calcifediol or calcitriol):ti,ab,kw	2417
#5	hypovitaminosis NEXT D?	303
#6	((D OR D2 OR D3) NEAR/3 supplement*):ti,ab,kw	5632
#7	#1 or #2 or #3 or #4 or #5 or #6	14461
#8	MeSH descriptor: [Coronavirus] this term only	2
#9	MeSH descriptor: [Betacoronavirus] this term only	2
#10	MeSH descriptor: [Betacoronavirus 1] this term only	0
#11	MeSH descriptor: [Coronavirus OC43, Human] this term only	0
#12	MeSH descriptor: [Middle East Respiratory Syndrome Coronavirus] explode all trees	1
#13	MeSH descriptor: [SARS Virus] this term only	9
#14	MeSH descriptor: [Coronavirus Infections] this term only	137
#15	MeSH descriptor: [Severe Acute Respiratory Syndrome] this term only	107

For peer review only - http://bmjopen.bmj.com/site/about/guidelines.xhtml

#16 (((corona* or corono*) near/1 (virus* or viral* or virinae*)) or coronavirus* or coronovirus* or coronavirinae* or Coronavirus* or Coronovirus* or Wuhan* or Hubei* or Huanan or "2019-nCoV" or 2019nCoV or nCoV2019 or "nCoV-2019" or "COVID-19" or COVID19 or "CORVID-19" or CORVID19 or "WN-CoV" or WNCoV or "HCoV-19" or HCoV19 or CoV or "2019 novel*" or Ncov or "n-cov" or "SARS-CoV-2" or "SARSCoV-2" or "SARSCoV2" or "SARS-CoV2" or SARSCov19 or "SARS-Cov19" or "SARSCov-19" or "SARS-Cov-19" or Ncovor or Ncorona* or Ncorono* or NcovWuhan* or NcovHubei* or NcovChina* or NcovChinese*):ti,ab,kw 616

#17 ("severe acute respiratory syndrome" or SARS or "Middle East respiratory syndrome" or MERS):ti,ab,kw 351

#18 (betacoronavirus* or betacoronavirinae*):ti,ab,kw 4

#19 MeSH descriptor: [Respiratory Tract Infections] explode all trees 14360

#20 (("acute respiratory" NEXT infection*) or ("severe respiratory" NEXT infection*) or ("acute respiratory tract" NEXT infection*) or ("severe respiratory tract" NEXT infection*) or influenza or "common cold" or pneumonia or bronchitis):ti,ab,kw 25944

#21 #8 or #9 or #10 or #11 or #12 or #13 or #14 or #15 or #16 or #17 or #18 or #19 or #20 32554

#22 #7 and #21 329

CDSR: 3

Expert consultation

One additional study identified:

Martineau AR, Jolliffe DA, Hooper RL, et al., (2017) Vitamin D supplementation to prevent acute respiratory tract infections: systematic review and meta-analysis of individual participant data. BMJ. 2017;356:i6583. doi:10.1136/bmj.i6583

For peer review only

For peer review only - http://bmjopen.bmj.com/site/about/guidelines.xhtml

2. Full details of the study eligibility criteria

Include	Exclude
**P- Population**<br>1) Patients acutely ill with Betacoronavirus infection [SARS-CoV, MERS-CoV, SARS-CoV-2]<br>2) or at risk of acute illness with Betacoronavirus infection	Animals studies, modelling studies
**I – Intervention/exposure**<br>1) Vitamin D supplementation<br>2) Low Serum Vitamin D	
**O - Outcomes**<br>1) Betacoronavirus infection (to include serological evidence of infection or clinically confirmed symptomatic infection);<br>2) severity of Betacoronavirus infection (to include patients admitted to hospital or admitted to intensive care); mortality due to Betacoronavirus.<br>3) Mortality due to Betacoronavirus	
**C – Comparator**<br>1) No Vitamin D supplementation<br>2) high or normal Serum Vitamin D	
**S - Study design**<br>Randomised controlled trials and non-randomized studies will be eligible for inclusion in the review including, non randomized controlled trials, interrupted time series, controlled before-and-after studies, cohort studies, ecological studies, case reports and case series.	Qualitative studies, Non-primary research- reviews, editorials etc, guidelines and non-systematic reviews.
**Subgroups**<br>1. Ethnicity characteristics (White British, All Other White, Mixed, Asian, Black, Other)<br>2. Age characteristics (population by five-year age groups)	Non-English language. Non peer reviewed publication.

For peer review only

For peer review only - http://bmjopen.bmj.com/site/about/guidelines.xhtml

3. List of studies excluded at full text review

Excluded studies		Reason<br><br>*Non-eligible study design- e.g. review*<br>*Non-eligible population*<br>*Non-eligible intervention*<br>*No relevant outcome*<br>*No comparator group*
1	Adams, K. K., et al. (2020). "Myth Busters: Dietary Supplements and COVID-19." Annals of Pharmacotherapy: 1060028020928052.	Study design – commentary
2	Ahmed, I., et al. (2020). "First Covid-19 maternal mortality in the UK associated with thrombotic complications." British Journal of Haematology. **18**.	Study design – letter
3	Alpalhao, M. and P. Filipe (2020). "SARS-CoV-2 pandemic and Vitamin D deficiency - a double trouble." Photodermatology, Photoimmunology & Photomedicine **01**: 01.	Study design – letter
4	Annweiler, C., et al. (2020). COvid-19 and Vitamin D Supplementation: a Multicenter Randomized Controlled Trial of High Dose Versus Standard Dose Vitamin D3 in High-risk COVID-19 Patients (CoVitTrial). https://clinicaltrials.gov/show/NCT04344041.	Study design – on-going RCT completion date. July 2020
5	Arya, A. and V. D. Dwivedi (2020). "Synergistic effect of vitamin D and remdesivir can fight COVID-19." Journal of Biomolecular Structure & Dynamics: 1-2	Study design – letter
6	Banerjee, D., et al. (2020). "COVID-19 infection in kidney transplant recipients." Kidney International **97**(6): 1076-1082.	Study design – commentary
7	Caccialanza, R., et al. (2020). Early nutritional supplementation in non-critically ill patients hospitalized for the 2019 novel coronavirus disease (COVID-19): Rationale and feasibility of a shared pragmatic protocol. Nutrition: 110835.	Study design - protocol
8	Calder, P. C., et al. (2020). Optimal Nutritional Status for a Well-Functioning Immune System Is an Important Factor to Protect against Viral Infections. Nutrients: 12(4), 1181.	Study design -narrative review
9	Cao, Z., et al. (2020). SARS-CoV-2 & Covid-19: Key-Roles of the 'Renin-Angiotensin' System / Vitamin D Impacting Drug and Vaccine Developments. Infectious Disorders - Drug Targets. *(E-pub Ahead of Print)* DOI : 10.2174/1871526520999200505174704	Study design – editorial
10	Carter, S. J., et al. (2020). Considerations for obesity, vitamin D, and physical activity amidst the COVID-19 pandemic. Obesity **16**: 16.	Study design -narrative review

For peer review only - http://bmjopen.bmj.com/site/about/guidelines.xhtml

11	Daneshkhah, A., et al. (2020). The Possible Role of Vitamin D in Suppressing Cytokine Storm and Associated Mortality in COVID-19 Patients. medRxiv. DOI: https://doi.org/10.1101/2020.04.08.20058578	Study design – modelling
12	Davies G, Garami AR, Byers JC. Evidence Supports a Causal Model for Vitamin D in COVID-19 Outcomes. medRxiv, 2020. DOR: https://doi.org/10.1101/2020.05.01.20087965v3	Study design – modelling
13	de Lucena, T. M. C., et al. (2020). "Mechanism of inflammatory response in associated comorbidities in COVID-19." Diabetes & Metabolic Syndrome **14**(4): 597-600.	Study design -narrative review
14	Eroglu, C., et al. (2019). The relation between serum vitamin D levels, viral infections and severity of attacks in children with recurrent wheezing. Allergologia et Immunopathologia **47**(6): 591-597.	Population - Not COVID-19/SARs/MERs
15	Faul, J. L., et al. (2020). "Vitamin d deficiency and ards after sars-cov-2 infection." Irish Medical Journal **113**(5).	Study design – letter
16	Ghasemian, R., et al. (2020). "The Role of Vitamin D in The Age of COVID-19: A Systematic Review and Meta-Analysis Along with an Ecological Approach." MedRxiv : the Preprint Server for Health Sciences. DOI: 10.1101/2020.06.05.20123554	Study design -narrative review
17	Grant, W. B., et al. (2020). Evidence that Vitamin D Supplementation Could Reduce Risk of Influenza and COVID-19 Infections and Deaths. Nutrients **12**(4): 02.	Study design -narrative review
18	Heiser, K., et al. (2020). Identification of potential treatments for COVID-19 through artificial intelligence-enabled phenomic analysis of human cells infected with SARS-CoV-2. bioRxiv: 2020.2004.2021.054387.	Study design – modelling
19	Hribar, C. A., et al. (2020). "Potential Role of Vitamin D in the Elderly to Resist COVID-19 and to Slow Progression of Parkinson's Disease." Brain Sciences **10**(5): 08.	Study design -narrative review
20	Jakovac, H. (2020). COVID-19 and vitamin D-Is there a link and an opportunity for intervention? American Journal of Physiology - Endocrinology & Metabolism **318**(5): E589-E589.	Study design – letter
21	Jamaati, H., et al. (2020). A fourteen-day experience with coronavirus disease 2019 (COVID-19) induced acute respiratory distress syndrome (ARDS): An Iranian treatment protocol. Iranian Journal of Pharmaceutical Research **19**(1): 31-36.	Study design – treatment protocol/recommendation
22	Kalippurayil Moozhipurath, R., et al. (2020). "Evidence of Protective Role of Ultraviolet-B (UVB) Radiation in	Exposure – not vitamin D(UVB)

For peer review only

	Reducing COVID-19 Deaths." MedRxiv : the Preprint Server for Health Sciences. DOI 10.1101/2020.05.06.20093419	
23	Kara, M., et al. (2020). "'Scientific Strabismus' or Two Related Pandemics: COVID-19 & Vitamin D Deficiency." British Journal of Nutrition: 1-20.	Study design -narrative review
24	Koivisto, O., et al. (2020). Key Vitamin D Target Genes with Functions in the Immune System. Nutrients, **12**(4):1140.	Population - Outcomes - target genes
25	Kow, C. S., et al. (2020). "Vitamin D Supplementation in Influenza and COVID-19 Infections Comment on: "Evidence that Vitamin D Supplementation Could Reduce Risk of Influenza and COVID-19 Infections and Deaths" Nutrients 2020, 12(4), 988." Nutrients **12**(6): 01.	Study design – commentary
26	Kumar, V. and A. Srivastava (2020). "Spurious Correlation? A review of the relationship between Vitamin D and Covid-19 infection and mortality." MedRxiv : the Preprint Server for Health Sciences. DOI: 10.1101/2020.05.25.20110338	Study design -narrative review
27	Laird, E., et al. (2020). "Vitamin D and inflammation: Potential implications for severity of Covid-19." Irish Medical Journal **113**(5).	Study design -narrative review
28	La Vignera, S., et al. (2020). Sex-Specific SARS-CoV-2 Mortality: Among Hormone-Modulated ACE2 Expression, Risk of Venous Thromboembolism and Hypovitaminosis D. International journal of molecular sciences **21**(8):2948.	Study design – editorial
29	Li, A. Y., et al. (2020). Multivariate Analysis of Factors Affecting COVID-19 Case and Death Rate in U.S. Counties: The Significant Effects of Black Race and Temperature. medRxiv. DOI: https://doi.org/10.1101/2020.04.17.20069708	Intervention – not Vitamin D
30	Maestri, E., et al. (2020). Vitamin D and coronavirus: a new field of use?. Recenti Progressi in Medicina **111**(4): 253-256.	Study design -narrative review, non-English
31	Mansbach, J. M. and C. A. Camargo Jr., (2009). Respiratory Viruses in Bronchiolitis and Their Link to Recurrent Wheezing and Asthma. Clinics in Laboratory Medicine **29**(4): 741-755.	Population - Not COVID-19/SARs/MERs
32	Marik, P. E., et al. (2020). Does vitamin D status impact mortality from SARS-CoV-2 infection? Medicine in Drug Discovery: 100041-100041.	Study design - commentary
33	McKenna, M. J. and M. A. T. Flynn (2020). "Covid-19, cocooning and vitamin d intake requirements." Irish Medical Journal **113**(5).	Study design -narrative review

For peer review only

34	Mitchell, F. (2020). "Vitamin-D and COVID-19: do deficient risk a poorer outcome?" The Lancet Diabetes & Endocrinology **20**: 20.	Study design -narrative review
35	Molloy, E. J. and N. Murphy (2020). Vitamin D, Covid-19 and Children. Irish Medical Journal **113**(4): 64.	Study design -narrative review
36	McCartney, D. M. and D. G. Byrne (2020). Optimisation of Vitamin D Status for Enhanced Immuno-protection Against Covid-19. Irish Medical Journal **113**(4): 58.	Study design -narrative review
37	Rabbitt, L. and E. Slattery (2020). "Vitamin d and covid-19: A note of caution." Irish Medical Journal **113**(5).	Study design - letter
38	Ribeiro, H., et al. (2020). "Does Vitamin D play a role in the management of Covid-19 in Brazil?" Revista de Saude Publica **54**: 53.	Study design -narrative review
39	Romano, L., et al. (2020). "Short Report - Medical nutrition therapy for critically ill patients with COVID-19." European Review for Medical and Pharmacological Sciences **24**(7): 4035-4039.	Study design -narrative review
40	Silberstein, M. (2020). Vitamin D: A simpler alternative to tocilizumab for trial in COVID-19? Medical Hypotheses **140**: 109767-109767.	Study design -narrative review
41	Speeckaert, M. M. and J. R. Delanghe (2020). "Association between low vitamin D and COVID-19: don't forget the vitamin D binding protein." Aging Clinical & Experimental Research **28**: 28.	Study design - letter
42	Skutsch, M., et al. (2020). "The association of UV with rates of COVID-19 transmission and deaths in Mexico: the possible mediating role of vitamin D." MedRxiv : the Preprint Server for Health Sciences. DOI: 10.1101/2020.05.25.20112805	Study design – modelling
43	Suresh, P. S. (2020). "Hypovitaminosis D and COVID-19: Matter of Concern in India?" Indian Journal of Clinical Biochemistry.	Study design - letter
44	Taghizadieh, A., et al. (2020). "Acute kidney injury in pregnant women following SARS-CoV-2 infection: A case report from Iran." Respiratory Medicine Case Reports 30	Exposure – not vitamin D
45	Tan, S. H. S., et al. (2020). "Medications in COVID-19 patients: summarizing the current literature from an orthopaedic perspective." International Orthopaedics **22**: 22.	Study design -narrative review
46	Yalaki, Z., et al. (2019). Comparison of viral agents and vitamin D levels in children with acute bronchiolitis infection. Cocuk Enfeksiyon Dergisi **13**(1): e14-e20.	Population - COVID-19/SARs/MERs not specified
47	Zabetakis, I., et al. (2020). "COVID-19: The Inflammation Link and the Role of Nutrition in Potential Mitigation." Nutrients **12**(5): 19.	Study design -narrative review

For peer review only - http://bmjopen.bmj.com/site/about/guidelines.xhtml

48	Zemb, P., et al. (2020). "Vitamin D deficiency and COVID-19 pandemic." Journal of Global Antimicrobial Resistance **28**: 28.	Study design - commentary

4. **Articles included at full text, but later excluded at time of narrative synthesis**

49	Darling, A. L., et al. (2020). Vitamin D status, body mass index, ethnicity and COVID-19: Initial analysis of the first-reported UK Biobank COVID-19 positive cases (n 580) compared with negative controls (n 723). medRxiv.<br><br>https://www.medrxiv.org/content/10.1101/2020.04.29.20084277v1?versioned=TRUE	Not peer reviewed publication at time of narrative synthesis
50	De Smet, D., et al. (2020). Vitamin D deficiency as risk factor for severe COVID-19: a convergence of two pandemics. medRxiv.<br><br>https://www.medrxiv.org/content/10.1101/2020.05.01.20079376v2	Not peer reviewed publication at time of narrative synthesis
51	Lau, F. H., et al. (2020). Vitamin D Insufficiency is Prevalent in Severe COVID-19. medRxiv.<br><br>https://www.medrxiv.org/content/10.1101/2020.04.24.20075838v1?versioned=TRUE	Not peer reviewed publication at time of narrative synthesis
52	Meltzer, D. O., et al. (2020). "Association of Vitamin D Deficiency and Treatment with COVID-19 Incidence." MedRxiv : the Preprint Server for Health Sciences **13**: 13.<br><br>https://www.medrxiv.org/content/10.1101/2020.05.08.20095893v1	Not peer reviewed publication at time of narrative synthesis
53	Notari, A. and G. Torrieri (2020). "COVID-19 transmission risk factors." MedRxiv : the Preprint Server for Health Sciences.<br><br>https://www.medrxiv.org/content/10.1101/2020.05.08.20095083v1?versioned=TRUE	Not peer reviewed publication at time of narrative synthesis
54	Raisi-Estabragh, Z., et al. (2020). "Greater risk of severe COVID-19 in non-White ethnicities is not explained by cardiometabolic, socioeconomic, or behavioural factors, or by 25(OH)-vitamin D status: study of 1,326 cases from the UK Biobank." MedRxiv : the Preprint Server for Health Sciences.	Not peer reviewed publication at time of

For peer review only - http://bmjopen.bmj.com/site/about/guidelines.xhtml

	https://www.medrxiv.org/content/10.1101/2020.06.01.20118943v1?versioned=TRUE	narrative synthesis
55	Tan, C. W., et al. (2020). "A cohort study to evaluate the effect of combination Vitamin D, Magnesium and Vitamin B12 (DMB) on progression to severe outcome in older COVID-19 patients." MedRxiv : the Preprint Server for Health Sciences.<br><br>https://www.medrxiv.org/content/10.1101/2020.06.01.20112334v2	Not peer reviewed publication at time of narrative synthesis

For peer review only

Study details of the seven articles included at full text, but excluded at time of narrative synthesis						
**Study**	**Design/ setting**	**Population**	**Exposure/Intervention**	**Outcomes**	**Results**	**Limitations**
Darling, A. L., et al. (2020).	Retrospective cohort study<br><br>UK Biobank England cohort only	COVID-19 positive cases (n 580) Mean age 57.5 (SD 9.7)<br><br>COVID-19 negative controls (n 723) Mean age 57.9 (SD 8.7)	Serum 25(OH)D status Median (IQR) nmol/L<br><br>by gender (Male/Female), body mass index (Normal/underweight, overweight, obesity), ethnicity (Asian, Black, Mixed and Other, White)	COVID-19 test result	Serum 25(OH)D status similar in both groups:<br><br>COVID-19 positive cases (median IQR) = 43.3 (32.1) nmol/L)<br><br>COVID-19 negative controls (median (IQR) 44.1 (31.2) nmol/L) for COVID-19.<br><br>A logistic regression model suggests that being overweight (OR 1.51 CI 1.13-2.02) or obese (OR 1.67 CI 1.24-2.26); living in London (OR 1.45 CI 1.05-2.00); being male (OR 1.28 CI 1.01-1.61) and being of Asian, Black or Mixed ethnicity (OR 1.66 CI 1.08-2.54) is associated with a higher odds of testing positive for COVID-19	UK Biobank baseline samples collected in 2006-2010.
De Smet, D., et al. (2020).	Retrospective observational study<br><br>Central network hospital, West Flanders, Belgium	186 SARS-CoV-2 infected patients hospitalised from March 1, 2020 to April 7, 2020<br><br>(109 males [median age 68 years, IQR 53-79] 77 females [median age 71 years, IQR 65-74])<br><br>25(OH)D in COVID-19 patients was compared a control group of 2717 patients with similar age distribution, sampled from March 1, 2019 to April 30, 2019. (999 males [median age 69 years, IQR 53-81] and 1718 females [median age 68 years, IQR 43-83]).	25(OH)D levels	SARS-CoV-2 infection	COVID-19 patients had a lower median 25(OH)D on admission (18.6 ng/mL, IQR 12.6-25.3) than controls (21.5 ng/mL, IQR 13.9-20.8, P=0.0016) and a higher percentage of vitamin D deficiency (defined as 25(OH)D < 20ng/mL): 58.6% versus 45.2% (P=0.0005).<br><br>In male COVID-19 patients, vitamin D deficiency was lower median 25(OH)D (17.6 ng/mL, IQR 12.7-24.0 versus 20.3 ng/mL, IQR 13.7-28.3, P=0.0234) and a higher deficiency rate (67.0% versus 49.2%, P=0.0006) than male controls.	The prevalence and age/sex/ seasonal-distribution of vitamin D status was derived from the general population sampled from 16274 consecutive, unselected and unique patients from January 1, 2019 to December 31, 2019.

For peer review only - http://bmjopen.bmj.com/site/about/guidelines.xhtml

Lau, F. H., et al. (2020).	Retrospective observational study<br><br>A single, tertiary care academic (university) medical centre, Louisiana, New Orleans, USA	COVID-19 ICU patients (n 13) Mean age 61.5 (SD 15.7)<br><br>COVID-19 floor patients (n 7) Mean age 72.0 (SD 14.8)<br><br>Medical records of COVID-19 patients between March 27, 2020 and April 21, 2020<br><br>Vitamin D insufficiency (VDI) and COVID-19 metrics in ICU vs. floor patients	VDI: defined as serum 25(OH) D < 30 ng/mL)<br><br>Serum 25(OH) D status Mean (SD) ng/mL<br><br>by gender (Male/Female), body mass index (Normal, obesity), race (African American), age (elderly>65 years), hypertension	COVID-19 metrics	Overall, few significant differences were identified between ICU and floor patients: Lactate dehydrogenase was significantly higher among ICU patients (441.8 vs. 223.0, P=0.001). Also, body mass index was significantly higher among ICU patients (35.2 vs. 24.5, P=0.02).<br><br>Among ICU subjects, 11 (84.6%) had VDI, vs. 4 (57.1%) of floor subjects. 100% of ICU patients less than 75 years old had VDI (n=11). Among these, 64.6% (n=7) had critically low 25(OH) D (<20 ng/mL) and 3 had <10 ng/mL.<br><br>VDI is highly prevalent in dark-skinned people (82.1% of African Americans vs. 41.6% overall).<br><br>Male/Female ratio was 1.24 and 1.44 for COVID-19 and VDI respectively.	Statistical analysis was limited by the small number of subjects.
Meltzer, D. O., et al. (2020).	Retrospective cohort study<br><br>University of Chicago Medicine, USA	4,314 patients tested for COVID-19 from 3/3/2020 to 4/10/2020. Among these, 499 had a vitamin D level in the year before testing.<br><br>COVID-19 positive cases with vitamin D deficient (n 178) Mean age 45.6<br><br>COVID-19 positive cases with not vitamin D deficient (n 321) Mean age 50.7	Vitamin D deficiency: defined by the most recent 25(OH) D <20ng/ml or 1,25-dihydroxycholecalciferol <18pg/ml within 1 year before COVID-19 testing. Treatment: defined by the most recent vitamin D type and dose, and treatment changes between the time of the most recent vitamin D level and time of COVID-19 testing<br><br>Vitamin D deficiency and treatment changes were combined to categorize vitamin D status at the time of COVID-19 testing as:<br>1)Likely deficient (last-level-deficient/treatment-not-increased)	Testing positive for COVID-19	In multivariable analysis, testing positive for COVID-19 was associated with increasing age (RR (age<50)=1.05, P<0.021; RR (age≥50)=1.02, P<0.064)), non-white race (RR=2.54, P<0.01) and being likely vitamin D deficient (deficient/treatment-not-increased: RR=1.77, P<0.02) as compared to likely vitamin D sufficient (not-deficient/treatment-not-decreased), with predicted COVID-19 rates in the vitamin D deficient group of 21.6% (95%CI [14.0%-29.2%]) vs 12.2% (95%CI [8.9%-15.4%]) in the vitamin D sufficient group.<br><br>Vitamin D deficiency declined with increasing vitamin D dose (especially of vitamin D3). Vitamin D dose was not significantly associated with testing positive for COVID-19 (P=0.18).	The associations observed might not reflect causal effects of vitamin D deficiency on COVID-19. This is because vitamin D deficiency can reflect a range of chronic health conditions or behavioural factors which plausibly decrease the likelihood of treatment of vitamin D

For peer review only - http://bmjopen.bmj.com/site/about/guidelines.xhtml

			2)Likely sufficient (last-level-not-deficient/treatment-not-decreased)<br>3)Uncertain deficiency (last-level-deficient/treatment-increased or last-level-not-deficient/treatment-decreased)<br><br>by age (<50, ≥50), gender (Male/Female), race (White, other than White), ethnicity (Hispanic, not Hispanic), body mass index, employee status, comorbidity indicators (e.g. hypertension)			deficiency and increase COVID-19 risk.
Notari, A. and G. Torrieri (2020).	Correlational study<br><br>126 countries, Only 50 countries for vitamin D	The number of cases follows in its early stages an almost exponential expansion. A starting point in each country was chosen: the first day di with 30 cases and fitted for 12 days. Thus, capturing the early exponential growth.<br><br>Countries with too small total population (less than 300 thousands inhabitants) were excluded.	They analysed risk factors correlated with the initial transmission growth rate of COVID-19<br><br>Average annual level of serum Vitamin D and the seasonal level<br><br>The seasonal level is defined as: the amount during March or during winter for northern hemisphere, or during summer for southern hemisphere or the annual level for countries with little seasonal variation.	Growth rate of COVID-19	They looked for linear correlations of the exponents with other variables, for a sample of 126 countries.<br><br>They found a positive correlation, i.e. faster spread of COVID-19, with high confidence level with the following variables, with respective p-value: low Temperature (4.10^{-7}), high ratio of old vs. working-age people (3.10^{-6}), life expectancy (8.10^{-6}), number of international tourists (1.10^{-5}), earlier epidemic starting date di (2.10^{-5}), high level of physical contact in greeting habits (6.10^{-5}), lung cancer prevalence (6.10^{-5}), obesity in males (1.10^{-4}), share of population in urban areas (2.10^{-4}), cancer prevalence (3.10^{-4}), alcohol consumption (0.0019), daily smoking prevalence (0.0036), UV index (0.004, smaller sample, 73 countries), low Vitamin D serum levels (0.002-0.006, smaller sample, 50 countries). There is highly significant correlation also with blood type. Also, positive correlation with moderate CI (p-value of 0.02-0.03) with: CO2/SO emissions, type-1 diabetes in children, and low vaccination coverage for Tuberculosis (BCG).	The dataset for the annual vitamin D was built with the available literature, which is quite inhomogeneous.<br><br>The dataset for the seasonal levels is more restricted. This is because the relative literature is less complete. So, for this the authors have included only 42 countries.

For peer review only

					Vitamin D is not highly correlated with UV index due to different food consumption in different countries.	
Raisi-Estabragh, Z., et al. (2020).	Retrospective cohort study<br><br>UK Biobank	4,510 UK participants tested for COVID-19.<br><br>Latest data release (29/05/2020) includes test results from 16/03/2020 to 18/05/2020.<br><br>COVID-19 positive cases (n 1,326) Mean age 68.11 (SD 9.23)<br><br>COVID-19 negative controls (n 3,184) Mean age 68.91 (SD 8.72)	Serum 25(OH) D levels nmol/L<br><br>Multivariate logistic regression models by age, gender (Male/Female), ethnicity (Caucasian (any White background) and non-Caucasian: Black, Asian, Chinese) to test whether addition of: 1)cardio metabolic factors (e.g. hypertension, body mass index); 2) 25(OH)-vitamin D; 3) poor diet; 4) Townsend deprivation score; 5) housing; or 6)behavioural factors attenuated sex/ethnicity associations with COVID-19 status	COVID-19 test result<br><br>Greater risk of severe COVID-19	Over-representation of men and non-White ethnicities in the COVID-19 positive group.<br><br>Non-Whites had, on average, poorer cardio metabolic profile, lower 25(OH)-vitamin D, greater material deprivation, and were more likely to live in larger households and flats.<br><br>Male sex, non-White ethnicity, higher body mass index, Townsend deprivation score, and household overcrowding were independently associated with significantly greater odds of COVID-19. The pattern of association was consistent for men and women; cardio metabolic, socio-demographic and behavioural factors did not attenuate sex/ethnicity associations.	UK Biobank baseline samples collected in 2006-2010.<br><br>Aggregating all Black and Minority Ethnic (BAME) populations into one cohort might overlook important differences between non-Caucasian ethnicities.<br><br>The current dataset does not allow assessment of specific COVID-19 health outcomes.
Tan, C. W., et al. (2020).	Cohort observational study<br><br>A tertiary academic hospital, Singapore	All 43 consecutive hospitalized COVID-19 patients aged 50 and above.<br><br>Between 15 January and 15 April 2020.	DMB = a single daily oral dose of vitamin D3 1000 IU, magnesium 150mg and vitamin B12 500mcg for up to 14 days<br><br>Adjusted for age, gender and comorbidities	Deterioration post-DMB administration leading to any form of oxygen therapy and/or intensive care	Duration of therapy: days, Median 5 (IQR 4-7)<br><br>Significantly fewer DMB patients than controls required initiation of oxygen therapy subsequently throughout their hospitalization (17.6% vs 61.5%, P=0.006).<br><br>On univariate analysis, increasing age and presence of comorbidities were associated	Small sample size, and the lack of systematic biologic measures to support their findings.

For peer review only - http://bmjopen.bmj.com/site/about/guidelines.xhtml

		17 patients received Vitamin D, Magnesium, Vitamin B12 (DMB): Mean age 58.4 (SD 7.0)<br><br>26 patients did not: Mean age 64.1 (SD 7.9)		support for COVID-19 patients	with significantly higher OR for oxygen therapy, while exposure to DMB therapy was associated with a significantly improved OR 0.13 (95% CI: 0.03 – 0.59, P=0.008) .<br><br>On multivariate analysis, increasing age was associated with significantly higher OR for oxygen therapy, while exposure to DMB therapy was associated with a significantly improved OR 0.15 (95% CI: 0.025 – 0.93, P=0.041).	

For peer review only

5. Risk of bias of included studies

Risk of bias assessment using the Downs and Black Checklist[61]

Study	Quality score	Reviewer notes
Hastie et al., 2020[69]	14/20<br><br>Seven domains were not applicable and therefore not assessed, 2 reporting, 1 external validity 3 internal validity (bias) and 1 internal validity (confounding).	The study could not be scored for 3 questions as we were unable to determine; 1) the representativeness of the subjects who were prepared to participate from entire population from which they were recruited, 2) whether losses to follow-up were taken into account as patients lost to follow-up were not reported and 3) whether the study had sufficient power to detect a clinically important effect.<br><br>The study did not score a point for 3 questions; 1) providing the number and a description of the characteristics of patients lost to follow-up, 2) stating whether study subjects in different intervention groups we recruited over the same period of time and 3) for assignment concealment as it was a non-randomised study. The study scored partially (only 1 point not two) for clearly described distributions of principal confounders in each group of subjects to be compared.
D'Avolio et al, 2020[72]	13/15<br><br>Twelve domains were not applicable therefore not assessed, 3 reporting, 1 external validity, 4 internal validity (bias) and 4 internal validity (confounding).	The study could not be scored for the 'power' domain as we were unable to determine from the article whether the study had sufficient power to detect a clinically important effect where the probability value for a difference being due to chance is less than 5%.<br><br>The study did not score 1 point in the 'external validity' domain as those subjects who were prepared to participate were not representative of the entire population from which they were recruited.
Fasano et al., 2020[73]	12/17<br>Ten domains were not applicable therefore not assessed, 3 reporting, 1 external validity, 4 internal validity (bias) and 2 internal validity (confounding).	The study could not be scored for 4 items, the 'power' domain and one question in the 'Internal validity - confounding (selection bias)' as the study did not specify the time period over which patients were recruited. It could also not be scored for 2 questions in the 'External validity domain', 1) the representativeness of the subjects asked to participate in the study compared to the entire population from which they were recruited and 2) the representativeness of those subjects who were prepared to participate compared to the entire population from which they were recruited.<br><br>The study did not score 1 point as the effect of the main confounders was not investigated or confounding was demonstrated but no adjustment was made in the final analyses. The study scored two points for presentation of potential confounders.

For peer review only - http://bmjopen.bmj.com/site/about/guidelines.xhtml

Ilie et al., 2020[70]	4/20<br>Seven domains were not applicable and therefore not assessed, 1 reporting, 1 external validity, 3 internal validity (bias), 1 internal validity (confounding) and 1 for power.	The study could not be scored for 9 questions. Two in the 'reporting' domain, 1) interventions of interest not clearly described, 2) the main findings of the study are not clearly described. Two 'External validity' questions 1) the representativeness of the subjects asked to participate in the study compared to the entire population from which they were recruited and 2) the representativeness of those subjects who were prepared to participate compared to the entire population from which they were recruited. Two 'Internal validity – bias' domain questions 1) all analyses that had not been planned at the outset of the study were not clearly indicated (results of the study based on "data dredging", were not made clear), and 2) it was not clear is the statistical techniques used were appropriate to the data. Three 'Internal validity - confounding (selection bias)' domain questions, 1) no information provided concerning the source of patients included in the study 2) does not specify the time period over which patients were recruited, and 3) the numbers of patients lost to follow-up are not reported.<br><br>The study did not score 7 points for the following; 3 reporting issues 1) no description of the characteristics of participants included in the study 2) no description of the distributions of principal confounders in each group of subjects to be compared, and 3) no description of the characteristics of patients lost to follow-up. Two internal validity bias issues 1) differences in follow-up were ignored and 2) no evidence that the main measure used were accurate (valid and reliable).

For peer review only

PRISMA 2009 Checklist

Section/topic	#	Checklist item	Reported on page #
**TITLE**			
Title	1	Identify the report as a systematic review, meta-analysis, or both.	1
**ABSTRACT**			
Structured summary	2	Provide a structured summary including, as applicable: background; objectives; data sources; study eligibility criteria, participants, and interventions; study appraisal and synthesis methods; results; limitations; conclusions and implications of key findings; systematic review registration number.	3
**INTRODUCTION**			
Rationale	3	Describe the rationale for the review in the context of what is already known.	5
Objectives	4	Provide an explicit statement of questions being addressed with reference to participants, interventions, comparisons, outcomes, and study design (PICOS).	6
**METHODS**			
Protocol and registration	5	Indicate if a review protocol exists, if and where it can be accessed (e.g., Web address), and, if available, provide registration information including registration number.	6
Eligibility criteria	6	Specify study characteristics (e.g., PICOS, length of follow-up) and report characteristics (e.g., years considered, language, publication status) used as criteria for eligibility, giving rationale.	7
Information sources	7	Describe all information sources (e.g., databases with dates of coverage, contact with study authors to identify additional studies) in the search and date last searched.	6
Search	8	Present full electronic search strategy for at least one database, including any limits used, such that it could be repeated.	Supp appendix 1
Study selection	9	State the process for selecting studies (i.e., screening, eligibility, included in systematic review, and, if applicable, included in the meta-analysis).	7 and supp appendix 2
Data collection process	10	Describe method of data extraction from reports (e.g., piloted forms, independently, in duplicate) and any processes for obtaining and confirming data from investigators.	8
Data items	11	List and define all variables for which data were sought (e.g., PICOS, funding sources) and any assumptions and simplifications made.	8
Risk of bias in individual studies	12	Describe methods used for assessing risk of bias of individual studies (including specification of whether this was done at the study or outcome level), and how this information is to be used in any data synthesis.	8

For peer review only

PRISMA 2009 Checklist

Summary measures	13	State the principal summary measures (e.g., risk ratio, difference in means).	8
Synthesis of results	14	Describe the methods of handling data and combining results of studies, if done, including measures of consistency (e.g., I^2) for each meta-analysis.	8

Section/topic	#	Checklist item	Reported on page #
Risk of bias across studies	15	Specify any assessment of risk of bias that may affect the cumulative evidence (e.g., publication bias, selective reporting within studies).	n/a
Additional analyses	16	Describe methods of additional analyses (e.g., sensitivity or subgroup analyses, meta-regression), if done, indicating which were pre-specified.	8
**RESULTS**			
Study selection	17	Give numbers of studies screened, assessed for eligibility, and included in the review, with reasons for exclusions at each stage, ideally with a flow diagram.	8
Study characteristics	18	For each study, present characteristics for which data were extracted (e.g., study size, PICOS, follow-up period) and provide the citations.	8 and supp appendix 3
Risk of bias within studies	19	Present data on risk of bias of each study and, if available, any outcome level assessment (see item 12).	8/9 supp appendix 4
Results of individual studies	20	For all outcomes considered (benefits or harms), present, for each study: (a) simple summary data for each intervention group (b) effect estimates and confidence intervals, ideally with a forest plot.	8/9/10 and supp appendix 3
Synthesis of results	21	Present results of each meta-analysis done, including confidence intervals and measures of consistency.	n/a
Risk of bias across studies	22	Present results of any assessment of risk of bias across studies (see Item 15).	n/a
Additional analysis	23	Give results of additional analyses, if done (e.g., sensitivity or subgroup analyses, meta-regression [see Item 16]).	10
**DISCUSSION**			
Summary of evidence	24	Summarize the main findings including the strength of evidence for each main outcome; consider their relevance to key groups (e.g., healthcare providers, users, and policy makers).	11/12/13
Limitations	25	Discuss limitations at study and outcome level (e.g., risk of bias), and at review-level (e.g., incomplete retrieval of identified research, reporting bias).	13

For peer review only - http://bmjopen.bmj.com/site/about/guidelines.xhtml

PRISMA 2009 Checklist

Conclusions	26	Provide a general interpretation of the results in the context of other evidence, and implications for future research.	13/14
**FUNDING**			
Funding	27	Describe sources of funding for the systematic review and other support (e.g., supply of data); role of funders for the systematic review.	15

From: Moher D, Liberati A, Tetzlaff J, Altman DG, The PRISMA Group (2009). Preferred Reporting Items for Systematic Reviews and Meta-Analyses: The PRISMA Statement. PLoS Med 6(7): e1000097. doi:10.1371/journal.pmed1000097

For more information, visit: **www.prisma-statement.org**.

For peer review only

For peer review only - http://bmjopen.bmj.com/site/about/guidelines.xhtml

BMJ Open

Association between vitamin D supplementation or level and susceptibility to COVID-19 infection including clinical course, morbidity and mortality outcomes? A systematic review.

Journal:	*BMJ Open*
Manuscript ID	bmjopen-2020-043737.R1
Article Type:	Original research
Date Submitted by the Author:	30-Oct-2020
Complete List of Authors:	Grove, Amy; Warwick Life Sciences, Division of Health Sciences, Warwick Medical School<br>Osokogu, Osemeke; University of Warwick, Warwick Medical School<br>Al-Khudairy, Lena; University of Warwick Warwick Medical School, Population, evidence and technology<br>Mehrabian, Amin; University of Warwick, Warwick Medical School; University of Medical Sciences, 2) Department of Pharmaceutical Nanotechnology<br>Zanganeh, Mandana; University of Warwick, Warwick Medical School<br>Brown, Anna; University of Warwick, Warwick Medical School<br>Court, Rachel; University of Warwick, Health Sciences<br>Taylor-Phillips, Sian; University of Warwick, Warwick Medical School<br>Uthman, Olalekan; University of Warwick, Warwick-Centre for Applied Health Research (WCAHRD)<br>McCarthy, Noel; University of Warwick, Warwick Medical School<br>Kumar, Sudhesh; University of Warwick, Medical School<br>Clarke, Aileen; University of Warwick, Division of Health Sciences
**Primary Subject Heading**:	Public health
Secondary Subject Heading:	Infectious diseases
Keywords:	COVID-19, Public health < INFECTIOUS DISEASES, NUTRITION & DIETETICS

SCHOLARONE™ Manuscripts

I, the Submitting Author has the right to grant and does grant on behalf of all authors of the Work (as defined in the below author licence), an exclusive licence and/or a non-exclusive licence for contributions from authors who are: i) UK Crown employees; ii) where BMJ has agreed a CC-BY licence shall apply, and/or iii) in accordance with the terms applicable for US Federal Government officers or employees acting as part of their official duties; on a worldwide, perpetual, irrevocable, royalty-free basis to BMJ Publishing Group Ltd ("BMJ") its licensees and where the relevant Journal is co-owned by BMJ to the co-owners of the Journal, to publish the Work in this journal and any other BMJ products and to exploit all rights, as set out in our licence.

The Submitting Author accepts and understands that any supply made under these terms is made by BMJ to the Submitting Author unless you are acting as an employee on behalf of your employer or a postgraduate student of an affiliated institution which is paying any applicable article publishing charge ("APC") for Open Access articles. Where the Submitting Author wishes to make the Work available on an Open Access basis (and intends to pay the relevant APC), the terms of reuse of such Open Access shall be governed by a Creative Commons licence – details of these licences and which Creative Commons licence will apply to this Work are set out in our licence referred to above.

Other than as permitted in any relevant BMJ Author's Self Archiving Policies, I confirm this Work has not been accepted for publication elsewhere, is not being considered for publication elsewhere and does not duplicate material already published. I confirm all authors consent to publication of this Work and authorise the granting of this licence.

For peer review only

Title page

Title

Association between vitamin D supplementation or level and susceptibility to COVID-19 infection including clinical course, morbidity and mortality outcomes? A systematic review.

Author's names

Amy Grove (0000-0002-8027-7274), Osemeke Osokogu, Lena Al-Khudairy, Amin Mehrabian, Mandana Zanganeh, Anna Brown, Rachel Court, Sian Taylor-Phillips, Olalekan Uthman, Noel McCarthy, Sudhesh Kumar, Aileen Clarke (0000-0001-8299-3146).[1]

Address for each author
Author's names and positions

1) Warwick Medical School, University of Warwick, Coventry, CV4 7AL, UK

Dr Amy Grove
Associate Professor of Health Technology Assessment and Implementation Science

Dr Osemeke Osokogu
Research Fellow

Dr Lena Al-Khudairy
Senior Research Fellow

Dr Mandana Zanganeh
Research Fellow

Mrs Anna Brown
Information Specialist

Mrs Rachel Court
Information Specialist

Professor Sian Taylor-Phillips
Professor of Population Health

Professor Olalekan Uthman
Professor of Evidence Synthesis

For peer review only - http://bmjopen.bmj.com/site/about/guidelines.xhtml

Professor Noel McCarthy
Professor of Public Health

Professor Sudhesh Kumar
Dean of Medicine

Professor Aileen Clarke
Professor of Public Health and Health Services Research

2) Department of Pharmaceutical Nanotechnology, School of Pharmacy, Mashhad University of Medical Sciences, Mashhad, Iran

Mr Amin Mehrabian
Research Fellow

Corresponding Author

Dr Amy Grove, Room A101, Division of Health Sciences, Warwick Medical School, University of Warwick, Coventry, CV4 7AL
Email: A.L.Grove@warwick.ac.uk

For peer review only

Abstract

Objective: To systemically review and critically appraise published studies of the association between vitamin D supplementation or level and susceptibility to COVID-19 infection, including clinical course, morbidity and mortality outcomes.

Design: Systematic review.

Data sources: MEDLINE (OVID), Embase (OVID), Cochrane Central Register of Controlled Trials, MedRxiv and BioRxiv preprint databases. COVID-19 databases of the WHO, Cochrane, CEBM Oxford, and Bern University up to 10 June 2020.

Study selection: Studies which assessed Vitamin D supplementation and/or Low Serum Vitamin D in patients acutely ill with, or at risk of severe betacoronavirus infection (SARS-CoV, MERS-CoV, SARS-CoV-2).

Data extraction: Two authors independently extracted data using a predefined data extraction form and assessed risk of bias using the Downs and Black Quality Assessment Checklist.

Results: Searches elicited 449 papers, 59 studies were eligible full text assessment and four met the eligibility criteria of this review. The four studies were narratively synthesised and included: 1) a cross-sectional study (n=107) suggesting an inverse association between serum vitamin D and SARS-CoV-2, 2) a retrospective cohort study (348,598 participants, 449 cases) in which univariable analysis showed that vitamin D protects against COVID-19, 3) an ecological country level study demonstrating a negative correlation between vitamin D and COVID-19 case numbers and mortality, and 4) a case-control survey (n=1,486) showing cases with confirmed/probable COVID-19 reported lower vitamin D supplementation . All studies were at high/unclear risk of bias.

Conclusion: There is no robust evidence of a negative association between vitamin D and COVID-19 infection. No relevant randomised control trials were identified and there is no robust peer reviewed published evidence of association between Vitamin D levels and severity of symptoms or mortality due to COVID-19. Guideline producers should acknowledge that benefits of vitamin D supplementation in COVID-19 infection are as yet unproven despite increasing interest from the media and academic community.

For peer review only

Strengths and limitations of this study

- The strengths of this systematic review include that it is reported in accordance with the Preferred Reporting Items for Systematic Reviews and Meta-Analyses (PRISMA) checklist.
- The review was conducted by two independent reviewers to ensure robustness of this work.
- We searched multiple living systematic review databases to enable us to capture publications in a fast moving field of research.
- To increase transparency we have provided the details of our full search stratergy in the supplement information.
- The limitations of the study relate to the small amount of evidence available which was, at risk of bias and which limits the inferences that can be drawn.
- The review was restricted to the English language, therefore non English language papers may have been missed.

For peer review only

For peer review only - http://bmjopen.bmj.com/site/about/guidelines.xhtml

Introduction

COVID-19, a novel viral infection caused by Severe Acute Respiratory Syndrome Coronavirus two (SARS-CoV-2) was declared a pandemic by The World Health Organization (WHO) on 11 March 2020.[1] Mild COVID-19 infection may manifest as high temperature, a continuous cough and a loss of or change in sense of smell or taste.[2 3] However, more severe and critical cases can result in inflammation of the lungs, low oxygen levels and acute respiratory distress syndrome.[4] Interest is mounting regarding the association of vitamin D supplementation or level with susceptibility to COVID-19 infection due to the recognised modulating effects of vitamin D on the immune system and immune response.

Vitamin D can modulate the immune system through highly expressed receptors in most non-skeletal tissues.[5 6] Two of the most common analogues of vitamin D which are found in food and used as a dietary supplement are D_2 (ergocalciferol) and D_3 (cholecalciferol, also made by the skin when exposed to sunlight).[7] Both D_2 and D_3 can be hydroxylated by liver enzymes CYP2R1 and CYP27A1 to form calcidiol (25(OH)D). The active metabolite of vitamin D, calcitriol (1α,25(OH)2D), results from the action of CYP27B enzyme on calcidiol. CYP27B is found in several tissues including the kidney, skin, bones, and immune system.[8 9] Tumour necrosis factor α (TNF α) and interferon (IFNγ) are examples of inflammatory cytokines that stimulate the CYP27B enzymes of the immune system.[10-20] Vitamin D can interact with both the innate and cellular immune systems through these mechanisms.

Current Public Health England (PHE),[21] National Institutes of Health[22] and European Food Safety Authority[23] recommendations highlight the importance of vitamin D to population health. Vitamin D deficiency is defined as less than 25 nmol/L (10ng/ml) measured in blood serum.[21] UK guideline recommendations suggest that people take a supplement of 10 micrograms of vitamin D per day during the winter months or throughout the year if they do not spend time outdoors or if they cover the majority of their skin when outside.[21] Published editorials, journal commentaries [24-29] and news media reports [30-32] suggest that individuals with low blood serum concentrations of vitamin D might be at higher risk of infection with COVID-19, or upon infection have worse outcomes than individuals with normal/high serum vitamin D.[33]

Several observational studies have reported associations between low serum vitamin D and chronic[34] and acute conditions such as susceptibility to acute respiratory tract infections (RTI).[35-37] Most recently, Martineau and colleagues (2017) conducted a systematic review and meta-analysis of individual participant data from randomised controlled trials (RCTs) to assess the overall effect of vitamin D supplementation on risk of acute RTI.[38] They reported vitamin D supplementation to be safe while protecting against acute RTI overall (adjusted odds ratio 0.88, 95% confidence interval 0.81 to 0.96; P for heterogeneity <0.001). Patients very deficient in vitamin D benefited the most (adjusted odds ratio 0.75, 0.60 to 0.95; p for

For peer review only - http://bmjopen.bmj.com/site/about/guidelines.xhtml

interaction=0.006).[38] Critiques of this review have suggested that the findings should be interpreted as hypothesis generating only, as the results are heterogeneous and not sufficiently applicable to the general population.[39] Recent rapid reviews of vitamin D for treatment or prevention in COVID-19 reported no evidence that vitamin D deficiency predisposes to COVID-19, or that vitamin D supplementation is effective in prevention or treatment of COVID-19.[40 41] However, data sources included in the rapid review were limited.[42] Given the remaining uncertainty, it is timely to systematically review and critically appraise all peer reviewed published evidence to assess the association of vitamin D supplementation or level with susceptibility to COVID-19 infection including clinical course, morbidity and mortality outcomes.

Methods

Protocol registration

The methods were prespecified in a protocol that was registered with the PROSPERO International Prospective Register of Systematic Reviews (https://www.crd.york.ac.uk/prospero/display_record.php?ID=CRD42020182876). Research ethics committee approval was not required for this study.

We undertook a systematic review to answer the following question: Is vitamin D supplementation or level associated with susceptibility to severe betacoronavirus infection (Severe Acute Respiratory Syndrome [SARS-CoV], Middle East Respiratory Syndrome [MERSCoV], Severe Acute Respiratory Syndrome two [SARS-CoV-2]) including clinical course, morbidity and mortality outcomes?

Our review was conceptualised and written in accordance with the PRISMA statement.[43]

Data sources and search

The search strategy was developed by the information specialists in collaboration with the research team and clinical advisors. We searched MEDLINE (OVID interface), Embase (OVID interface), Cochrane Central Register of Controlled Trials, MedRxiv and BioRxiv preprint databases on 6th-8th May 2020. We searched the global research on COVID-19 developed by the WHO,[44] CEBM Oxford,[45] and the living systematic review developed by Bern University[46] on 10 May 2020. We updated the database searches on 10th June 2020 to capture articles which may have been published since the initial search was conducted.

We searched additional resources including relevant systematic reviews (in MEDLINE [OVID interface], Embase [OVID interface] and Cochrane Database of Systematic Reviews, 19th May 2020), relavent refrences and contacted experts for additional evidence. Our full search record is included in the supplementary information.

For peer review only - http://bmjopen.bmj.com/site/about/guidelines.xhtml
For peer review only

Study eligibility

We developed pre-defined study eligibility criteria aligned to the research question (Table 1). We imposed a date restriction of January 2002, to capture all published articles since SARS-CoV was first discovered in Asia in February 2003.[47] We limited to English language only.

Table 1. Study eligibility criteria

Study eligibility criteria
P - Population<br><br>1) Patients acutely ill with betacoronavirus infection [SARS-CoV, MERS-CoV, SARS-CoV-2]<br>2) or at risk of acute illness with betacoronavirus infection<br><br>I - Intervention/exposure<br><br>1) Vitamin D supplementation<br>2) Low Serum vitamin D<br><br>O - Outcomes<br><br>1) Betacoronavirus infection (to include serological evidence of infection or clinically confirmed symptomatic infection);<br>2) Severe betacoronavirus infection (to include patients admitted to hospital or admitted to intensive care); mortality due to betacoronavirus infection<br>3) Mortality due to betacoronavirus infection<br><br>C – Comparators<br><br>1) No vitamin D supplementation<br>2) High or normal serum vitamin D<br><br>S - Study design<br><br>Peer reviewed publications of randomised controlled trials and non-randomised studies were eligible for inclusion; including, non randomised controlled trials, interrupted time series analyses, controlled before-and-after studies, cohort studies, ecological studies, case reports and case series.<br><br>Subgroups<br><br>1. Ethnicity characteristics (White British, all other White, Mixed, Asian, Black, Other)<br>2. Age characteristics (population by five-year age groups)

For peer review only

Article selection

Following the article search, we systematically identified and removed any duplicate citations using EndNote X9 software. Using titles and abstracts, de-duplicated citations were screened by two independent reviewers (OO, MZ, AM, AG) and checked by a third (AC). All articles deemed ineligible were excluded at this stage. We identified and obtained all remaining articles for full text screening, which was performed independently by at least two reviewers against the pre-specified eligibility criteria (Table 1). Where disagreements regarding the inclusion of articles arose, a third reviewer (AC) was consulted to reach a final decision.

For peer review only - http://bmjopen.bmj.com/site/about/guidelines.xhtml

Data extraction

Two reviewers independently (LAK, MZ, OO, AM) extracted data from eligible full-text papers using a prespecified data extraction form. The accuracy of all the data extraction was independently assessed by a third reviewer (AG). Where reported, we sought to extract data from each article relevant to the research question, including details of population, intervention/exposure, comparator, outcomes and any detail related to the two pre-specified subgroups: ethnicity characteristics and age characteristics. Disagreements between reviewers were resolved by discussion and agreement, or via consultation with a third reviewer (AC).

Risk of bias

The included studies had observational study designs aimed at answering a specific question. Therefore, risk of bias of included full-text papers was assessed using the Downs and Black Quality Assessment Checklist.[48] Two reviewers (AM, MZ, OO) independently assessed the risk of bias of the included studies and the accuracy of the assessment was evaluated by a third reviewer (LAK).

Data analysis

We anticipated that identified studies would be too heterogeneous to facilitate pooling of study data and planned a narrative synthesis. Nevertheless, we intended to consider pooling outcomes data in a meta-analysis using a random-effects model if appropriate.

Patient and public involvement

Due to the rapid timeframe of this systematic review it was not possible for our research team to involve patients or the public in the design, conduct, or reporting of our study.

Results

After searching databases, assessing the reference lists of 17 narrative reviews[27 28 33 49-62] and one additional article identified through consultation with clinical experts,[38] we identified 499 citations. Following removal of duplicates and screening of titles and abstracts, we retrieved 59 full-text papers of which four met the full eligibility criteria (see **Error! Reference source not found.**). The electronic supplement includes a list of reasons for excluding studies at full text review. Seven articles closely met the eligibility criteria but were excluded as they were not available as peer reviewed publications at the time of our narrative synthesis, details of these seven studies[63-69] is provided in the electronic supplement.

<Figure 1 approximatley here >

For peer review only - http://bmjopen.bmj.com/site/about/guidelines.xhtml

The charteristics of the four included studies are presented in

For peer review only

For peer review only - http://bmjopen.bmj.com/site/about/guidelines.xhtml

Table 2. All four included studies were conducted in Europe and published in April or May 2020. One study was based on data from UK residents exclusively,[70] another included data on residents in 20 European countries, including the UK.[71] The studies were observational design and no relevant RCT were identified or included in the review. All four studies were at high or unclear risk of bias and scored poorly across several domains of the Downs and Black Quality Assessment Checklist,[48] including external validity, internal validity and power. A prominent issue amongst the included studies was that the authors did not perfom adequate multivariable adjustment to correct for confounding.[72] [73] [74] Ecological bias was present in Ilie et al., 2020[71] which may result from spatial and temporal scale differences between country level mean levels of vitamin D. However, several domains in each risk of bias assessment were not applicable or not reported and therefore, could not be scored using the Downs and Black Quality Assessment Checklist.[48] Detailed risk of bias scores are provided in the electronic supplement.

For peer review only

For peer review only - http://bmjopen.bmj.com/site/about/guidelines.xhtml

Table 2. Characteristics of the four included studies

Study	Design/Setting	Population	Exposure/Intervention	Outcomes	Results	Limitations
**Serum vitamin D**						
D'Avolio et al. 2020[73]	Cross-sectional study<br><br>Canton of Tessin, Switzerland	107 patients with data on SARS-CoV-2 and 25(OH)D measurement	Vitamin D analysis, conducted within seven weeks of the SARS-CoV-2 polymerase chain reaction (PCR) result<br><br>Control patients with 25(OH)D data during the same period	SARS-CoV-2 infection	Group 1 comprised 27 patients with positive PCR test results for SARS-CoV-2 while group 2 comprised 80 patients with a negative PCR result for SARS-CoV-2<br><br>Significantly lower 25(OH)D levels (p = 0.004) in SARS-CoV-2 patients even after stratifying patients according to age >70 years	Few patients from a single hospital<br><br>No available clinical information about the severity of COVID-19 symptoms<br><br>No data on other potential confounding variable<br><br>SARS-CoV-2 and the 25(OH)D status were performed on different days
Hastie et al. 2020[70]	Retrospective cohort study<br><br>UK Biobank Cohort including England, Scotland and Wales	502,624 participants aged 37-73 years between 2006 and 2010	Biochemical assay of 25(OH)D, a measure of vitamin D status<br><br>Vitamin D was imputed if it was below or above the limit of detection	Confirmed COVID-19 infection (at least one positive test result)	Complete data on 348,598 UK Biobank participants<br><br>449 had confirmed COVID-19 infection. Of these, 385 (85.8%) were White compared to 64 (14.2%) non-White (Black, South Asian and others)<br><br>Vitamin D was associated with COVID-19 infection univariably but not after adjustment for confounders. Ethnicity was associated with COVID-19 infection	UK Biobank is not representative of the general population<br><br>Baseline measurements, including 25(OH)D concentration and health status, were obtained a decade prior to conduct of the study

For peer review only - http://bmjopen.bmj.com/site/about/guidelines.xhtml

Ilie et al. 2020[71]	Ecological study<br><br>20 European countries	Population of 20 included European countries	Mean levels of vitamin D in each country	Cases of COVID-19 per 1 million population in each country<br><br>Deaths from COVID-19 per 1 million population	Negative correlations between mean levels of vitamin D and the number of COVID-19 cases per 1 million, and mortality per 1 million	The number of cases per country is affected by the number of tests performed and by the different measures taken by each country to prevent the spread of infection
**Vitamin D supplementation**						
Fasano et al. 2020[74]	Case-control survey<br><br>A single tertiary centre in Lombardy, Italy	1,486 Parkinson's disease (PD) patients were included in the survey<br><br>1,207 family members (controls)	Vitamin D	'Confirmed' or 'probable' diagnosis of COVID-19	12.4% of PD patients with confirmed or probable COVID-19 had been taking vitamin D<br><br>22.9% of PD patients without COVID-19 had been taking vitamin D	Well-known limitation of a telephone survey<br><br>Community-dwelling PD patients<br><br>Some patients could not be reached possibly due to death from COVID-19<br><br>COVID-19 diagnosis could not be confirmed in many cases<br><br>Younger age of non- PD COVID-19 cases

For peer review only

Serum vitamin D

D'Avolio et al., 2020[73] used a cross sectional design with data on nasopharyngeal swab polymerase chain reaction (PCR) analysis for SARS-CoV-2 and a 25(OH)D measurement taken from patients between 1st March and 14th April 2020. PCR positives (median age = 74 years [IQR 65–81];male = 70.4%) had significantly (P= 0.004) lower serum 25(OH)D levels (median = 11.1 ng/mL [IQR 8.2–21.0]) than PCR negatives (median age = 73 years [IQR 61–82]; male = 48.8%; median 25(OH)D = 24.6 ng/mL [IQR 8.9–30.5]). Although gender and age stratified analysis showed no significant differences, older (>70 years) SARS-CoV-2 positive (n=18) participants had significantly lower median serum 25 (0H) D levels (9.3 ng/mL [IQR 8.1–19.9] than older SARS-CoV-2 negatives (n=43) (23.1 ng/mL [IQR 8.5–31.7]) (P = 0.037).

Hastie et al., 2020[70] is a retrospective cohort study that utilised data from the UK Biobank, [72] using data from 348,598 people with complete information on vitamin D and covariates; 449 people tested positive for COVID-19 infection. COVID-19 positives were older (Median = 49 years; Interquartile Range [IQR] = 40-58) than COVID-19 negatives (Median = 49 years; IQR = 38 - 57) with p-value of <0.05. Multivariable analysis showed that age at assessment (OR = 1.02; 95% CI = 1.00 - 1.03; P= 0.016) and non-White ethnicity (Black OR = 4.30, 95% CI = 2.92 - 6.31, P= < 0.001; South Asian OR = 2.42, 95% CI = 1.50 - 3.93, P= <0.001) were associated with confirmed COVID-19 infection. There was no significant interaction between ethnicity and vitamin D deficiency (OR = 0.90; 95% CI = 0.66 - 1.23; P= 0.515). Median vitamin D concentration at recruitment was lower for people with subsequent confirmed COVID-19 infection (28.7 [IQR 10.0-43.8] nmol/l) than for other participants (32.7 [IQR 10.0-47.2] nmol/l) (P= <0.01). Although univariable analysis suggested an association between vitamin D and COVID-19 (OR=0.99; 95% CI 0.99 – 0.999; P=0.013), this association became insignificant (OR=1.00; 95% CI = 0.998-1.01; P=0.208) after adjustment for covariates.[70]

Ilie et al., 2020[71] used an ecological study design reporting on 20 European countries as at 8th April 2020; the data pertains to mean levels of vitamin D, cases of COVID-19 infection per million population and deaths from COVID-19 per million population. The authors performed Pearson Correlation Coefficient Calculations and reported a negative correlation between mean levels of vitamin D (Mean 56.79 nmol/l, SD 10.61) and numbers of cases of COVID-19 infection per million population in each country (Mean cases 1393.4, SD 1129.984, $r(20) = -0.44$; P = 0.05). Additionally, a negative correlation was reported between mean vitamin D levels and the number of deaths caused by COVID-19 per million population in each country (Mean 80.42, SD 94.61, $r(20) = -0.4378$; P = 0.05). Sweden had the highest mean level of vitamin D (73.5 nmol/l) compared to Spain which had a mean level of 42.5 nmol/l). The number of cases of COVID-19 per million population was 834 in Sweden and 3,137 in Spain. Likewise, at the time of the study, there were 68 deaths from COVID-19 per million population in Sweden and 314 in Spain.

For peer review only - http://bmjopen.bmj.com/site/about/guidelines.xhtml

Vitamin D supplementation

Fasano et al., 2020[74] investigated patients in a case-control phone survey in Lombardy, Italy. COVID-19 diagnosis was confirmed using a nasopharyngeal swab or probable based on : a) presence of persistent COVID-19-related symptoms (≥3 including fever or ≥5 without fever); or b) ≥1 symptom in presence of suggestive chest radiologic signs; and/or c) living with a family member with a confirmed diagnosis of COVID-19. 1,486 participants were included in the survey (32 confirmed COVID-19, 73 probable COVID-19 and 1,381 unaffected). Confirmed/probable COVID-19 cases (mean age = 70.5 [Standard Deviation [SD] = 10.1]; male = 53%) self- reported a significantly lower intake of vitamin D supplementation (12.4%) compared to unaffected cases (22.9%; mean age = 73.0 [SD = 9.5], male = 57%). The age-adjusted OR (OR 0.56 [95% CI = 0.32-0.99], P= 0.048) suggested a protective effect of vitamin D intake.

Subgroup evaluation

We planned to perform subgroup analyses by age and ethnicity. According to Hastie et al., 2020[70] multivariable analysis showed that age at assessment (OR = 1.02; 95% CI = 1.00 - 1.03; P= 0.016) and non-White ethnicity (Black OR = 4.30, 95% CI = 2.92 - 6.31, P= < 0.001; South Asians OR = 2.42, 95% CI = 1.50 - 3.93, P= <0.001) were associated with confirmed COVID-19 infection. However, Hastie et al found no significant interaction between ethnicity and vitamin D deficiency (OR = 0.90; 95% CI = 0.66 - 1.23; P= 0.515).

Discussion

This systematic review of non-randomised studies has shown no robust evidence of a association between vitamin D and COVID-19 infection. We identified four studies for inclusion in a narrative synthesis which were all at high or unclear risk of bias. A univariable analysis of data from the UK Biobank database revealed an association between vitamin D and COVID-19 infection (OR=0.99; 95% CI 0.99 – 0.999; P=0.013). However, this association became insignificant (OR=1.00; 95% CI=0.998-1.01; P=0.208) after adjustment for 13 other covariates, suggesting that the initial association was due to one or more confounding variables.[70]This view is further strengthened by the demonstration of highly significant associations between age and ethnicity characteristics as predictor variables, and COVID-19 infection as the outcome variable. Overall, the UK Biobank study showed no effect, however it should be noted that the UK Biobank data included only one measurement of Vitamin D levels taken between 10 and 14 years prior to the outbreak of COVID-19. This is a significant study limitation.

Liu et al., 2020[75] concluded that patients over 60 years experienced more severe manifestations and had longer disease courses of COVID-19 compared to patients below 60 years.[75] And other studies have shown that older (rather than younger) people are more likely to die from COVID-19 infection.[76-79]

For peer review only - http://bmjopen.bmj.com/site/about/guidelines.xhtml

Non-White people are known to be more susceptible to COVID-19 infection and tend to develop worse outcomes,[80] a finding that our review has further substantiated.[70] Ethnicity is a multi-faceted construct that includes genetic make-up, socio-cultural identity and behavioural patterns.[81] It has been shown to be associated with differing susceptibility and treatment outcomes in a number of diseases.[82 83 84] Hastie et al., 2020[#ref} did not find any interaction between ethnicity and vitamin D deficiency and although Ilie et al 2020[71] identified a relationship, the study is subject to ecological bias. Ilie et al 2020[71] compared vitamin D levels and rates of COVID-19 infection aross 20 European countries, and therefore many relevant factors were not accounted for in the analysis. Given the findings so far from our review we consider that there is paucity of data on vitamin D levels and morbidity and mortality from COVID-19 and there is no evidence from RCTs on outcomes of vitamin D supplementation on severity of symptoms or mortality to date. However a relationship between ethnicity, vitamin D (serum levels or supplementation) and susceptibility to or severity of COVID-19 infection cannot yet be ruled out.

Risk of bias assessments demonstrate that all studies were at high or unclear risk of bias. All studies were observational designs and therefore subject to confounding. The persistent calls for high-dose vitamin D supplementation[85] arise from speculation about presumed mechanisms.[86 87] Our systematic review found no robust evidence that low levels of Vitamin D are associated with an increased likelihood of COVID-19 infection. More robust prognostic studies could be combined in a systematic review where a prognostic factor research question is phrased, and considerations of participation, attrition, prognostic factor measurement, confounding measurement and account, outcome measurement, and analysis and reporting are evaluated.

Our systematic review identified no relevant RCTs, nevertheless we are aware of two ongoing RCTs investigating the effects of vitamin D on COVID-19, the ZnD3-CoVici study, France (NCT04351490)[88] and the CoVitTrial, France (NCT04344041).[89] Both trials have an estimated study completion date of July 2020. Inclusion of data from these studies in future systematic reviews and meta-analyses may enable us to potentially draw better stronger conclusions on this topic. Results from the ongoing international VITDALIZE Study (NCT03188796) may also contribute to our understanding of the effect of high dose vitamin D3 on mortality.[90]

Study limitations

We performed a full systematic review of the published evidence available, and simultaneous independent screening, data extraction and risk of bias assessments. However, our study is limited by the small amount of evidence available which was, moreover, at risk of bias. This limits the inferences that can be drawn. Seven eligible studies were excluded because they are not available as peer reviewed publications.[63-69] If published, these seven studies would be included in a future update of this review. A final limitation is that the review was restricted

For peer review only - http://bmjopen.bmj.com/site/about/guidelines.xhtml

to English language only. Therefore, articles published in other languages may have been excluded.

Implications for practice

Our review does not provide evidence for or against additional or high dose vitamin D supplementation specifically in relation to COVID-19. Treatment as standard practice for people who are deficient is pre-existing practice across Europe[23] the US[91] and in the UK.[21] Current guidelines from PHE suggest that the entire UK population should take vitamin D supplements to prevent vitamin D deficiency in winter or with inadequate sunlight exposure to sun in summer.[21] This review does not give evidence to drive a change in this current advice. Treatment recommendations for patients should be updated following the publication of results from ongoing and new well designed adequately powered randomised controlled trials

Conclusion

This systematic review identified no robust evidence to enable us to assess an association between vitamin D supplementation or level with susceptibility to COVID-19 infection including clinical course, morbidity and mortality outcomes. All studies were at high or unclear risk of bias. Both age and ethnicity were associated with vitamin D levels even after multivariable adjustment. Black and South Asian people had a much higher risk of confirmed COVID-19 compared to White people. However, there was no interaction between the association of ethnicity and vitamin D deficiency with COVID-19. There were no papers reporting association of vitamin D with severity of symptoms or mortality due to COVID-19.

For peer review only

For peer review only - http://bmjopen.bmj.com/site/about/guidelines.xhtml

Acknowledgements:

We thank Mrs Kath Charters, Welsh Ambulance Services NHS Trust for comments on the study design and drafts of this manuscript and for valuable advice and helpful discussions.

Contributorship statement:

SK, AG and AC conceived the study. AG, AC, NMcC, SK, STP and OU designed the study. RC and AB developed the search stratergies, performed all searches and database management and created the bibliography. AG, AC, AM, OO, MZ screened titles and abstracts for inclusion. AG, OO, AM, MZ, LAK, AC screened at full text and extracted and analysed data. OO, AM, MZ, LAK performed risk of bias assessments. AC, SK and NMcC assisted in the interpretation from a clinical perspective. STP, LAK, OU offered technical and methodological support. AG and OO wrote the first draft, all authors revised content. All authors approved the final manuscript. AG and AC are the guarantors. The corresponding author attests that all listed authors meet authorship criteria and that no others meeting the criteria have been omitted.

Copyright/license for publication:

The Corresponding Author has the right to grant on behalf of all authors and does grant on behalf of all authors, a worldwide licence to the Publishers and its licensees in perpetuity, in all forms, formats and media (whether known now or created in the future), to i) publish, reproduce, distribute, display and store the Contribution, ii) translate the Contribution into other languages, create adaptations, reprints, include within collections and create summaries, extracts and/or, abstracts of the Contribution, iii) create any other derivative work(s) based on the Contribution, iv) to exploit all subsidiary rights in the Contribution, v) the inclusion of electronic links from the Contribution to third party material where-ever it may be located; and, vi) licence any third party to do any or all of the above.

Funding:

Dr Amy Grove is supported by the National Institute for Health Research (NIHR) Advanced Fellowship Programme Reference (AF-300060). Dr Taylor-Phillips is supported by an NIHR Career Development Fellowship (CDF-2016-09-018). Prof Clarke and Dr Al-Khudairy are supported by the NIHR Applied Research Centre West Midlands (ARC-WM). All other authors, with the exception of Professor Uthman, Professor Kumar and Professor McCarthy, are supported by the NIHR, Evidence Synthesis Programme (HTA 14.25.05). The views expressed are those of the authors and not necessarily those of the NIHR or the Department of Health and Social Care.

Competing interests:

All authors have completed the ICMJE uniform disclosure form at www.icmje.org/coi_disclosure.pdf and declare: no support from any organisation for the submitted work; no competing interests with regards to the submitted work.

For peer review only - http://bmjopen.bmj.com/site/about/guidelines.xhtml

Ethical statement:

Not required

Data sharing statement:

The study protocol is available

Systematic review protocol registration: CRD42020182876 available online via PROSPERO at https://www.crd.york.ac.uk/prospero/display_record.php?ID=CRD42020182876. All included studies are publicly available. Additional data are available upon reasonable request by emailing the corresponding author.

For peer review only

For peer review only - http://bmjopen.bmj.com/site/about/guidelines.xhtml

References

1. World Health Organization. Coronavirus disease (COVID-19) pandemic. 2020. https://www.who.int/emergencies/diseases/novel-coronavirus-2019
2. NHS. Check if you or your child has coronavirus symptoms. 2020. https://www.nhs.uk/conditions/coronavirus-covid-19/symptoms/#symptoms
3. Argenziano MG, Bruce SL, Slater CL, et al. Characterization and clinical course of 1000 patients with coronavirus disease 2019 in New York: retrospective case series. *BMJ* 2020;369:m1996. doi: 10.1136/bmj.m1996
4. Matthay MA, Aldrich JM, Gotts JE. Treatment for severe acute respiratory distress syndrome from COVID-19. *Lancet Respir Med* 2020;8(5):433-34. doi: 10.1016/s2213-2600(20)30127-2
5. Gruber BM. Fenomen witaminy D [The phenomenon of vitamin D]. *Postepy Hig Med Dosw (Online)* 2015;69:127-39
6. Gruber-Bzura BM. Vitamin D and influenza: prevention or therapy? *Int J Mol Sci* 2018;19(8):2419. doi: 10.3390/ijms19082419
7. Holick MF. High prevalence of vitamin D inadequacy and implications for health. *Mayo Clin Proc* 2006;81(3):353-73. doi: 10.4065/81.3.353
8. Sigmundsdottir H, Pan J, Debes GF, et al. DCs metabolize sunlight-induced vitamin D3 to 'program' T cell attraction to the epidermal chemokine CCL27. *Nat Immunol* 2007;8(3):285-93. doi: 10.1038/ni1433
9. Kogawa M, Findlay DM, Anderson PH, et al. Osteoclastic metabolism of 25(OH)-vitamin D3: a potential mechanism for optimization of bone resorption. *Endocrinology* 2010;151(10):4613-25. doi: 10.1210/en.2010-0334
10. Jones G. Vitamin D safety: its mechanisms and application. *Standardy Medyczne, Pediatria* 2012;9:605-09
11. Kim D. The role of vitamin D in thyroid diseases. *Int J Mol Sci* 2017;18(9):1949. doi: 10.3390/ijms18091949
12. Beard JA, Bearden A, Striker R. Vitamin D and the anti-viral state. *J Clin Virol* 2011;50(3):194-200. doi: 10.1016/j.jcv.2010.12.006
13. Liu PT, Stenger S, Li H, et al. Toll-like receptor triggering of a vitamin D-mediated human antimicrobial response. *Science* 2006;311(5768):1770-3. doi: 10.1126/science.1123933
14. Adams JS, Ren S, Liu PT, et al. Vitamin D-directed rheostatic regulation of monocyte antibacterial responses. *J Immunol* 2009;182(7):4289-95. doi: 10.4049/jimmunol.0803736
15. Laaksi I. Vitamin D and respiratory infection in adults. *Proc Nutr Soc* 2012;71(1):90-7. doi: 10.1017/s0029665111003351
16. Sharifi A, Vahedi H, Nedjat S, Rafiei H, Hosseinzadeh-Attar MJ. Effect of single-dose injection of vitamin D on immune cytokines in ulcerative colitis patients: a randomized placebo-controlled trial. *APMIS* 2019;127(10):681-87. doi: 10.1111/apm.12982
17. Cantorna MT, Snyder L, Lin YD, Yang L. Vitamin D and 1,25(OH)2D regulation of T cells. *Nutrients* 2015;7(4):3011-21. doi: 10.3390/nu7043011
18. Jeffery LE, Burke F, Mura M, et al. 1,25-Dihydroxyvitamin D3 and IL-2 combine to inhibit T cell production of inflammatory cytokines and promote development of regulatory

For peer review only

T cells expressing CTLA-4 and FoxP3. *J Immunol* 2009;183(9):5458-67. doi: 10.4049/jimmunol.0803217
19. Lei GS, Zhang C, Cheng BH, Lee CH. Mechanisms of action of vitamin D as supplemental therapy for pneumocystis pneumonia. *Antimicrob Agents Chemother* 2017;61(10):e01226-17. doi: 10.1128/aac.01226-17
20. Colunga Biancatelli RML, Berrill M, Marik PE. The antiviral properties of vitamin C. *Expert Rev Anti Infect Ther* 2020;18(2):99-101. doi: 10.1080/14787210.2020.1706483
21. Scientific Advisory Committee on Nutrition. SACN vitamin D and health report. 21 July 2016. https://www.gov.uk/government/publications/sacn-vitamin-d-and-health-report
22. National Institutes of Health, Office of Dietary Supplements. Vitamin D: fact sheet for health professionals. 24 March 2020. https://ods.od.nih.gov/factsheets/VitaminD-HealthProfessional/#h2
23. EFSA Panel on Dietetic Products, Nutrition and Allergies. Dietary reference values for vitamin D. *EFSA Journal* 2016;14(10):e04547. doi: 10.2903/j.efsa.2016.4547
24. Cao Z, Wu Y, Faucon E, Sabatier JM. SARS-CoV-2 & Covid-19: key-roles of the 'renin-angiotensin' system / vitamin D impacting drug and vaccine developments. *Infect Disord Drug Targets* 2020;20(1). doi: 10.2174/1871526520999200505174704
25. Jakovac H. COVID-19 and vitamin D: is there a link and an opportunity for intervention? *Am J Physiol Endocrinol Metab* 2020;318(5):E589. doi: 10.1152/ajpendo.00138.2020
26. Mitchell F. Vitamin-D and COVID-19: do deficient risk a poorer outcome? *Lancet Diabetes Endocrinol* 2020;8(7):570. doi: 10.1016/S2213-8587(20)30183-2
27. Silberstein M. Vitamin D: a simpler alternative to tocilizumab for trial in COVID-19? *Med Hypotheses* 2020;140:109767. doi: 10.1016/j.mehy.2020.109767
28. Calder PC, Carr AC, Gombart AF, Eggersdorfer M. Optimal nutritional status for a well-functioning immune system is an important factor to protect against viral infections. *Nutrients* 2020;12(4):1181. doi: https://dx.doi.org/10.3390/nu12041181
29. Tian Y, Rong L. Letter: Covid-19, and vitamin D. Authors' reply. *Aliment Pharmacol Ther* 2020;51(10):995-96. doi: 10.1111/apt.15764
30. Busby M. UK public health bodies reviewing vitamin D's effects on coronavirus. The Guardian; 17 June 2020. https://www.theguardian.com/world/2020/jun/17/uk-ministers-order-urgent-vitamin-d-coronavirus-review
31. Coronavirus: Should I start taking vitamin D? BBC News; 18 June 2020. https://www.bbc.co.uk/news/health-52371688
32. Torjesen I. Covid-19: Public health agencies review whether vitamin D supplements could reduce risk. *BMJ* 2020;369:m2475. doi: 10.1136/bmj.m2475
33. Grant WB, Lahore H, McDonnell SL, et al. Evidence that vitamin D supplementation could reduce risk of influenza and COVID-19 infections and deaths. *Nutrients* 2020;12(4):988. doi: 10.3390/nu12040988
34. Parker J, Hashmi O, Dutton D, et al. Levels of vitamin D and cardiometabolic disorders: systematic review and meta-analysis. *Maturitas* 2010;65(3):225-36. doi: 10.1016/j.maturitas.2009.12.013
35. Cannell JJ, Vieth R, Umhau JC, et al. Epidemic influenza and vitamin D. *Epidemiol Infect* 2006;134(6):1129-40. doi: 10.1017/s0950268806007175
36. Jolliffe DA, Griffiths CJ, Martineau AR. Vitamin D in the prevention of acute respiratory infection: systematic review of clinical studies. *J Steroid Biochem Mol Biol* 2013;136:321-9. doi: 10.1016/j.jsbmb.2012.11.017

For peer review only - http://bmjopen.bmj.com/site/about/guidelines.xhtml

37. Nnoaham KE, Clarke A. Low serum vitamin D levels and tuberculosis: a systematic review and meta-analysis. *Int J Epidemiol* 2008;37(1):113-19. doi: 10.1093/ije/dym247
38. Martineau AR, Jolliffe DA, Hooper RL, et al. Vitamin D supplementation to prevent acute respiratory tract infections: systematic review and meta-analysis of individual participant data. *BMJ* 2017;356:i6583. doi: 10.1136/bmj.i6583
39. Bolland MJ, Avenell A. Do Vitamin D supplements help prevent respiratory tract infections? *BMJ* 2017;356:j456. doi: 10.1136/bmj.j456
40. National Institute for Health and Care Excellence. COVID-19 rapid evidence summary: vitamin D for COVID-19. 29 June 2020. https://www.nice.org.uk/advice/es28/chapter/Key-messages
41. Royal Society. Vitamin D and covid-19. 18 June 2020. https://royalsociety.org/-/media/policy/projects/set-c/set-c-vitamin-d-and-covid-19.pdf
42. Lee J, van Hecke O, Roberts N, on behalf of the Oxford COVID-19 Evidence Service Team. Vitamin D: a rapid review of the evidence for treatment or prevention in COVID-19. Centre for Evidence-Based Medicine, University of Oxford; 1 May 2020. https://www.cebm.net/covid-19/vitamin-d-a-rapid-review-of-the-evidence-for-treatment-or-prevention-in-covid-19/
43. Moher D, Liberati A, Tetzlaff J, Altman DG. Preferred reporting items for systematic reviews and meta-analyses: the PRISMA statement. *BMJ* 2009;339:b2535. doi: 10.1136/bmj.b2535
44. World Health Organization. Global research on coronavirus disease (COVID-19) [online database]. 2020. https://www.who.int/emergencies/diseases/novel-coronavirus-2019/global-research-on-novel-coronavirus-2019-ncov
45. Oxford COVID-19 Evidence Service. Centre for Evidence-Based Medicine, University of Oxford; 2020. https://www.cebm.net/oxford-covid-19-evidence-service/
46. Counotte M, Imeri H, Ipekci M, Low N. Living evidence on COVID-19 [online database]. University of Bern Institute of Social and Preventive Medicine; 2020. https://zika.ispm.unibe.ch/assets/data/pub/ncov/
47. Centers for Disease Control and Prevention. CDC SARS response timeline. 2013. https://www.cdc.gov/about/history/sars/timeline.htm
48. Downs SH, Black N. The feasibility of creating a checklist for the assessment of the methodological quality both of randomised and non-randomised studies of health care interventions. *J Epidemiol Community Health* 1998;52(6):377-84. doi: 10.1136/jech.52.6.377
49. Carter SJ, Baranauskas MN, Fly AD. Considerations for obesity, vitamin D, and physical activity amidst the COVID-19 pandemic. *Obesity (Silver Spring)* 2020;28:1176-77. doi: 10.1002/oby.22838
50. Molloy EJ, Murphy N. Vitamin D, covid-19 and children. *Ir Med J* 2020;113(4):64
51. McCartney DM, Byrne DG. Optimisation of vitamin D status for enhanced immuno-protection against covid-19. *Ir Med J* 2020;113(4):P58
52. Adams KK, Baker WL, Sobieraj DM. Myth busters: dietary supplements and COVID-19. *Ann Pharmacother* 2020;54(8):820-26. doi: 10.1177/1060028020928052
53. de Lucena TMC, da Silva Santos AF, de Lima BR, de Albuquerque Borborema ME, de Azevedo Silva J. Mechanism of inflammatory response in associated comorbidities in COVID-19. *Diabetes Metab Syndr* 2020;14(4):597-600. doi: 10.1016/j.dsx.2020.05.025

For peer review only - http://bmjopen.bmj.com/site/about/guidelines.xhtml

54. Ghasemian R, Shamshirian A, Heydari K, et al. The role of vitamin D in the age of COVID-19: a systematic review and meta-analysis along with an ecological approach. *medRxiv* 2020. doi: 10.1101/2020.06.05.20123554
55. Hribar CA, Cobbold PH, Church FC. Potential role of vitamin D in the elderly to resist COVID-19 and to slow progression of Parkinson's disease. *Brain Sci* 2020;10(5):284. doi: 10.3390/brainsci10050284
56. Laird E, Rhodes J, Kenny RA. Vitamin D and inflammation: potential implications for severity of Covid-19. *Ir Med J* 2020;113(5):P81
57. McKenna MJ, Flynn MAT. Covid-19, cocooning and vitamin D intake requirements. *Ir Med J* 2020;113(5):P79
58. Ribeiro H, Santana KVS, Oliver SL, et al. Does vitamin D play a role in the management of Covid-19 in Brazil? *Rev Saude Publica* 2020;54:53
59. Speeckaert MM, Delanghe JR. Association between low vitamin D and COVID-19: don't forget the vitamin D binding protein. *Aging Clin Exp Res* 2020. doi: 10.1007/s40520-020-01607-y
60. Suresh PS. Hypovitaminosis D and COVID-19: matter of concern in India? *Indian J Clin Biochem* 2020. doi: 10.1007/s12291-020-00894-6
61. Tan SHS, Hong CC, Saha S, Murphy D, Hui JH. Medications in COVID-19 patients: summarizing the current literature from an orthopaedic perspective. *Int Orthop* 2020. doi: 10.1007/s00264-020-04643-5
62. Zabetakis I, Lordan R, Norton C, Tsoupras A. COVID-19: the inflammation link and the role of nutrition in potential mitigation. *Nutrients* 2020;12(5):1466. doi: 10.3390/nu12051466
63. Lau FH, Majumder R, Torabi R, et al. Vitamin D insufficiency is prevalent in severe COVID-19. *medRxiv* 2020. doi: 10.1101/2020.04.24.20075838
64. Darling AL, Ahmadi KR, Ward KA, et al. Vitamin D status, body mass index, ethnicity and COVID-19: initial analysis of the first-reported UK Biobank COVID-19 positive cases (n 580) compared with negative controls (n 723). *medRxiv* 2020. doi: 10.1101/2020.04.29.20084277
65. De Smet D, De Smet K, Herroelen P, Gryspeerdt S, Martens GA. Vitamin D deficiency as risk factor for severe COVID-19: a convergence of two pandemics. *medRxiv* 2020. doi: 10.1101/2020.05.01.20079376
66. Meltzer DO, Best TJ, Zhang H, Vokes T, Arora V, Solway J. Association of vitamin D deficiency and treatment with COVID-19 incidence. *medRxiv* 2020. doi: 10.1101/2020.05.08.20095893
67. Notari A, Torrieri G. COVID-19 transmission risk factors. *medRxiv* 2020. doi: 10.1101/2020.05.08.20095083
68. Raisi-Estabragh Z, McCracken C, Bethell MS, et al. Greater risk of severe COVID-19 in non-White ethnicities is not explained by cardiometabolic, socioeconomic, or behavioural factors, or by 25(OH)-vitamin D status: study of 1,326 cases from the UK Biobank. *medRxiv* 2020. doi: 10.1101/2020.06.01.20118943
69. Tan CW, Ho LP, Kalimuddin S, et al. A cohort study to evaluate the effect of combination vitamin D, magnesium and vitamin B12 (DMB) on progression to severe outcome in older COVID-19 patients. *medRxiv* 2020. doi: 10.1101/2020.06.01.20112334
70. Hastie CE, Mackay DF, Ho F, et al. Vitamin D concentrations and COVID-19 infection in UK Biobank. *Diabetes Metab Syndr* 2020;14(4):561-65. doi: 10.1016/j.dsx.2020.04.050

For peer review only - http://bmjopen.bmj.com/site/about/guidelines.xhtml

71. Ilie PC, Stefanescu S, Smith L. The role of vitamin D in the prevention of coronavirus disease 2019 infection and mortality. *Aging Clin Exp Res* 2020. doi: 10.1007/s40520-020-01570-8
72. UK Biobank. About UK Biobank. 17 June 2020. https://www.ukbiobank.ac.uk/about-biobank-uk/
73. D'Avolio A, Avataneo V, Manca A, et al. 25-hydroxyvitamin D concentrations are lower in patients with positive PCR for SARS-CoV-2. *Nutrients* 2020;12(5):1359. doi: 10.3390/nu12051359
74. Fasano A, Cereda E, Barichella M, et al. COVID-19 in Parkinson's Disease Patients Living in Lombardy, Italy. *Mov Disord* 2020. doi: 10.1002/mds.28176
75. Liu Y, Mao B, Liang S, et al. Association between age and clinical characteristics and outcomes of COVID-19. *Eur Respir J* 2020;55(5):2001112. doi: 10.1183/13993003.01112-2020
76. Verity R, Okell LC, Dorigatti I, et al. Estimates of the severity of coronavirus disease 2019: a model-based analysis. *Lancet Infect Dis* 2020;20(6):669-77. doi: 10.1016/s1473-3099(20)30243-7
77. Mahase E. Covid-19: death rate is 0.66% and increases with age, study estimates. *BMJ* 2020;369:m1327. doi: 10.1136/bmj.m1327
78. Fulzele S, Sahay B, Yusufu I, et al. COVID-19 virulence in aged patients might be impacted by the host cellular microRNAs abundance/profile. *Aging Dis* 2020;11(3):509-22. doi: 10.14336/ad.2020.0428
79. Koff WC, Williams MA. Covid-19 and immunity in aging populations: a new research agenda. *N Engl J Med* 2020. doi: 10.1056/NEJMp2006761
80. Pan D, Sze S, Minhas JS, et al. The impact of ethnicity on clinical outcomes in COVID-19: a systematic review. *EClinicalMedicine*. doi: 10.1016/j.eclinm.2020.100404
81. Lee C. "Race" and "ethnicity" in biomedical research: how do scientists construct and explain differences in health? *Soc Sci Med* 2009;68(6):1183-90. doi: 10.1016/j.socscimed.2008.12.036
82. Stead WW, Senner JW, Reddick WT, Lofgren JP. Racial differences in susceptibility to infection by Mycobacterium tuberculosis. *N Engl J Med* 1990;322(7):422-7. doi: 10.1056/nejm199002153220702
83. Nahid P, Horne DJ, Jarlsberg LG, et al. Racial differences in tuberculosis infection in United States communities: the coronary artery risk development in young adults study. *Clin Infect Dis* 2011;53(3):291-4. doi: 10.1093/cid/cir378
84. Bime C, Poongkunran C, Borgstrom M, et al. Racial differences in mortality from severe acute respiratory failure in the United States, 2008-2012. *Ann Am Thorac Soc* 2016;13(12):2184-89. doi: 10.1513/AnnalsATS.201605-359OC
85. Lanham-New SA, Webb AR, Cashman KD, et al. Vitamin D and SARS-CoV-2 virus/COVID-19 disease. *BMJ Nutrition, Prevention & Health* 2020:bmjnph-2020-000089. doi: 10.1136/bmjnph-2020-000089
86. Huang F, Zhang C, Liu Q, et al. Identification of amitriptyline HCl, flavin adenine dinucleotide, azacitidine and calcitriol as repurposing drugs for influenza A H5N1 virus-induced lung injury. *PLoS Pathog* 2020;16(3):e1008341. doi: 10.1371/journal.ppat.1008341
87. Lee C. Controversial effects of vitamin D and related genes on viral infections, pathogenesis, and treatment outcomes. *Nutrients* 2020;12(4):962. doi: 10.3390/nu12040962

For peer review only - http://bmjopen.bmj.com/site/about/guidelines.xhtml

88. Impact of zinc and vitamin D3 supplementation on the survival of aged patients infected with COVID-19. NCT04351490. ClinicalTrials.gov, U.S. National Library of Medicine; 2020. https://ClinicalTrials.gov/show/NCT04351490
89. COvid-19 and Vitamin D supplementation: a multicenter randomized controlled trial of high dose versus standard dose vitamin D3 in high-risk COVID-19 Patients (CoVitTrial). NCT04344041. ClinicalTrials.gov, U.S. National Library of Medicine; 2020. https://clinicaltrials.gov/show/NCT04344041
90. The VITDALIZE Study: effect of high-dose vitamin D3 on 28-day mortality in adult critically ill patients. NCT03188796. ClinicalTrials.gov, U.S. National Library of Medicine; 2017. https://ClinicalTrials.gov/show/NCT03188796
91. Scientific Advisory Committee on Nutrition, European Food Safety Authority. Joint explanatory note by the European Food Safety Authority and the UK Scientific Advisory Committee on Nutrition regarding dietary reference values for vitamin D. 2016. https://www.efsa.europa.eu/sites/default/files/documents/news/explanatory_note_EFSA_SACN_vitaminD.pdf

Figure legends

Figure 1. PRISMA flow diagram for the selection of studies

For peer review only

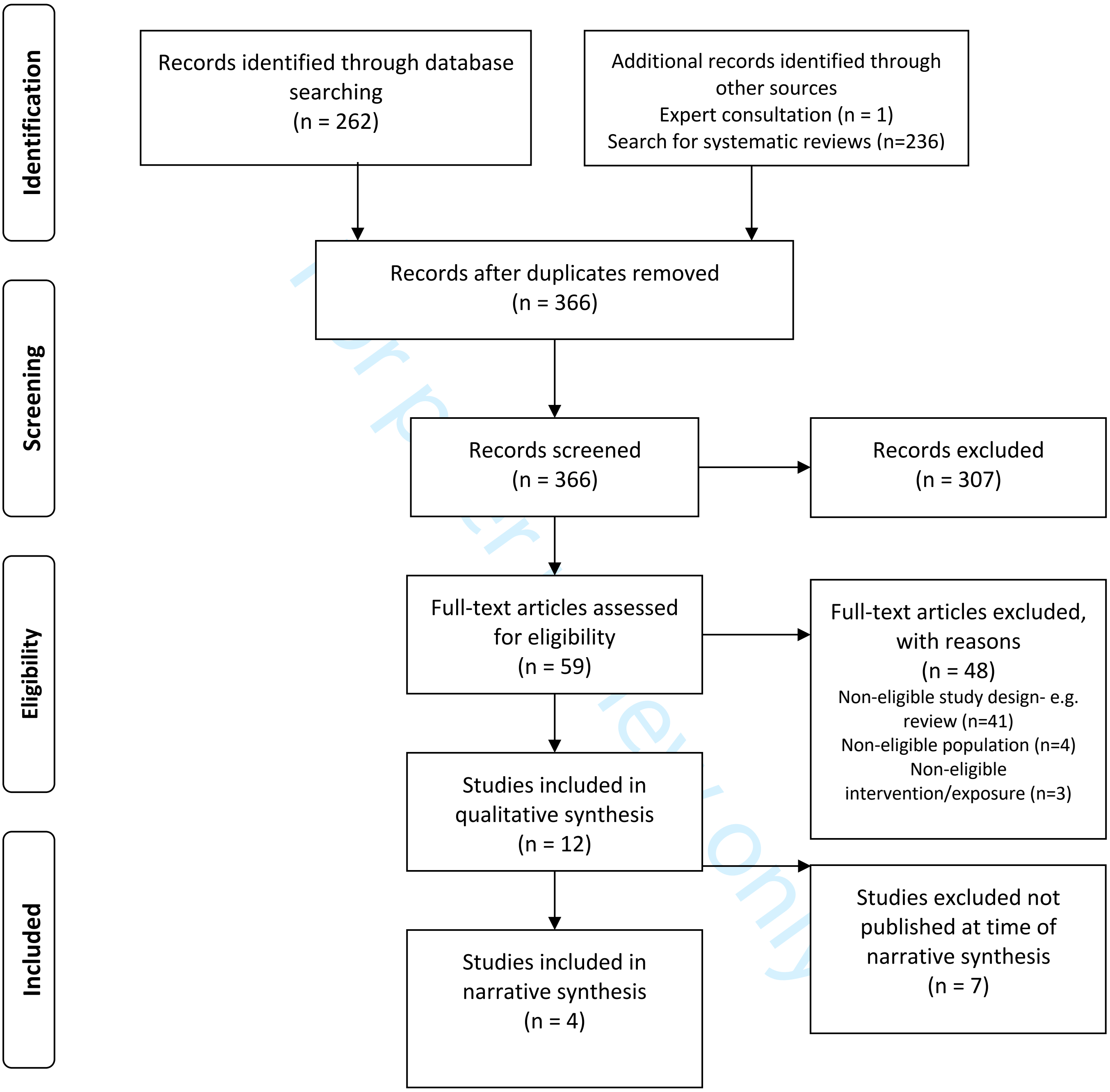
Identification
Screening
Eligibility
Included
Records identified through database searching (n = 262)
Additional records identified through other sources
Expert consultation (n = 1)
Search for systematic reviews (n=236)
Records after duplicates removed (n = 366)
Records screened (n = 366)
Records excluded (n = 307)
Full-text articles assessed for eligibility (n = 59)
Full-text articles excluded, with reasons (n = 48)
Non-eligible study design- e.g. review (n=41)
Non-eligible population (n=4)
Non-eligible intervention/exposure (n=3)
Studies included in qualitative synthesis (n = 12)
Studies excluded not published at time of narrative synthesis (n = 7)
Studies included in narrative synthesis (n = 4)

Supplemental file

Contents

1. Full record of search

2. Full details of the study eligibility criteria

3. List of studies excluded at full text review

4. Articles included at full text, but later excluded at time of narrative synthesis

5. Quality assessment of included studies

For peer review only

1. <u>Full record of search</u>

Medline (Ovid)

Search date: 06/05/2020
Database: Ovid MEDLINE(R) ALL <1946 to May 05, 2020>
Search Strategy:
----
1 exp Vitamin D/ (58492)
2 Vitamin D Deficiency/ (15552)
3 (vitamin D? or vit D? or D vitamin? or ergocalciferol or cholecalciferol or colecalciferol or calciferol or dihydroergocalciferol or sitocalciferol or dihydrotachysterol or dihydroxycholecalciferol? or hydroxycholecalciferol? or "25(OH)D?" or 25OHD? or calcidiol or hydroxyergocalciferol or alfacalcidol or alphacalcidol or "1,25(OH)2D" or hydroxyvitamin D? or dihydroxyvitamin D?).ab,kf,ti. (78232)
4 (paricalcitol or doxercalciferol or doxerocalciferol or calcifediol or calcitriol).ab,kf,ti. (5577)
5 hypovitaminosis D?.ab,kf,ti. (1775)
6 ((D or D2 or D3) adj3 supplement*).ab,kf,ti. (12158)
7 1 or 2 or 3 or 4 or 5 or 6 (92560)
8 coronavirus/ or betacoronavirus/ or betacoronavirus 1/ or coronavirus oc43, human/ or middle east respiratory syndrome coronavirus/ or sars virus/ (7431)
9 coronavirus infections/ or severe acute respiratory syndrome/ (10675)
10 (((corona* or corono*) adj1 (virus* or viral* or virinae*)) or coronavirus* or coronovirus* or coronavirinae* or Coronavirus* or Coronovirus* or Wuhan* or Hubei* or Huanan or "2019-nCoV" or 2019nCoV or nCoV2019 or "nCoV-2019" or "COVID-19" or COVID19 or "CORVID-19" or CORVID19 or "WN-CoV" or WNCoV or "HCoV-19" or HCoV19 or CoV or "2019 novel*" or Ncov or "n-cov" or "SARS-CoV-2" or "SARSCoV-2" or "SARSCoV2" or "SARS-CoV2" or SARSCov19 or "SARS-Cov19" or "SARSCov-19" or "SARS-Cov-19" or Ncovor or Ncorona* or Ncorono* or NcovWuhan* or NcovHubei* or NcovChina* or NcovChinese*).ab,kf,ti. (26891)
11 (severe acute respiratory syndrome or SARS or Middle East respiratory syndrome or MERS).ab,kf,ti. (16560)
12 (betacoronavirus* or betacoronavirinae*).ab,kf,ti. (280)
13 8 or 9 or 10 or 11 or 12 (37180)
14 7 and 13 (32)
15 exp Animals/ (23144176)

16 exp Humans/ (18448248)
17 15 not 16 (4695928)
18 14 not 17 (30)
19 limit 18 to yr="2002 -Current" (30)

Update
Search date: 10/6/2020
Actual databases searched: Ovid MEDLINE All <1946 to June 09, 2020>
Search strategy:
Re-ran search above plus…
20 limit 19 to ed=20200506-20200610 (8)
21 limit 19 to ep=20200506-20200610 (39)
22 limit 19 to dt=20200506-20200610 (43)
23 limit 19 to ez=20200506-20200610 (27)
24 20 or 21 or 22 or 23 (46)

Embase (Ovid)

Search date: 06/05/2020
Database: Embase <1974 to 2020 May 05>
Search Strategy:
----
1 exp vitamin D/ (139781)
2 vitamin D deficiency/ (29333)
3 (vitamin D? or vit D? or D vitamin? or ergocalciferol or cholecalciferol or colecalciferol or calciferol or dihydroergocalciferol or sitocalciferol or dihydrotachysterol or dihydroxycholecalciferol? or hydroxycholecalciferol? or "25(OH)D?" or 25OHD? or calcidiol or hydroxyergocalciferol or alfacalcidol or alphacalcidol or "1,25(OH)2D" or hydroxyvitamin D? or dihydroxyvitamin D?).ab,kw,ti. (112459)
4 (paricalcitol or doxercalciferol or doxerocalciferol or calcifediol or calcitriol).ab,kw,ti. (8478)
5 hypovitaminosis D?.ab,kw,ti. (3012)
6 ((D or D2 or D3) adj3 supplement*).ab,kw,ti. (19177)
7 1 or 2 or 3 or 4 or 5 or 6 (163395)
8 betacoronavirus 1/ or betacoronavirus/ or human coronavirus oc43/ (696)
9 Middle East respiratory syndrome coronavirus/ (2028)
10 sars-related coronavirus/ or sars coronavirus/ (6354)
11 Coronavirinae/ (2231)
12 coronavirus infection/ or middle east respiratory syndrome/ or severe acute respiratory syndrome/ (11950)
13 (((corona* or corono*) adj1 (virus* or viral* or virinae*)) or coronavirus* or coronovirus* or coronavirinae* or Coronavirus* or Coronovirus* or Wuhan* or Hubei* or Huanan or "2019-nCoV" or 2019nCoV or nCoV2019 or "nCoV-2019" or "COVID-19" or COVID19 or "CORVID-19" or CORVID19 or "WN-CoV" or WNCoV or "HCoV-19" or HCoV19 or CoV or "2019 novel*" or Ncov or "n-cov" or "SARS-CoV-2" or "SARSCoV-2" or "SARSCoV2" or "SARS-CoV2" or SARSCov19 or "SARS-Cov19" or "SARSCov-19" or "SARS-Cov-19" or Ncovor or Ncorona* or Ncorono* or NcovWuhan* or NcovHubei* or NcovChina* or NcovChinese*).ab,kw,ti. (27686)
14 (severe acute respiratory syndrome or SARS or Middle East respiratory syndrome or MERS).ab,kw,ti. (17146)
15 (betacoronavirus* or betacoronavirinae*).ab,kw,ti. (275)
16 8 or 9 or 10 or 11 or 12 or 13 or 14 or 15 (40716)

For peer review only

17 7 and 16 (61)
18 exp animal/ (25459151)
19 exp human/ (20834835)
20 18 not 19 (4624316)
21 17 not 20 (58)
22 limit 21 to yr="2002 -Current" (58)

Update
Search date: 10/6/2020
Actual databases searched: Ovid Embase <1974 to 2020 June 09>
Search strategy:
Re-ran search above plus…
22 limit 21 to yr="2002 -Current" (123)
23 limit 22 to dd=20200506-20200610 (39)
24 limit 22 to em=202005-202006 (0)
25 limit 22 to dc=20200506-20200610 (62)
26 23 or 24 or 25 (62)

MedrXiv (searched via Medrxivr https://mcguinlu.shinyapps.io/medrxivr/)

Search date: 07/05/2020
Search Strategy:

Topic 1:

[Vv]itamin D
[Vv]itamin D2
[Vv]itamin D3
calciferol
25OHD
25OHD3
[Hh]ypovitaminosis D

Topic 2:

[Cc]oronavirus
[Cc]orona(\\s)([[:graph:]]+\\s){0,1}virus
[Cc]oronavirinae
[Cc]ovid
COVID
nCoV
NCOV
Ncov
[Nn]-cov
N-COV
2019ncov
2019-ncov
ncov2019
ncov-2019

For peer review only

SARS
[Ss]evere [Aa]cute [Rr]espiratory [Ss]yndrome
[Mm]iddle [Ee]ast [Rr]espiratory [Ss]yndrome
MERS

Earliest record date
20190101
Latest record date
20200507
Remove older versions of the same record

6 results

Update
Search date: 10/6/2020
Re-ran search above changing record dates as follows:
Earliest record date
20200507
Latest record date
20200610
Remove older versions of the same record

11 results

BioRxiv
https://www.biorxiv.org/

Search date: 07/05/2020

65 Results
for abstract or title "vitamin D" (match phrase words)

22 Results
for full text or abstract or title "ergocalciferol cholecalciferol colecalciferol" (match whole any)

41 Results
for full text or abstract or title "25OHD 25OHD3" (match whole any)

Imported into EndNote and de-duplicated
92 results after deduplication

Searched in Endnote using the following search strategy:
coronavirus or corona or covid or SARS or MERS or betacoronavirus or ncov
Any Field

5 results

Update
Search date: 10/6/2020

For peer review only - http://bmjopen.bmj.com/site/about/guidelines.xhtml

1 Results
for abstract or title "vitamin D" (match phrase words) and posted between "07 May, 2020 and 10 Jun, 2020" – *animal study (also in both results sets below) so not exported to EndNote*

3 Results
for full text or abstract or title "ergocalciferol cholecalciferol colecalciferol" (match whole any) and posted between "07 May, 2020 and 10 Jun, 2020" *- 2 animal studies and 1 on sertraline in TB*

2 Results
for full text or abstract or title "25OHD 25OHD3" (match whole any) and posted between "07 May, 2020 and 10 Jun, 2020" *- 1 animal study, 1 non-clinical / non-coronavirus*

0 results relevant to coronaviruses

Cochrane Library

Search date: 08/05/2020

ID Search Hits
#1 MeSH descriptor: [Vitamin D] explode all trees 5224
#2 MeSH descriptor: [Vitamin D Deficiency] this term only 1226
#3 ((vitamin NEXT D?) or (vit NEXT D?) or (D NEXT vitamin?) or ergocalciferol or cholecalciferol or colecalciferol or calciferol or dihydroergocalciferol or sitocalciferol or dihydrotachysterol or dihydroxycholecalciferol? or hydroxycholecalciferol? or "25(OH)D" or "25(OH)D3" or 25OHD? or calcidiol or hydroxyergocalciferol or alfacalcidol or alphacalcidol or "1,25(OH)2D" or (hydroxyvitamin NEXT D?) or (dihydroxyvitamin NEXT D?)):ti,ab,kw 12959
#4 (paricalcitol or doxercalciferol or doxerocalciferol or calcifediol or calcitriol):ti,ab,kw 2417
#5 hypovitaminosis NEXT D? 303
#6 ((D OR D2 OR D3) NEAR/3 supplement*):ti,ab,kw 5633
#7 #1 or #2 or #3 or #4 or #5 or #6 14461
#8 MeSH descriptor: [Coronavirus] this term only 2
#9 MeSH descriptor: [Betacoronavirus] this term only 2
#10 MeSH descriptor: [Betacoronavirus 1] this term only 0
#11 MeSH descriptor: [Coronavirus OC43, Human] this term only 0
#12 MeSH descriptor: [Middle East Respiratory Syndrome Coronavirus] explode all trees 1
#13 MeSH descriptor: [SARS Virus] this term only 9
#14 MeSH descriptor: [Coronavirus Infections] this term only 137
#15 MeSH descriptor: [Severe Acute Respiratory Syndrome] this term only 107
#16 (((corona* or corono*) near/1 (virus* or viral* or virinae*)) or coronavirus* or coronovirus* or coronavirinae* or Coronavirus* or Coronovirus* or Wuhan* or Hubei* or Huanan or "2019-nCoV" or 2019nCoV or nCoV2019 or "nCoV-2019" or "COVID-19" or COVID19 or "CORVID-19" or CORVID19 or "WN-CoV" or WNCoV or "HCoV-19" or HCoV19 or CoV or "2019 novel*" or Ncov or "n-cov" or "SARS-CoV-2" or "SARSCoV-2" or "SARSCoV2" or "SARS-CoV2" or SARSCov19 or "SARS-Cov19" or "SARSCov-19" or "SARS-Cov-19" or Ncovor or Ncorona* or Ncorono* or NcovWuhan* or NcovHubei* or NcovChina* or NcovChinese*):ti,ab,kw 614
#17 ("severe acute respiratory syndrome" or SARS or "Middle East respiratory syndrome" or MERS):ti,ab,kw 350
#18 (betacoronavirus* or betacoronavirinae*):ti,ab,kw 4

For peer review only

#19 #8 or #9 or #10 or #11 or #12 or #13 or #14 or #15 or #16 or #17 or #18 798
#20 #7 and #19 3

[all 3 results were from CENTRAL]

Update
Search date: 10/06/2020
Re-ran search exactly as above and retrieved **5** results, all from CENTRAL. All 5 results exported to EndNote for deduplication.

Database of publications (living map of evidence) on coronavirus disease (COVID-19) developed by the University of Bern

Living Evidence on COVID-19
Contributors: Michel Counotte, Hira Imeri, Mert Ipekci, Nicola Low

https://zika.ispm.unibe.ch/assets/data/pub/ncov/

Search date: 10/05/2020 (14,988 entries)

Search: Title, Abstract
Search:

vitamin D 13
vitamin D2 0
vitamin D3 0
ergocalciferol 0
cholecalciferol 0
colecalciferol 0
25(OH)D 0
25OHD 0
25(OH)D3 0
25OHD3 0
hypovitaminosis D 1
Vitamin D Deficiency 1

Oxford COVID-19 Evidence Service
https://www.cebm.net/oxford-covid-19-evidence-service/
The Centre for Evidence-Based Medicine (CEBM) The University of Oxford

Search date: 10/05/2020 (142 articles)
vitamin D 1
vitamin D2 0
vitamin D3 0
ergocalciferol 0
cholecalciferol 0
colecalciferol 0
25(OH)D 0
25OHD 0

For peer review only

25(OH)D3 0
25OHD3 0
hypovitaminosis D 0
Vitamin D Deficiency 0

Database of publications on coronavirus disease (COVID-19) developed by WHO
https://www.who.int/emergencies/diseases/novel-coronavirus-2019/global-research-on-novel-coronavirus-2019-ncov

Search date: 10/05/2020 (15,253 entries)

Search: Title, Abstract, Subject

vitamin D 19
vitamin D2 0
vitamin D3 2
ergocalciferol 0
cholecalciferol 1
colecalciferol 0
25(OH)D 0
25OHD 0
25(OH)D3 0
25OHD3 0
hypovitaminosis D 1
Vitamin D Deficiency 2

Total: 25
After de-duplication: 20

Searches for systematic reviews, for reference checking

Medline

Search date: 19/05/2020
Database: Ovid MEDLINE(R) ALL <1946 to May 18, 2020>
Search Strategy:
----
1 exp Vitamin D/ (58577)
2 Vitamin D Deficiency/ (15588)
3 (vitamin D? or vit D? or D vitamin? or ergocalciferol or cholecalciferol or colecalciferol or calciferol or dihydroergocalciferol or sitocalciferol or dihydrotachysterol or dihydroxycholecalciferol? or hydroxycholecalciferol? or "25(OH)D?" or 25OHD? or calcidiol or hydroxyergocalciferol or alfacalcidol or alphacalcidol or "1,25(OH)2D" or hydroxyvitamin D? or dihydroxyvitamin D?).ab,kf,ti. (78395)
4 (paricalcitol or doxercalciferol or doxerocalciferol or calcifediol or calcitriol).ab,kf,ti. (5588)
5 hypovitaminosis D?.ab,kf,ti. (1780)
6 ((D or D2 or D3) adj3 supplement*).ab,kf,ti. (12198)
7 1 or 2 or 3 or 4 or 5 or 6 (92747)

For peer review only - http://bmjopen.bmj.com/site/about/guidelines.xhtml

8 coronavirus/ or betacoronavirus/ or betacoronavirus 1/ or coronavirus oc43, human/ or middle east respiratory syndrome coronavirus/ or sars virus/ (8161)
9 coronavirus infections/ or severe acute respiratory syndrome/ (11614)
10 (((corona* or corono*) adj1 (virus* or viral* or virinae*)) or coronavirus* or coronovirus* or coronavirinae* or Coronavirus* or Coronovirus* or Wuhan* or Hubei* or Huanan or "2019-nCoV" or 2019nCoV or nCoV2019 or "nCoV-2019" or "COVID-19" or COVID19 or "CORVID-19" or CORVID19 or "WN-CoV" or WNCoV or "HCoV-19" or HCoV19 or CoV or "2019 novel*" or Ncov or "n-cov" or "SARS-CoV-2" or "SARSCoV-2" or "SARSCoV2" or "SARS-CoV2" or SARSCov19 or "SARS-Cov19" or "SARSCov-19" or "SARS-Cov-19" or Ncovor or Ncorona* or Ncorono* or NcovWuhan* or NcovHubei* or NcovChina* or NcovChinese*).ab,kf,ti. (31115)
11 (severe acute respiratory syndrome or SARS or Middle East respiratory syndrome or MERS).ab,kf,ti. (17795)
12 (betacoronavirus* or betacoronavirinae*).ab,kf,ti. (294)
13 exp Respiratory Tract Infections/ (356696)
14 (acute respiratory infection* or severe respiratory infection* or acute respiratory tract infection* or severe respiratory tract infection* or influenza or common cold or pneumonia or bronchitis).ab,kf,ti. (234266)
15 8 or 9 or 10 or 11 or 12 or 13 or 14 (503079)
16 7 and 15 (1062)
17 (metaanalys* or "meta analys*" or "meta-analys*").tw. (169008)
18 (systematic* adj3 review*).mp. (200684)
19 meta analysis.pt. (114746)
20 17 or 18 or 19 (301767)
21 16 and 20 (55)

Embase

Search date: 19/05/2020

Database: Embase Classic+Embase <1947 to 2020 Week 20>
Search Strategy:
----
1 exp vitamin D/ (147053)
2 vitamin D deficiency/ (30106)
3 (vitamin D? or vit D? or D vitamin? or ergocalciferol or cholecalciferol or colecalciferol or calciferol or dihydroergocalciferol or sitocalciferol or dihydrotachysterol or dihydroxycholecalciferol? or hydroxycholecalciferol? or "25(OH)D?" or 25OHD? or calcidiol or hydroxyergocalciferol or alfacalcidol or alphacalcidol or "1,25(OH)2D" or hydroxyvitamin D? or dihydroxyvitamin D?).ab,kw,ti. (118981)
4 (paricalcitol or doxercalciferol or doxerocalciferol or calcifediol or calcitriol).ab,kw,ti. (8485)
5 hypovitaminosis D?.ab,kw,ti. (3033)
6 ((D or D2 or D3) adj3 supplement*).ab,kw,ti. (19335)
7 1 or 2 or 3 or 4 or 5 or 6 (172654)
8 betacoronavirus 1/ or betacoronavirus/ or human coronavirus oc43/ (1085)
9 Middle East respiratory syndrome coronavirus/ (2082)
10 sars-related coronavirus/ or sars coronavirus/ (6062)
11 Coronavirinae/ (2060)
12 coronavirus infection/ or middle east respiratory syndrome/ or severe acute respiratory syndrome/ (12565)

For peer review only - http://bmjopen.bmj.com/site/about/guidelines.xhtml

13 (((corona* or corono*) adj1 (virus* or viral* or virinae*)) or coronavirus* or coronovirus* or coronavirinae* or Coronavirus* or Coronovirus* or Wuhan* or Hubei* or Huanan or "2019-nCoV" or 2019nCoV or nCoV2019 or "nCoV-2019" or "COVID-19" or COVID19 or "CORVID-19" or CORVID19 or "WN-CoV" or WNCoV or "HCoV-19" or HCoV19 or CoV or "2019 novel*" or Ncov or "n-cov" or "SARS-CoV-2" or "SARSCoV-2" or "SARSCoV2" or "SARS-CoV2" or SARSCov19 or "SARS-Cov19" or "SARSCov-19" or "SARS-Cov-19" or Ncovor or Ncorona* or Ncorono* or NcovWuhan* or NcovHubei* or NcovChina* or NcovChinese*).ab,kw,ti. (30532)
14 (severe acute respiratory syndrome or SARS or Middle East respiratory syndrome or MERS).ab,kw,ti. (17954)
15 (betacoronavirus* or betacoronavirinae*).ab,kw,ti. (286)
16 exp respiratory tract infection/ (460049)
17 (acute respiratory infection* or severe respiratory infection* or acute respiratory tract infection* or severe respiratory tract infection* or influenza or common cold or pneumonia or bronchitis).ab,kw,ti. (329779)
18 8 or 9 or 10 or 11 or 12 or 13 or 14 or 15 or 16 or 17 (674800)
19 7 and 18 (3315)
20 (metaanalys* or "meta analys*" or "meta-analys*").mp. (294469)
21 (systematic* adj2 review*).mp. (330720)
22 20 or 21 (475492)
23 19 and 22 (219)
24 limit 19 to (meta analysis or "systematic review") (145)
25 23 or 24 (219)
26 limit 25 to (conference abstract or conference paper or "conference review" or editorial or letter) (41)
27 25 not 26 (178)

Cochrane Database of Systematic Reviews (Cochrane Library)

Search Name: Vitamin D Covid and Acute Respiratory Infections SRs
Date Run: 20/05/2020 18:30:28
Comment:

ID	Search	Hits
#1	MeSH descriptor: [Vitamin D] explode all trees	5224
#2	MeSH descriptor: [Vitamin D Deficiency] this term only	1226
#3	((vitamin NEXT D?) or (vit NEXT D?) or (D NEXT vitamin?) or ergocalciferol or cholecalciferol or colecalciferol or calciferol or dihydroergocalciferol or sitocalciferol or dihydrotachysterol or dihydroxycholecalciferol? or hydroxycholecalciferol? or "25(OH)D" or "25(OH)D3" or 25OHD? or calcidiol or hydroxyergocalciferol or alfacalcidol or alphacalcidol or "1,25(OH)2D" or (hydroxyvitamin NEXT D?) or (dihydroxyvitamin NEXT D?)):ti,ab,kw	12959
#4	(paricalcitol or doxercalciferol or doxerocalciferol or calcifediol or calcitriol):ti,ab,kw	2417
#5	hypovitaminosis NEXT D?	303
#6	((D OR D2 OR D3) NEAR/3 supplement*):ti,ab,kw	5632
#7	#1 or #2 or #3 or #4 or #5 or #6	14461
#8	MeSH descriptor: [Coronavirus] this term only	2
#9	MeSH descriptor: [Betacoronavirus] this term only	2
#10	MeSH descriptor: [Betacoronavirus 1] this term only	0
#11	MeSH descriptor: [Coronavirus OC43, Human] this term only	0
#12	MeSH descriptor: [Middle East Respiratory Syndrome Coronavirus] explode all trees	1

For peer review only - http://bmjopen.bmj.com/site/about/guidelines.xhtml

#13 MeSH descriptor: [SARS Virus] this term only 9
#14 MeSH descriptor: [Coronavirus Infections] this term only 137
#15 MeSH descriptor: [Severe Acute Respiratory Syndrome] this term only 107
#16 (((corona* or corono*) near/1 (virus* or viral* or virinae*)) or coronavirus* or coronovirus* or coronavirinae* or Coronavirus* or Coronovirus* or Wuhan* or Hubei* or Huanan or "2019-nCoV" or 2019nCoV or nCoV2019 or "nCoV-2019" or "COVID-19" or COVID19 or "CORVID-19" or CORVID19 or "WN-CoV" or WNCoV or "HCoV-19" or HCoV19 or CoV or "2019 novel*" or Ncov or "n-cov" or "SARS-CoV-2" or "SARSCoV-2" or "SARSCoV2" or "SARS-CoV2" or SARSCov19 or "SARS-Cov19" or "SARSCov-19" or "SARS-Cov-19" or Ncovor or Ncorona* or Ncorono* or NcovWuhan* or NcovHubei* or NcovChina* or NcovChinese*):ti,ab,kw 616
#17 ("severe acute respiratory syndrome" or SARS or "Middle East respiratory syndrome" or MERS):ti,ab,kw 351
#18 (betacoronavirus* or betacoronavirinae*):ti,ab,kw 4
#19 MeSH descriptor: [Respiratory Tract Infections] explode all trees 14360
#20 (("acute respiratory" NEXT infection*) or ("severe respiratory" NEXT infection*) or ("acute respiratory tract" NEXT infection*) or ("severe respiratory tract" NEXT infection*) or influenza or "common cold" or pneumonia or bronchitis):ti,ab,kw 25944
#21 #8 or #9 or #10 or #11 or #12 or #13 or #14 or #15 or #16 or #17 or #18 or #19 or #20 32554
#22 #7 and #21 329

CDSR: 3

Expert consultation

One additional study identified:

Martineau AR, Jolliffe DA, Hooper RL, et al., (2017) Vitamin D supplementation to prevent acute respiratory tract infections: systematic review and meta-analysis of individual participant data. BMJ. 2017;356:i6583. doi:10.1136/bmj.i6583

For peer review only

2. Full details of the study eligibility criteria

Include	Exclude
**P- Population**<br><br>1) Patients acutely ill with Betacoronavirus infection [SARS-CoV, MERS-CoV, SARS-CoV-2]<br>2) or at risk of acute illness with Betacoronavirus infection	Animals studies, modelling studies
**I – Intervention/exposure**<br><br>1) Vitamin D supplementation<br>2) Low Serum Vitamin D	
**O - Outcomes**<br><br>1) Betacoronavirus infection (to include serological evidence of infection or clinically confirmed symptomatic infection);<br>2) severity of Betacoronavirus infection (to include patients admitted to hospital or admitted to intensive care); mortality due to Betacoronavirus.<br>3) Mortality due to Betacoronavirus	
**C – Comparator**<br><br>1) No Vitamin D supplementation<br>2) high or normal Serum Vitamin D	
**S - Study design**<br>Randomised controlled trials and non-randomized studies will be eligible for inclusion in the review including, non randomized controlled trials, interrupted time series, controlled before-and-after studies, cohort studies, ecological studies, case reports and case series.	Qualitative studies, Non-primary research- reviews, editorials etc, guidelines and non-systematic reviews.
**Subgroups**<br><br>1. Ethnicity characteristics (White British, All Other White, Mixed, Asian, Black, Other)<br>2. Age characteristics (population by five-year age groups)	Non-English language. Non peer reviewed publication.

For peer review only

3. List of studies excluded at full text review

Excluded studies		Reason<br><br>*Non-eligible study design- e.g. review*<br>*Non-eligible population*<br>*Non-eligible intervention*<br>*No relevant outcome*<br>*No comparator group*
1	Adams, K. K., et al. (2020). "Myth Busters: Dietary Supplements and COVID-19." Annals of Pharmacotherapy: 1060028020928052.	Study design – commentary
2	Ahmed, I., et al. (2020). "First Covid-19 maternal mortality in the UK associated with thrombotic complications." British Journal of Haematology. **18**.	Study design – letter
3	Alpalhao, M. and P. Filipe (2020). "SARS-CoV-2 pandemic and Vitamin D deficiency - a double trouble." Photodermatology, Photoimmunology & Photomedicine **01**: 01.	Study design – letter
4	Annweiler, C., et al. (2020). COvid-19 and Vitamin D Supplementation: a Multicenter Randomized Controlled Trial of High Dose Versus Standard Dose Vitamin D3 in High-risk COVID-19 Patients (CoVitTrial). https://clinicaltrials.gov/show/NCT04344041.	Study design – on-going RCT completion date. July 2020
5	Arya, A. and V. D. Dwivedi (2020). "Synergistic effect of vitamin D and remdesivir can fight COVID-19." Journal of Biomolecular Structure & Dynamics: 1-2	Study design – letter
6	Banerjee, D., et al. (2020). "COVID-19 infection in kidney transplant recipients." Kidney International **97**(6): 1076-1082.	Study design – commentary
7	Caccialanza, R., et al. (2020). Early nutritional supplementation in non-critically ill patients hospitalized for the 2019 novel coronavirus disease (COVID-19): Rationale and feasibility of a shared pragmatic protocol. Nutrition: 110835.	Study design - protocol
8	Calder, P. C., et al. (2020). Optimal Nutritional Status for a Well-Functioning Immune System Is an Important Factor to Protect against Viral Infections. Nutrients: 12(4), 1181.	Study design -narrative review
9	Cao, Z., et al. (2020). SARS-CoV-2 & Covid-19: Key-Roles of the 'Renin-Angiotensin' System / Vitamin D Impacting Drug and Vaccine Developments. Infectious Disorders - Drug Targets. *(E-pub Ahead of Print)* DOI : 10.2174/1871526520999200505174704	Study design – editorial
10	Carter, S. J., et al. (2020). Considerations for obesity, vitamin D, and physical activity amidst the COVID-19 pandemic. Obesity **16**: 16.	Study design -narrative review

For peer review only - http://bmjopen.bmj.com/site/about/guidelines.xhtml

11	Daneshkhah, A., et al. (2020). The Possible Role of Vitamin D in Suppressing Cytokine Storm and Associated Mortality in COVID-19 Patients. medRxiv. DOI: https://doi.org/10.1101/2020.04.08.20058578	Study design – modelling
12	Davies G, Garami AR, Byers JC. Evidence Supports a Causal Model for Vitamin D in COVID-19 Outcomes. medRxiv, 2020. DOR: https://doi.org/10.1101/2020.05.01.20087965v3	Study design – modelling
13	de Lucena, T. M. C., et al. (2020). "Mechanism of inflammatory response in associated comorbidities in COVID-19." Diabetes & Metabolic Syndrome **14**(4): 597-600.	Study design -narrative review
14	Eroglu, C., et al. (2019). The relation between serum vitamin D levels, viral infections and severity of attacks in children with recurrent wheezing. Allergologia et Immunopathologia **47**(6): 591-597.	Population - Not COVID-19/SARs/MERs
15	Faul, J. L., et al. (2020). "Vitamin d deficiency and ards after sars-cov-2 infection." Irish Medical Journal **113**(5).	Study design – letter
16	Ghasemian, R., et al. (2020). "The Role of Vitamin D in The Age of COVID-19: A Systematic Review and Meta-Analysis Along with an Ecological Approach." MedRxiv : the Preprint Server for Health Sciences. DOI: 10.1101/2020.06.05.20123554	Study design -narrative review
17	Grant, W. B., et al. (2020). Evidence that Vitamin D Supplementation Could Reduce Risk of Influenza and COVID-19 Infections and Deaths. Nutrients **12**(4): 02.	Study design -narrative review
18	Heiser, K., et al. (2020). Identification of potential treatments for COVID-19 through artificial intelligence-enabled phenomic analysis of human cells infected with SARS-CoV-2. bioRxiv: 2020.2004.2021.054387.	Study design – modelling
19	Hribar, C. A., et al. (2020). "Potential Role of Vitamin D in the Elderly to Resist COVID-19 and to Slow Progression of Parkinson's Disease." Brain Sciences **10**(5): 08.	Study design -narrative review
20	Jakovac, H. (2020). COVID-19 and vitamin D-Is there a link and an opportunity for intervention? American Journal of Physiology - Endocrinology & Metabolism **318**(5): E589-E589.	Study design – letter
21	Jamaati, H., et al. (2020). A fourteen-day experience with coronavirus disease 2019 (COVID-19) induced acute respiratory distress syndrome (ARDS): An Iranian treatment protocol. Iranian Journal of Pharmaceutical Research **19**(1): 31-36.	Study design – treatment protocol/recommendation
22	Kalippurayil Moozhipurath, R., et al. (2020). "Evidence of Protective Role of Ultraviolet-B (UVB) Radiation in	Exposure – not vitamin D(UVB)

For peer review only - http://bmjopen.bmj.com/site/about/guidelines.xhtml

	Reducing COVID-19 Deaths." MedRxiv : the Preprint Server for Health Sciences. DOI 10.1101/2020.05.06.20093419	
23	Kara, M., et al. (2020). "'Scientific Strabismus' or Two Related Pandemics: COVID-19 & Vitamin D Deficiency." British Journal of Nutrition: 1-20.	Study design -narrative review
24	Koivisto, O., et al. (2020). Key Vitamin D Target Genes with Functions in the Immune System. Nutrients, **12**(4):1140.	Population - Outcomes - target genes
25	Kow, C. S., et al. (2020). "Vitamin D Supplementation in Influenza and COVID-19 Infections Comment on: "Evidence that Vitamin D Supplementation Could Reduce Risk of Influenza and COVID-19 Infections and Deaths" Nutrients 2020, 12(4), 988." Nutrients **12**(6): 01.	Study design – commentary
26	Kumar, V. and A. Srivastava (2020). "Spurious Correlation? A review of the relationship between Vitamin D and Covid-19 infection and mortality." MedRxiv : the Preprint Server for Health Sciences. DOI: 10.1101/2020.05.25.20110338	Study design -narrative review
27	Laird, E., et al. (2020). "Vitamin D and inflammation: Potential implications for severity of Covid-19." Irish Medical Journal **113**(5).	Study design -narrative review
28	La Vignera, S., et al. (2020). Sex-Specific SARS-CoV-2 Mortality: Among Hormone-Modulated ACE2 Expression, Risk of Venous Thromboembolism and Hypovitaminosis D. International journal of molecular sciences **21**(8):2948.	Study design – editorial
29	Li, A. Y., et al. (2020). Multivariate Analysis of Factors Affecting COVID-19 Case and Death Rate in U.S. Counties: The Significant Effects of Black Race and Temperature. medRxiv. DOI: https://doi.org/10.1101/2020.04.17.20069708	Intervention – not Vitamin D
30	Maestri, E., et al. (2020). Vitamin D and coronavirus: a new field of use?. Recenti Progressi in Medicina **111**(4): 253-256.	Study design -narrative review, non-English
31	Mansbach, J. M. and C. A. Camargo Jr., (2009). Respiratory Viruses in Bronchiolitis and Their Link to Recurrent Wheezing and Asthma. Clinics in Laboratory Medicine **29**(4): 741-755.	Population - Not COVID-19/SARs/MERs
32	Marik, P. E., et al. (2020). Does vitamin D status impact mortality from SARS-CoV-2 infection? Medicine in Drug Discovery: 100041-100041.	Study design - commentary
33	McKenna, M. J. and M. A. T. Flynn (2020). "Covid-19, cocooning and vitamin d intake requirements." Irish Medical Journal **113**(5).	Study design -narrative review

For peer review only

34	Mitchell, F. (2020). "Vitamin-D and COVID-19: do deficient risk a poorer outcome?" The Lancet Diabetes & Endocrinology **20**: 20.	Study design -narrative review
35	Molloy, E. J. and N. Murphy (2020). Vitamin D, Covid-19 and Children. Irish Medical Journal **113**(4): 64.	Study design -narrative review
36	McCartney, D. M. and D. G. Byrne (2020). Optimisation of Vitamin D Status for Enhanced Immuno-protection Against Covid-19. Irish Medical Journal **113**(4): 58.	Study design -narrative review
37	Rabbitt, L. and E. Slattery (2020). "Vitamin d and covid-19: A note of caution." Irish Medical Journal **113**(5).	Study design - letter
38	Ribeiro, H., et al. (2020). "Does Vitamin D play a role in the management of Covid-19 in Brazil?" Revista de Saude Publica **54**: 53.	Study design -narrative review
39	Romano, L., et al. (2020). "Short Report - Medical nutrition therapy for critically ill patients with COVID-19." European Review for Medical and Pharmacological Sciences **24**(7): 4035-4039.	Study design -narrative review
40	Silberstein, M. (2020). Vitamin D: A simpler alternative to tocilizumab for trial in COVID-19? Medical Hypotheses **140**: 109767-109767.	Study design -narrative review
41	Speeckaert, M. M. and J. R. Delanghe (2020). "Association between low vitamin D and COVID-19: don't forget the vitamin D binding protein." Aging Clinical & Experimental Research **28**: 28.	Study design - letter
42	Skutsch, M., et al. (2020). "The association of UV with rates of COVID-19 transmission and deaths in Mexico: the possible mediating role of vitamin D." MedRxiv : the Preprint Server for Health Sciences. DOI: 10.1101/2020.05.25.20112805	Study design – modelling
43	Suresh, P. S. (2020). "Hypovitaminosis D and COVID-19: Matter of Concern in India?" Indian Journal of Clinical Biochemistry.	Study design - letter
44	Taghizadieh, A., et al. (2020). "Acute kidney injury in pregnant women following SARS-CoV-2 infection: A case report from Iran." Respiratory Medicine Case Reports 30	Exposure – not vitamin D
45	Tan, S. H. S., et al. (2020). "Medications in COVID-19 patients: summarizing the current literature from an orthopaedic perspective." International Orthopaedics **22**: 22.	Study design -narrative review
46	Yalaki, Z., et al. (2019). Comparison of viral agents and vitamin D levels in children with acute bronchiolitis infection. Cocuk Enfeksiyon Dergisi **13**(1): e14-e20.	Population - COVID-19/SARs/MERs not specified
47	Zabetakis, I., et al. (2020). "COVID-19: The Inflammation Link and the Role of Nutrition in Potential Mitigation." Nutrients **12**(5): 19.	Study design -narrative review

For peer review only - http://bmjopen.bmj.com/site/about/guidelines.xhtml

48	Zemb, P., et al. (2020). "Vitamin D deficiency and COVID-19 pandemic." Journal of Global Antimicrobial Resistance **28**: 28.	Study design - commentary

4. Articles included at full text, but later excluded at time of narrative synthesis

	Citation record	Exclusion reason	Update performed 8th October 2020
49	Darling, A. L., et al. (2020). Vitamin D status, body mass index, ethnicity and COVID-19: Initial analysis of the first-reported UK Biobank COVID-19 positive cases (n 580) compared with negative controls (n 723). medRxiv.<br><br>https://www.medrxiv.org/content/10.1101/2020.04.29.20084277v1?versioned=TRUE	Not peer reviewed publication at time of narrative synthesis	No update available
50	De Smet, D., et al. (2020). Vitamin D deficiency as risk factor for severe COVID-19: a convergence of two pandemics. medRxiv.<br><br>https://www.medrxiv.org/content/10.1101/2020.05.01.20079376v2	Not peer reviewed publication at time of narrative synthesis	No update available
51	Lau, F. H., et al. (2020). Vitamin D Insufficiency is Prevalent in Severe COVID-19. medRxiv.<br><br>https://www.medrxiv.org/content/10.1101/2020.04.24.20075838v1?versioned=TRUE	Not peer reviewed publication at time of narrative synthesis	No update available
52	Meltzer, D. O., et al. (2020). "Association of Vitamin D Deficiency and Treatment with COVID-19 Incidence." MedRxiv : the Preprint Server for Health Sciences **13**: 13.	Not peer reviewed public	An updated publication is available at https://jamanetwork.com/journals/jamanetworkopen/fullarticle/2770157<br><br>Citation

For peer review only

	https://www.medrxiv.org/content/10.1101/2020.05.08.20095893v1	ation at time of narrative synthesis	Meltzer DO, Best TJ, Zhang H, Vokes T, Arora V, Solway J. Association of Vitamin D Status and Other Clinical Characteristics With COVID-19 Test Results. *JAMA Netw Open.* 2020;3(9):e2019722. doi:10.1001/jamanetworkopen.2020.19722
53	Notari, A. and G. Torrieri (2020). "COVID-19 transmission risk factors." MedRxiv : the Preprint Server for Health Sciences.<br><br>https://www.medrxiv.org/content/10.1101/2020.05.08.20095083v1?versioned=TRUE	Not peer reviewed publication at time of narrative synthesis	No update available
54	Raisi-Estabragh, Z., et al. (2020). "Greater risk of severe COVID-19 in non-White ethnicities is not explained by cardiometabolic, socioeconomic, or behavioural factors, or by 25(OH)-vitamin D status: study of 1,326 cases from the UK Biobank." MedRxiv : the Preprint Server for Health Sciences.<br><br>https://www.medrxiv.org/content/10.1101/2020.06.01.20118943v1?versioned=TRUE	Not peer reviewed publication at time of narrative synthesis	An updated publication is available at https://academic.oup.com/jpubhealth/article/42/3/451/5859581<br><br>Citation<br>Zahra Raisi-Estabragh, Celeste McCracken, Mae S Bethell, Jackie Cooper, Cyrus Cooper, Mark J Caulfield, Patricia B Munroe, Nicholas C Harvey, Steffen E Petersen, Greater risk of severe COVID-19 in Black, Asian and Minority Ethnic populations is not explained by cardiometabolic, socioeconomic or behavioural factors, or by 25(OH)-vitamin D status: study of 1326 cases from the UK Biobank, *Journal of Public Health*, Volume 42, Issue 3, September 2020, Pages 451–460, https://doi.org/10.1093/pubmed/fdaa095
55	Tan, C. W., et al. (2020). "A cohort study to evaluate the effect of combination Vitamin D, Magnesium and Vitamin B12 (DMB) on progression to severe outcome in older COVID-19 patients." MedRxiv : the Preprint Server for Health Sciences.<br><br>https://www.medrxiv.org/content/10.1101/2020.06.01.20112334v2	Not peer reviewed publication at time of narrative synthesis	No update available

For peer review only - http://bmjopen.bmj.com/site/about/guidelines.xhtml

Study details of the seven articles included at full text, but excluded at time of narrative synthesis						
**Study**	**Design/ setting**	**Population**	**Exposure/Intervention**	**Outcomes**	**Results**	**Limitations**
Darling, A. L., et al. (2020)[1]	Retrospective cohort study<br><br>UK Biobank England cohort only	COVID-19 positive cases (n 580) Mean age 57.5 (SD 9.7)<br><br>COVID-19 negative controls (n 723) Mean age 57.9 (SD 8.7)	Serum 25(OH)D status Median (IQR) nmol/L<br><br>by gender (Male/Female), body mass index (Normal/underweight, overweight, obesity), ethnicity (Asian, Black, Mixed and Other, White)	COVID-19 test result	Serum 25(OH)D status similar in both groups:<br><br>COVID-19 positive cases (median IQR) = 43.3 (32.1) nmol/L)<br><br>COVID-19 negative controls (median (IQR) 44.1 (31.2) nmol/L) for COVID-19.<br><br>A logistic regression model suggests that being overweight (OR 1.51 CI 1.13-2.02) or obese (OR 1.67 CI 1.24-2.26); living in London (OR 1.45 CI 1.05-2.00); being male (OR 1.28 CI 1.01-1.61) and being of Asian, Black or Mixed ethnicity (OR 1.66 CI 1.08-2.54) is associated with a higher odds of testing positive for COVID-19	UK Biobank baseline samples collected in 2006-2010.
De Smet, D., et al. (2020)[2]	Retrospective observational study<br><br>Central network hospital, West Flanders, Belgium	186 SARS-CoV-2 infected patients hospitalised from March 1, 2020 to April 7, 2020<br><br>(109 males [median age 68 years, IQR 53-79] 77 females [median age 71 years, IQR 65-74])<br><br>25(OH)D in COVID-19 patients was compared a control group of 2717 patients with similar age distribution, sampled from March 1, 2019 to April 30, 2019. (999 males [median age 69 years, IQR 53-81] and 1718 females [median age 68 years, IQR 43-83]).	25(OH)D levels	SARS-CoV-2 infection	COVID-19 patients had a lower median 25(OH)D on admission (18.6 ng/mL, IQR 12.6-25.3) than controls (21.5 ng/mL, IQR 13.9-20.8, P=0.0016) and a higher percentage of vitamin D deficiency (defined as 25(OH)D < 20ng/mL): 58.6% versus 45.2% (P=0.0005).<br><br>In male COVID-19 patients, vitamin D deficiency was lower median 25(OH)D (17.6 ng/mL, IQR 12.7-24.0 versus 20.3 ng/mL, IQR 13.7-28.3, P=0.0234) and a higher deficiency rate (67.0% versus 49.2%, P=0.0006) than male controls.	The prevalence and age/sex/ seasonal-distribution of vitamin D status was derived from the general population sampled from 16274 consecutive, unselected and unique patients from January 1, 2019 to December 31, 2019.

For peer review only - http://bmjopen.bmj.com/site/about/guidelines.xhtml

Lau, F. H., et al. (2020)[3]	Retrospective observational study<br><br>A single, tertiary care academic (university) medical centre, Louisiana, New Orleans, USA	COVID-19 ICU patients (n 13) Mean age 61.5 (SD 15.7)<br><br>COVID-19 floor patients (n 7) Mean age 72.0 (SD 14.8)<br><br>Medical records of COVID-19 patients between March 27, 2020 and April 21, 2020<br><br>Vitamin D insufficiency (VDI) and COVID-19 metrics in ICU vs. floor patients	VDI: defined as serum 25(OH) D < 30 ng/mL)<br><br>Serum 25(OH) D status Mean (SD) ng/mL<br><br>by gender (Male/Female), body mass index (Normal, obesity), race (African American), age (elderly>65 years), hypertension	COVID-19 metrics	Overall, few significant differences were identified between ICU and floor patients: Lactate dehydrogenase was significantly higher among ICU patients (441.8 vs. 223.0, P=0.001). Also, body mass index was significantly higher among ICU patients (35.2 vs. 24.5, P=0.02).<br><br>Among ICU subjects, 11 (84.6%) had VDI, vs. 4 (57.1%) of floor subjects. 100% of ICU patients less than 75 years old had VDI (n=11). Among these, 64.6% (n=7) had critically low 25(OH) D (<20 ng/mL) and 3 had <10 ng/mL.<br><br>VDI is highly prevalent in dark-skinned people (82.1% of African Americans vs. 41.6% overall).<br><br>Male/Female ratio was 1.24 and 1.44 for COVID-19 and VDI respectively.	Statistical analysis was limited by the small number of subjects.
Meltzer, D. O., et al. (2020)[4]	Retrospective cohort study<br><br>University of Chicago Medicine, USA	4,314 patients tested for COVID-19 from 3/3/2020 to 4/10/2020. Among these, 499 had a vitamin D level in the year before testing.<br><br>COVID-19 positive cases with vitamin D deficient (n 178) Mean age 45.6<br><br>COVID-19 positive cases with not vitamin D deficient (n 321) Mean age 50.7	Vitamin D deficiency: defined by the most recent 25(OH) D <20ng/ml or 1,25-dihydroxycholecalciferol <18pg/ml within 1 year before COVID-19 testing. Treatment: defined by the most recent vitamin D type and dose, and treatment changes between the time of the most recent vitamin D level and time of COVID-19 testing<br><br>Vitamin D deficiency and treatment changes were combined to categorize vitamin D status at the time of COVID-19 testing as:<br>1)Likely deficient (last-level-deficient/treatment-not-increased)	Testing positive for COVID-19	In multivariable analysis, testing positive for COVID-19 was associated with increasing age (RR (age<50)=1.05, P<0.021; RR (age≥50)=1.02, P<0.064)), non-white race (RR=2.54, P<0.01) and being likely vitamin D deficient (deficient/treatment-not-increased: RR=1.77, P<0.02) as compared to likely vitamin D sufficient (not-deficient/treatment-not-decreased), with predicted COVID-19 rates in the vitamin D deficient group of 21.6% (95%CI [14.0%-29.2%]) vs 12.2% (95%CI [8.9%-15.4%]) in the vitamin D sufficient group.<br><br>Vitamin D deficiency declined with increasing vitamin D dose (especially of vitamin D3). Vitamin D dose was not significantly associated with testing positive for COVID-19 (P=0.18).	The associations observed might not reflect causal effects of vitamin D deficiency on COVID-19. This is because vitamin D deficiency can reflect a range of chronic health conditions or behavioural factors which plausibly decrease the likelihood of treatment of vitamin D

For peer review only - http://bmjopen.bmj.com/site/about/guidelines.xhtml

			2)Likely sufficient (last-level-not-deficient/treatment-not-decreased)<br>3)Uncertain deficiency (last-level-deficient/treatment-increased or last-level-not-deficient/treatment-decreased)<br><br>by age (<50, ≥50), gender (Male/Female), race (White, other than White), ethnicity (Hispanic, not Hispanic), body mass index, employee status, comorbidity indicators (e.g. hypertension)			deficiency and increase COVID-19 risk.
Notari, A. and G. Torrieri (2020)[5]	Correlational study<br><br>126 countries, Only 50 countries for vitamin D	The number of cases follows in its early stages an almost exponential expansion. A starting point in each country was chosen: the first day di with 30 cases and fitted for 12 days. Thus, capturing the early exponential growth.<br><br>Countries with too small total population (less than 300 thousands inhabitants) were excluded.	They analysed risk factors correlated with the initial transmission growth rate of COVID-19<br><br>Average annual level of serum Vitamin D and the seasonal level<br><br>The seasonal level is defined as: the amount during March or during winter for northern hemisphere, or during summer for southern hemisphere or the annual level for countries with little seasonal variation.	Growth rate of COVID-19	They looked for linear correlations of the exponents with other variables, for a sample of 126 countries.<br><br>They found a positive correlation, i.e. faster spread of COVID-19, with high confidence level with the following variables, with respective p-value: low Temperature (4.10^{-7}), high ratio of old vs. working-age people (3.10^{-6}), life expectancy (8.10^{-6}), number of international tourists (1.10^{-5}), earlier epidemic starting date di (2.10^{-5}), high level of physical contact in greeting habits (6.10^{-5}), lung cancer prevalence (6.10^{-5}), obesity in males (1.10^{-4}), share of population in urban areas (2.10^{-4}), cancer prevalence (3.10^{-4}), alcohol consumption (0.0019), daily smoking prevalence (0.0036), UV index (0.004, smaller sample, 73 countries), low Vitamin D serum levels (0.002-0.006, smaller sample, 50 countries). There is highly significant correlation also with blood type. Also, positive correlation with moderate CI (p-value of 0.02-0.03) with: CO2/SO emissions, type-1 diabetes in children, and low vaccination coverage for Tuberculosis (BCG).	The dataset for the annual vitamin D was built with the available literature, which is quite inhomogeneous.<br><br>The dataset for the seasonal levels is more restricted. This is because the relative literature is less complete. So, for this the authors have included only 42 countries.

For peer review only - http://bmjopen.bmj.com/site/about/guidelines.xhtml
For peer review only

					Vitamin D is not highly correlated with UV index due to different food consumption in different countries.	
Raisi-Estabragh, Z., et al. (2020)[6]	Retrospective cohort study<br><br>UK Biobank	4,510 UK participants tested for COVID-19.<br><br>Latest data release (29/05/2020) includes test results from 16/03/2020 to 18/05/2020.<br><br>COVID-19 positive cases (n 1,326) Mean age 68.11 (SD 9.23)<br><br>COVID-19 negative controls (n 3,184) Mean age 68.91 (SD 8.72)	Serum 25(OH) D levels nmol/L<br><br>Multivariate logistic regression models by age, gender (Male/Female), ethnicity (Caucasian (any White background) and non-Caucasian: Black, Asian, Chinese) to test whether addition of: 1)cardio metabolic factors (e.g. hypertension, body mass index); 2) 25(OH)-vitamin D; 3) poor diet; 4) Townsend deprivation score; 5) housing; or 6)behavioural factors attenuated sex/ethnicity associations with COVID-19 status	COVID-19 test result<br><br>Greater risk of severe COVID-19	Over-representation of men and non-White ethnicities in the COVID-19 positive group.<br><br>Non-Whites had, on average, poorer cardio metabolic profile, lower 25(OH)-vitamin D, greater material deprivation, and were more likely to live in larger households and flats.<br><br>Male sex, non-White ethnicity, higher body mass index, Townsend deprivation score, and household overcrowding were independently associated with significantly greater odds of COVID-19. The pattern of association was consistent for men and women; cardio metabolic, socio-demographic and behavioural factors did not attenuate sex/ethnicity associations.	UK Biobank baseline samples collected in 2006-2010.<br><br>Aggregating all Black and Minority Ethnic (BAME) populations into one cohort might overlook important differences between non-Caucasian ethnicities.<br><br>The current dataset does not allow assessment of specific COVID-19 health outcomes.
Tan, C. W., et al. (2020)[7]	Cohort observational study<br><br>A tertiary academic hospital, Singapore	All 43 consecutive hospitalized COVID-19 patients aged 50 and above.<br><br>Between 15 January and 15 April 2020.	DMB = a single daily oral dose of vitamin D3 1000 IU, magnesium 150mg and vitamin B12 500mcg for up to 14 days<br><br>Adjusted for age, gender and comorbidities	Deterioration post-DMB administration leading to any form of oxygen therapy and/or intensive care	Duration of therapy: days, Median 5 (IQR 4-7)<br><br>Significantly fewer DMB patients than controls required initiation of oxygen therapy subsequently throughout their hospitalization (17.6% vs 61.5%, P=0.006).<br><br>On univariate analysis, increasing age and presence of comorbidities were associated	Small sample size, and the lack of systematic biologic measures to support their findings.

For peer review only - http://bmjopen.bmj.com/site/about/guidelines.xhtml

		17 patients received Vitamin D, Magnesium, Vitamin B12 (DMB): Mean age 58.4 (SD 7.0)<br><br>26 patients did not: Mean age 64.1 (SD 7.9)		support for COVID-19 patients	with significantly higher OR for oxygen therapy, while exposure to DMB therapy was associated with a significantly improved OR 0.13 (95% CI: 0.03 – 0.59, P=0.008) .<br><br>On multivariate analysis, increasing age was associated with significantly higher OR for oxygen therapy, while exposure to DMB therapy was associated with a significantly improved OR 0.15 (95% CI: 0.025 – 0.93, P=0.041).	

For peer review only

5. Risk of bias of included studies

Risk of bias assessment using the Downs and Black Checklist[8]

Study	Quality score	Reviewer notes
Hastie et al., 2020[9]	14/20<br><br>Seven domains were not applicable and therefore not assessed, 2 reporting, 1 external validity 3 internal validity (bias) and 1 internal validity (confounding).	The study could not be scored for 3 questions as we were unable to determine; 1) the representativeness of the subjects who were prepared to participate from entire population from which they were recruited, 2) whether losses to follow-up were taken into account as patients lost to follow-up were not reported and 3) whether the study had sufficient power to detect a clinically important effect.<br><br>The study did not score a point for 3 questions; 1) providing the number and a description of the characteristics of patients lost to follow-up, 2) stating whether study subjects in different intervention groups we recruited over the same period of time and 3) for assignment concealment as it was a non-randomised study. The study scored partially (only 1 point not two) for clearly described distributions of principal confounders in each group of subjects to be compared.
D'Avolio et al, 2020[10]	13/15<br><br>Twelve domains were not applicable therefore not assessed, 3 reporting, 1 external validity, 4 internal validity (bias) and 4 internal validity (confounding).	The study could not be scored for the 'power' domain as we were unable to determine from the article whether the study had sufficient power to detect a clinically important effect where the probability value for a difference being due to chance is less than 5%.<br><br>The study did not score 1 point in the 'external validity' domain as those subjects who were prepared to participate were not representative of the entire population from which they were recruited.
Fasano et al., 2020[11]	12/17<br>Ten domains were not applicable therefore not assessed, 3 reporting, 1 external validity, 4 internal validity (bias) and 2 internal validity (confounding).	The study could not be scored for 4 items, the 'power' domain and one question in the 'Internal validity - confounding (selection bias)' as the study did not specify the time period over which patients were recruited. It could also not be scored for 2 questions in the 'External validity domain', 1) the representativeness of the subjects asked to participate in the study compared to the entire population from which they were recruited and 2) the representativeness of those subjects who were prepared to participate compared to the entire population from which they were recruited.<br><br>The study did not score 1 point as the effect of the main confounders was not investigated or confounding was demonstrated but no adjustment was made in the final analyses. The study scored two points for presentation of potential confounders.

For peer review only - http://bmjopen.bmj.com/site/about/guidelines.xhtml

Ilie et al., 2020[12]	4/20<br>Seven domains were not applicable and therefore not assessed, 1 reporting, 1 external validity, 3 internal validity (bias), 1 internal validity (confounding) and 1 for power.	The study could not be scored for 9 questions. Two in the 'reporting' domain, 1) interventions of interest not clearly described, 2) the main findings of the study are not clearly described. Two 'External validity' questions 1) the representativeness of the subjects asked to participate in the study compared to the entire population from which they were recruited and 2) the representativeness of those subjects who were prepared to participate compared to the entire population from which they were recruited. Two 'Internal validity – bias' domain questions 1) all analyses that had not been planned at the outset of the study were not clearly indicated (results of the study based on "data dredging", were not made clear), and 2) it was not clear is the statistical techniques used were appropriate to the data. Three 'Internal validity - confounding (selection bias)' domain questions, 1) no information provided concerning the source of patients included in the study 2) does not specify the time period over which patients were recruited, and 3) the numbers of patients lost to follow-up are not reported.<br><br>The study did not score 7 points for the following; 3 reporting issues 1) no description of the characteristics of participants included in the study 2) no description of the distributions of principal confounders in each group of subjects to be compared, and 3) no description of the characteristics of patients lost to follow-up. Two internal validity bias issues 1) differences in follow-up were ignored and 2) no evidence that the main measure used were accurate (valid and reliable).
**Note:** For each included study, the maximum possible quality score was dependent on which domains could be assessed based on the study design. The higher the score assigned to a study, the lower the risk of bias. For example, Hastie et al. 2020[9] was assigned a score of 14 out of a maximum possible score of 20, suggesting good quality and therefore low risk of bias compared to the other studies.		

For peer review only

References

1. Darling AL, Ahmadi KR, Ward KA, et al. Vitamin D status, body mass index, ethnicity and COVID-19: initial analysis of the first-reported UK Biobank COVID-19 positive cases (n 580) compared with negative controls (n 723). *medRxiv* 2020. doi: 10.1101/2020.04.29.20084277
2. De Smet D, De Smet K, Herroelen P, Gryspeerdt S, Martens GA. Vitamin D deficiency as risk factor for severe COVID-19: a convergence of two pandemics. *medRxiv* 2020. doi: 10.1101/2020.05.01.20079376
3. Lau FH, Majumder R, Torabi R, et al. Vitamin D insufficiency is prevalent in severe COVID-19. *medRxiv* 2020. doi: 10.1101/2020.04.24.20075838

For peer review only - http://bmjopen.bmj.com/site/about/guidelines.xhtml

4. Meltzer DO, Best TJ, Zhang H, Vokes T, Arora V, Solway J. Association of vitamin D deficiency and treatment with COVID-19 incidence. *medRxiv* 2020. doi: 10.1101/2020.05.08.20095893
5. Notari A, Torrieri G. COVID-19 transmission risk factors. *medRxiv* 2020. doi: 10.1101/2020.05.08.20095083
6. Raisi-Estabragh Z, McCracken C, Bethell MS, et al. Greater risk of severe COVID-19 in non-White ethnicities is not explained by cardiometabolic, socioeconomic, or behavioural factors, or by 25(OH)-vitamin D status: study of 1,326 cases from the UK Biobank. *medRxiv* 2020. doi: 10.1101/2020.06.01.20118943
7. Tan CW, Ho LP, Kalimuddin S, et al. A cohort study to evaluate the effect of combination vitamin D, magnesium and vitamin B12 (DMB) on progression to severe outcome in older COVID-19 patients. *medRxiv* 2020. doi: 10.1101/2020.06.01.20112334
8. Downs SH, Black N. The feasibility of creating a checklist for the assessment of the methodological quality both of randomised and non-randomised studies of health care interventions. *J Epidemiol Community Health* 1998;52(6):377-84. doi: 10.1136/jech.52.6.377
9. Hastie CE, Mackay DF, Ho F, et al. Vitamin D concentrations and COVID-19 infection in UK Biobank. *Diabetes Metab Syndr* 2020;14(4):561-65. doi: 10.1016/j.dsx.2020.04.050
10. D'Avolio A, Avataneo V, Manca A, et al. 25-hydroxyvitamin D concentrations are lower in patients with positive PCR for SARS-CoV-2. *Nutrients* 2020;12(5):1359. doi: 10.3390/nu12051359
11. Fasano A, Cereda E, Barichella M, et al. COVID-19 in Parkinson's Disease Patients Living in Lombardy, Italy. *Mov Disord* 2020. doi: 10.1002/mds.28176
12. Ilie PC, Stefanescu S, Smith L. The role of vitamin D in the prevention of coronavirus disease 2019 infection and mortality. *Aging Clin Exp Res* 2020. doi: 10.1007/s40520-020-01570-8

For peer review only

For peer review only - http://bmjopen.bmj.com/site/about/guidelines.xhtml

PRISMA 2009 Checklist

Section/topic	#	Checklist item	Reported on page #
**TITLE**			
Title	1	Identify the report as a systematic review, meta-analysis, or both.	1
**ABSTRACT**			
Structured summary	2	Provide a structured summary including, as applicable: background; objectives; data sources; study eligibility criteria, participants, and interventions; study appraisal and synthesis methods; results; limitations; conclusions and implications of key findings; systematic review registration number.	3
**INTRODUCTION**			
Rationale	3	Describe the rationale for the review in the context of what is already known.	5
Objectives	4	Provide an explicit statement of questions being addressed with reference to participants, interventions, comparisons, outcomes, and study design (PICOS).	6
**METHODS**			
Protocol and registration	5	Indicate if a review protocol exists, if and where it can be accessed (e.g., Web address), and, if available, provide registration information including registration number.	6
Eligibility criteria	6	Specify study characteristics (e.g., PICOS, length of follow-up) and report characteristics (e.g., years considered, language, publication status) used as criteria for eligibility, giving rationale.	7
Information sources	7	Describe all information sources (e.g., databases with dates of coverage, contact with study authors to identify additional studies) in the search and date last searched.	6
Search	8	Present full electronic search strategy for at least one database, including any limits used, such that it could be repeated.	Supp appendix 1
Study selection	9	State the process for selecting studies (i.e., screening, eligibility, included in systematic review, and, if applicable, included in the meta-analysis).	7 and supp appendix 2
Data collection process	10	Describe method of data extraction from reports (e.g., piloted forms, independently, in duplicate) and any processes for obtaining and confirming data from investigators.	8
Data items	11	List and define all variables for which data were sought (e.g., PICOS, funding sources) and any assumptions and simplifications made.	8
Risk of bias in individual studies	12	Describe methods used for assessing risk of bias of individual studies (including specification of whether this was done at the study or outcome level), and how this information is to be used in any data synthesis.	8

For peer review only

For peer review only - http://bmjopen.bmj.com/site/about/guidelines.xhtml

PRISMA 2009 Checklist

Page 1 of 2

Section/topic	#	Checklist item	Reported on page #
Summary measures	13	State the principal summary measures (e.g., risk ratio, difference in means).	8
Synthesis of results	14	Describe the methods of handling data and combining results of studies, if done, including measures of consistency (e.g., I^2) for each meta-analysis.	8
Risk of bias across studies	15	Specify any assessment of risk of bias that may affect the cumulative evidence (e.g., publication bias, selective reporting within studies).	n/a
Additional analyses	16	Describe methods of additional analyses (e.g., sensitivity or subgroup analyses, meta-regression), if done, indicating which were pre-specified.	8
**RESULTS**			
Study selection	17	Give numbers of studies screened, assessed for eligibility, and included in the review, with reasons for exclusions at each stage, ideally with a flow diagram.	8
Study characteristics	18	For each study, present characteristics for which data were extracted (e.g., study size, PICOS, follow-up period) and provide the citations.	8 and supp appendix 3
Risk of bias within studies	19	Present data on risk of bias of each study and, if available, any outcome level assessment (see item 12).	8/9 supp appendix 4
Results of individual studies	20	For all outcomes considered (benefits or harms), present, for each study: (a) simple summary data for each intervention group (b) effect estimates and confidence intervals, ideally with a forest plot.	8/9/10 and supp appendix 3
Synthesis of results	21	Present results of each meta-analysis done, including confidence intervals and measures of consistency.	n/a
Risk of bias across studies	22	Present results of any assessment of risk of bias across studies (see Item 15).	n/a
Additional analysis	23	Give results of additional analyses, if done (e.g., sensitivity or subgroup analyses, meta-regression [see Item 16]).	10
**DISCUSSION**			
Summary of evidence	24	Summarize the main findings including the strength of evidence for each main outcome; consider their relevance to key groups (e.g., healthcare providers, users, and policy makers).	11/12/13
Limitations	25	Discuss limitations at study and outcome level (e.g., risk of bias), and at review-level (e.g., incomplete retrieval of identified research, reporting bias).	13

For peer review only

PRISMA 2009 Checklist

Conclusions	26	Provide a general interpretation of the results in the context of other evidence, and implications for future research.	13/14
**FUNDING**			
Funding	27	Describe sources of funding for the systematic review and other support (e.g., supply of data); role of funders for the systematic review.	15

From: Moher D, Liberati A, Tetzlaff J, Altman DG, The PRISMA Group (2009). Preferred Reporting Items for Systematic Reviews and Meta-Analyses: The PRISMA Statement. PLoS Med 6(7): e1000097. doi:10.1371/journal.pmed1000097

For more information, visit: **www.prisma-statement.org**.

For peer review only

For peer review only - http://bmjopen.bmj.com/site/about/guidelines.xhtml

BMJ Open

Association between vitamin D supplementation or level and susceptibility to COVID-19 infection including clinical course, morbidity and mortality outcomes? A systematic review.

Journal:	*BMJ Open*
Manuscript ID	bmjopen-2020-043737.R2
Article Type:	Original research
Date Submitted by the Author:	09-Jan-2021
Complete List of Authors:	Grove, Amy; Warwick Life Sciences, Division of Health Sciences, Warwick Medical School<br>Osokogu, Osemeke; University of Warwick, Warwick Medical School<br>Al-Khudairy, Lena; University of Warwick Warwick Medical School, Population, evidence and technology<br>Mehrabian, Amin; University of Warwick, Warwick Medical School; University of Medical Sciences, 2) Department of Pharmaceutical Nanotechnology<br>Zanganeh, Mandana; University of Warwick, Warwick Medical School<br>Brown, Anna; University of Warwick, Warwick Medical School<br>Court, Rachel; University of Warwick, Health Sciences<br>Taylor-Phillips, Sian; University of Warwick, Warwick Medical School<br>Uthman, Olalekan; University of Warwick, Warwick-Centre for Applied Health Research (WCAHRD)<br>McCarthy, Noel; University of Warwick, Warwick Medical School<br>Kumar, Sudhesh; University of Warwick, Medical School<br>Clarke, Aileen; University of Warwick, Division of Health Sciences
**Primary Subject Heading**:	Public health
Secondary Subject Heading:	Infectious diseases
Keywords:	COVID-19, Public health < INFECTIOUS DISEASES, NUTRITION & DIETETICS

SCHOLARONE™ Manuscripts

I, the Submitting Author has the right to grant and does grant on behalf of all authors of the Work (as defined in the below author licence), an exclusive licence and/or a non-exclusive licence for contributions from authors who are: i) UK Crown employees; ii) where BMJ has agreed a CC-BY licence shall apply, and/or iii) in accordance with the terms applicable for US Federal Government officers or employees acting as part of their official duties; on a worldwide, perpetual, irrevocable, royalty-free basis to BMJ Publishing Group Ltd ("BMJ") its licensees and where the relevant Journal is co-owned by BMJ to the co-owners of the Journal, to publish the Work in this journal and any other BMJ products and to exploit all rights, as set out in our licence.

The Submitting Author accepts and understands that any supply made under these terms is made by BMJ to the Submitting Author unless you are acting as an employee on behalf of your employer or a postgraduate student of an affiliated institution which is paying any applicable article publishing charge ("APC") for Open Access articles. Where the Submitting Author wishes to make the Work available on an Open Access basis (and intends to pay the relevant APC), the terms of reuse of such Open Access shall be governed by a Creative Commons licence – details of these licences and which Creative Commons licence will apply to this Work are set out in our licence referred to above.

Other than as permitted in any relevant BMJ Author's Self Archiving Policies, I confirm this Work has not been accepted for publication elsewhere, is not being considered for publication elsewhere and does not duplicate material already published. I confirm all authors consent to publication of this Work and authorise the granting of this licence.

For peer review only

Title page

Title

Association between vitamin D supplementation or level and susceptibility to COVID-19 infection including clinical course, morbidity and mortality outcomes? A systematic review.

Author's names

Amy Grove (0000-0002-8027-7274), Osemeke Osokogu, Lena Al-Khudairy, Amin Mehrabian, Mandana Zanganeh, Anna Brown, Rachel Court, Sian Taylor-Phillips, Olalekan Uthman, Noel McCarthy, Sudhesh Kumar, Aileen Clarke (0000-0001-8299-3146).[1]

Address for each author

Author's names and positions

1) Warwick Medical School, University of Warwick, Coventry, CV4 7AL, UK

Dr Amy Grove
Associate Professor of Health Technology Assessment and Implementation Science

Dr Osemeke Osokogu
Research Fellow

Dr Lena Al-Khudairy
Senior Research Fellow

Dr Mandana Zanganeh
Research Fellow

Mrs Anna Brown
Information Specialist

Mrs Rachel Court
Information Specialist

Professor Sian Taylor-Phillips
Professor of Population Health

Professor Olalekan Uthman
Professor of Evidence Synthesis

For peer review only

Professor Noel McCarthy
Professor of Public Health

Professor Sudhesh Kumar
Dean of Medicine

Professor Aileen Clarke
Professor of Public Health and Health Services Research

2) Department of Pharmaceutical Nanotechnology, School of Pharmacy, Mashhad University of Medical Sciences, Mashhad, Iran

Mr Amin Mehrabian
Research Fellow

Corresponding Author

Dr Amy Grove, Room A101, Division of Health Sciences, Warwick Medical School, University of Warwick, Coventry, CV4 7AL
Email: A.L.Grove@warwick.ac.uk

For peer review only

Abstract

Objective: To systemically review and critically appraise published studies of the association between vitamin D supplementation or level and susceptibility to COVID-19 infection, including clinical course, morbidity and mortality outcomes.

Design: Systematic review.

Data sources: MEDLINE (OVID), Embase (OVID), Cochrane Central Register of Controlled Trials, MedRxiv and BioRxiv preprint databases. COVID-19 databases of the WHO, Cochrane, CEBM Oxford, and Bern University up to 10 June 2020.

Study selection: Studies which assessed Vitamin D supplementation and/or Low Serum Vitamin D in patients acutely ill with, or at risk of severe betacoronavirus infection (SARS-CoV, MERS-CoV, SARS-CoV-2).

Data extraction: Two authors independently extracted data using a predefined data extraction form and assessed risk of bias using the Downs and Black Quality Assessment Checklist.

Results: Searches elicited 449 papers, 59 studies were eligible full text assessment and four met the eligibility criteria of this review. The four studies were narratively synthesised and included: 1) a cross-sectional study (n=107) suggesting an inverse association between serum vitamin D and SARS-CoV-2, 2) a retrospective cohort study (348,598 participants, 449 cases) in which univariable analysis showed that vitamin D protects against COVID-19, 3) an ecological country level study demonstrating a negative correlation between vitamin D and COVID-19 case numbers and mortality, and 4) a case-control survey (n=1,486) showing cases with confirmed/probable COVID-19 reported lower vitamin D supplementation . All studies were at high/unclear risk of bias.

Conclusion: There is no robust evidence of a negative association between vitamin D and COVID-19 infection. No relevant randomised control trials were identified and there is no robust peer reviewed published evidence of association between Vitamin D levels and severity of symptoms or mortality due to COVID-19. Guideline producers should acknowledge that benefits of vitamin D supplementation in COVID-19 infection are as yet unproven despite increasing interest from the media and academic community.

For peer review only - http://bmjopen.bmj.com/site/about/guidelines.xhtml

Strengths and limitations of this study

- The strengths of this systematic review include that it is reported in accordance with the Preferred Reporting Items for Systematic Reviews and Meta-Analyses (PRISMA) checklist.
- The review was conducted by two independent reviewers to ensure robustness of this work.
- We searched multiple living systematic review databases to enable us to capture publications in a fast moving field of research.
- The limitations of the study relate to the small amount of evidence available which was, at risk of bias and which limits the inferences that can be drawn.
- The review was restricted to the English language, therefore non English language papers may have been missed.

For peer review only

For peer review only - http://bmjopen.bmj.com/site/about/guidelines.xhtml

Introduction

COVID-19, a novel viral infection caused by Severe Acute Respiratory Syndrome Coronavirus two (SARS-CoV-2) was declared a pandemic by The World Health Organization (WHO) on 11 March 2020.[1] Mild COVID-19 infection may manifest as high temperature, a continuous cough and a loss of or change in sense of smell or taste.[2 3] However, more severe and critical cases can result in inflammation of the lungs, low oxygen levels and acute respiratory distress syndrome.[4] Interest is mounting regarding the association of vitamin D supplementation or level with susceptibility to COVID-19 infection due to the recognised modulating effects of vitamin D on the immune system and immune response.

Vitamin D can modulate the immune system through highly expressed receptors in most non-skeletal tissues.[5 6] Two of the most common analogues of vitamin D which are found in food and used as a dietary supplement are D_2 (ergocalciferol) and D_3 (cholecalciferol, also made by the skin when exposed to sunlight).[7] Both D_2 and D_3 can be hydroxylated by liver enzymes CYP2R1 and CYP27A1 to form calcidiol (25(OH)D). The active metabolite of vitamin D, calcitriol (1α,25(OH)2D), results from the action of CYP27B enzyme on calcidiol. CYP27B is found in several tissues including the kidney, skin, bones, and immune system.[8 9] Tumour necrosis factor α (TNF α) and interferon (IFNγ) are examples of inflammatory cytokines that stimulate the CYP27B enzymes of the immune system.[10-20] Vitamin D can interact with both the innate and cellular immune systems through these mechanisms.

Current Public Health England (PHE),[21] National Institutes of Health[22] and European Food Safety Authority[23] recommendations highlight the importance of vitamin D to population health. Vitamin D deficiency is defined as less than 25 nmol/L (10ng/ml) measured in blood serum.[21] UK guideline recommendations suggest that people take a supplement of 10 micrograms of vitamin D per day during the winter months or throughout the year if they do not spend time outdoors or if they cover the majority of their skin when outside.[21] Published editorials, journal commentaries [24-29] and news media reports [30-32] suggest that individuals with low blood serum concentrations of vitamin D might be at higher risk of infection with COVID-19, or upon infection have worse outcomes than individuals with normal/high serum vitamin D.[33]

Several observational studies have reported associations between low serum vitamin D and chronic[34] and acute conditions such as susceptibility to acute respiratory tract infections (RTI).[35-37] Most recently, Martineau and colleagues (2017) conducted a systematic review and meta-analysis of individual participant data from randomised controlled trials (RCTs) to assess the overall effect of vitamin D supplementation on risk of acute RTI.[38] They reported vitamin D supplementation to be safe while protecting against acute RTI overall (adjusted odds ratio 0.88, 95% confidence interval 0.81 to 0.96; P for heterogeneity <0.001). Patients very deficient in vitamin D benefited the most (adjusted odds ratio 0.75, 0.60 to 0.95; p for

For peer review only - http://bmjopen.bmj.com/site/about/guidelines.xhtml

interaction=0.006).[38] Critiques of this review have suggested that the findings should be interpreted as hypothesis generating only, as the results are heterogeneous and not sufficiently applicable to the general population.[39] Recent rapid reviews of vitamin D for treatment or prevention in COVID-19 reported no evidence that vitamin D deficiency predisposes to COVID-19, or that vitamin D supplementation is effective in prevention or treatment of COVID-19.[40 41] However, data sources included in the rapid review were limited.[42] Given the remaining uncertainty, it is timely to systematically review and critically appraise all peer reviewed published evidence to assess the association of vitamin D supplementation or level with susceptibility to COVID-19 infection including clinical course, morbidity and mortality outcomes.

Methods

Protocol registration

The methods were prespecified in a protocol that was registered with the PROSPERO International Prospective Register of Systematic Reviews (https://www.crd.york.ac.uk/prospero/display_record.php?ID=CRD42020182876). Research ethics committee approval was not required for this study.

We undertook a systematic review to answer the following question: Is vitamin D supplementation or level associated with susceptibility to severe betacoronavirus infection (Severe Acute Respiratory Syndrome [SARS-CoV], Middle East Respiratory Syndrome [MERSCoV], Severe Acute Respiratory Syndrome two [SARS-CoV-2]) including clinical course, morbidity and mortality outcomes?

Our review was conceptualised and written in accordance with the PRISMA statement.[43]

Data sources and search

The search strategy was developed by the information specialists in collaboration with the research team and clinical advisors. We searched MEDLINE (OVID interface), Embase (OVID interface), Cochrane Central Register of Controlled Trials, MedRxiv and BioRxiv preprint databases on 6th-8th May 2020. We searched the global research on COVID-19 developed by the WHO,[44] CEBM Oxford,[45] and the living systematic review developed by Bern University[46] on 10 May 2020. We updated the database searches on 10th June 2020 to capture articles which may have been published since the initial search was conducted.

We searched additional resources including relevant systematic reviews (in MEDLINE [OVID interface], Embase [OVID interface] and Cochrane Database of Systematic Reviews, 19th May 2020), relavent refrences and contacted experts for additional evidence. Our full search record is included in the supplementary information.

For peer review only - http://bmjopen.bmj.com/site/about/guidelines.xhtml

Study eligibility

We developed pre-defined study eligibility criteria aligned to the research question (Table 1). We imposed a date restriction of January 2002, to capture all published articles since SARS-CoV was first discovered in Asia in February 2003.[47] We limited to English language only.

Table 1. Study eligibility criteria

Study eligibility criteria
P - Population<br><br>1) Patients acutely ill with betacoronavirus infection [SARS-CoV, MERS-CoV, SARS-CoV-2]<br>2) or at risk of acute illness with betacoronavirus infection<br><br>I - Intervention/exposure<br><br>1) Vitamin D supplementation<br>2) Low Serum vitamin D<br><br>O - Outcomes<br><br>1) Betacoronavirus infection (to include serological evidence of infection or clinically confirmed symptomatic infection);<br>2) Severe betacoronavirus infection (to include patients admitted to hospital or admitted to intensive care); mortality due to betacoronavirus infection<br>3) Mortality due to betacoronavirus infection<br><br>C – Comparators<br><br>1) No vitamin D supplementation<br>2) High or normal serum vitamin D<br><br>S - Study design<br><br>Peer reviewed publications of randomised controlled trials and non-randomised studies were eligible for inclusion; including, non randomised controlled trials, interrupted time series analyses, controlled before-and-after studies, cohort studies, ecological studies, case reports and case series.<br><br>Subgroups<br><br>1. Ethnicity characteristics (White British, all other White, Mixed, Asian, Black, Other)<br>2. Age characteristics (population by five-year age groups)

Article selection

Following the article search, we systematically identified and removed any duplicate citations using EndNote X9 software. Using titles and abstracts, de-duplicated citations were screened by two independent reviewers (OO, MZ, AM, AG) and checked by a third (AC). All articles deemed ineligible were excluded at this stage. We identified and obtained all remaining articles for full text screening, which was performed independently by at least two reviewers against the pre-specified eligibility criteria (Table 1). Where disagreements regarding the inclusion of articles arose, a third reviewer (AC) was consulted to reach a final decision.

For peer review only

Data extraction

Two reviewers independently (LAK, MZ, OO, AM) extracted data from eligible full-text papers using a prespecified data extraction form. The accuracy of all the data extraction was independently assessed by a third reviewer (AG). Where reported, we sought to extract data from each article relevant to the research question, including details of population, intervention/exposure, comparator, outcomes and any detail related to the two pre-specified subgroups: ethnicity characteristics and age characteristics. Disagreements between reviewers were resolved by discussion and agreement, or via consultation with a third reviewer (AC).

Risk of bias

The included studies had observational study designs aimed at answering a specific question. Therefore, risk of bias of included full-text papers was assessed using the Downs and Black Quality Assessment Checklist.[48] Two reviewers (AM, MZ, OO) independently assessed the risk of bias of the included studies and the accuracy of the assessment was evaluated by a third reviewer (LAK).

Data analysis

We anticipated that identified studies would be too heterogeneous to facilitate pooling of study data and planned a narrative synthesis. Nevertheless, we intended to consider pooling outcomes data in a meta-analysis using a random-effects model if appropriate.

Patient and public involvement

Due to the rapid timeframe of this systematic review it was not possible for our research team to involve patients or the public in the design, conduct, or reporting of our study.

Results

After searching databases, assessing the reference lists of 17 narrative reviews[27 28 33 49-62] and one additional article identified through consultation with clinical experts,[38] we identified 499 citations. Following removal of duplicates and screening of titles and abstracts, we retrieved 59 full-text papers of which four met the full eligibility criteria (see **Error! Reference source not found.**). The electronic supplement includes a list of reasons for excluding studies at full text review. Seven articles closely met the eligibility criteria but were excluded as they were not available as peer reviewed publications at the time of our narrative synthesis, details of these seven studies[63-69] is provided in the electronic supplement.

<Figure 1 approximatley here >

For peer review only - http://bmjopen.bmj.com/site/about/guidelines.xhtml

The charteristics of the four included studies are presented in

For peer review only

For peer review only - http://bmjopen.bmj.com/site/about/guidelines.xhtml

Table 2. All four included studies were conducted in Europe and published in April or May 2020. One study was based on data from UK residents exclusively,[70] another included data on residents in 20 European countries, including the UK.[71] The studies were observational design and no relevant RCT were identified or included in the review. All four studies were at high or unclear risk of bias and scored poorly across several domains of the Downs and Black Quality Assessment Checklist,[48] including external validity, internal validity and power. A prominent issue amongst the included studies was that the authors did not perfom adequate multivariable adjustment to correct for confounding.[72] [73] [74] Ecological bias was present in Ilie et al., 2020[71] which may result from spatial and temporal scale differences between country level mean levels of vitamin D. However, several domains in each risk of bias assessment were not applicable or not reported and therefore, could not be scored using the Downs and Black Quality Assessment Checklist.[48] Detailed risk of bias scores are provided in the electronic supplement.

For peer review only

For peer review only - http://bmjopen.bmj.com/site/about/guidelines.xhtml

Table 2. Characteristics of the four included studies

Study	Design/Setting	Population	Exposure/Intervention	Outcomes	Results	Limitations
**Serum vitamin D**						
D'Avolio et al. 2020[73]	Cross-sectional study<br><br>Canton of Tessin, Switzerland	107 patients with data on SARS-CoV-2 and 25(OH)D measurement	Vitamin D analysis, conducted within seven weeks of the SARS-CoV-2 polymerase chain reaction (PCR) result<br><br>Control patients with 25(OH)D data during the same period	SARS-CoV-2 infection	Group 1 comprised 27 patients with positive PCR test results for SARS-CoV-2 while group 2 comprised 80 patients with a negative PCR result for SARS-CoV-2<br><br>Significantly lower 25(OH)D levels (p = 0.004) in SARS-CoV-2 patients even after stratifying patients according to age >70 years	Few patients from a single hospital<br><br>No available clinical information about the severity of COVID-19 symptoms<br><br>No data on other potential confounding variable<br><br>SARS-CoV-2 and the 25(OH)D status were performed on different days
Hastie et al. 2020[70]	Retrospective cohort study<br><br>UK Biobank Cohort including England, Scotland and Wales	502,624 participants aged 37-73 years between 2006 and 2010	Biochemical assay of 25(OH)D, a measure of vitamin D status<br><br>Vitamin D was imputed if it was below or above the limit of detection	Confirmed COVID-19 infection (at least one positive test result)	Complete data on 348,598 UK Biobank participants<br><br>449 had confirmed COVID-19 infection. Of these, 385 (85.8%) were White compared to 64 (14.2%) non-White (Black, South Asian and others)<br><br>Vitamin D was associated with COVID-19 infection univariably but not after adjustment for confounders. Ethnicity was associated with COVID-19 infection	UK Biobank is not representative of the general population<br><br>Baseline measurements, including 25(OH)D concentration and health status, were obtained a decade prior to conduct of the study

For peer review only - http://bmjopen.bmj.com/site/about/guidelines.xhtml

Ilie et al. 2020[71]	Ecological study<br><br>20 European countries	Population of 20 included European countries	Mean levels of vitamin D in each country	Cases of COVID-19 per 1 million population in each country<br><br>Deaths from COVID-19 per 1 million population	Negative correlations between mean levels of vitamin D and the number of COVID-19 cases per 1 million, and mortality per 1 million	The number of cases per country is affected by the number of tests performed and by the different measures taken by each country to prevent the spread of infection
**Vitamin D supplementation**						
Fasano et al. 2020[74]	Case-control survey<br><br>A single tertiary centre in Lombardy, Italy	1,486 Parkinson's disease (PD) patients were included in the survey<br><br>1,207 family members (controls)	Vitamin D	'Confirmed' or 'probable' diagnosis of COVID-19	12.4% of PD patients with confirmed or probable COVID-19 had been taking vitamin D<br><br>22.9% of PD patients without COVID-19 had been taking vitamin D	Well-known limitation of a telephone survey<br><br>Community-dwelling PD patients<br><br>Some patients could not be reached possibly due to death from COVID-19<br><br>COVID-19 diagnosis could not be confirmed in many cases<br><br>Younger age of non- PD COVID-19 cases

For peer review only

For peer review only - http://bmjopen.bmj.com/site/about/guidelines.xhtml

Serum vitamin D

D'Avolio et al., 2020[73] used a cross sectional design with data on nasopharyngeal swab polymerase chain reaction (PCR) analysis for SARS-CoV-2 and a 25(OH)D measurement taken from patients between 1st March and 14th April 2020. PCR positives (median age = 74 years [IQR 65–81];male = 70.4%) had significantly (P= 0.004) lower serum 25(OH)D levels (median = 11.1 ng/mL [IQR 8.2–21.0]) than PCR negatives (median age = 73 years [IQR 61–82]; male = 48.8%; median 25(OH)D = 24.6 ng/mL [IQR 8.9–30.5]). Although gender and age stratified analysis showed no significant differences, older (>70 years) SARS-CoV-2 positive (n=18) participants had significantly lower median serum 25 (0H) D levels (9.3 ng/mL [IQR 8.1–19.9] than older SARS-CoV-2 negatives (n=43) (23.1 ng/mL [IQR 8.5–31.7]) (P = 0.037).

Hastie et al., 2020[70] is a retrospective cohort study that utilised data from the UK Biobank, [72] using data from 348,598 people with complete information on vitamin D and covariates; 449 people tested positive for COVID-19 infection. COVID-19 positives were older (Median = 49 years; Interquartile Range [IQR] = 40-58) than COVID-19 negatives (Median = 49 years; IQR = 38 - 57) with p-value of <0.05. Multivariable analysis showed that age at assessment (OR = 1.02; 95% CI = 1.00 - 1.03; P= 0.016) and non-White ethnicity (Black OR = 4.30, 95% CI = 2.92 - 6.31, P= < 0.001; South Asian OR = 2.42, 95% CI = 1.50 - 3.93, P= <0.001) were associated with confirmed COVID-19 infection. There was no significant interaction between ethnicity and vitamin D deficiency (OR = 0.90; 95% CI = 0.66 - 1.23; P= 0.515). Median vitamin D concentration at recruitment was lower for people with subsequent confirmed COVID-19 infection (28.7 [IQR 10.0-43.8] nmol/l) than for other participants (32.7 [IQR 10.0-47.2] nmol/l) (P= <0.01). Although univariable analysis suggested an association between vitamin D and COVID-19 (OR=0.99; 95% CI 0.99 – 0.999; P=0.013), this association became insignificant (OR=1.00; 95% CI = 0.998-1.01; P=0.208) after adjustment for covariates.[70]

Ilie et al., 2020[71] used an ecological study design reporting on 20 European countries as at 8th April 2020; the data pertains to mean levels of vitamin D, cases of COVID-19 infection per million population and deaths from COVID-19 per million population. The authors performed Pearson Correlation Coefficient Calculations and reported a negative correlation between mean levels of vitamin D (Mean 56.79 nmol/l, SD 10.61) and numbers of cases of COVID-19 infection per million population in each country (Mean cases 1393.4, SD 1129.984, $r(20) = -0.44$; P = 0.05). Additionally, a negative correlation was reported between mean vitamin D levels and the number of deaths caused by COVID-19 per million population in each country (Mean 80.42, SD 94.61, $r(20) = -0.4378$; P = 0.05). Sweden had the highest mean level of vitamin D (73.5 nmol/l) compared to Spain which had a mean level of 42.5 nmol/l). The number of cases of COVID-19 per million population was 834 in Sweden and 3,137 in Spain. Likewise, at the time of the study, there were 68 deaths from COVID-19 per million population in Sweden and 314 in Spain.

For peer review only - http://bmjopen.bmj.com/site/about/guidelines.xhtml

Vitamin D supplementation

Fasano et al., 2020[74] investigated patients in a case-control phone survey in Lombardy, Italy. COVID-19 diagnosis was confirmed using a nasopharyngeal swab or probable based on : a) presence of persistent COVID-19-related symptoms (≥3 including fever or ≥5 without fever); or b) ≥1 symptom in presence of suggestive chest radiologic signs; and/or c) living with a family member with a confirmed diagnosis of COVID-19. 1,486 participants were included in the survey (32 confirmed COVID-19, 73 probable COVID-19 and 1,381 unaffected). Confirmed/probable COVID-19 cases (mean age = 70.5 [Standard Deviation [SD] = 10.1]; male = 53%) self- reported a significantly lower intake of vitamin D supplementation (12.4%) compared to unaffected cases (22.9%; mean age = 73.0 [SD = 9.5], male = 57%). The age-adjusted OR (OR 0.56 [95% CI = 0.32-0.99], P= 0.048) suggested a protective effect of vitamin D intake.

Subgroup evaluation

We planned to perform subgroup analyses by age and ethnicity. According to Hastie et al., 2020[70] multivariable analysis showed that age at assessment (OR = 1.02; 95% CI = 1.00 - 1.03; P= 0.016) and non-White ethnicity (Black OR = 4.30, 95% CI = 2.92 - 6.31, P= < 0.001; South Asians OR = 2.42, 95% CI = 1.50 - 3.93, P= <0.001) were associated with confirmed COVID-19 infection. However, Hastie et al found no significant interaction between ethnicity and vitamin D deficiency (OR = 0.90; 95% CI = 0.66 - 1.23; P= 0.515).

Discussion

This systematic review of non-randomised studies has shown no robust evidence of a association between vitamin D and COVID-19 infection. We identified four studies for inclusion in a narrative synthesis which were all at high or unclear risk of bias. A univariable analysis of data from the UK Biobank database revealed an association between vitamin D and COVID-19 infection (OR=0.99; 95% CI 0.99 – 0.999; P=0.013). However, this association became insignificant (OR=1.00; 95% CI=0.998-1.01; P=0.208) after adjustment for 13 other covariates, suggesting that the initial association was due to one or more confounding variables.[70]This view is further strengthened by the demonstration of highly significant associations between age and ethnicity characteristics as predictor variables, and COVID-19 infection as the outcome variable. Overall, the UK Biobank study showed no effect, however it should be noted that the UK Biobank data included only one measurement of Vitamin D levels taken between 10 and 14 years prior to the outbreak of COVID-19. This is a significant study limitation.

Liu et al., 2020[75] concluded that patients over 60 years experienced more severe manifestations and had longer disease courses of COVID-19 compared to patients below 60 years.[75] And other studies have shown that older (rather than younger) people are more likely to die from COVID-19 infection.[76-79]

For peer review only

Non-White people are known to be more susceptible to COVID-19 infection and tend to develop worse outcomes,[80] a finding that our review has further substantiated.[70] Ethnicity is a multi-faceted construct that includes genetic make-up, socio-cultural identity and behavioural patterns.[81] It has been shown to be associated with differing susceptibility and treatment outcomes in a number of diseases.[82 83 84] Hastie et al., 2020[#ref} did not find any interaction between ethnicity and vitamin D deficiency and although Ilie et al 2020[71] identified a relationship, the study is subject to ecological bias. Ilie et al 2020[71] compared vitamin D levels and rates of COVID-19 infection aross 20 European countries, and therefore many relevant factors were not accounted for in the analysis. Given the findings so far from our review we consider that there is paucity of data on vitamin D levels and morbidity and mortality from COVID-19 and there is no evidence from RCTs on outcomes of vitamin D supplementation on severity of symptoms or mortality to date. However a relationship between ethnicity, vitamin D (serum levels or supplementation) and susceptibility to or severity of COVID-19 infection cannot yet be ruled out.

Risk of bias assessments demonstrate that all studies were at high or unclear risk of bias. All studies were observational designs and therefore subject to confounding. The persistent calls for high-dose vitamin D supplementation[85] arise from speculation about presumed mechanisms.[86 87] Our systematic review found no robust evidence that low levels of Vitamin D are associated with an increased likelihood of COVID-19 infection. More robust prognostic studies could be combined in a systematic review where a prognostic factor research question is phrased, and considerations of participation, attrition, prognostic factor measurement, confounding measurement and account, outcome measurement, and analysis and reporting are evaluated.

Our systematic review identified no relevant RCTs, nevertheless we are aware of two ongoing RCTs investigating the effects of vitamin D on COVID-19, the ZnD3-CoVici study, France (NCT04351490)[88] and the CoVitTrial, France (NCT04344041).[89] Both trials have an estimated study completion date of July 2020. Inclusion of data from these studies in future systematic reviews and meta-analyses may enable us to potentially draw better stronger conclusions on this topic. Results from the ongoing international VITDALIZE Study (NCT03188796) may also contribute to our understanding of the effect of high dose vitamin D3 on mortality.[90]

Study limitations

We performed a full systematic review of the published evidence available, and simultaneous independent screening, data extraction and risk of bias assessments. However, our study is limited by the small amount of evidence available which was, moreover, at risk of bias. This limits the inferences that can be drawn. Seven eligible studies were excluded because they are not available as peer reviewed publications.[63-69] If published, these seven studies would be included in a future update of this review. A final limitation is that the review was restricted

For peer review only - http://bmjopen.bmj.com/site/about/guidelines.xhtml

to English language only. Therefore, articles published in other languages may have been excluded.

Implications for practice

Our review does not provide evidence for or against additional or high dose vitamin D supplementation specifically in relation to COVID-19. Treatment as standard practice for people who are deficient is pre-existing practice across Europe[23] the US[91] and in the UK.[21] Current guidelines from PHE suggest that the entire UK population should take vitamin D supplements to prevent vitamin D deficiency in winter or with inadequate sunlight exposure to sun in summer.[21] This review does not give evidence to drive a change in this current advice. Treatment recommendations for patients should be updated following the publication of results from ongoing and new well designed adequately powered randomised controlled trials

Conclusion

This systematic review identified no robust evidence to enable us to assess an association between vitamin D supplementation or level with susceptibility to COVID-19 infection including clinical course, morbidity and mortality outcomes. All studies were at high or unclear risk of bias. Both age and ethnicity were associated with vitamin D levels even after multivariable adjustment. Black and South Asian people had a much higher risk of confirmed COVID-19 compared to White people. However, there was no interaction between the association of ethnicity and vitamin D deficiency with COVID-19. There were no papers reporting association of vitamin D with severity of symptoms or mortality due to COVID-19.

For peer review only

Acknowledgements:
We thank Mrs Kath Charters, Welsh Ambulance Services NHS Trust for comments on the study design and drafts of this manuscript and for valuable advice and helpful discussions.

Contributorship statement:
SK, AG and AC conceived the study. AG, AC, NMcC, SK, STP and OU designed the study. RC and AB developed the search stratergies, performed all searches and database management and created the bibliography. AG, AC, AM, OO, MZ screened titles and abstracts for inclusion. AG, OO, AM, MZ, LAK, AC screened at full text and extracted and analysed data. OO, AM, MZ, LAK performed risk of bias assessments. AC, SK and NMcC assisted in the interpretation from a clinical perspective. STP, LAK, OU offered technical and methodological support. AG and OO wrote the first draft, all authors revised content. All authors approved the final manuscript. AG and AC are the guarantors. The corresponding author attests that all listed authors meet authorship criteria and that no others meeting the criteria have been omitted.

Copyright/license for publication:
The Corresponding Author has the right to grant on behalf of all authors and does grant on behalf of all authors, a worldwide licence to the Publishers and its licensees in perpetuity, in all forms, formats and media (whether known now or created in the future), to i) publish, reproduce, distribute, display and store the Contribution, ii) translate the Contribution into other languages, create adaptations, reprints, include within collections and create summaries, extracts and/or, abstracts of the Contribution, iii) create any other derivative work(s) based on the Contribution, iv) to exploit all subsidiary rights in the Contribution, v) the inclusion of electronic links from the Contribution to third party material where-ever it may be located; and, vi) licence any third party to do any or all of the above.

Funding:
Dr Amy Grove is supported by the National Institute for Health Research (NIHR) Advanced Fellowship Programme Reference (AF-300060). Dr Taylor-Phillips is supported by an NIHR Career Development Fellowship (CDF-2016-09-018). Prof Clarke and Dr Al-Khudairy are supported by the NIHR Applied Research Centre West Midlands (ARC-WM). All other authors, with the exception of Professor Uthman, Professor Kumar and Professor McCarthy, are supported by the NIHR, Evidence Synthesis Programme (HTA 14.25.05). The views expressed are those of the authors and not necessarily those of the NIHR or the Department of Health and Social Care.

Competing interests:
All authors have completed the ICMJE uniform disclosure form at www.icmje.org/coi_disclosure.pdf and declare: no support from any organisation for the submitted work; no competing interests with regards to the submitted work.

For peer review only

Ethical statement:

Not required

Data sharing statement:

The study protocol is available

Systematic review protocol registration: CRD42020182876 available online via PROSPERO at https://www.crd.york.ac.uk/prospero/display_record.php?ID=CRD42020182876. All included studies are publicly available. Additional data are available upon reasonable request by emailing the corresponding author.

For peer review only

References

1. World Health Organization. Coronavirus disease (COVID-19) pandemic. 2020. https://www.who.int/emergencies/diseases/novel-coronavirus-2019
2. NHS. Check if you or your child has coronavirus symptoms. 2020. https://www.nhs.uk/conditions/coronavirus-covid-19/symptoms/#symptoms
3. Argenziano MG, Bruce SL, Slater CL, et al. Characterization and clinical course of 1000 patients with coronavirus disease 2019 in New York: retrospective case series. *BMJ* 2020;369:m1996. doi: 10.1136/bmj.m1996
4. Matthay MA, Aldrich JM, Gotts JE. Treatment for severe acute respiratory distress syndrome from COVID-19. *Lancet Respir Med* 2020;8(5):433-34. doi: 10.1016/s2213-2600(20)30127-2
5. Gruber BM. Fenomen witaminy D [The phenomenon of vitamin D]. *Postepy Hig Med Dosw (Online)* 2015;69:127-39
6. Gruber-Bzura BM. Vitamin D and influenza: prevention or therapy? *Int J Mol Sci* 2018;19(8):2419. doi: 10.3390/ijms19082419
7. Holick MF. High prevalence of vitamin D inadequacy and implications for health. *Mayo Clin Proc* 2006;81(3):353-73. doi: 10.4065/81.3.353
8. Sigmundsdottir H, Pan J, Debes GF, et al. DCs metabolize sunlight-induced vitamin D3 to 'program' T cell attraction to the epidermal chemokine CCL27. *Nat Immunol* 2007;8(3):285-93. doi: 10.1038/ni1433
9. Kogawa M, Findlay DM, Anderson PH, et al. Osteoclastic metabolism of 25(OH)-vitamin D3: a potential mechanism for optimization of bone resorption. *Endocrinology* 2010;151(10):4613-25. doi: 10.1210/en.2010-0334
10. Jones G. Vitamin D safety: its mechanisms and application. *Standardy Medyczne, Pediatria* 2012;9:605-09
11. Kim D. The role of vitamin D in thyroid diseases. *Int J Mol Sci* 2017;18(9):1949. doi: 10.3390/ijms18091949
12. Beard JA, Bearden A, Striker R. Vitamin D and the anti-viral state. *J Clin Virol* 2011;50(3):194-200. doi: 10.1016/j.jcv.2010.12.006
13. Liu PT, Stenger S, Li H, et al. Toll-like receptor triggering of a vitamin D-mediated human antimicrobial response. *Science* 2006;311(5768):1770-3. doi: 10.1126/science.1123933
14. Adams JS, Ren S, Liu PT, et al. Vitamin D-directed rheostatic regulation of monocyte antibacterial responses. *J Immunol* 2009;182(7):4289-95. doi: 10.4049/jimmunol.0803736
15. Laaksi I. Vitamin D and respiratory infection in adults. *Proc Nutr Soc* 2012;71(1):90-7. doi: 10.1017/s0029665111003351
16. Sharifi A, Vahedi H, Nedjat S, Rafiei H, Hosseinzadeh-Attar MJ. Effect of single-dose injection of vitamin D on immune cytokines in ulcerative colitis patients: a randomized placebo-controlled trial. *APMIS* 2019;127(10):681-87. doi: 10.1111/apm.12982
17. Cantorna MT, Snyder L, Lin YD, Yang L. Vitamin D and 1,25(OH)2D regulation of T cells. *Nutrients* 2015;7(4):3011-21. doi: 10.3390/nu7043011
18. Jeffery LE, Burke F, Mura M, et al. 1,25-Dihydroxyvitamin D3 and IL-2 combine to inhibit T cell production of inflammatory cytokines and promote development of regulatory

For peer review only - http://bmjopen.bmj.com/site/about/guidelines.xhtml

T cells expressing CTLA-4 and FoxP3. *J Immunol* 2009;183(9):5458-67. doi: 10.4049/jimmunol.0803217
19. Lei GS, Zhang C, Cheng BH, Lee CH. Mechanisms of action of vitamin D as supplemental therapy for pneumocystis pneumonia. *Antimicrob Agents Chemother* 2017;61(10):e01226-17. doi: 10.1128/aac.01226-17
20. Colunga Biancatelli RML, Berrill M, Marik PE. The antiviral properties of vitamin C. *Expert Rev Anti Infect Ther* 2020;18(2):99-101. doi: 10.1080/14787210.2020.1706483
21. Scientific Advisory Committee on Nutrition. SACN vitamin D and health report. 21 July 2016. https://www.gov.uk/government/publications/sacn-vitamin-d-and-health-report
22. National Institutes of Health, Office of Dietary Supplements. Vitamin D: fact sheet for health professionals. 24 March 2020. https://ods.od.nih.gov/factsheets/VitaminD-HealthProfessional/#h2
23. EFSA Panel on Dietetic Products, Nutrition and Allergies. Dietary reference values for vitamin D. *EFSA Journal* 2016;14(10):e04547. doi: 10.2903/j.efsa.2016.4547
24. Cao Z, Wu Y, Faucon E, Sabatier JM. SARS-CoV-2 & Covid-19: key-roles of the 'renin-angiotensin' system / vitamin D impacting drug and vaccine developments. *Infect Disord Drug Targets* 2020;20(1). doi: 10.2174/1871526520999200505174704
25. Jakovac H. COVID-19 and vitamin D: is there a link and an opportunity for intervention? *Am J Physiol Endocrinol Metab* 2020;318(5):E589. doi: 10.1152/ajpendo.00138.2020
26. Mitchell F. Vitamin-D and COVID-19: do deficient risk a poorer outcome? *Lancet Diabetes Endocrinol* 2020;8(7):570. doi: 10.1016/S2213-8587(20)30183-2
27. Silberstein M. Vitamin D: a simpler alternative to tocilizumab for trial in COVID-19? *Med Hypotheses* 2020;140:109767. doi: 10.1016/j.mehy.2020.109767
28. Calder PC, Carr AC, Gombart AF, Eggersdorfer M. Optimal nutritional status for a well-functioning immune system is an important factor to protect against viral infections. *Nutrients* 2020;12(4):1181. doi: https://dx.doi.org/10.3390/nu12041181
29. Tian Y, Rong L. Letter: Covid-19, and vitamin D. Authors' reply. *Aliment Pharmacol Ther* 2020;51(10):995-96. doi: 10.1111/apt.15764
30. Busby M. UK public health bodies reviewing vitamin D's effects on coronavirus. The Guardian; 17 June 2020. https://www.theguardian.com/world/2020/jun/17/uk-ministers-order-urgent-vitamin-d-coronavirus-review
31. Coronavirus: Should I start taking vitamin D? BBC News; 18 June 2020. https://www.bbc.co.uk/news/health-52371688
32. Torjesen I. Covid-19: Public health agencies review whether vitamin D supplements could reduce risk. *BMJ* 2020;369:m2475. doi: 10.1136/bmj.m2475
33. Grant WB, Lahore H, McDonnell SL, et al. Evidence that vitamin D supplementation could reduce risk of influenza and COVID-19 infections and deaths. *Nutrients* 2020;12(4):988. doi: 10.3390/nu12040988
34. Parker J, Hashmi O, Dutton D, et al. Levels of vitamin D and cardiometabolic disorders: systematic review and meta-analysis. *Maturitas* 2010;65(3):225-36. doi: 10.1016/j.maturitas.2009.12.013
35. Cannell JJ, Vieth R, Umhau JC, et al. Epidemic influenza and vitamin D. *Epidemiol Infect* 2006;134(6):1129-40. doi: 10.1017/s0950268806007175
36. Jolliffe DA, Griffiths CJ, Martineau AR. Vitamin D in the prevention of acute respiratory infection: systematic review of clinical studies. *J Steroid Biochem Mol Biol* 2013;136:321-9. doi: 10.1016/j.jsbmb.2012.11.017

For peer review only - http://bmjopen.bmj.com/site/about/guidelines.xhtml

37. Nnoaham KE, Clarke A. Low serum vitamin D levels and tuberculosis: a systematic review and meta-analysis. *Int J Epidemiol* 2008;37(1):113-19. doi: 10.1093/ije/dym247
38. Martineau AR, Jolliffe DA, Hooper RL, et al. Vitamin D supplementation to prevent acute respiratory tract infections: systematic review and meta-analysis of individual participant data. *BMJ* 2017;356:i6583. doi: 10.1136/bmj.i6583
39. Bolland MJ, Avenell A. Do Vitamin D supplements help prevent respiratory tract infections? *BMJ* 2017;356:j456. doi: 10.1136/bmj.j456
40. National Institute for Health and Care Excellence. COVID-19 rapid evidence summary: vitamin D for COVID-19. 29 June 2020. https://www.nice.org.uk/advice/es28/chapter/Key-messages
41. Royal Society. Vitamin D and covid-19. 18 June 2020. https://royalsociety.org/-/media/policy/projects/set-c/set-c-vitamin-d-and-covid-19.pdf
42. Lee J, van Hecke O, Roberts N, on behalf of the Oxford COVID-19 Evidence Service Team. Vitamin D: a rapid review of the evidence for treatment or prevention in COVID-19. Centre for Evidence-Based Medicine, University of Oxford; 1 May 2020. https://www.cebm.net/covid-19/vitamin-d-a-rapid-review-of-the-evidence-for-treatment-or-prevention-in-covid-19/
43. Moher D, Liberati A, Tetzlaff J, Altman DG. Preferred reporting items for systematic reviews and meta-analyses: the PRISMA statement. *BMJ* 2009;339:b2535. doi: 10.1136/bmj.b2535
44. World Health Organization. Global research on coronavirus disease (COVID-19) [online database]. 2020. https://www.who.int/emergencies/diseases/novel-coronavirus-2019/global-research-on-novel-coronavirus-2019-ncov
45. Oxford COVID-19 Evidence Service. Centre for Evidence-Based Medicine, University of Oxford; 2020. https://www.cebm.net/oxford-covid-19-evidence-service/
46. Counotte M, Imeri H, Ipekci M, Low N. Living evidence on COVID-19 [online database]. University of Bern Institute of Social and Preventive Medicine; 2020. https://zika.ispm.unibe.ch/assets/data/pub/ncov/
47. Centers for Disease Control and Prevention. CDC SARS response timeline. 2013. https://www.cdc.gov/about/history/sars/timeline.htm
48. Downs SH, Black N. The feasibility of creating a checklist for the assessment of the methodological quality both of randomised and non-randomised studies of health care interventions. *J Epidemiol Community Health* 1998;52(6):377-84. doi: 10.1136/jech.52.6.377
49. Carter SJ, Baranauskas MN, Fly AD. Considerations for obesity, vitamin D, and physical activity amidst the COVID-19 pandemic. *Obesity (Silver Spring)* 2020;28:1176-77. doi: 10.1002/oby.22838
50. Molloy EJ, Murphy N. Vitamin D, covid-19 and children. *Ir Med J* 2020;113(4):64
51. McCartney DM, Byrne DG. Optimisation of vitamin D status for enhanced immuno-protection against covid-19. *Ir Med J* 2020;113(4):P58
52. Adams KK, Baker WL, Sobieraj DM. Myth busters: dietary supplements and COVID-19. *Ann Pharmacother* 2020;54(8):820-26. doi: 10.1177/1060028020928052
53. de Lucena TMC, da Silva Santos AF, de Lima BR, de Albuquerque Borborema ME, de Azevedo Silva J. Mechanism of inflammatory response in associated comorbidities in COVID-19. *Diabetes Metab Syndr* 2020;14(4):597-600. doi: 10.1016/j.dsx.2020.05.025

For peer review only - http://bmjopen.bmj.com/site/about/guidelines.xhtml

54. Ghasemian R, Shamshirian A, Heydari K, et al. The role of vitamin D in the age of COVID-19: a systematic review and meta-analysis along with an ecological approach. *medRxiv* 2020. doi: 10.1101/2020.06.05.20123554
55. Hribar CA, Cobbold PH, Church FC. Potential role of vitamin D in the elderly to resist COVID-19 and to slow progression of Parkinson's disease. *Brain Sci* 2020;10(5):284. doi: 10.3390/brainsci10050284
56. Laird E, Rhodes J, Kenny RA. Vitamin D and inflammation: potential implications for severity of Covid-19. *Ir Med J* 2020;113(5):P81
57. McKenna MJ, Flynn MAT. Covid-19, cocooning and vitamin D intake requirements. *Ir Med J* 2020;113(5):P79
58. Ribeiro H, Santana KVS, Oliver SL, et al. Does vitamin D play a role in the management of Covid-19 in Brazil? *Rev Saude Publica* 2020;54:53
59. Speeckaert MM, Delanghe JR. Association between low vitamin D and COVID-19: don't forget the vitamin D binding protein. *Aging Clin Exp Res* 2020. doi: 10.1007/s40520-020-01607-y
60. Suresh PS. Hypovitaminosis D and COVID-19: matter of concern in India? *Indian J Clin Biochem* 2020. doi: 10.1007/s12291-020-00894-6
61. Tan SHS, Hong CC, Saha S, Murphy D, Hui JH. Medications in COVID-19 patients: summarizing the current literature from an orthopaedic perspective. *Int Orthop* 2020. doi: 10.1007/s00264-020-04643-5
62. Zabetakis I, Lordan R, Norton C, Tsoupras A. COVID-19: the inflammation link and the role of nutrition in potential mitigation. *Nutrients* 2020;12(5):1466. doi: 10.3390/nu12051466
63. Lau FH, Majumder R, Torabi R, et al. Vitamin D insufficiency is prevalent in severe COVID-19. *medRxiv* 2020. doi: 10.1101/2020.04.24.20075838
64. Darling AL, Ahmadi KR, Ward KA, et al. Vitamin D status, body mass index, ethnicity and COVID-19: initial analysis of the first-reported UK Biobank COVID-19 positive cases (n 580) compared with negative controls (n 723). *medRxiv* 2020. doi: 10.1101/2020.04.29.20084277
65. De Smet D, De Smet K, Herroelen P, Gryspeerdt S, Martens GA. Vitamin D deficiency as risk factor for severe COVID-19: a convergence of two pandemics. *medRxiv* 2020. doi: 10.1101/2020.05.01.20079376
66. Meltzer DO, Best TJ, Zhang H, Vokes T, Arora V, Solway J. Association of vitamin D deficiency and treatment with COVID-19 incidence. *medRxiv* 2020. doi: 10.1101/2020.05.08.20095893
67. Notari A, Torrieri G. COVID-19 transmission risk factors. *medRxiv* 2020. doi: 10.1101/2020.05.08.20095083
68. Raisi-Estabragh Z, McCracken C, Bethell MS, et al. Greater risk of severe COVID-19 in non-White ethnicities is not explained by cardiometabolic, socioeconomic, or behavioural factors, or by 25(OH)-vitamin D status: study of 1,326 cases from the UK Biobank. *medRxiv* 2020. doi: 10.1101/2020.06.01.20118943
69. Tan CW, Ho LP, Kalimuddin S, et al. A cohort study to evaluate the effect of combination vitamin D, magnesium and vitamin B12 (DMB) on progression to severe outcome in older COVID-19 patients. *medRxiv* 2020. doi: 10.1101/2020.06.01.20112334
70. Hastie CE, Mackay DF, Ho F, et al. Vitamin D concentrations and COVID-19 infection in UK Biobank. *Diabetes Metab Syndr* 2020;14(4):561-65. doi: 10.1016/j.dsx.2020.04.050

For peer review only - http://bmjopen.bmj.com/site/about/guidelines.xhtml

71. Ilie PC, Stefanescu S, Smith L. The role of vitamin D in the prevention of coronavirus disease 2019 infection and mortality. *Aging Clin Exp Res* 2020. doi: 10.1007/s40520-020-01570-8
72. UK Biobank. About UK Biobank. 17 June 2020. https://www.ukbiobank.ac.uk/about-biobank-uk/
73. D'Avolio A, Avataneo V, Manca A, et al. 25-hydroxyvitamin D concentrations are lower in patients with positive PCR for SARS-CoV-2. *Nutrients* 2020;12(5):1359. doi: 10.3390/nu12051359
74. Fasano A, Cereda E, Barichella M, et al. COVID-19 in Parkinson's Disease Patients Living in Lombardy, Italy. *Mov Disord* 2020. doi: 10.1002/mds.28176
75. Liu Y, Mao B, Liang S, et al. Association between age and clinical characteristics and outcomes of COVID-19. *Eur Respir J* 2020;55(5):2001112. doi: 10.1183/13993003.01112-2020
76. Verity R, Okell LC, Dorigatti I, et al. Estimates of the severity of coronavirus disease 2019: a model-based analysis. *Lancet Infect Dis* 2020;20(6):669-77. doi: 10.1016/s1473-3099(20)30243-7
77. Mahase E. Covid-19: death rate is 0.66% and increases with age, study estimates. *BMJ* 2020;369:m1327. doi: 10.1136/bmj.m1327
78. Fulzele S, Sahay B, Yusufu I, et al. COVID-19 virulence in aged patients might be impacted by the host cellular microRNAs abundance/profile. *Aging Dis* 2020;11(3):509-22. doi: 10.14336/ad.2020.0428
79. Koff WC, Williams MA. Covid-19 and immunity in aging populations: a new research agenda. *N Engl J Med* 2020. doi: 10.1056/NEJMp2006761
80. Pan D, Sze S, Minhas JS, et al. The impact of ethnicity on clinical outcomes in COVID-19: a systematic review. *EClinicalMedicine*. doi: 10.1016/j.eclinm.2020.100404
81. Lee C. "Race" and "ethnicity" in biomedical research: how do scientists construct and explain differences in health? *Soc Sci Med* 2009;68(6):1183-90. doi: 10.1016/j.socscimed.2008.12.036
82. Stead WW, Senner JW, Reddick WT, Lofgren JP. Racial differences in susceptibility to infection by Mycobacterium tuberculosis. *N Engl J Med* 1990;322(7):422-7. doi: 10.1056/nejm199002153220702
83. Nahid P, Horne DJ, Jarlsberg LG, et al. Racial differences in tuberculosis infection in United States communities: the coronary artery risk development in young adults study. *Clin Infect Dis* 2011;53(3):291-4. doi: 10.1093/cid/cir378
84. Bime C, Poongkunran C, Borgstrom M, et al. Racial differences in mortality from severe acute respiratory failure in the United States, 2008-2012. *Ann Am Thorac Soc* 2016;13(12):2184-89. doi: 10.1513/AnnalsATS.201605-359OC
85. Lanham-New SA, Webb AR, Cashman KD, et al. Vitamin D and SARS-CoV-2 virus/COVID-19 disease. *BMJ Nutrition, Prevention & Health* 2020:bmjnph-2020-000089. doi: 10.1136/bmjnph-2020-000089
86. Huang F, Zhang C, Liu Q, et al. Identification of amitriptyline HCl, flavin adenine dinucleotide, azacitidine and calcitriol as repurposing drugs for influenza A H5N1 virus-induced lung injury. *PLoS Pathog* 2020;16(3):e1008341. doi: 10.1371/journal.ppat.1008341
87. Lee C. Controversial effects of vitamin D and related genes on viral infections, pathogenesis, and treatment outcomes. *Nutrients* 2020;12(4):962. doi: 10.3390/nu12040962

For peer review only - http://bmjopen.bmj.com/site/about/guidelines.xhtml

88. Impact of zinc and vitamin D3 supplementation on the survival of aged patients infected with COVID-19. NCT04351490. ClinicalTrials.gov, U.S. National Library of Medicine; 2020. https://ClinicalTrials.gov/show/NCT04351490
89. COvid-19 and Vitamin D supplementation: a multicenter randomized controlled trial of high dose versus standard dose vitamin D3 in high-risk COVID-19 Patients (CoVitTrial). NCT04344041. ClinicalTrials.gov, U.S. National Library of Medicine; 2020. https://clinicaltrials.gov/show/NCT04344041
90. The VITDALIZE Study: effect of high-dose vitamin D3 on 28-day mortality in adult critically ill patients. NCT03188796. ClinicalTrials.gov, U.S. National Library of Medicine; 2017. https://ClinicalTrials.gov/show/NCT03188796
91. Scientific Advisory Committee on Nutrition, European Food Safety Authority. Joint explanatory note by the European Food Safety Authority and the UK Scientific Advisory Committee on Nutrition regarding dietary reference values for vitamin D. 2016. https://www.efsa.europa.eu/sites/default/files/documents/news/explanatory_note_EFSA_SACN_vitaminD.pdf

Figure legends

Figure 1. PRISMA flow diagram for the selection of studies

For peer review only

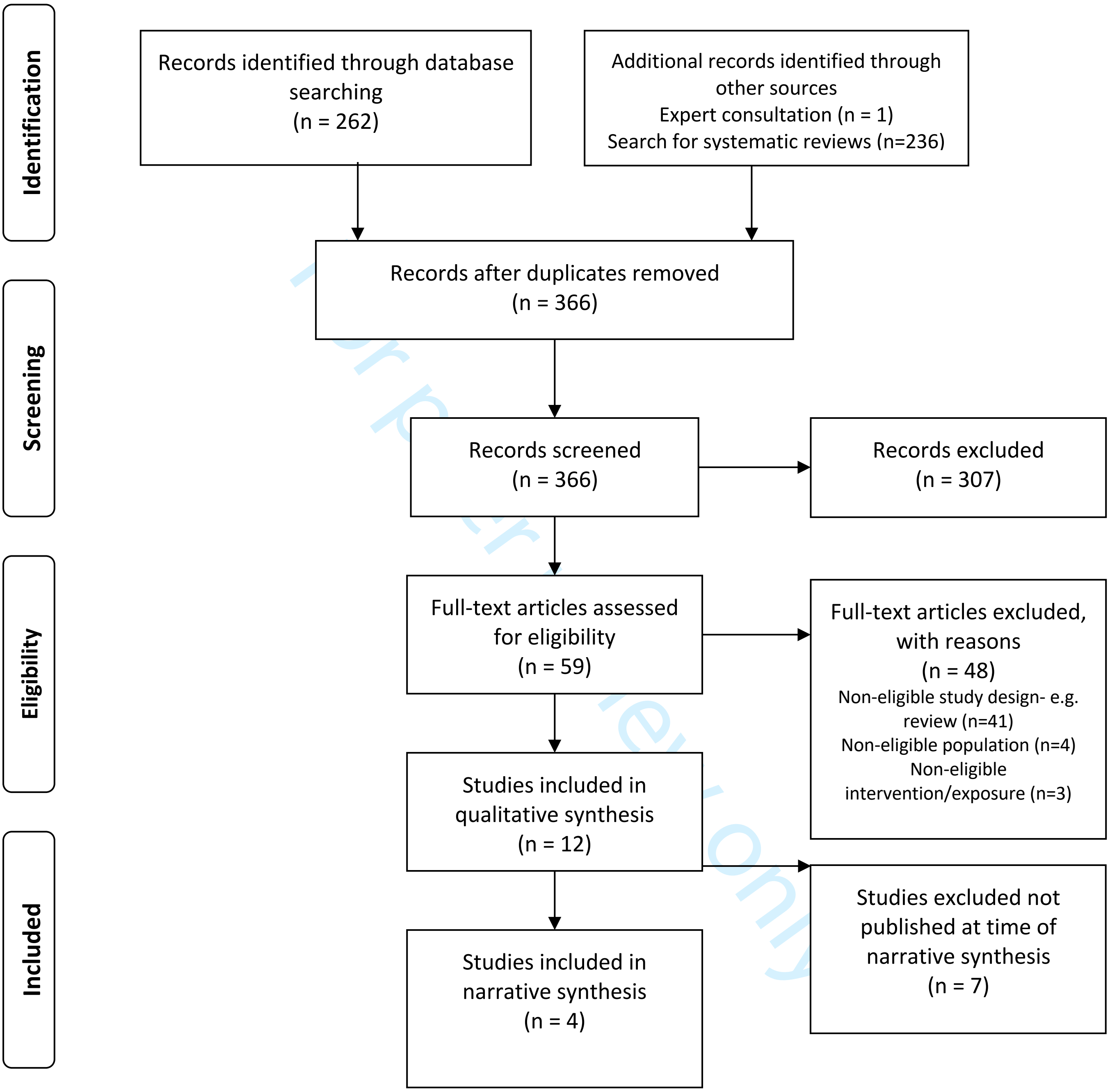
Identification
Screening
Eligibility
Included
Records identified through database searching (n = 262)
Additional records identified through other sources
Expert consultation (n = 1)
Search for systematic reviews (n=236)
Records after duplicates removed (n = 366)
Records screened (n = 366)
Records excluded (n = 307)
Full-text articles assessed for eligibility (n = 59)
Full-text articles excluded, with reasons (n = 48)
Non-eligible study design- e.g. review (n=41)
Non-eligible population (n=4)
Non-eligible intervention/exposure (n=3)
Studies included in qualitative synthesis (n = 12)
Studies excluded not published at time of narrative synthesis (n = 7)
Studies included in narrative synthesis (n = 4)

Supplemental file

Contents

1. Full record of search

2. Full details of the study eligibility criteria

3. List of studies excluded at full text review

4. Articles included at full text, but later excluded at time of narrative synthesis

5. Quality assessment of included studies

1. <u>Full record of search</u>

Medline (Ovid)

Search date: 06/05/2020
Database: Ovid MEDLINE(R) ALL <1946 to May 05, 2020>
Search Strategy:
----
1 exp Vitamin D/ (58492)
2 Vitamin D Deficiency/ (15552)
3 (vitamin D? or vit D? or D vitamin? or ergocalciferol or cholecalciferol or colecalciferol or calciferol or dihydroergocalciferol or sitocalciferol or dihydrotachysterol or dihydroxycholecalciferol? or hydroxycholecalciferol? or "25(OH)D?" or 25OHD? or calcidiol or hydroxyergocalciferol or alfacalcidol or alphacalcidol or "1,25(OH)2D" or hydroxyvitamin D? or dihydroxyvitamin D?).ab,kf,ti. (78232)
4 (paricalcitol or doxercalciferol or doxerocalciferol or calcifediol or calcitriol).ab,kf,ti. (5577)
5 hypovitaminosis D?.ab,kf,ti. (1775)
6 ((D or D2 or D3) adj3 supplement*).ab,kf,ti. (12158)
7 1 or 2 or 3 or 4 or 5 or 6 (92560)
8 coronavirus/ or betacoronavirus/ or betacoronavirus 1/ or coronavirus oc43, human/ or middle east respiratory syndrome coronavirus/ or sars virus/ (7431)
9 coronavirus infections/ or severe acute respiratory syndrome/ (10675)
10 (((corona* or corono*) adj1 (virus* or viral* or virinae*)) or coronavirus* or coronovirus* or coronavirinae* or Coronavirus* or Coronovirus* or Wuhan* or Hubei* or Huanan or "2019-nCoV" or 2019nCoV or nCoV2019 or "nCoV-2019" or "COVID-19" or COVID19 or "CORVID-19" or CORVID19 or "WN-CoV" or WNCoV or "HCoV-19" or HCoV19 or CoV or "2019 novel*" or Ncov or "n-cov" or "SARS-CoV-2" or "SARSCoV-2" or "SARSCoV2" or "SARS-CoV2" or SARSCov19 or "SARS-Cov19" or "SARSCov-19" or "SARS-Cov-19" or Ncovor or Ncorona* or Ncorono* or NcovWuhan* or NcovHubei* or NcovChina* or NcovChinese*).ab,kf,ti. (26891)
11 (severe acute respiratory syndrome or SARS or Middle East respiratory syndrome or MERS).ab,kf,ti. (16560)
12 (betacoronavirus* or betacoronavirinae*).ab,kf,ti. (280)
13 8 or 9 or 10 or 11 or 12 (37180)
14 7 and 13 (32)
15 exp Animals/ (23144176)

For peer review only

16 exp Humans/ (18448248)
17 15 not 16 (4695928)
18 14 not 17 (30)
19 limit 18 to yr="2002 -Current" (30)

Update
Search date: 10/6/2020
Actual databases searched: Ovid MEDLINE All <1946 to June 09, 2020>
Search strategy:
Re-ran search above plus…
20 limit 19 to ed=20200506-20200610 (8)
21 limit 19 to ep=20200506-20200610 (39)
22 limit 19 to dt=20200506-20200610 (43)
23 limit 19 to ez=20200506-20200610 (27)
24 20 or 21 or 22 or 23 (46)

Embase (Ovid)

Search date: 06/05/2020
Database: Embase <1974 to 2020 May 05>
Search Strategy:
----
1 exp vitamin D/ (139781)
2 vitamin D deficiency/ (29333)
3 (vitamin D? or vit D? or D vitamin? or ergocalciferol or cholecalciferol or colecalciferol or calciferol or dihydroergocalciferol or sitocalciferol or dihydrotachysterol or dihydroxycholecalciferol? or hydroxycholecalciferol? or "25(OH)D?" or 25OHD? or calcidiol or hydroxyergocalciferol or alfacalcidol or alphacalcidol or "1,25(OH)2D" or hydroxyvitamin D? or dihydroxyvitamin D?).ab,kw,ti. (112459)
4 (paricalcitol or doxercalciferol or doxerocalciferol or calcifediol or calcitriol).ab,kw,ti. (8478)
5 hypovitaminosis D?.ab,kw,ti. (3012)
6 ((D or D2 or D3) adj3 supplement*).ab,kw,ti. (19177)
7 1 or 2 or 3 or 4 or 5 or 6 (163395)
8 betacoronavirus 1/ or betacoronavirus/ or human coronavirus oc43/ (696)
9 Middle East respiratory syndrome coronavirus/ (2028)
10 sars-related coronavirus/ or sars coronavirus/ (6354)
11 Coronavirinae/ (2231)
12 coronavirus infection/ or middle east respiratory syndrome/ or severe acute respiratory syndrome/ (11950)
13 (((corona* or corono*) adj1 (virus* or viral* or virinae*)) or coronavirus* or coronovirus* or coronavirinae* or Coronavirus* or Coronovirus* or Wuhan* or Hubei* or Huanan or "2019-nCoV" or 2019nCoV or nCoV2019 or "nCoV-2019" or "COVID-19" or COVID19 or "CORVID-19" or CORVID19 or "WN-CoV" or WNCoV or "HCoV-19" or HCoV19 or CoV or "2019 novel*" or Ncov or "n-cov" or "SARS-CoV-2" or "SARSCoV-2" or "SARSCoV2" or "SARS-CoV2" or SARSCov19 or "SARS-Cov19" or "SARSCov-19" or "SARS-Cov-19" or Ncovor or Ncorona* or Ncorono* or NcovWuhan* or NcovHubei* or NcovChina* or NcovChinese*).ab,kw,ti. (27686)
14 (severe acute respiratory syndrome or SARS or Middle East respiratory syndrome or MERS).ab,kw,ti. (17146)
15 (betacoronavirus* or betacoronavirinae*).ab,kw,ti. (275)
16 8 or 9 or 10 or 11 or 12 or 13 or 14 or 15 (40716)

For peer review only

17 7 and 16 (61)
18 exp animal/ (25459151)
19 exp human/ (20834835)
20 18 not 19 (4624316)
21 17 not 20 (58)
22 limit 21 to yr="2002 -Current" (58)

Update
Search date: 10/6/2020
Actual databases searched: Ovid Embase <1974 to 2020 June 09>
Search strategy:
Re-ran search above plus…
22 limit 21 to yr="2002 -Current" (123)
23 limit 22 to dd=20200506-20200610 (39)
24 limit 22 to em=202005-202006 (0)
25 limit 22 to dc=20200506-20200610 (62)
26 23 or 24 or 25 (62)

MedrXiv (searched via Medrxivr https://mcguinlu.shinyapps.io/medrxivr/)

Search date: 07/05/2020
Search Strategy:

Topic 1:

[Vv]itamin D
[Vv]itamin D2
[Vv]itamin D3
calciferol
25OHD
25OHD3
[Hh]ypovitaminosis D

Topic 2:

[Cc]oronavirus
[Cc]orona(\\s)([[:graph:]]+\\s){0,1}virus
[Cc]oronavirinae
[Cc]ovid
COVID
nCoV
NCOV
Ncov
[Nn]-cov
N-COV
2019ncov
2019-ncov
ncov2019
ncov-2019

For peer review only - http://bmjopen.bmj.com/site/about/guidelines.xhtml

SARS
[Ss]evere [Aa]cute [Rr]espiratory [Ss]yndrome
[Mm]iddle [Ee]ast [Rr]espiratory [Ss]yndrome
MERS

Earliest record date
20190101
Latest record date
20200507
Remove older versions of the same record

6 results

<u>Update</u>
Search date: 10/6/2020
Re-ran search above changing record dates as follows:
Earliest record date
20200507
Latest record date
20200610
Remove older versions of the same record

11 results

BioRxiv
https://www.biorxiv.org/

Search date: 07/05/2020

65 Results
for abstract or title "vitamin D" (match phrase words)

22 Results
for full text or abstract or title "ergocalciferol cholecalciferol colecalciferol" (match whole any)

41 Results
for full text or abstract or title "25OHD 25OHD3" (match whole any)

Imported into EndNote and de-duplicated
92 results after deduplication

Searched in Endnote using the following search strategy:
coronavirus or corona or covid or SARS or MERS or betacoronavirus or ncov
Any Field

5 results

<u>Update</u>
Search date: 10/6/2020

For peer review only - http://bmjopen.bmj.com/site/about/guidelines.xhtml

1 Results
for abstract or title "vitamin D" (match phrase words) and posted between "07 May, 2020 and 10 Jun, 2020" – *animal study (also in both results sets below) so not exported to EndNote*

3 Results
for full text or abstract or title "ergocalciferol cholecalciferol colecalciferol" (match whole any) and posted between "07 May, 2020 and 10 Jun, 2020" - *2 animal studies and 1 on sertraline in TB*

2 Results
for full text or abstract or title "25OHD 25OHD3" (match whole any) and posted between "07 May, 2020 and 10 Jun, 2020" - *1 animal study, 1 non-clinical / non-coronavirus*

0 results relevant to coronaviruses

Cochrane Library

Search date: 08/05/2020

ID Search Hits
#1 MeSH descriptor: [Vitamin D] explode all trees 5224
#2 MeSH descriptor: [Vitamin D Deficiency] this term only 1226
#3 ((vitamin NEXT D?) or (vit NEXT D?) or (D NEXT vitamin?) or ergocalciferol or cholecalciferol or colecalciferol or calciferol or dihydroergocalciferol or sitocalciferol or dihydrotachysterol or dihydroxycholecalciferol? or hydroxycholecalciferol? or "25(OH)D" or "25(OH)D3" or 25OHD? or calcidiol or hydroxyergocalciferol or alfacalcidol or alphacalcidol or "1,25(OH)2D" or (hydroxyvitamin NEXT D?) or (dihydroxyvitamin NEXT D?)):ti,ab,kw 12959
#4 (paricalcitol or doxercalciferol or doxerocalciferol or calcifediol or calcitriol):ti,ab,kw 2417
#5 hypovitaminosis NEXT D? 303
#6 ((D OR D2 OR D3) NEAR/3 supplement*):ti,ab,kw 5633
#7 #1 or #2 or #3 or #4 or #5 or #6 14461
#8 MeSH descriptor: [Coronavirus] this term only 2
#9 MeSH descriptor: [Betacoronavirus] this term only 2
#10 MeSH descriptor: [Betacoronavirus 1] this term only 0
#11 MeSH descriptor: [Coronavirus OC43, Human] this term only 0
#12 MeSH descriptor: [Middle East Respiratory Syndrome Coronavirus] explode all trees 1
#13 MeSH descriptor: [SARS Virus] this term only 9
#14 MeSH descriptor: [Coronavirus Infections] this term only 137
#15 MeSH descriptor: [Severe Acute Respiratory Syndrome] this term only 107
#16 (((corona* or corono*) near/1 (virus* or viral* or virinae*)) or coronavirus* or coronovirus* or coronavirinae* or Coronavirus* or Coronovirus* or Wuhan* or Hubei* or Huanan or "2019-nCoV" or 2019nCoV or nCoV2019 or "nCoV-2019" or "COVID-19" or COVID19 or "CORVID-19" or CORVID19 or "WN-CoV" or WNCoV or "HCoV-19" or HCoV19 or CoV or "2019 novel*" or Ncov or "n-cov" or "SARS-CoV-2" or "SARSCoV-2" or "SARSCoV2" or "SARS-CoV2" or SARSCov19 or "SARS-Cov19" or "SARSCov-19" or "SARS-Cov-19" or Ncovor or Ncorona* or Ncorono* or NcovWuhan* or NcovHubei* or NcovChina* or NcovChinese*):ti,ab,kw 614
#17 ("severe acute respiratory syndrome" or SARS or "Middle East respiratory syndrome" or MERS):ti,ab,kw 350
#18 (betacoronavirus* or betacoronavirinae*):ti,ab,kw 4

For peer review only - http://bmjopen.bmj.com/site/about/guidelines.xhtml

#19 #8 or #9 or #10 or #11 or #12 or #13 or #14 or #15 or #16 or #17 or #18 798
#20 #7 and #19 3

[all 3 results were from CENTRAL]

Update
Search date: 10/06/2020
Re-ran search exactly as above and retrieved **5** results, all from CENTRAL. All 5 results exported to EndNote for deduplication.

Database of publications (living map of evidence) on coronavirus disease (COVID-19) developed by the University of Bern

Living Evidence on COVID-19
Contributors: Michel Counotte, Hira Imeri, Mert Ipekci, Nicola Low

https://zika.ispm.unibe.ch/assets/data/pub/ncov/

Search date: 10/05/2020 (14,988 entries)

Search: Title, Abstract
Search:

vitamin D 13
vitamin D2 0
vitamin D3 0
ergocalciferol 0
cholecalciferol 0
colecalciferol 0
25(OH)D 0
25OHD 0
25(OH)D3 0
25OHD3 0
hypovitaminosis D 1
Vitamin D Deficiency 1

Oxford COVID-19 Evidence Service
https://www.cebm.net/oxford-covid-19-evidence-service/
The Centre for Evidence-Based Medicine (CEBM) The University of Oxford

Search date: 10/05/2020 (142 articles)
vitamin D 1
vitamin D2 0
vitamin D3 0
ergocalciferol 0
cholecalciferol 0
colecalciferol 0
25(OH)D 0
25OHD 0

For peer review only - http://bmjopen.bmj.com/site/about/guidelines.xhtml
For peer review only

25(OH)D3 0
25OHD3 0
hypovitaminosis D 0
Vitamin D Deficiency 0

Database of publications on coronavirus disease (COVID-19) developed by WHO
https://www.who.int/emergencies/diseases/novel-coronavirus-2019/global-research-on-novel-coronavirus-2019-ncov

Search date: 10/05/2020 (15,253 entries)

Search: Title, Abstract, Subject

vitamin D 19
vitamin D2 0
vitamin D3 2
ergocalciferol 0
cholecalciferol 1
colecalciferol 0
25(OH)D 0
25OHD 0
25(OH)D3 0
25OHD3 0
hypovitaminosis D 1
Vitamin D Deficiency 2

Total: 25
After de-duplication: 20

Searches for systematic reviews, for reference checking

Medline

Search date: 19/05/2020
Database: Ovid MEDLINE(R) ALL <1946 to May 18, 2020>
Search Strategy:
----
1 exp Vitamin D/ (58577)
2 Vitamin D Deficiency/ (15588)
3 (vitamin D? or vit D? or D vitamin? or ergocalciferol or cholecalciferol or colecalciferol or calciferol or dihydroergocalciferol or sitocalciferol or dihydrotachysterol or dihydroxycholecalciferol? or hydroxycholecalciferol? or "25(OH)D?" or 25OHD? or calcidiol or hydroxyergocalciferol or alfacalcidol or alphacalcidol or "1,25(OH)2D" or hydroxyvitamin D? or dihydroxyvitamin D?).ab,kf,ti. (78395)
4 (paricalcitol or doxercalciferol or doxerocalciferol or calcifediol or calcitriol).ab,kf,ti. (5588)
5 hypovitaminosis D?.ab,kf,ti. (1780)
6 ((D or D2 or D3) adj3 supplement*).ab,kf,ti. (12198)
7 1 or 2 or 3 or 4 or 5 or 6 (92747)

For peer review only

8 coronavirus/ or betacoronavirus/ or betacoronavirus 1/ or coronavirus oc43, human/ or middle east respiratory syndrome coronavirus/ or sars virus/ (8161)
9 coronavirus infections/ or severe acute respiratory syndrome/ (11614)
10 (((corona* or corono*) adj1 (virus* or viral* or virinae*)) or coronavirus* or coronovirus* or coronavirinae* or Coronavirus* or Coronovirus* or Wuhan* or Hubei* or Huanan or "2019-nCoV" or 2019nCoV or nCoV2019 or "nCoV-2019" or "COVID-19" or COVID19 or "CORVID-19" or CORVID19 or "WN-CoV" or WNCoV or "HCoV-19" or HCoV19 or CoV or "2019 novel*" or Ncov or "n-cov" or "SARS-CoV-2" or "SARSCoV-2" or "SARSCoV2" or "SARS-CoV2" or SARSCov19 or "SARS-Cov19" or "SARSCov-19" or "SARS-Cov-19" or Ncovor or Ncorona* or Ncorono* or NcovWuhan* or NcovHubei* or NcovChina* or NcovChinese*).ab,kf,ti. (31115)
11 (severe acute respiratory syndrome or SARS or Middle East respiratory syndrome or MERS).ab,kf,ti. (17795)
12 (betacoronavirus* or betacoronavirinae*).ab,kf,ti. (294)
13 exp Respiratory Tract Infections/ (356696)
14 (acute respiratory infection* or severe respiratory infection* or acute respiratory tract infection* or severe respiratory tract infection* or influenza or common cold or pneumonia or bronchitis).ab,kf,ti. (234266)
15 8 or 9 or 10 or 11 or 12 or 13 or 14 (503079)
16 7 and 15 (1062)
17 (metaanalys* or "meta analys*" or "meta-analys*").tw. (169008)
18 (systematic* adj3 review*).mp. (200684)
19 meta analysis.pt. (114746)
20 17 or 18 or 19 (301767)
21 16 and 20 (55)

Embase

Search date: 19/05/2020

Database: Embase Classic+Embase <1947 to 2020 Week 20>
Search Strategy:
----
1 exp vitamin D/ (147053)
2 vitamin D deficiency/ (30106)
3 (vitamin D? or vit D? or D vitamin? or ergocalciferol or cholecalciferol or colecalciferol or calciferol or dihydroergocalciferol or sitocalciferol or dihydrotachysterol or dihydroxycholecalciferol? or hydroxycholecalciferol? or "25(OH)D?" or 25OHD? or calcidiol or hydroxyergocalciferol or alfacalcidol or alphacalcidol or "1,25(OH)2D" or hydroxyvitamin D? or dihydroxyvitamin D?).ab,kw,ti. (118981)
4 (paricalcitol or doxercalciferol or doxerocalciferol or calcifediol or calcitriol).ab,kw,ti. (8485)
5 hypovitaminosis D?.ab,kw,ti. (3033)
6 ((D or D2 or D3) adj3 supplement*).ab,kw,ti. (19335)
7 1 or 2 or 3 or 4 or 5 or 6 (172654)
8 betacoronavirus 1/ or betacoronavirus/ or human coronavirus oc43/ (1085)
9 Middle East respiratory syndrome coronavirus/ (2082)
10 sars-related coronavirus/ or sars coronavirus/ (6062)
11 Coronavirinae/ (2060)
12 coronavirus infection/ or middle east respiratory syndrome/ or severe acute respiratory syndrome/ (12565)

For peer review only

13 (((corona* or corono*) adj1 (virus* or viral* or virinae*)) or coronavirus* or coronovirus* or coronavirinae* or Coronavirus* or Coronovirus* or Wuhan* or Hubei* or Huanan or "2019-nCoV" or 2019nCoV or nCoV2019 or "nCoV-2019" or "COVID-19" or COVID19 or "CORVID-19" or CORVID19 or "WN-CoV" or WNCoV or "HCoV-19" or HCoV19 or CoV or "2019 novel*" or Ncov or "n-cov" or "SARS-CoV-2" or "SARSCoV-2" or "SARSCoV2" or "SARS-CoV2" or SARSCov19 or "SARS-Cov19" or "SARSCov-19" or "SARS-Cov-19" or Ncovor or Ncorona* or Ncorono* or NcovWuhan* or NcovHubei* or NcovChina* or NcovChinese*).ab,kw,ti. (30532)
14 (severe acute respiratory syndrome or SARS or Middle East respiratory syndrome or MERS).ab,kw,ti. (17954)
15 (betacoronavirus* or betacoronavirinae*).ab,kw,ti. (286)
16 exp respiratory tract infection/ (460049)
17 (acute respiratory infection* or severe respiratory infection* or acute respiratory tract infection* or severe respiratory tract infection* or influenza or common cold or pneumonia or bronchitis).ab,kw,ti. (329779)
18 8 or 9 or 10 or 11 or 12 or 13 or 14 or 15 or 16 or 17 (674800)
19 7 and 18 (3315)
20 (metaanalys* or "meta analys*" or "meta-analys*").mp. (294469)
21 (systematic* adj2 review*).mp. (330720)
22 20 or 21 (475492)
23 19 and 22 (219)
24 limit 19 to (meta analysis or "systematic review") (145)
25 23 or 24 (219)
26 limit 25 to (conference abstract or conference paper or "conference review" or editorial or letter) (41)
27 25 not 26 (178)

Cochrane Database of Systematic Reviews (Cochrane Library)

Search Name: Vitamin D Covid and Acute Respiratory Infections SRs
Date Run: 20/05/2020 18:30:28
Comment:

ID	Search	Hits
#1	MeSH descriptor: [Vitamin D] explode all trees	5224
#2	MeSH descriptor: [Vitamin D Deficiency] this term only	1226
#3	((vitamin NEXT D?) or (vit NEXT D?) or (D NEXT vitamin?) or ergocalciferol or cholecalciferol or colecalciferol or calciferol or dihydroergocalciferol or sitocalciferol or dihydrotachysterol or dihydroxycholecalciferol? or hydroxycholecalciferol? or "25(OH)D" or "25(OH)D3" or 25OHD? or calcidiol or hydroxyergocalciferol or alfacalcidol or alphacalcidol or "1,25(OH)2D" or (hydroxyvitamin NEXT D?) or (dihydroxyvitamin NEXT D?)):ti,ab,kw	12959
#4	(paricalcitol or doxercalciferol or doxerocalciferol or calcifediol or calcitriol):ti,ab,kw	2417
#5	hypovitaminosis NEXT D?	303
#6	((D OR D2 OR D3) NEAR/3 supplement*):ti,ab,kw	5632
#7	#1 or #2 or #3 or #4 or #5 or #6	14461
#8	MeSH descriptor: [Coronavirus] this term only	2
#9	MeSH descriptor: [Betacoronavirus] this term only	2
#10	MeSH descriptor: [Betacoronavirus 1] this term only	0
#11	MeSH descriptor: [Coronavirus OC43, Human] this term only	0
#12	MeSH descriptor: [Middle East Respiratory Syndrome Coronavirus] explode all trees	1

For peer review only - http://bmjopen.bmj.com/site/about/guidelines.xhtml

#13 MeSH descriptor: [SARS Virus] this term only 9
#14 MeSH descriptor: [Coronavirus Infections] this term only 137
#15 MeSH descriptor: [Severe Acute Respiratory Syndrome] this term only 107
#16 (((corona* or corono*) near/1 (virus* or viral* or virinae*)) or coronavirus* or coronovirus* or coronavirinae* or Coronavirus* or Coronovirus* or Wuhan* or Hubei* or Huanan or "2019-nCoV" or 2019nCoV or nCoV2019 or "nCoV-2019" or "COVID-19" or COVID19 or "CORVID-19" or CORVID19 or "WN-CoV" or WNCoV or "HCoV-19" or HCoV19 or CoV or "2019 novel*" or Ncov or "n-cov" or "SARS-CoV-2" or "SARSCoV-2" or "SARSCoV2" or "SARS-CoV2" or SARSCov19 or "SARS-Cov19" or "SARSCov-19" or "SARS-Cov-19" or Ncovor or Ncorona* or Ncorono* or NcovWuhan* or NcovHubei* or NcovChina* or NcovChinese*):ti,ab,kw 616
#17 ("severe acute respiratory syndrome" or SARS or "Middle East respiratory syndrome" or MERS):ti,ab,kw 351
#18 (betacoronavirus* or betacoronavirinae*):ti,ab,kw 4
#19 MeSH descriptor: [Respiratory Tract Infections] explode all trees 14360
#20 (("acute respiratory" NEXT infection*) or ("severe respiratory" NEXT infection*) or ("acute respiratory tract" NEXT infection*) or ("severe respiratory tract" NEXT infection*) or influenza or "common cold" or pneumonia or bronchitis):ti,ab,kw 25944
#21 #8 or #9 or #10 or #11 or #12 or #13 or #14 or #15 or #16 or #17 or #18 or #19 or #20 32554
#22 #7 and #21 329

CDSR: 3

Expert consultation

One additional study identified:

Martineau AR, Jolliffe DA, Hooper RL, et al., (2017) Vitamin D supplementation to prevent acute respiratory tract infections: systematic review and meta-analysis of individual participant data. BMJ. 2017;356:i6583. doi:10.1136/bmj.i6583

For peer review only

2. Full details of the study eligibility criteria

Include	Exclude
**P- Population**<br>1) Patients acutely ill with Betacoronavirus infection [SARS-CoV, MERS-CoV, SARS-CoV-2]<br>2) or at risk of acute illness with Betacoronavirus infection	Animals studies, modelling studies
**I – Intervention/exposure**<br>1) Vitamin D supplementation<br>2) Low Serum Vitamin D	
**O - Outcomes**<br>1) Betacoronavirus infection (to include serological evidence of infection or clinically confirmed symptomatic infection);<br>2) severity of Betacoronavirus infection (to include patients admitted to hospital or admitted to intensive care); mortality due to Betacoronavirus.<br>3) Mortality due to Betacoronavirus	
**C – Comparator**<br>1) No Vitamin D supplementation<br>2) high or normal Serum Vitamin D	
**S - Study design**<br>Randomised controlled trials and non-randomized studies will be eligible for inclusion in the review including, non randomized controlled trials, interrupted time series, controlled before-and-after studies, cohort studies, ecological studies, case reports and case series.	Qualitative studies, Non-primary research- reviews, editorials etc, guidelines and non-systematic reviews.
**Subgroups**<br>1. Ethnicity characteristics (White British, All Other White, Mixed, Asian, Black, Other)<br>2. Age characteristics (population by five-year age groups)	Non-English language. Non peer reviewed publication.

For peer review only

3. List of studies excluded at full text review

Excluded studies		Reason<br><br>*Non-eligible study design- e.g. review*<br>*Non-eligible population*<br>*Non-eligible intervention*<br>*No relevant outcome*<br>*No comparator group*
1	Adams, K. K., et al. (2020). "Myth Busters: Dietary Supplements and COVID-19." Annals of Pharmacotherapy: 1060028020928052.	Study design – commentary
2	Ahmed, I., et al. (2020). "First Covid-19 maternal mortality in the UK associated with thrombotic complications." British Journal of Haematology. **18**.	Study design – letter
3	Alpalhao, M. and P. Filipe (2020). "SARS-CoV-2 pandemic and Vitamin D deficiency - a double trouble." Photodermatology, Photoimmunology & Photomedicine **01**: 01.	Study design – letter
4	Annweiler, C., et al. (2020). COvid-19 and Vitamin D Supplementation: a Multicenter Randomized Controlled Trial of High Dose Versus Standard Dose Vitamin D3 in High-risk COVID-19 Patients (CoVitTrial). https://clinicaltrials.gov/show/NCT04344041.	Study design – on-going RCT completion date. July 2020
5	Arya, A. and V. D. Dwivedi (2020). "Synergistic effect of vitamin D and remdesivir can fight COVID-19." Journal of Biomolecular Structure & Dynamics: 1-2	Study design – letter
6	Banerjee, D., et al. (2020). "COVID-19 infection in kidney transplant recipients." Kidney International **97**(6): 1076-1082.	Study design – commentary
7	Caccialanza, R., et al. (2020). Early nutritional supplementation in non-critically ill patients hospitalized for the 2019 novel coronavirus disease (COVID-19): Rationale and feasibility of a shared pragmatic protocol. Nutrition: 110835.	Study design - protocol
8	Calder, P. C., et al. (2020). Optimal Nutritional Status for a Well-Functioning Immune System Is an Important Factor to Protect against Viral Infections. Nutrients: 12(4), 1181.	Study design -narrative review
9	Cao, Z., et al. (2020). SARS-CoV-2 & Covid-19: Key-Roles of the 'Renin-Angiotensin' System / Vitamin D Impacting Drug and Vaccine Developments. Infectious Disorders - Drug Targets. *(E-pub Ahead of Print)* DOI : 10.2174/1871526520999200505174704	Study design – editorial
10	Carter, S. J., et al. (2020). Considerations for obesity, vitamin D, and physical activity amidst the COVID-19 pandemic. Obesity **16**: 16.	Study design -narrative review

For peer review only - http://bmjopen.bmj.com/site/about/guidelines.xhtml

11	Daneshkhah, A., et al. (2020). The Possible Role of Vitamin D in Suppressing Cytokine Storm and Associated Mortality in COVID-19 Patients. medRxiv. DOI: https://doi.org/10.1101/2020.04.08.20058578	Study design – modelling
12	Davies G, Garami AR, Byers JC. Evidence Supports a Causal Model for Vitamin D in COVID-19 Outcomes. medRxiv, 2020. DOR: https://doi.org/10.1101/2020.05.01.20087965v3	Study design – modelling
13	de Lucena, T. M. C., et al. (2020). "Mechanism of inflammatory response in associated comorbidities in COVID-19." Diabetes & Metabolic Syndrome **14**(4): 597-600.	Study design -narrative review
14	Eroglu, C., et al. (2019). The relation between serum vitamin D levels, viral infections and severity of attacks in children with recurrent wheezing. Allergologia et Immunopathologia **47**(6): 591-597.	Population - Not COVID-19/SARs/MERs
15	Faul, J. L., et al. (2020). "Vitamin d deficiency and ards after sars-cov-2 infection." Irish Medical Journal **113**(5).	Study design – letter
16	Ghasemian, R., et al. (2020). "The Role of Vitamin D in The Age of COVID-19: A Systematic Review and Meta-Analysis Along with an Ecological Approach." MedRxiv : the Preprint Server for Health Sciences. DOI: 10.1101/2020.06.05.20123554	Study design -narrative review
17	Grant, W. B., et al. (2020). Evidence that Vitamin D Supplementation Could Reduce Risk of Influenza and COVID-19 Infections and Deaths. Nutrients **12**(4): 02.	Study design -narrative review
18	Heiser, K., et al. (2020). Identification of potential treatments for COVID-19 through artificial intelligence-enabled phenomic analysis of human cells infected with SARS-CoV-2. bioRxiv: 2020.2004.2021.054387.	Study design – modelling
19	Hribar, C. A., et al. (2020). "Potential Role of Vitamin D in the Elderly to Resist COVID-19 and to Slow Progression of Parkinson's Disease." Brain Sciences **10**(5): 08.	Study design -narrative review
20	Jakovac, H. (2020). COVID-19 and vitamin D-Is there a link and an opportunity for intervention? American Journal of Physiology - Endocrinology & Metabolism **318**(5): E589-E589.	Study design – letter
21	Jamaati, H., et al. (2020). A fourteen-day experience with coronavirus disease 2019 (COVID-19) induced acute respiratory distress syndrome (ARDS): An Iranian treatment protocol. Iranian Journal of Pharmaceutical Research **19**(1): 31-36.	Study design – treatment protocol/recommendation
22	Kalippurayil Moozhipurath, R., et al. (2020). "Evidence of Protective Role of Ultraviolet-B (UVB) Radiation in	Exposure – not vitamin D(UVB)

For peer review only - http://bmjopen.bmj.com/site/about/guidelines.xhtml

	Reducing COVID-19 Deaths." MedRxiv : the Preprint Server for Health Sciences. DOI 10.1101/2020.05.06.20093419	
23	Kara, M., et al. (2020). "'Scientific Strabismus' or Two Related Pandemics: COVID-19 & Vitamin D Deficiency." British Journal of Nutrition: 1-20.	Study design -narrative review
24	Koivisto, O., et al. (2020). Key Vitamin D Target Genes with Functions in the Immune System. Nutrients, **12**(4):1140.	Population - Outcomes - target genes
25	Kow, C. S., et al. (2020). "Vitamin D Supplementation in Influenza and COVID-19 Infections Comment on: "Evidence that Vitamin D Supplementation Could Reduce Risk of Influenza and COVID-19 Infections and Deaths" Nutrients 2020, 12(4), 988." Nutrients **12**(6): 01.	Study design – commentary
26	Kumar, V. and A. Srivastava (2020). "Spurious Correlation? A review of the relationship between Vitamin D and Covid-19 infection and mortality." MedRxiv : the Preprint Server for Health Sciences. DOI: 10.1101/2020.05.25.20110338	Study design -narrative review
27	Laird, E., et al. (2020). "Vitamin D and inflammation: Potential implications for severity of Covid-19." Irish Medical Journal **113**(5).	Study design -narrative review
28	La Vignera, S., et al. (2020). Sex-Specific SARS-CoV-2 Mortality: Among Hormone-Modulated ACE2 Expression, Risk of Venous Thromboembolism and Hypovitaminosis D. International journal of molecular sciences **21**(8):2948.	Study design – editorial
29	Li, A. Y., et al. (2020). Multivariate Analysis of Factors Affecting COVID-19 Case and Death Rate in U.S. Counties: The Significant Effects of Black Race and Temperature. medRxiv. DOI: https://doi.org/10.1101/2020.04.17.20069708	Intervention – not Vitamin D
30	Maestri, E., et al. (2020). Vitamin D and coronavirus: a new field of use?. Recenti Progressi in Medicina **111**(4): 253-256.	Study design -narrative review, non-English
31	Mansbach, J. M. and C. A. Camargo Jr., (2009). Respiratory Viruses in Bronchiolitis and Their Link to Recurrent Wheezing and Asthma. Clinics in Laboratory Medicine **29**(4): 741-755.	Population - Not COVID-19/SARs/MERs
32	Marik, P. E., et al. (2020). Does vitamin D status impact mortality from SARS-CoV-2 infection? Medicine in Drug Discovery: 100041-100041.	Study design - commentary
33	McKenna, M. J. and M. A. T. Flynn (2020). "Covid-19, cocooning and vitamin d intake requirements." Irish Medical Journal **113**(5).	Study design -narrative review

For peer review only - http://bmjopen.bmj.com/site/about/guidelines.xhtml

34	Mitchell, F. (2020). "Vitamin-D and COVID-19: do deficient risk a poorer outcome?" The Lancet Diabetes & Endocrinology **20**: 20.	Study design -narrative review
35	Molloy, E. J. and N. Murphy (2020). Vitamin D, Covid-19 and Children. Irish Medical Journal **113**(4): 64.	Study design -narrative review
36	McCartney, D. M. and D. G. Byrne (2020). Optimisation of Vitamin D Status for Enhanced Immuno-protection Against Covid-19. Irish Medical Journal **113**(4): 58.	Study design -narrative review
37	Rabbitt, L. and E. Slattery (2020). "Vitamin d and covid-19: A note of caution." Irish Medical Journal **113**(5).	Study design - letter
38	Ribeiro, H., et al. (2020). "Does Vitamin D play a role in the management of Covid-19 in Brazil?" Revista de Saude Publica **54**: 53.	Study design -narrative review
39	Romano, L., et al. (2020). "Short Report - Medical nutrition therapy for critically ill patients with COVID-19." European Review for Medical and Pharmacological Sciences **24**(7): 4035-4039.	Study design -narrative review
40	Silberstein, M. (2020). Vitamin D: A simpler alternative to tocilizumab for trial in COVID-19? Medical Hypotheses **140**: 109767-109767.	Study design -narrative review
41	Speeckaert, M. M. and J. R. Delanghe (2020). "Association between low vitamin D and COVID-19: don't forget the vitamin D binding protein." Aging Clinical & Experimental Research **28**: 28.	Study design - letter
42	Skutsch, M., et al. (2020). "The association of UV with rates of COVID-19 transmission and deaths in Mexico: the possible mediating role of vitamin D." MedRxiv : the Preprint Server for Health Sciences. DOI: 10.1101/2020.05.25.20112805	Study design – modelling
43	Suresh, P. S. (2020). "Hypovitaminosis D and COVID-19: Matter of Concern in India?" Indian Journal of Clinical Biochemistry.	Study design - letter
44	Taghizadieh, A., et al. (2020). "Acute kidney injury in pregnant women following SARS-CoV-2 infection: A case report from Iran." Respiratory Medicine Case Reports 30	Exposure – not vitamin D
45	Tan, S. H. S., et al. (2020). "Medications in COVID-19 patients: summarizing the current literature from an orthopaedic perspective." International Orthopaedics **22**: 22.	Study design -narrative review
46	Yalaki, Z., et al. (2019). Comparison of viral agents and vitamin D levels in children with acute bronchiolitis infection. Cocuk Enfeksiyon Dergisi **13**(1): e14-e20.	Population - COVID-19/SARs/MERs not specified
47	Zabetakis, I., et al. (2020). "COVID-19: The Inflammation Link and the Role of Nutrition in Potential Mitigation." Nutrients **12**(5): 19.	Study design -narrative review

For peer review only - http://bmjopen.bmj.com/site/about/guidelines.xhtml

48	Zemb, P., et al. (2020). "Vitamin D deficiency and COVID-19 pandemic." Journal of Global Antimicrobial Resistance **28**: 28.	Study design - commentary

4. Articles included at full text, but later excluded at time of narrative synthesis

	Citation record	Exclusion reason	Update performed 8th October 2020
49	Darling, A. L., et al. (2020). Vitamin D status, body mass index, ethnicity and COVID-19: Initial analysis of the first-reported UK Biobank COVID-19 positive cases (n 580) compared with negative controls (n 723). medRxiv.<br><br>https://www.medrxiv.org/content/10.1101/2020.04.29.20084277v1?versioned=TRUE	Not peer reviewed publication at time of narrative synthesis	No update available
50	De Smet, D., et al. (2020). Vitamin D deficiency as risk factor for severe COVID-19: a convergence of two pandemics. medRxiv.<br><br>https://www.medrxiv.org/content/10.1101/2020.05.01.20079376v2	Not peer reviewed publication at time of narrative synthesis	No update available
51	Lau, F. H., et al. (2020). Vitamin D Insufficiency is Prevalent in Severe COVID-19. medRxiv.<br><br>https://www.medrxiv.org/content/10.1101/2020.04.24.20075838v1?versioned=TRUE	Not peer reviewed publication at time of narrative synthesis	No update available
52	Meltzer, D. O., et al. (2020). "Association of Vitamin D Deficiency and Treatment with COVID-19 Incidence." MedRxiv : the Preprint Server for Health Sciences **13**: 13.	Not peer reviewed public	An updated publication is available at https://jamanetwork.com/journals/jamanetworkopen/fullarticle/2770157<br><br>Citation

For peer review only - http://bmjopen.bmj.com/site/about/guidelines.xhtml

	https://www.medrxiv.org/content/10.1101/2020.05.08.20095893v1	ation at time of narrative synthesis	Meltzer DO, Best TJ, Zhang H, Vokes T, Arora V, Solway J. Association of Vitamin D Status and Other Clinical Characteristics With COVID-19 Test Results. *JAMA Netw Open.* 2020;3(9):e2019722. doi:10.1001/jamanetworkopen.2020.19722
53	Notari, A. and G. Torrieri (2020). "COVID-19 transmission risk factors." MedRxiv : the Preprint Server for Health Sciences.<br><br>https://www.medrxiv.org/content/10.1101/2020.05.08.20095083v1?versioned=TRUE	Not peer reviewed publication at time of narrative synthesis	No update available
54	Raisi-Estabragh, Z., et al. (2020). "Greater risk of severe COVID-19 in non-White ethnicities is not explained by cardiometabolic, socioeconomic, or behavioural factors, or by 25(OH)-vitamin D status: study of 1,326 cases from the UK Biobank." MedRxiv : the Preprint Server for Health Sciences.<br><br>https://www.medrxiv.org/content/10.1101/2020.06.01.20118943v1?versioned=TRUE	Not peer reviewed publication at time of narrative synthesis	An updated publication is available at https://academic.oup.com/jpubhealth/article/42/3/451/5859581<br><br>Citation<br>Zahra Raisi-Estabragh, Celeste McCracken, Mae S Bethell, Jackie Cooper, Cyrus Cooper, Mark J Caulfield, Patricia B Munroe, Nicholas C Harvey, Steffen E Petersen, Greater risk of severe COVID-19 in Black, Asian and Minority Ethnic populations is not explained by cardiometabolic, socioeconomic or behavioural factors, or by 25(OH)-vitamin D status: study of 1326 cases from the UK Biobank, *Journal of Public Health*, Volume 42, Issue 3, September 2020, Pages 451–460, https://doi.org/10.1093/pubmed/fdaa095
55	Tan, C. W., et al. (2020). "A cohort study to evaluate the effect of combination Vitamin D, Magnesium and Vitamin B12 (DMB) on progression to severe outcome in older COVID-19 patients." MedRxiv : the Preprint Server for Health Sciences.<br><br>https://www.medrxiv.org/content/10.1101/2020.06.01.20112334v2	Not peer reviewed publication at time of narrative synthesis	No update available

For peer review only - http://bmjopen.bmj.com/site/about/guidelines.xhtml

For peer review only

Study details of the seven articles included at full text, but excluded at time of narrative synthesis						
**Study**	**Design/ setting**	**Population**	**Exposure/Intervention**	**Outcomes**	**Results**	**Limitations**
Darling, A. L., et al. (2020)[1]	Retrospective cohort study<br><br>UK Biobank England cohort only	COVID-19 positive cases (n 580) Mean age 57.5 (SD 9.7)<br><br>COVID-19 negative controls (n 723) Mean age 57.9 (SD 8.7)	Serum 25(OH)D status Median (IQR) nmol/L<br><br>by gender (Male/Female), body mass index (Normal/underweight, overweight, obesity), ethnicity (Asian, Black, Mixed and Other, White)	COVID-19 test result	Serum 25(OH)D status similar in both groups:<br><br>COVID-19 positive cases (median IQR) = 43.3 (32.1) nmol/L)<br><br>COVID-19 negative controls (median (IQR) 44.1 (31.2) nmol/L) for COVID-19.<br><br>A logistic regression model suggests that being overweight (OR 1.51 CI 1.13-2.02) or obese (OR 1.67 CI 1.24-2.26); living in London (OR 1.45 CI 1.05-2.00); being male (OR 1.28 CI 1.01-1.61) and being of Asian, Black or Mixed ethnicity (OR 1.66 CI 1.08-2.54) is associated with a higher odds of testing positive for COVID-19	UK Biobank baseline samples collected in 2006-2010.
De Smet, D., et al. (2020)[2]	Retrospective observational study<br><br>Central network hospital, West Flanders, Belgium	186 SARS-CoV-2 infected patients hospitalised from March 1, 2020 to April 7, 2020<br><br>(109 males [median age 68 years, IQR 53-79] 77 females [median age 71 years, IQR 65-74])<br><br>25(OH)D in COVID-19 patients was compared a control group of 2717 patients with similar age distribution, sampled from March 1, 2019 to April 30, 2019. (999 males [median age 69 years, IQR 53-81] and 1718 females [median age 68 years, IQR 43-83]).	25(OH)D levels	SARS-CoV-2 infection	COVID-19 patients had a lower median 25(OH)D on admission (18.6 ng/mL, IQR 12.6-25.3) than controls (21.5 ng/mL, IQR 13.9-20.8, P=0.0016) and a higher percentage of vitamin D deficiency (defined as 25(OH)D < 20ng/mL): 58.6% versus 45.2% (P=0.0005).<br><br>In male COVID-19 patients, vitamin D deficiency was lower median 25(OH)D (17.6 ng/mL, IQR 12.7-24.0 versus 20.3 ng/mL, IQR 13.7-28.3, P=0.0234) and a higher deficiency rate (67.0% versus 49.2%, P=0.0006) than male controls.	The prevalence and age/sex/ seasonal-distribution of vitamin D status was derived from the general population sampled from 16274 consecutive, unselected and unique patients from January 1, 2019 to December 31, 2019.

For peer review only - http://bmjopen.bmj.com/site/about/guidelines.xhtml

Lau, F. H., et al. (2020)[3]	Retrospective observational study<br><br>A single, tertiary care academic (university) medical centre, Louisiana, New Orleans, USA	COVID-19 ICU patients (n 13) Mean age 61.5 (SD 15.7)<br><br>COVID-19 floor patients (n 7) Mean age 72.0 (SD 14.8)<br><br>Medical records of COVID-19 patients between March 27, 2020 and April 21, 2020<br><br>Vitamin D insufficiency (VDI) and COVID-19 metrics in ICU vs. floor patients	VDI: defined as serum 25(OH) D < 30 ng/mL)<br><br>Serum 25(OH) D status Mean (SD) ng/mL<br><br>by gender (Male/Female), body mass index (Normal, obesity), race (African American), age (elderly>65 years), hypertension	COVID-19 metrics	Overall, few significant differences were identified between ICU and floor patients: Lactate dehydrogenase was significantly higher among ICU patients (441.8 vs. 223.0, P=0.001). Also, body mass index was significantly higher among ICU patients (35.2 vs. 24.5, P=0.02).<br><br>Among ICU subjects, 11 (84.6%) had VDI, vs. 4 (57.1%) of floor subjects. 100% of ICU patients less than 75 years old had VDI (n=11). Among these, 64.6% (n=7) had critically low 25(OH) D (<20 ng/mL) and 3 had <10 ng/mL.<br><br>VDI is highly prevalent in dark-skinned people (82.1% of African Americans vs. 41.6% overall).<br><br>Male/Female ratio was 1.24 and 1.44 for COVID-19 and VDI respectively.	Statistical analysis was limited by the small number of subjects.
Meltzer, D. O., et al. (2020)[4]	Retrospective cohort study<br><br>University of Chicago Medicine, USA	4,314 patients tested for COVID-19 from 3/3/2020 to 4/10/2020. Among these, 499 had a vitamin D level in the year before testing.<br><br>COVID-19 positive cases with vitamin D deficient (n 178) Mean age 45.6<br><br>COVID-19 positive cases with not vitamin D deficient (n 321) Mean age 50.7	Vitamin D deficiency: defined by the most recent 25(OH) D <20ng/ml or 1,25-dihydroxycholecalciferol <18pg/ml within 1 year before COVID-19 testing. Treatment: defined by the most recent vitamin D type and dose, and treatment changes between the time of the most recent vitamin D level and time of COVID-19 testing<br><br>Vitamin D deficiency and treatment changes were combined to categorize vitamin D status at the time of COVID-19 testing as:<br>1)Likely deficient (last-level-deficient/treatment-not-increased)	Testing positive for COVID-19	In multivariable analysis, testing positive for COVID-19 was associated with increasing age (RR (age<50)=1.05, P<0.021; RR (age≥50)=1.02, P<0.064)), non-white race (RR=2.54, P<0.01) and being likely vitamin D deficient (deficient/treatment-not-increased: RR=1.77, P<0.02) as compared to likely vitamin D sufficient (not-deficient/treatment-not-decreased), with predicted COVID-19 rates in the vitamin D deficient group of 21.6% (95%CI [14.0%-29.2%]) vs 12.2% (95%CI [8.9%-15.4%]) in the vitamin D sufficient group.<br><br>Vitamin D deficiency declined with increasing vitamin D dose (especially of vitamin D3). Vitamin D dose was not significantly associated with testing positive for COVID-19 (P=0.18).	The associations observed might not reflect causal effects of vitamin D deficiency on COVID-19. This is because vitamin D deficiency can reflect a range of chronic health conditions or behavioural factors which plausibly decrease the likelihood of treatment of vitamin D

For peer review only - http://bmjopen.bmj.com/site/about/guidelines.xhtml

			2)Likely sufficient (last-level-not-deficient/treatment-not-decreased)<br>3)Uncertain deficiency (last-level-deficient/treatment-increased or last-level-not-deficient/treatment-decreased)<br><br>by age (<50, ≥50), gender (Male/Female), race (White, other than White), ethnicity (Hispanic, not Hispanic), body mass index, employee status, comorbidity indicators (e.g. hypertension)			deficiency and increase COVID-19 risk.
Notari, A. and G. Torrieri (2020)[5]	Correlational study<br><br>126 countries, Only 50 countries for vitamin D	The number of cases follows in its early stages an almost exponential expansion. A starting point in each country was chosen: the first day di with 30 cases and fitted for 12 days. Thus, capturing the early exponential growth.<br><br>Countries with too small total population (less than 300 thousands inhabitants) were excluded.	They analysed risk factors correlated with the initial transmission growth rate of COVID-19<br><br>Average annual level of serum Vitamin D and the seasonal level<br><br>The seasonal level is defined as: the amount during March or during winter for northern hemisphere, or during summer for southern hemisphere or the annual level for countries with little seasonal variation.	Growth rate of COVID-19	They looked for linear correlations of the exponents with other variables, for a sample of 126 countries.<br><br>They found a positive correlation, i.e. faster spread of COVID-19, with high confidence level with the following variables, with respective p-value: low Temperature (4.10^{-7}), high ratio of old vs. working-age people (3.10^{-6}), life expectancy (8.10^{-6}), number of international tourists (1.10^{-5}), earlier epidemic starting date di (2.10^{-5}), high level of physical contact in greeting habits (6.10^{-5}), lung cancer prevalence (6.10^{-5}), obesity in males (1.10^{-4}), share of population in urban areas (2.10^{-4}), cancer prevalence (3.10^{-4}), alcohol consumption (0.0019), daily smoking prevalence (0.0036), UV index (0.004, smaller sample, 73 countries), low Vitamin D serum levels (0.002-0.006, smaller sample, 50 countries). There is highly significant correlation also with blood type. Also, positive correlation with moderate CI (p-value of 0.02-0.03) with: CO2/SO emissions, type-1 diabetes in children, and low vaccination coverage for Tuberculosis (BCG).	The dataset for the annual vitamin D was built with the available literature, which is quite inhomogeneous.<br><br>The dataset for the seasonal levels is more restricted. This is because the relative literature is less complete. So, for this the authors have included only 42 countries.

For peer review only

					Vitamin D is not highly correlated with UV index due to different food consumption in different countries.	
Raisi-Estabragh, Z., et al. (2020)[6]	Retrospective cohort study<br><br>UK Biobank	4,510 UK participants tested for COVID-19.<br><br>Latest data release (29/05/2020) includes test results from 16/03/2020 to 18/05/2020.<br><br>COVID-19 positive cases (n 1,326) Mean age 68.11 (SD 9.23)<br><br>COVID-19 negative controls (n 3,184) Mean age 68.91 (SD 8.72)	Serum 25(OH) D levels nmol/L<br><br>Multivariate logistic regression models by age, gender (Male/Female), ethnicity (Caucasian (any White background) and non-Caucasian: Black, Asian, Chinese) to test whether addition of: 1)cardio metabolic factors (e.g. hypertension, body mass index); 2) 25(OH)-vitamin D; 3) poor diet; 4) Townsend deprivation score; 5) housing; or 6)behavioural factors attenuated sex/ethnicity associations with COVID-19 status	COVID-19 test result<br><br>Greater risk of severe COVID-19	Over-representation of men and non-White ethnicities in the COVID-19 positive group.<br><br>Non-Whites had, on average, poorer cardio metabolic profile, lower 25(OH)-vitamin D, greater material deprivation, and were more likely to live in larger households and flats.<br><br>Male sex, non-White ethnicity, higher body mass index, Townsend deprivation score, and household overcrowding were independently associated with significantly greater odds of COVID-19. The pattern of association was consistent for men and women; cardio metabolic, socio-demographic and behavioural factors did not attenuate sex/ethnicity associations.	UK Biobank baseline samples collected in 2006-2010.<br><br>Aggregating all Black and Minority Ethnic (BAME) populations into one cohort might overlook important differences between non-Caucasian ethnicities.<br><br>The current dataset does not allow assessment of specific COVID-19 health outcomes.
Tan, C. W., et al. (2020)[7]	Cohort observational study<br><br>A tertiary academic hospital, Singapore	All 43 consecutive hospitalized COVID-19 patients aged 50 and above.<br><br>Between 15 January and 15 April 2020.	DMB = a single daily oral dose of vitamin D3 1000 IU, magnesium 150mg and vitamin B12 500mcg for up to 14 days<br><br>Adjusted for age, gender and comorbidities	Deterioration post-DMB administration leading to any form of oxygen therapy and/or intensive care	Duration of therapy: days, Median 5 (IQR 4-7)<br><br>Significantly fewer DMB patients than controls required initiation of oxygen therapy subsequently throughout their hospitalization (17.6% vs 61.5%, P=0.006).<br><br>On univariate analysis, increasing age and presence of comorbidities were associated	Small sample size, and the lack of systematic biologic measures to support their findings.

For peer review only - http://bmjopen.bmj.com/site/about/guidelines.xhtml

		17 patients received Vitamin D, Magnesium, Vitamin B12 (DMB): Mean age 58.4 (SD 7.0)<br><br>26 patients did not: Mean age 64.1 (SD 7.9)		support for COVID-19 patients	with significantly higher OR for oxygen therapy, while exposure to DMB therapy was associated with a significantly improved OR 0.13 (95% CI: 0.03 – 0.59, P=0.008) .<br><br>On multivariate analysis, increasing age was associated with significantly higher OR for oxygen therapy, while exposure to DMB therapy was associated with a significantly improved OR 0.15 (95% CI: 0.025 – 0.93, P=0.041).	

For peer review only

5. Risk of bias of included studies

Risk of bias assessment using the Downs and Black Checklist[8]

Study	Quality score	Reviewer notes
Hastie et al., 2020[9]	14/20<br><br>Seven domains were not applicable and therefore not assessed, 2 reporting, 1 external validity 3 internal validity (bias) and 1 internal validity (confounding).	The study could not be scored for 3 questions as we were unable to determine; 1) the representativeness of the subjects who were prepared to participate from entire population from which they were recruited, 2) whether losses to follow-up were taken into account as patients lost to follow-up were not reported and 3) whether the study had sufficient power to detect a clinically important effect.<br><br>The study did not score a point for 3 questions; 1) providing the number and a description of the characteristics of patients lost to follow-up, 2) stating whether study subjects in different intervention groups we recruited over the same period of time and 3) for assignment concealment as it was a non-randomised study. The study scored partially (only 1 point not two) for clearly described distributions of principal confounders in each group of subjects to be compared.
D'Avolio et al, 2020[10]	13/15<br><br>Twelve domains were not applicable therefore not assessed, 3 reporting, 1 external validity, 4 internal validity (bias) and 4 internal validity (confounding).	The study could not be scored for the 'power' domain as we were unable to determine from the article whether the study had sufficient power to detect a clinically important effect where the probability value for a difference being due to chance is less than 5%.<br><br>The study did not score 1 point in the 'external validity' domain as those subjects who were prepared to participate were not representative of the entire population from which they were recruited.
Fasano et al., 2020[11]	12/17<br>Ten domains were not applicable therefore not assessed, 3 reporting, 1 external validity, 4 internal validity (bias) and 2 internal validity (confounding).	The study could not be scored for 4 items, the 'power' domain and one question in the 'Internal validity - confounding (selection bias)' as the study did not specify the time period over which patients were recruited. It could also not be scored for 2 questions in the 'External validity domain', 1) the representativeness of the subjects asked to participate in the study compared to the entire population from which they were recruited and 2) the representativeness of those subjects who were prepared to participate compared to the entire population from which they were recruited.<br><br>The study did not score 1 point as the effect of the main confounders was not investigated or confounding was demonstrated but no adjustment was made in the final analyses. The study scored two points for presentation of potential confounders.

For peer review only - http://bmjopen.bmj.com/site/about/guidelines.xhtml

Ilie et al., 2020[12]	4/20<br>Seven domains were not applicable and therefore not assessed, 1 reporting, 1 external validity, 3 internal validity (bias), 1 internal validity (confounding) and 1 for power.	The study could not be scored for 9 questions. Two in the 'reporting' domain, 1) interventions of interest not clearly described, 2) the main findings of the study are not clearly described. Two 'External validity' questions 1) the representativeness of the subjects asked to participate in the study compared to the entire population from which they were recruited and 2) the representativeness of those subjects who were prepared to participate compared to the entire population from which they were recruited. Two 'Internal validity – bias' domain questions 1) all analyses that had not been planned at the outset of the study were not clearly indicated (results of the study based on "data dredging", were not made clear), and 2) it was not clear is the statistical techniques used were appropriate to the data. Three 'Internal validity - confounding (selection bias)' domain questions, 1) no information provided concerning the source of patients included in the study 2) does not specify the time period over which patients were recruited, and 3) the numbers of patients lost to follow-up are not reported.<br><br>The study did not score 7 points for the following; 3 reporting issues 1) no description of the characteristics of participants included in the study 2) no description of the distributions of principal confounders in each group of subjects to be compared, and 3) no description of the characteristics of patients lost to follow-up. Two internal validity bias issues 1) differences in follow-up were ignored and 2) no evidence that the main measure used were accurate (valid and reliable).
**Note:** For each included study, the maximum possible quality score was dependent on which domains could be assessed based on the study design. The higher the score assigned to a study, the lower the risk of bias. For example, Hastie et al. 2020[9] was assigned a score of 14 out of a maximum possible score of 20, suggesting good quality and therefore low risk of bias compared to the other studies.		

References

1. Darling AL, Ahmadi KR, Ward KA, et al. Vitamin D status, body mass index, ethnicity and COVID-19: initial analysis of the first-reported UK Biobank COVID-19 positive cases (n 580) compared with negative controls (n 723). *medRxiv* 2020. doi: 10.1101/2020.04.29.20084277
2. De Smet D, De Smet K, Herroelen P, Gryspeerdt S, Martens GA. Vitamin D deficiency as risk factor for severe COVID-19: a convergence of two pandemics. *medRxiv* 2020. doi: 10.1101/2020.05.01.20079376
3. Lau FH, Majumder R, Torabi R, et al. Vitamin D insufficiency is prevalent in severe COVID-19. *medRxiv* 2020. doi: 10.1101/2020.04.24.20075838

For peer review only - http://bmjopen.bmj.com/site/about/guidelines.xhtml

4. Meltzer DO, Best TJ, Zhang H, Vokes T, Arora V, Solway J. Association of vitamin D deficiency and treatment with COVID-19 incidence. *medRxiv* 2020. doi: 10.1101/2020.05.08.20095893
5. Notari A, Torrieri G. COVID-19 transmission risk factors. *medRxiv* 2020. doi: 10.1101/2020.05.08.20095083
6. Raisi-Estabragh Z, McCracken C, Bethell MS, et al. Greater risk of severe COVID-19 in non-White ethnicities is not explained by cardiometabolic, socioeconomic, or behavioural factors, or by 25(OH)-vitamin D status: study of 1,326 cases from the UK Biobank. *medRxiv* 2020. doi: 10.1101/2020.06.01.20118943
7. Tan CW, Ho LP, Kalimuddin S, et al. A cohort study to evaluate the effect of combination vitamin D, magnesium and vitamin B12 (DMB) on progression to severe outcome in older COVID-19 patients. *medRxiv* 2020. doi: 10.1101/2020.06.01.20112334
8. Downs SH, Black N. The feasibility of creating a checklist for the assessment of the methodological quality both of randomised and non-randomised studies of health care interventions. *J Epidemiol Community Health* 1998;52(6):377-84. doi: 10.1136/jech.52.6.377
9. Hastie CE, Mackay DF, Ho F, et al. Vitamin D concentrations and COVID-19 infection in UK Biobank. *Diabetes Metab Syndr* 2020;14(4):561-65. doi: 10.1016/j.dsx.2020.04.050
10. D'Avolio A, Avataneo V, Manca A, et al. 25-hydroxyvitamin D concentrations are lower in patients with positive PCR for SARS-CoV-2. *Nutrients* 2020;12(5):1359. doi: 10.3390/nu12051359
11. Fasano A, Cereda E, Barichella M, et al. COVID-19 in Parkinson's Disease Patients Living in Lombardy, Italy. *Mov Disord* 2020. doi: 10.1002/mds.28176
12. Ilie PC, Stefanescu S, Smith L. The role of vitamin D in the prevention of coronavirus disease 2019 infection and mortality. *Aging Clin Exp Res* 2020. doi: 10.1007/s40520-020-01570-8

For peer review only

For peer review only - http://bmjopen.bmj.com/site/about/guidelines.xhtml

PRISMA 2009 Checklist

Section/topic	#	Checklist item	Reported on page #
**TITLE**			
Title	1	Identify the report as a systematic review, meta-analysis, or both.	1
**ABSTRACT**			
Structured summary	2	Provide a structured summary including, as applicable: background; objectives; data sources; study eligibility criteria, participants, and interventions; study appraisal and synthesis methods; results; limitations; conclusions and implications of key findings; systematic review registration number.	3
**INTRODUCTION**			
Rationale	3	Describe the rationale for the review in the context of what is already known.	5
Objectives	4	Provide an explicit statement of questions being addressed with reference to participants, interventions, comparisons, outcomes, and study design (PICOS).	6
**METHODS**			
Protocol and registration	5	Indicate if a review protocol exists, if and where it can be accessed (e.g., Web address), and, if available, provide registration information including registration number.	6
Eligibility criteria	6	Specify study characteristics (e.g., PICOS, length of follow-up) and report characteristics (e.g., years considered, language, publication status) used as criteria for eligibility, giving rationale.	7
Information sources	7	Describe all information sources (e.g., databases with dates of coverage, contact with study authors to identify additional studies) in the search and date last searched.	6
Search	8	Present full electronic search strategy for at least one database, including any limits used, such that it could be repeated.	Supp appendix 1
Study selection	9	State the process for selecting studies (i.e., screening, eligibility, included in systematic review, and, if applicable, included in the meta-analysis).	7 and supp appendix 2
Data collection process	10	Describe method of data extraction from reports (e.g., piloted forms, independently, in duplicate) and any processes for obtaining and confirming data from investigators.	8
Data items	11	List and define all variables for which data were sought (e.g., PICOS, funding sources) and any assumptions and simplifications made.	8
Risk of bias in individual studies	12	Describe methods used for assessing risk of bias of individual studies (including specification of whether this was done at the study or outcome level), and how this information is to be used in any data synthesis.	8

For peer review only

For peer review only - http://bmjopen.bmj.com/site/about/guidelines.xhtml

PRISMA 2009 Checklist

Summary measures	13	State the principal summary measures (e.g., risk ratio, difference in means).	8
Synthesis of results	14	Describe the methods of handling data and combining results of studies, if done, including measures of consistency (e.g., I^2) for each meta-analysis.	8

Section/topic	#	Checklist item	Reported on page #
Risk of bias across studies	15	Specify any assessment of risk of bias that may affect the cumulative evidence (e.g., publication bias, selective reporting within studies).	n/a
Additional analyses	16	Describe methods of additional analyses (e.g., sensitivity or subgroup analyses, meta-regression), if done, indicating which were pre-specified.	8
**RESULTS**			
Study selection	17	Give numbers of studies screened, assessed for eligibility, and included in the review, with reasons for exclusions at each stage, ideally with a flow diagram.	8
Study characteristics	18	For each study, present characteristics for which data were extracted (e.g., study size, PICOS, follow-up period) and provide the citations.	8 and supp appendix 3
Risk of bias within studies	19	Present data on risk of bias of each study and, if available, any outcome level assessment (see item 12).	8/9 supp appendix 4
Results of individual studies	20	For all outcomes considered (benefits or harms), present, for each study: (a) simple summary data for each intervention group (b) effect estimates and confidence intervals, ideally with a forest plot.	8/9/10 and supp appendix 3
Synthesis of results	21	Present results of each meta-analysis done, including confidence intervals and measures of consistency.	n/a
Risk of bias across studies	22	Present results of any assessment of risk of bias across studies (see Item 15).	n/a
Additional analysis	23	Give results of additional analyses, if done (e.g., sensitivity or subgroup analyses, meta-regression [see Item 16]).	10
**DISCUSSION**			
Summary of evidence	24	Summarize the main findings including the strength of evidence for each main outcome; consider their relevance to key groups (e.g., healthcare providers, users, and policy makers).	11/12/13
Limitations	25	Discuss limitations at study and outcome level (e.g., risk of bias), and at review-level (e.g., incomplete retrieval of identified research, reporting bias).	13

For peer review only

For peer review only - http://bmjopen.bmj.com/site/about/guidelines.xhtml

PRISMA 2009 Checklist

Conclusions	26	Provide a general interpretation of the results in the context of other evidence, and implications for future research.	13/14
**FUNDING**			
Funding	27	Describe sources of funding for the systematic review and other support (e.g., supply of data); role of funders for the systematic review.	15

From: Moher D, Liberati A, Tetzlaff J, Altman DG, The PRISMA Group (2009). Preferred Reporting Items for Systematic Reviews and Meta-Analyses: The PRISMA Statement. PLoS Med 6(7): e1000097. doi:10.1371/journal.pmed1000097

For more information, visit: www.prisma-statement.org.

For peer review only

For peer review only - http://bmjopen.bmj.com/site/about/guidelines.xhtml

BMJ Open

Association between vitamin D supplementation or serum vitamin D level and susceptibility to SARS-CoV-2 infection or COVID-19 including clinical course, morbidity and mortality outcomes? A systematic review.

Journal:	*BMJ Open*
Manuscript ID	bmjopen-2020-043737.R3
Article Type:	Original research
Date Submitted by the Author:	20-Apr-2021
Complete List of Authors:	Grove, Amy; Warwick Life Sciences, Division of Health Sciences, Warwick Medical School<br>Osokogu, Osemeke; University of Warwick, Warwick Medical School<br>Al-Khudairy, Lena; University of Warwick Warwick Medical School, Population, evidence and technology<br>Mehrabian, Amin; University of Warwick, Warwick Medical School; University of Medical Sciences, 2) Department of Pharmaceutical Nanotechnology<br>Zanganeh, Mandana; University of Warwick, Warwick Medical School<br>Brown, Anna; University of Warwick, Warwick Medical School<br>Court, Rachel; University of Warwick, Health Sciences<br>Taylor-Phillips, Sian; University of Warwick, Warwick Medical School<br>Uthman, Olalekan; University of Warwick, Warwick-Centre for Applied Health Research (WCAHRD)<br>McCarthy, Noel; University of Warwick, Warwick Medical School<br>Kumar, Sudhesh; University of Warwick, Medical School<br>Clarke, Aileen; University of Warwick, Division of Health Sciences
**Primary Subject Heading**:	Public health
Secondary Subject Heading:	Infectious diseases
Keywords:	COVID-19, Public health < INFECTIOUS DISEASES, NUTRITION & DIETETICS

SCHOLARONE™
Manuscripts

I, the Submitting Author has the right to grant and does grant on behalf of all authors of the Work (as defined in the below author licence), an exclusive licence and/or a non-exclusive licence for contributions from authors who are: i) UK Crown employees; ii) where BMJ has agreed a CC-BY licence shall apply, and/or iii) in accordance with the terms applicable for US Federal Government officers or employees acting as part of their official duties; on a worldwide, perpetual, irrevocable, royalty-free basis to BMJ Publishing Group Ltd ("BMJ") its licensees and where the relevant Journal is co-owned by BMJ to the co-owners of the Journal, to publish the Work in this journal and any other BMJ products and to exploit all rights, as set out in our licence.

The Submitting Author accepts and understands that any supply made under these terms is made by BMJ to the Submitting Author unless you are acting as an employee on behalf of your employer or a postgraduate student of an affiliated institution which is paying any applicable article publishing charge ("APC") for Open Access articles. Where the Submitting Author wishes to make the Work available on an Open Access basis (and intends to pay the relevant APC), the terms of reuse of such Open Access shall be governed by a Creative Commons licence – details of these licences and which Creative Commons licence will apply to this Work are set out in our licence referred to above.

Other than as permitted in any relevant BMJ Author's Self Archiving Policies, I confirm this Work has not been accepted for publication elsewhere, is not being considered for publication elsewhere and does not duplicate material already published. I confirm all authors consent to publication of this Work and authorise the granting of this licence.

For peer review only

Title page

Title

Association between vitamin D supplementation or serum vitamin D level and susceptibility to SARS-CoV-2 infection or COVID-19 including clinical course, morbidity and mortality outcomes? A systematic review.

Author's names

Amy Grove (0000-0002-8027-7274), Osemeke Osokogu, Lena Al-Khudairy, Amin Mehrabian, Mandana Zanganeh, Anna Brown, Rachel Court, Sian Taylor-Phillips, Olalekan Uthman, Noel McCarthy, Sudhesh Kumar, Aileen Clarke (0000-0001-8299-3146).[1]

Address for each author
Author's names and positions

1) Warwick Medical School, University of Warwick, Coventry, CV4 7AL, UK

Dr Amy Grove
Associate Professor of Health Technology Assessment and Implementation Science

Dr Osemeke Osokogu
Research Fellow

Dr Lena Al-Khudairy
Senior Research Fellow

Dr Mandana Zanganeh
Research Fellow

Mrs Anna Brown
Information Specialist

Mrs Rachel Court
Information Specialist

Professor Sian Taylor-Phillips
Professor of Population Health

Professor Olalekan Uthman

For peer review only

For peer review only - http://bmjopen.bmj.com/site/about/guidelines.xhtml

Professor of Evidence Synthesis
Professor Noel McCarthy
Professor of Public Health

Professor Sudhesh Kumar
Dean of Medicine

Professor Aileen Clarke
Professor of Public Health and Health Services Research

2) Department of Pharmaceutical Nanotechnology, School of Pharmacy, Mashhad University of Medical Sciences, Mashhad, Iran

Mr Amin Mehrabian
Research Fellow

Corresponding Author

Dr Amy Grove, Room A101, Division of Health Sciences, Warwick Medical School, University of Warwick, Coventry, CV4 7AL
Email: A.L.Grove@warwick.ac.uk

For peer review only

Abstract

Objective: To systemically review and critically appraise published studies of the association between vitamin D supplementation or serum vitamin D level and susceptibility to SARS-CoV-2 infection or COVID-19, including clinical course, morbidity and mortality outcomes.

Design: Systematic review.

Data sources: MEDLINE (OVID), Embase (OVID), Cochrane Central Register of Controlled Trials, MedRxiv and BioRxiv preprint databases. COVID-19 databases of the WHO, Cochrane, CEBM Oxford, and Bern University up to 10 June 2020.

Study selection: Studies which assessed Vitamin D supplementation and/or Low Serum Vitamin D in patients acutely ill with, or at risk of severe betacoronavirus infection (SARS-CoV, MERS-CoV, SARS-CoV-2).

Data extraction: Two authors independently extracted data using a predefined data extraction form and assessed risk of bias using the Downs and Black Quality Assessment Checklist.

Results: Searches elicited 449 papers, 59 studies were eligible full text assessment and four met the eligibility criteria of this review. The four studies were narratively synthesised and included: 1) a cross-sectional study (n=107) suggesting an inverse association between serum vitamin D and SARS-CoV-2, 2) a retrospective cohort study (348,598 participants, 449 cases) in which univariable analysis showed that vitamin D protects against COVID-19, 3) an ecological country level study demonstrating a negative correlation between vitamin D and COVID-19 case numbers and mortality, and 4) a case-control survey (n=1,486) showing cases with confirmed/probable COVID-19 reported lower vitamin D supplementation. All studies were at high/unclear risk of bias.

Conclusion: There is no robust evidence of a negative association between vitamin D and COVID-19. No relevant randomised control trials were identified and there is no robust peer reviewed published evidence of association between Vitamin D levels and severity of symptoms or mortality due to COVID-19. Guideline producers should acknowledge that benefits of vitamin D supplementation in COVID-19 are as yet unproven despite increasing interest.

For peer review only - http://bmjopen.bmj.com/site/about/guidelines.xhtml

Strengths and limitations of this study

- The strengths of this systematic review include that it is reported in accordance with the Preferred Reporting Items for Systematic Reviews and Meta-Analyses (PRISMA) checklist.
- The review was conducted by two independent reviewers to ensure robustness of this work.
- We searched multiple living systematic review databases to enable us to capture publications in a fast moving field of research.
- The limitations of the study relate to the small amount of evidence available which was, at risk of bias and which limits the inferences that can be drawn.
- The review was restricted to the English language, therefore non English language papers may have been missed.

For peer review only

For peer review only - http://bmjopen.bmj.com/site/about/guidelines.xhtml

Introduction

COVID-19, a novel viral infection caused by Severe Acute Respiratory Syndrome Coronavirus two (SARS-CoV-2) was declared a pandemic by The World Health Organization (WHO) on 11 March 2020.[1] Mild COVID-19 may manifest as high temperature, a continuous cough and a loss of or change in sense of smell or taste.[2 3] However, more severe and critical cases can result in inflammation of the lungs, low oxygen levels and acute respiratory distress syndrome.[4] Interest is mounting regarding the association of vitamin D supplementation or level with susceptibility to COVID-19 due to the recognised modulating effects of vitamin D on the immune system and immune response.

Vitamin D can modulate the immune system through highly expressed receptors in most non-skeletal tissues.[5 6] Two of the most common analogues of vitamin D which are found in food and used as a dietary supplement are D_2 (ergocalciferol) and D_3 (cholecalciferol, also made by the skin when exposed to sunlight).[7] Both D_2 and D_3 can be hydroxylated by liver enzymes CYP2R1 and CYP27A1 to form calcidiol (25(OH)D). The active metabolite of vitamin D, calcitriol (1α,25(OH)2D), results from the action of CYP27B enzyme on calcidiol. CYP27B is found in several tissues including the kidney, skin, bones, and immune system.[8 9] Tumour necrosis factor α (TNF α) and interferon (IFNγ) are examples of inflammatory cytokines that stimulate the CYP27B enzymes of the immune system.[10-20] Vitamin D can interact with both the innate and cellular immune systems through these mechanisms.

Current Public Health England (PHE),[21] National Institutes of Health[22] and European Food Safety Authority[23] recommendations highlight the importance of vitamin D to population health. Vitamin D deficiency is defined as less than 25 nmol/L (10ng/ml) measured in blood serum.[21] UK guideline recommendations suggest that people take a supplement of 10 micrograms of vitamin D per day during the winter months or throughout the year if they do not spend time outdoors or if they cover the majority of their skin when outside.[21] Published editorials, journal commentaries [24-29] and news media reports [30-32] suggest that individuals with low blood serum concentrations of vitamin D might be at higher risk of infection with COVID-19, or upon infection have worse outcomes than individuals with normal/high serum vitamin D.[33]

Several observational studies have reported associations between low serum vitamin D and chronic[34] and acute conditions such as susceptibility to acute respiratory tract infections (RTI).[35-37] Most recently, Martineau and colleagues (2017) conducted a systematic review and meta-analysis of individual participant data from randomised controlled trials (RCTs) to assess the overall effect of vitamin D supplementation on risk of acute RTI.[38] They reported vitamin D supplementation to be safe while protecting against acute RTI overall (adjusted odds ratio 0.88, 95% confidence interval 0.81 to 0.96; P for heterogeneity <0.001). Patients very deficient in vitamin D benefited the most (adjusted odds ratio 0.75, 0.60 to 0.95; p for

For peer review only - http://bmjopen.bmj.com/site/about/guidelines.xhtml

interaction=0.006).[38] Critiques of this review have suggested that the findings should be interpreted as hypothesis generating only, as the results are heterogeneous and not sufficiently applicable to the general population.[39] Recent rapid reviews of vitamin D for treatment or prevention in COVID-19 reported no evidence that vitamin D deficiency predisposes to COVID-19, or that vitamin D supplementation is effective in prevention or treatment of COVID-19.[40 41] However, data sources included in the rapid review were limited.[42] Given the remaining uncertainty, it is timely to systematically review and critically appraise all peer reviewed published evidence to assess the association of vitamin D supplementation or level with susceptibility to COVID-19 including clinical course, morbidity and mortality outcomes.

Methods

Protocol registration

The methods were prespecified in a protocol that was registered with the PROSPERO International Prospective Register of Systematic Reviews (https://www.crd.york.ac.uk/prospero/display_record.php?ID=CRD42020182876). Research ethics committee approval was not required for this study.

We undertook a systematic review to answer the following question: Is vitamin D supplementation or level associated with susceptibility to severe betacoronavirus infection (Severe Acute Respiratory Syndrome [SARS-CoV], Middle East Respiratory Syndrome [MERSCoV], Severe Acute Respiratory Syndrome two [SARS-CoV-2]) including clinical course, morbidity and mortality outcomes?

Our review was conceptualised and written in accordance with the PRISMA statement.[43]

Data sources and search

The search strategy was developed by the information specialists in collaboration with the research team and clinical advisors. We searched MEDLINE (OVID interface), Embase (OVID interface), Cochrane Central Register of Controlled Trials, MedRxiv and BioRxiv preprint databases on 6th-8th May 2020. We searched the global research on COVID-19 developed by the WHO,[44] CEBM Oxford,[45] and the living systematic review developed by Bern University[46] on 10 May 2020. We updated the database searches on 10th June 2020 to capture articles which may have been published since the initial search was conducted.

We searched additional resources including relevant systematic reviews (in MEDLINE [OVID interface], Embase [OVID interface] and Cochrane Database of Systematic Reviews, 19th May 2020), relavent refrences and contacted experts for additional evidence. Our full search record is included in the supplementary information.

For peer review only - http://bmjopen.bmj.com/site/about/guidelines.xhtml

Study eligibility

We developed pre-defined study eligibility criteria aligned to the research question (Table 1). We imposed a date restriction of January 2002, to capture all published articles since SARS-CoV was first discovered in Asia in February 2003.[47] We limited to English language only.

Table 1. Study eligibility criteria

Study eligibility criteria
P - Population<br><br>1) Patients acutely ill with betacoronavirus infection [SARS-CoV, MERS-CoV, SARS-CoV-2]<br>2) or at risk of acute illness with betacoronavirus infection<br><br>I - Intervention/exposure<br><br>1) Vitamin D supplementation<br>2) Low Serum vitamin D<br><br>O - Outcomes<br><br>1) Betacoronavirus infection (to include serological evidence of infection or clinically confirmed symptomatic infection);<br>2) Severe betacoronavirus infection (to include patients admitted to hospital or admitted to intensive care); mortality due to betacoronavirus infection<br>3) Mortality due to betacoronavirus infection<br><br>C – Comparators<br><br>1) No vitamin D supplementation<br>2) High or normal serum vitamin D<br><br>S - Study design<br><br>Peer reviewed publications of randomised controlled trials and non-randomised studies were eligible for inclusion; including, non randomised controlled trials, interrupted time series analyses, controlled before-and-after studies, cohort studies, ecological studies, case reports and case series.<br><br>Subgroups<br><br>1. Ethnicity characteristics (White British, all other White, Mixed, Asian, Black, Other)<br>2. Age characteristics (population by five-year age groups)

Article selection

Following the article search, we systematically identified and removed any duplicate citations using EndNote X9 software. Using titles and abstracts, de-duplicated citations were screened by two independent reviewers (OO, MZ, AM, AG) and checked by a third (AC). All articles deemed ineligible were excluded at this stage. We identified and obtained all remaining articles for full text screening, which was performed independently by at least two reviewers against the pre-specified eligibility criteria (Table 1). Where disagreements regarding the inclusion of articles arose, a third reviewer (AC) was consulted to reach a final decision.

For peer review only - http://bmjopen.bmj.com/site/about/guidelines.xhtml

Data extraction

Two reviewers independently (LAK, MZ, OO, AM) extracted data from eligible full-text papers using a prespecified data extraction form. The accuracy of all the data extraction was independently assessed by a third reviewer (AG). Where reported, we sought to extract data from each article relevant to the research question, including details of population, intervention/exposure, comparator, outcomes and any detail related to the two pre-specified subgroups: ethnicity characteristics and age characteristics. Disagreements between reviewers were resolved by discussion and agreement, or via consultation with a third reviewer (AC).

Risk of bias

The included studies had observational study designs aimed at answering a specific question. Therefore, risk of bias of included full-text papers was assessed using the Downs and Black Quality Assessment Checklist.[48] Two reviewers (AM, MZ, OO) independently assessed the risk of bias of the included studies and the accuracy of the assessment was evaluated by a third reviewer (LAK).

Data analysis

We anticipated that identified studies would be too heterogeneous to facilitate pooling of study data and planned a narrative synthesis. Nevertheless, we intended to consider pooling outcomes data in a meta-analysis using a random-effects model if appropriate.

Patient and public involvement

Due to the rapid timeframe of this systematic review it was not possible for our research team to involve patients or the public in the design, conduct, or reporting of our study.

Results

After searching databases, assessing the reference lists of 17 narrative reviews[27 28 33 49-62] and one additional article identified through consultation with clinical experts,[38] we identified 499 citations. Following removal of duplicates and screening of titles and abstracts, we retrieved 59 full-text papers of which four met the full eligibility criteria (see **Error! Reference source not found.**). The electronic supplement includes a list of reasons for excluding studies at full text review. Seven articles closely met the eligibility criteria but were excluded as they were not available as peer reviewed publications at the time of our narrative synthesis, details of these seven studies[63-69] is provided in the electronic supplement.

<Figure 1 approximatley here >

For peer review only - http://bmjopen.bmj.com/site/about/guidelines.xhtml

The charteristics of the four included studies are presented in

For peer review only

For peer review only - http://bmjopen.bmj.com/site/about/guidelines.xhtml

Table 2. All four included studies were conducted in Europe and published in April or May 2020. One study was based on data from UK residents exclusively,[70] another included data on residents in 20 European countries, including the UK.[71] The studies were observational design and no relevant RCT were identified or included in the review. All four studies were at high or unclear risk of bias and scored poorly across several domains of the Downs and Black Quality Assessment Checklist,[48] including external validity, internal validity and power. A prominent issue amongst the included studies was that the authors did not perfom adequate multivariable adjustment to correct for confounding.[72] [73] [74] Ecological bias was present in Ilie et al., 2020[71] which may result from spatial and temporal scale differences between country level mean levels of vitamin D. However, several domains in each risk of bias assessment were not applicable or not reported and therefore, could not be scored using the Downs and Black Quality Assessment Checklist.[48] Detailed risk of bias scores are provided in the electronic supplement.

For peer review only

Table 2. Characteristics of the four included studies

Study	Design/Setting	Population	Exposure/Intervention	Outcomes	Results	Limitations
**Serum vitamin D**						
D'Avolio et al. 2020[73]	Cross-sectional study<br><br>Canton of Tessin, Switzerland	107 patients with data on SARS-CoV-2 and 25(OH)D measurement	Vitamin D analysis, conducted within seven weeks of the SARS-CoV-2 polymerase chain reaction (PCR) result<br><br>Control patients with 25(OH)D data during the same period	SARS-CoV-2 infection	Group 1 comprised 27 patients with positive PCR test results for SARS-CoV-2 while group 2 comprised 80 patients with a negative PCR result for SARS-CoV-2<br><br>Significantly lower 25(OH)D levels (p = 0.004) in SARS-CoV-2 patients even after stratifying patients according to age >70 years	Few patients from a single hospital<br><br>No available clinical information about the severity of COVID-19 symptoms<br><br>No data on other potential confounding variable<br><br>SARS-CoV-2 and the 25(OH)D status were performed on different days
Hastie et al. 2020[70]	Retrospective cohort study<br><br>UK Biobank Cohort including England, Scotland and Wales	502,624 participants aged 37-73 years between 2006 and 2010	Biochemical assay of 25(OH)D, a measure of vitamin D status<br><br>Vitamin D was imputed if it was below or above the limit of detection	Confirmed COVID-19 (at least one positive test result)	Complete data on 348,598 UK Biobank participants<br><br>449 had confirmed COVID-19. Of these, 385 (85.8%) were White compared to 64 (14.2%) non-White (Black, South Asian and others)<br><br>Vitamin D was associated with COVID-19 univariably but not after adjustment for confounders. Ethnicity was associated with COVID-19	UK Biobank is not representative of the general population<br><br>Baseline measurements, including 25(OH)D concentration and health status, were obtained a decade prior to conduct of the study

For peer review only - http://bmjopen.bmj.com/site/about/guidelines.xhtml

Ilie et al. 2020[71]	Ecological study<br><br>20 European countries	Population of 20 included European countries	Mean levels of vitamin D in each country	Cases of COVID-19 per 1 million population in each country<br><br>Deaths from COVID-19 per 1 million population	Negative correlations between mean levels of vitamin D and the number of COVID-19 cases per 1 million, and mortality per 1 million	The number of cases per country is affected by the number of tests performed and by the different measures taken by each country to prevent the spread of infection
**Vitamin D supplementation**						
Fasano et al. 2020[74]	Case-control survey<br><br>A single tertiary centre in Lombardy, Italy	1,486 Parkinson's disease (PD) patients were included in the survey<br><br>1,207 family members (controls)	Vitamin D	'Confirmed' or 'probable' diagnosis of COVID-19	12.4% of PD patients with confirmed or probable COVID-19 had been taking vitamin D<br><br>22.9% of PD patients without COVID-19 had been taking vitamin D	Well-known limitation of a telephone survey<br><br>Community-dwelling PD patients<br><br>Some patients could not be reached possibly due to death from COVID-19<br><br>COVID-19 diagnosis could not be confirmed in many cases<br><br>Younger age of non- PD COVID-19 cases

For peer review only

Serum vitamin D

D'Avolio et al., 2020[73] used a cross sectional design with data on nasopharyngeal swab polymerase chain reaction (PCR) analysis for SARS-CoV-2 and a 25(OH)D measurement taken from patients between 1st March and 14th April 2020. PCR positives (median age = 74 years [IQR 65–81];male = 70.4%) had significantly (P= 0.004) lower serum 25(OH)D levels (median = 11.1 ng/mL [IQR 8.2–21.0]) than PCR negatives (median age = 73 years [IQR 61–82]; male = 48.8%; median 25(OH)D = 24.6 ng/mL [IQR 8.9–30.5]). Although gender and age stratified analysis showed no significant differences, older (>70 years) SARS-CoV-2 positive (n=18) participants had significantly lower median serum 25 (OH) D levels (9.3 ng/mL [IQR 8.1–19.9] than older SARS-CoV-2 negatives (n=43) (23.1 ng/mL [IQR 8.5–31.7]) (P = 0.037).

Hastie et al., 2020[70] is a retrospective cohort study that utilised data from the UK Biobank, [72] using data from 348,598 people with complete information on vitamin D and covariates; 449 people tested positive for COVID-19. COVID-19 positives were older (Median = 49 years; Interquartile Range [IQR] = 40-58) than COVID-19 negatives (Median = 49 years; IQR = 38 - 57) with p-value of <0.05. Multivariable analysis showed that age at assessment (OR = 1.02; 95% CI = 1.00 - 1.03; P= 0.016) and non-White ethnicity (Black OR = 4.30, 95% CI = 2.92 - 6.31, P= < 0.001; South Asian OR = 2.42, 95% CI = 1.50 - 3.93, P= <0.001) were associated with confirmed COVID-19. There was no significant interaction between ethnicity and vitamin D deficiency (OR = 0.90; 95% CI = 0.66 - 1.23; P= 0.515). Median vitamin D concentration at recruitment was lower for people with subsequent confirmed COVID-19 (28.7 [IQR 10.0-43.8] nmol/l) than for other participants (32.7 [IQR 10.0-47.2] nmol/l) (P= <0.01). Although univariable analysis suggested an association between vitamin D and COVID-19 (OR=0.99; 95% CI 0.99 – 0.999; P=0.013), this association became insignificant (OR=1.00; 95% CI = 0.998-1.01; P=0.208) after adjustment for covariates.[70]

Ilie et al., 2020[71] used an ecological study design reporting on 20 European countries as at 8th April 2020; the data pertains to mean levels of vitamin D, cases of COVID-19 per million population and deaths from COVID-19 per million population. The authors performed Pearson Correlation Coefficient Calculations and reported a negative correlation between mean levels of vitamin D (Mean 56.79 nmol/l, SD 10.61) and numbers of cases of COVID-19 per million population in each country (Mean cases 1393.4, SD 1129.984, $r(20) = -0.44$; P = 0.05). Additionally, a negative correlation was reported between mean vitamin D levels and the number of deaths caused by COVID-19 per million population in each country (Mean 80.42, SD 94.61, $r(20) = -0.4378$; P = 0.05). Sweden had the highest mean level of vitamin D (73.5 nmol/l) compared to Spain which had a mean level of 42.5 nmol/l). The number of cases of COVID-19 per million population was 834 in Sweden and 3,137 in Spain. Likewise, at the time of the study, there were 68 deaths from COVID-19 per million population in Sweden and 314 in Spain.

For peer review only - http://bmjopen.bmj.com/site/about/guidelines.xhtml

Vitamin D supplementation

Fasano et al., 2020[74] investigated patients in a case-control phone survey in Lombardy, Italy. COVID-19 diagnosis was confirmed using a nasopharyngeal swab or probable based on : a) presence of persistent COVID-19-related symptoms (≥3 including fever or ≥5 without fever); or b) ≥1 symptom in presence of suggestive chest radiologic signs; and/or c) living with a family member with a confirmed diagnosis of COVID-19. 1,486 participants were included in the survey (32 confirmed COVID-19, 73 probable COVID-19 and 1,381 unaffected). Confirmed/probable COVID-19 cases (mean age = 70.5 [Standard Deviation [SD] = 10.1]; male = 53%) self- reported a significantly lower intake of vitamin D supplementation (12.4%) compared to unaffected cases (22.9%; mean age = 73.0 [SD = 9.5], male = 57%). The age-adjusted OR (OR 0.56 [95% CI = 0.32-0.99], P= 0.048) suggested a protective effect of vitamin D intake.

Subgroup evaluation

We planned to perform subgroup analyses by age and ethnicity. According to Hastie et al., 2020[70] multivariable analysis showed that age at assessment (OR = 1.02; 95% CI = 1.00 - 1.03; P= 0.016) and non-White ethnicity (Black OR = 4.30, 95% CI = 2.92 - 6.31, P= < 0.001; South Asians OR = 2.42, 95% CI = 1.50 - 3.93, P= <0.001) were associated with confirmed COVID-19. However, Hastie et al found no significant interaction between ethnicity and vitamin D deficiency (OR = 0.90; 95% CI = 0.66 - 1.23; P= 0.515).

Discussion

This systematic review of non-randomised studies has shown no robust evidence of a association between vitamin D and COVID-19. We identified four studies for inclusion in a narrative synthesis which were all at high or unclear risk of bias. A univariable analysis of data from the UK Biobank database revealed an association between vitamin D and COVID-19 (OR=0.99; 95% CI 0.99 – 0.999; P=0.013). However, this association became insignificant (OR=1.00; 95% CI=0.998-1.01; P=0.208) after adjustment for 13 other covariates, suggesting that the initial association was due to one or more confounding variables.[70]This view is further strengthened by the demonstration of highly significant associations between age and ethnicity characteristics as predictor variables, and COVID-19 as the outcome variable. Overall, the UK Biobank study showed no effect, however it should be noted that the UK Biobank data included only one measurement of Vitamin D levels taken between 10 and 14 years prior to the outbreak of COVID-19. This is a significant study limitation.

Liu et al., 2020[75] concluded that patients over 60 years experienced more severe manifestations and had longer disease courses of COVID-19 compared to patients below 60 years.[75] And other studies have shown that older (rather than younger) people are more likely to die from COVID-19.[76-79]

For peer review only

Non-White people are known to be more susceptible to COVID-19 and tend to develop worse outcomes,[80] a finding that our review has further substantiated.[70] Ethnicity is a multi-faceted construct that includes genetic make-up, socio-cultural identity and behavioural patterns.[81] It has been shown to be associated with differing susceptibility and treatment outcomes in a number of diseases.[82 83 84] Hastie et al., 2020[70] did not find any interaction between ethnicity and vitamin D deficiency and although Ilie et al 2020[71] identified a relationship, the study is subject to ecological bias. Ilie et al 2020[71] compared vitamin D levels and rates of COVID-19 aross 20 European countries, and therefore many relevant factors were not accounted for in the analysis. Given the findings so far from our review we consider that there is paucity of data on vitamin D levels and morbidity and mortality from COVID-19 and there is no evidence from RCTs on outcomes of vitamin D supplementation on severity of symptoms or mortality to date. However a relationship between ethnicity, vitamin D (serum levels or supplementation) and susceptibility to or severity of COVID-19 cannot yet be ruled out.

Risk of bias assessments demonstrate that all studies were at high or unclear risk of bias. All studies were observational designs and therefore subject to confounding. The persistent calls for high-dose vitamin D supplementation[85] arise from speculation about presumed mechanisms.[86 87] Our systematic review found no robust evidence that low levels of Vitamin D are associated with an increased likelihood of COVID-19. More robust prognostic studies could be combined in a systematic review where a prognostic factor research question is phrased, and considerations of participation, attrition, prognostic factor measurement, confounding measurement and account, outcome measurement, and analysis and reporting are evaluated.

Our systematic review identified no relevant RCTs, nevertheless we are aware of two ongoing RCTs investigating the effects of vitamin D on COVID-19, the ZnD3-CoVici study, France (NCT04351490)[88] and the CoVitTrial, France (NCT04344041).[89] Both trials have an estimated study completion date of July 2020. Inclusion of data from these studies in future systematic reviews and meta-analyses may enable us to potentially draw better stronger conclusions on this topic. Results from the ongoing international VITDALIZE Study (NCT03188796) may also contribute to our understanding of the effect of high dose vitamin D3 on mortality.[90]

Study limitations

We performed a full systematic review of the published evidence available, and simultaneous independent screening, data extraction and risk of bias assessments. However, our study is limited by the small amount of evidence available which was, moreover, at risk of bias. This limits the inferences that can be drawn. Seven eligible studies were excluded because they are not available as peer reviewed publications.[63-69] If published, these seven studies would be included in a future update of this review. A final limitation is that the review was restricted to English language only. Therefore, articles published in other languages may have been excluded.

For peer review only - http://bmjopen.bmj.com/site/about/guidelines.xhtml

Implications for practice

Our review does not provide evidence for or against additional or high dose vitamin D supplementation specifically in relation to COVID-19. Treatment as standard practice for people who are deficient is pre-existing practice across Europe[23] the US[22] and in the UK.[21] Current guidelines from PHE suggest that the entire UK population should take vitamin D supplements to prevent vitamin D deficiency in winter or with inadequate sunlight exposure to sun in summer.[21] This review does not give evidence to drive a change in this current advice. Treatment recommendations for patients should be updated following the publication of results from ongoing and new well designed adequately powered randomised controlled trials

Conclusion

This systematic review identified no robust evidence to enable us to assess an association between vitamin D supplementation or serum vitamin D level with susceptibility to COVID-19 including clinical course, morbidity and mortality outcomes. All studies were at high or unclear risk of bias. Both age and ethnicity were associated with vitamin D levels even after multivariable adjustment. Black and South Asian people had a much higher risk of confirmed COVID-19 compared to White people. However, there was no interaction between the association of ethnicity and vitamin D deficiency with COVID-19. There were no papers reporting association of vitamin D with severity of symptoms or mortality due to COVID-19.

For peer review only

For peer review only - http://bmjopen.bmj.com/site/about/guidelines.xhtml

Acknowledgements:
We thank Mrs Kath Charters, Welsh Ambulance Services NHS Trust for comments on the study design and drafts of this manuscript and for valuable advice and helpful discussions.

Contributorship statement:
SK, AG and AC conceived the study. AG, AC, NMcC, SK, STP and OU designed the study. RC and AB developed the search stratergies, performed all searches and database management and created the bibliography. AG, AC, AM, OO, MZ screened titles and abstracts for inclusion. AG, OO, AM, MZ, LAK, AC screened at full text and extracted and analysed data. OO, AM, MZ, LAK performed risk of bias assessments. AC, SK and NMcC assisted in the interpretation from a clinical perspective. STP, LAK, OU offered technical and methodological support. AG and OO wrote the first draft, all authors revised content. All authors approved the final manuscript. AG and AC are the guarantors. The corresponding author attests that all listed authors meet authorship criteria and that no others meeting the criteria have been omitted.

Copyright/license for publication:
The Corresponding Author has the right to grant on behalf of all authors and does grant on behalf of all authors, a worldwide licence to the Publishers and its licensees in perpetuity, in all forms, formats and media (whether known now or created in the future), to i) publish, reproduce, distribute, display and store the Contribution, ii) translate the Contribution into other languages, create adaptations, reprints, include within collections and create summaries, extracts and/or, abstracts of the Contribution, iii) create any other derivative work(s) based on the Contribution, iv) to exploit all subsidiary rights in the Contribution, v) the inclusion of electronic links from the Contribution to third party material where-ever it may be located; and, vi) licence any third party to do any or all of the above.

Funding:
Dr Amy Grove is supported by the National Institute for Health Research (NIHR) Advanced Fellowship Programme Reference (AF-300060). Dr Taylor-Phillips is supported by an NIHR Career Development Fellowship (CDF-2016-09-018). Prof Clarke and Dr Al-Khudairy are supported by the NIHR Applied Research Centre West Midlands (ARC-WM). All other authors, with the exception of Professor Uthman, Professor Kumar and Professor McCarthy, are supported by the NIHR, Evidence Synthesis Programme (HTA 14.25.05). The views expressed are those of the authors and not necessarily those of the NIHR or the Department of Health and Social Care.

Competing interests:
All authors have completed the ICMJE uniform disclosure form at www.icmje.org/coi_disclosure.pdf and declare: no support from any organisation for the submitted work; no competing interests with regards to the submitted work.

For peer review only - http://bmjopen.bmj.com/site/about/guidelines.xhtml

Ethical approval statement:

Not required. Systematic review - secondary research.

Data sharing statement:

The study protocol is available

Systematic review protocol registration: CRD42020182876 available online via PROSPERO at https://www.crd.york.ac.uk/prospero/display_record.php?ID=CRD42020182876. All included studies are publicly available. Additional data are available upon reasonable request by emailing the corresponding author.

For peer review only

For peer review only - http://bmjopen.bmj.com/site/about/guidelines.xhtml

References

1. World Health Organization. Coronavirus disease (COVID-19) pandemic. 2020. https://www.who.int/emergencies/diseases/novel-coronavirus-2019
2. NHS. Check if you or your child has coronavirus symptoms. 2020. https://www.nhs.uk/conditions/coronavirus-covid-19/symptoms/#symptoms
3. Argenziano MG, Bruce SL, Slater CL, et al. Characterization and clinical course of 1000 patients with coronavirus disease 2019 in New York: retrospective case series. *BMJ* 2020;369:m1996. doi: 10.1136/bmj.m1996
4. Matthay MA, Aldrich JM, Gotts JE. Treatment for severe acute respiratory distress syndrome from COVID-19. *Lancet Respir Med* 2020;8(5):433-34. doi: 10.1016/s2213-2600(20)30127-2
5. Gruber BM. Fenomen witaminy D [The phenomenon of vitamin D]. *Postepy Hig Med Dosw (Online)* 2015;69:127-39
6. Gruber-Bzura BM. Vitamin D and influenza: prevention or therapy? *Int J Mol Sci* 2018;19(8):2419. doi: 10.3390/ijms19082419
7. Holick MF. High prevalence of vitamin D inadequacy and implications for health. *Mayo Clin Proc* 2006;81(3):353-73. doi: 10.4065/81.3.353
8. Sigmundsdottir H, Pan J, Debes GF, et al. DCs metabolize sunlight-induced vitamin D3 to 'program' T cell attraction to the epidermal chemokine CCL27. *Nat Immunol* 2007;8(3):285-93. doi: 10.1038/ni1433
9. Kogawa M, Findlay DM, Anderson PH, et al. Osteoclastic metabolism of 25(OH)-vitamin D3: a potential mechanism for optimization of bone resorption. *Endocrinology* 2010;151(10):4613-25. doi: 10.1210/en.2010-0334
10. Jones G. Vitamin D safety: its mechanisms and application. *Standardy Medyczne, Pediatria* 2012;9:605-09
11. Kim D. The role of vitamin D in thyroid diseases. *Int J Mol Sci* 2017;18(9):1949. doi: 10.3390/ijms18091949
12. Beard JA, Bearden A, Striker R. Vitamin D and the anti-viral state. *J Clin Virol* 2011;50(3):194-200. doi: 10.1016/j.jcv.2010.12.006
13. Liu PT, Stenger S, Li H, et al. Toll-like receptor triggering of a vitamin D-mediated human antimicrobial response. *Science* 2006;311(5768):1770-3. doi: 10.1126/science.1123933
14. Adams JS, Ren S, Liu PT, et al. Vitamin D-directed rheostatic regulation of monocyte antibacterial responses. *J Immunol* 2009;182(7):4289-95. doi: 10.4049/jimmunol.0803736
15. Laaksi I. Vitamin D and respiratory infection in adults. *Proc Nutr Soc* 2012;71(1):90-7. doi: 10.1017/s0029665111003351
16. Sharifi A, Vahedi H, Nedjat S, Rafiei H, Hosseinzadeh-Attar MJ. Effect of single-dose injection of vitamin D on immune cytokines in ulcerative colitis patients: a randomized placebo-controlled trial. *APMIS* 2019;127(10):681-87. doi: 10.1111/apm.12982
17. Cantorna MT, Snyder L, Lin YD, Yang L. Vitamin D and 1,25(OH)2D regulation of T cells. *Nutrients* 2015;7(4):3011-21. doi: 10.3390/nu7043011
18. Jeffery LE, Burke F, Mura M, et al. 1,25-Dihydroxyvitamin D3 and IL-2 combine to inhibit T cell production of inflammatory cytokines and promote development of regulatory

For peer review only

T cells expressing CTLA-4 and FoxP3. *J Immunol* 2009;183(9):5458-67. doi: 10.4049/jimmunol.0803217
19. Lei GS, Zhang C, Cheng BH, Lee CH. Mechanisms of action of vitamin D as supplemental therapy for pneumocystis pneumonia. *Antimicrob Agents Chemother* 2017;61(10):e01226-17. doi: 10.1128/aac.01226-17
20. Colunga Biancatelli RML, Berrill M, Marik PE. The antiviral properties of vitamin C. *Expert Rev Anti Infect Ther* 2020;18(2):99-101. doi: 10.1080/14787210.2020.1706483
21. Scientific Advisory Committee on Nutrition. SACN vitamin D and health report. 21 July 2016. https://www.gov.uk/government/publications/sacn-vitamin-d-and-health-report
22. National Institutes of Health, Office of Dietary Supplements. Vitamin D: fact sheet for health professionals. 24 March 2020. https://ods.od.nih.gov/factsheets/VitaminD-HealthProfessional/#h2
23. EFSA Panel on Dietetic Products, Nutrition and Allergies. Dietary reference values for vitamin D. *EFSA Journal* 2016;14(10):e04547. doi: 10.2903/j.efsa.2016.4547
24. Cao Z, Wu Y, Faucon E, Sabatier JM. SARS-CoV-2 & Covid-19: key-roles of the 'renin-angiotensin' system / vitamin D impacting drug and vaccine developments. *Infect Disord Drug Targets* 2020;20(1). doi: 10.2174/1871526520999200505174704
25. Jakovac H. COVID-19 and vitamin D: is there a link and an opportunity for intervention? *Am J Physiol Endocrinol Metab* 2020;318(5):E589. doi: 10.1152/ajpendo.00138.2020
26. Mitchell F. Vitamin-D and COVID-19: do deficient risk a poorer outcome? *Lancet Diabetes Endocrinol* 2020;8(7):570. doi: 10.1016/S2213-8587(20)30183-2
27. Silberstein M. Vitamin D: a simpler alternative to tocilizumab for trial in COVID-19? *Med Hypotheses* 2020;140:109767. doi: 10.1016/j.mehy.2020.109767
28. Calder PC, Carr AC, Gombart AF, Eggersdorfer M. Optimal nutritional status for a well-functioning immune system is an important factor to protect against viral infections. *Nutrients* 2020;12(4):1181. doi: https://dx.doi.org/10.3390/nu12041181
29. Tian Y, Rong L. Letter: Covid-19, and vitamin D. Authors' reply. *Aliment Pharmacol Ther* 2020;51(10):995-96. doi: 10.1111/apt.15764
30. Busby M. UK public health bodies reviewing vitamin D's effects on coronavirus. The Guardian; 17 June 2020. https://www.theguardian.com/world/2020/jun/17/uk-ministers-order-urgent-vitamin-d-coronavirus-review
31. Coronavirus: Should I start taking vitamin D? BBC News; 18 June 2020. https://www.bbc.co.uk/news/health-52371688
32. Torjesen I. Covid-19: Public health agencies review whether vitamin D supplements could reduce risk. *BMJ* 2020;369:m2475. doi: 10.1136/bmj.m2475
33. Grant WB, Lahore H, McDonnell SL, et al. Evidence that vitamin D supplementation could reduce risk of influenza and COVID-19 infections and deaths. *Nutrients* 2020;12(4):988. doi: 10.3390/nu12040988
34. Parker J, Hashmi O, Dutton D, et al. Levels of vitamin D and cardiometabolic disorders: systematic review and meta-analysis. *Maturitas* 2010;65(3):225-36. doi: 10.1016/j.maturitas.2009.12.013
35. Cannell JJ, Vieth R, Umhau JC, et al. Epidemic influenza and vitamin D. *Epidemiol Infect* 2006;134(6):1129-40. doi: 10.1017/s0950268806007175
36. Jolliffe DA, Griffiths CJ, Martineau AR. Vitamin D in the prevention of acute respiratory infection: systematic review of clinical studies. *J Steroid Biochem Mol Biol* 2013;136:321-9. doi: 10.1016/j.jsbmb.2012.11.017

For peer review only - http://bmjopen.bmj.com/site/about/guidelines.xhtml

37. Nnoaham KE, Clarke A. Low serum vitamin D levels and tuberculosis: a systematic review and meta-analysis. *Int J Epidemiol* 2008;37(1):113-19. doi: 10.1093/ije/dym247
38. Martineau AR, Jolliffe DA, Hooper RL, et al. Vitamin D supplementation to prevent acute respiratory tract infections: systematic review and meta-analysis of individual participant data. *BMJ* 2017;356:i6583. doi: 10.1136/bmj.i6583
39. Bolland MJ, Avenell A. Do Vitamin D supplements help prevent respiratory tract infections? *BMJ* 2017;356:j456. doi: 10.1136/bmj.j456
40. National Institute for Health and Care Excellence. COVID-19 rapid evidence summary: vitamin D for COVID-19. 29 June 2020. https://www.nice.org.uk/advice/es28/chapter/Key-messages
41. Royal Society. Vitamin D and covid-19. 18 June 2020. https://royalsociety.org/-/media/policy/projects/set-c/set-c-vitamin-d-and-covid-19.pdf
42. Lee J, van Hecke O, Roberts N, on behalf of the Oxford COVID-19 Evidence Service Team. Vitamin D: a rapid review of the evidence for treatment or prevention in COVID-19. Centre for Evidence-Based Medicine, University of Oxford; 1 May 2020. https://www.cebm.net/covid-19/vitamin-d-a-rapid-review-of-the-evidence-for-treatment-or-prevention-in-covid-19/
43. Moher D, Liberati A, Tetzlaff J, Altman DG. Preferred reporting items for systematic reviews and meta-analyses: the PRISMA statement. *BMJ* 2009;339:b2535. doi: 10.1136/bmj.b2535
44. World Health Organization. Global research on coronavirus disease (COVID-19) [online database]. 2020. https://www.who.int/emergencies/diseases/novel-coronavirus-2019/global-research-on-novel-coronavirus-2019-ncov
45. Oxford COVID-19 Evidence Service. Centre for Evidence-Based Medicine, University of Oxford; 2020. https://www.cebm.net/oxford-covid-19-evidence-service/
46. Counotte M, Imeri H, Ipekci M, Low N. Living evidence on COVID-19 [online database]. University of Bern Institute of Social and Preventive Medicine; 2020. https://zika.ispm.unibe.ch/assets/data/pub/ncov/
47. Centers for Disease Control and Prevention. CDC SARS response timeline. 2013. https://www.cdc.gov/about/history/sars/timeline.htm
48. Downs SH, Black N. The feasibility of creating a checklist for the assessment of the methodological quality both of randomised and non-randomised studies of health care interventions. *J Epidemiol Community Health* 1998;52(6):377-84. doi: 10.1136/jech.52.6.377
49. Carter SJ, Baranauskas MN, Fly AD. Considerations for obesity, vitamin D, and physical activity amidst the COVID-19 pandemic. *Obesity (Silver Spring)* 2020;28:1176-77. doi: 10.1002/oby.22838
50. Molloy EJ, Murphy N. Vitamin D, covid-19 and children. *Ir Med J* 2020;113(4):64
51. McCartney DM, Byrne DG. Optimisation of vitamin D status for enhanced immuno-protection against covid-19. *Ir Med J* 2020;113(4):P58
52. Adams KK, Baker WL, Sobieraj DM. Myth busters: dietary supplements and COVID-19. *Ann Pharmacother* 2020;54(8):820-26. doi: 10.1177/1060028020928052
53. de Lucena TMC, da Silva Santos AF, de Lima BR, de Albuquerque Borborema ME, de Azevedo Silva J. Mechanism of inflammatory response in associated comorbidities in COVID-19. *Diabetes Metab Syndr* 2020;14(4):597-600. doi: 10.1016/j.dsx.2020.05.025

For peer review only

54. Ghasemian R, Shamshirian A, Heydari K, et al. The role of vitamin D in the age of COVID-19: a systematic review and meta-analysis along with an ecological approach. *medRxiv* 2020. doi: 10.1101/2020.06.05.20123554
55. Hribar CA, Cobbold PH, Church FC. Potential role of vitamin D in the elderly to resist COVID-19 and to slow progression of Parkinson's disease. *Brain Sci* 2020;10(5):284. doi: 10.3390/brainsci10050284
56. Laird E, Rhodes J, Kenny RA. Vitamin D and inflammation: potential implications for severity of Covid-19. *Ir Med J* 2020;113(5):P81
57. McKenna MJ, Flynn MAT. Covid-19, cocooning and vitamin D intake requirements. *Ir Med J* 2020;113(5):P79
58. Ribeiro H, Santana KVS, Oliver SL, et al. Does vitamin D play a role in the management of Covid-19 in Brazil? *Rev Saude Publica* 2020;54:53
59. Speeckaert MM, Delanghe JR. Association between low vitamin D and COVID-19: don't forget the vitamin D binding protein. *Aging Clin Exp Res* 2020. doi: 10.1007/s40520-020-01607-y
60. Suresh PS. Hypovitaminosis D and COVID-19: matter of concern in India? *Indian J Clin Biochem* 2020. doi: 10.1007/s12291-020-00894-6
61. Tan SHS, Hong CC, Saha S, Murphy D, Hui JH. Medications in COVID-19 patients: summarizing the current literature from an orthopaedic perspective. *Int Orthop* 2020. doi: 10.1007/s00264-020-04643-5
62. Zabetakis I, Lordan R, Norton C, Tsoupras A. COVID-19: the inflammation link and the role of nutrition in potential mitigation. *Nutrients* 2020;12(5):1466. doi: 10.3390/nu12051466
63. Lau FH, Majumder R, Torabi R, et al. Vitamin D insufficiency is prevalent in severe COVID-19. *medRxiv* 2020. doi: 10.1101/2020.04.24.20075838
64. Darling AL, Ahmadi KR, Ward KA, et al. Vitamin D status, body mass index, ethnicity and COVID-19: initial analysis of the first-reported UK Biobank COVID-19 positive cases (n 580) compared with negative controls (n 723). *medRxiv* 2020. doi: 10.1101/2020.04.29.20084277
65. De Smet D, De Smet K, Herroelen P, Gryspeerdt S, Martens GA. Vitamin D deficiency as risk factor for severe COVID-19: a convergence of two pandemics. *medRxiv* 2020. doi: 10.1101/2020.05.01.20079376
66. Meltzer DO, Best TJ, Zhang H, Vokes T, Arora V, Solway J. Association of vitamin D deficiency and treatment with COVID-19 incidence. *medRxiv* 2020. doi: 10.1101/2020.05.08.20095893
67. Notari A, Torrieri G. COVID-19 transmission risk factors. *medRxiv* 2020. doi: 10.1101/2020.05.08.20095083
68. Raisi-Estabragh Z, McCracken C, Bethell MS, et al. Greater risk of severe COVID-19 in non-White ethnicities is not explained by cardiometabolic, socioeconomic, or behavioural factors, or by 25(OH)-vitamin D status: study of 1,326 cases from the UK Biobank. *medRxiv* 2020. doi: 10.1101/2020.06.01.20118943
69. Tan CW, Ho LP, Kalimuddin S, et al. A cohort study to evaluate the effect of combination vitamin D, magnesium and vitamin B12 (DMB) on progression to severe outcome in older COVID-19 patients. *medRxiv* 2020. doi: 10.1101/2020.06.01.20112334
70. Hastie CE, Mackay DF, Ho F, et al. Vitamin D concentrations and COVID-19 infection in UK Biobank. *Diabetes Metab Syndr* 2020;14(4):561-65. doi: 10.1016/j.dsx.2020.04.050

For peer review only - http://bmjopen.bmj.com/site/about/guidelines.xhtml

71. Ilie PC, Stefanescu S, Smith L. The role of vitamin D in the prevention of coronavirus disease 2019 infection and mortality. *Aging Clin Exp Res* 2020. doi: 10.1007/s40520-020-01570-8
72. UK Biobank. About UK Biobank. 17 June 2020. https://www.ukbiobank.ac.uk/about-biobank-uk/
73. D'Avolio A, Avataneo V, Manca A, et al. 25-hydroxyvitamin D concentrations are lower in patients with positive PCR for SARS-CoV-2. *Nutrients* 2020;12(5):1359. doi: 10.3390/nu12051359
74. Fasano A, Cereda E, Barichella M, et al. COVID-19 in Parkinson's Disease Patients Living in Lombardy, Italy. *Mov Disord* 2020. doi: 10.1002/mds.28176
75. Liu Y, Mao B, Liang S, et al. Association between age and clinical characteristics and outcomes of COVID-19. *Eur Respir J* 2020;55(5):2001112. doi: 10.1183/13993003.01112-2020
76. Verity R, Okell LC, Dorigatti I, et al. Estimates of the severity of coronavirus disease 2019: a model-based analysis. *Lancet Infect Dis* 2020;20(6):669-77. doi: 10.1016/s1473-3099(20)30243-7
77. Mahase E. Covid-19: death rate is 0.66% and increases with age, study estimates. *BMJ* 2020;369:m1327. doi: 10.1136/bmj.m1327
78. Fulzele S, Sahay B, Yusufu I, et al. COVID-19 virulence in aged patients might be impacted by the host cellular microRNAs abundance/profile. *Aging Dis* 2020;11(3):509-22. doi: 10.14336/ad.2020.0428
79. Koff WC, Williams MA. Covid-19 and immunity in aging populations: a new research agenda. *N Engl J Med* 2020. doi: 10.1056/NEJMp2006761
80. Pan D, Sze S, Minhas JS, et al. The impact of ethnicity on clinical outcomes in COVID-19: a systematic review. *EClinicalMedicine*. doi: 10.1016/j.eclinm.2020.100404
81. Lee C. "Race" and "ethnicity" in biomedical research: how do scientists construct and explain differences in health? *Soc Sci Med* 2009;68(6):1183-90. doi: 10.1016/j.socscimed.2008.12.036
82. Stead WW, Senner JW, Reddick WT, Lofgren JP. Racial differences in susceptibility to infection by Mycobacterium tuberculosis. *N Engl J Med* 1990;322(7):422-7. doi: 10.1056/nejm199002153220702
83. Nahid P, Horne DJ, Jarlsberg LG, et al. Racial differences in tuberculosis infection in United States communities: the coronary artery risk development in young adults study. *Clin Infect Dis* 2011;53(3):291-4. doi: 10.1093/cid/cir378
84. Bime C, Poongkunran C, Borgstrom M, et al. Racial differences in mortality from severe acute respiratory failure in the United States, 2008-2012. *Ann Am Thorac Soc* 2016;13(12):2184-89. doi: 10.1513/AnnalsATS.201605-359OC
85. Lanham-New SA, Webb AR, Cashman KD, et al. Vitamin D and SARS-CoV-2 virus/COVID-19 disease. *BMJ Nutrition, Prevention & Health* 2020:bmjnph-2020-000089. doi: 10.1136/bmjnph-2020-000089
86. Huang F, Zhang C, Liu Q, et al. Identification of amitriptyline HCl, flavin adenine dinucleotide, azacitidine and calcitriol as repurposing drugs for influenza A H5N1 virus-induced lung injury. *PLoS Pathog* 2020;16(3):e1008341. doi: 10.1371/journal.ppat.1008341
87. Lee C. Controversial effects of vitamin D and related genes on viral infections, pathogenesis, and treatment outcomes. *Nutrients* 2020;12(4):962. doi: 10.3390/nu12040962

For peer review only - http://bmjopen.bmj.com/site/about/guidelines.xhtml

88. Impact of zinc and vitamin D3 supplementation on the survival of aged patients infected with COVID-19. NCT04351490. ClinicalTrials.gov, U.S. National Library of Medicine; 2020. https://ClinicalTrials.gov/show/NCT04351490
89. COvid-19 and Vitamin D supplementation: a multicenter randomized controlled trial of high dose versus standard dose vitamin D3 in high-risk COVID-19 Patients (CoVitTrial). NCT04344041. ClinicalTrials.gov, U.S. National Library of Medicine; 2020. https://clinicaltrials.gov/show/NCT04344041
90. The VITDALIZE Study: effect of high-dose vitamin D3 on 28-day mortality in adult critically ill patients. NCT03188796. ClinicalTrials.gov, U.S. National Library of Medicine; 2017. https://ClinicalTrials.gov/show/NCT03188796

Figure legends

Figure 1. PRISMA flow diagram for the selection of studies

For peer review only

For peer review only - http://bmjopen.bmj.com/site/about/guidelines.xhtml

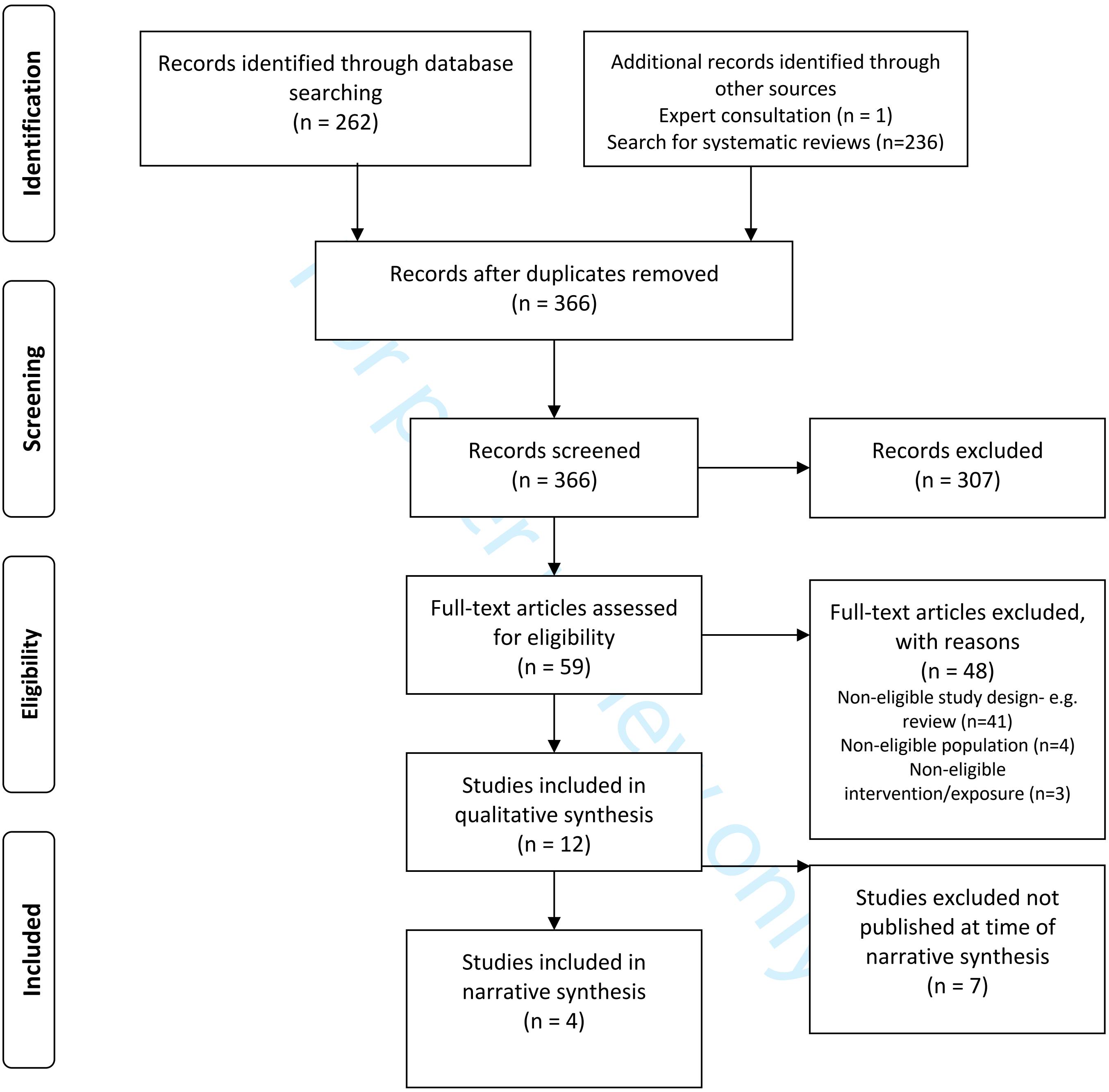
Identification
Screening
Eligibility
Included
Records identified through database searching (n = 262)
Additional records identified through other sources
Expert consultation (n = 1)
Search for systematic reviews (n=236)
Records after duplicates removed (n = 366)
Records screened (n = 366)
Records excluded (n = 307)
Full-text articles assessed for eligibility (n = 59)
Full-text articles excluded, with reasons (n = 48)
Non-eligible study design- e.g. review (n=41)
Non-eligible population (n=4)
Non-eligible intervention/exposure (n=3)
Studies included in qualitative synthesis (n = 12)
Studies excluded not published at time of narrative synthesis (n = 7)
Studies included in narrative synthesis (n = 4)

Supplemental file

Contents

1. Full record of search
2. Full details of the study eligibility criteria
3. List of studies excluded at full text review
4. Articles included at full text, but later excluded at time of narrative synthesis
5. Quality assessment of included studies

For peer review only

1. <u>Full record of search</u>

Medline (Ovid)

Search date: 06/05/2020
Database: Ovid MEDLINE(R) ALL <1946 to May 05, 2020>
Search Strategy:
----
1 exp Vitamin D/ (58492)
2 Vitamin D Deficiency/ (15552)
3 (vitamin D? or vit D? or D vitamin? or ergocalciferol or cholecalciferol or colecalciferol or calciferol or dihydroergocalciferol or sitocalciferol or dihydrotachysterol or dihydroxycholecalciferol? or hydroxycholecalciferol? or "25(OH)D?" or 25OHD? or calcidiol or hydroxyergocalciferol or alfacalcidol or alphacalcidol or "1,25(OH)2D" or hydroxyvitamin D? or dihydroxyvitamin D?).ab,kf,ti. (78232)
4 (paricalcitol or doxercalciferol or doxerocalciferol or calcifediol or calcitriol).ab,kf,ti. (5577)
5 hypovitaminosis D?.ab,kf,ti. (1775)
6 ((D or D2 or D3) adj3 supplement*).ab,kf,ti. (12158)
7 1 or 2 or 3 or 4 or 5 or 6 (92560)
8 coronavirus/ or betacoronavirus/ or betacoronavirus 1/ or coronavirus oc43, human/ or middle east respiratory syndrome coronavirus/ or sars virus/ (7431)
9 coronavirus infections/ or severe acute respiratory syndrome/ (10675)
10 (((corona* or corono*) adj1 (virus* or viral* or virinae*)) or coronavirus* or coronovirus* or coronavirinae* or Coronavirus* or Coronovirus* or Wuhan* or Hubei* or Huanan or "2019-nCoV" or 2019nCoV or nCoV2019 or "nCoV-2019" or "COVID-19" or COVID19 or "CORVID-19" or CORVID19 or "WN-CoV" or WNCoV or "HCoV-19" or HCoV19 or CoV or "2019 novel*" or Ncov or "n-cov" or "SARS-CoV-2" or "SARSCoV-2" or "SARSCoV2" or "SARS-CoV2" or SARSCov19 or "SARS-Cov19" or "SARSCov-19" or "SARS-Cov-19" or Ncovor or Ncorona* or Ncorono* or NcovWuhan* or NcovHubei* or NcovChina* or NcovChinese*).ab,kf,ti. (26891)
11 (severe acute respiratory syndrome or SARS or Middle East respiratory syndrome or MERS).ab,kf,ti. (16560)
12 (betacoronavirus* or betacoronavirinae*).ab,kf,ti. (280)
13 8 or 9 or 10 or 11 or 12 (37180)
14 7 and 13 (32)
15 exp Animals/ (23144176)

16 exp Humans/ (18448248)
17 15 not 16 (4695928)
18 14 not 17 (30)
19 limit 18 to yr="2002 -Current" (30)

Update
Search date: 10/6/2020
Actual databases searched: Ovid MEDLINE All <1946 to June 09, 2020>
Search strategy:
Re-ran search above plus…
20 limit 19 to ed=20200506-20200610 (8)
21 limit 19 to ep=20200506-20200610 (39)
22 limit 19 to dt=20200506-20200610 (43)
23 limit 19 to ez=20200506-20200610 (27)
24 20 or 21 or 22 or 23 (46)

For peer review only

Embase (Ovid)

Search date: 06/05/2020
Database: Embase <1974 to 2020 May 05>
Search Strategy:
----
1 exp vitamin D/ (139781)
2 vitamin D deficiency/ (29333)
3 (vitamin D? or vit D? or D vitamin? or ergocalciferol or cholecalciferol or colecalciferol or calciferol or dihydroergocalciferol or sitocalciferol or dihydrotachysterol or dihydroxycholecalciferol? or hydroxycholecalciferol? or "25(OH)D?" or 25OHD? or calcidiol or hydroxyergocalciferol or alfacalcidol or alphacalcidol or "1,25(OH)2D" or hydroxyvitamin D? or dihydroxyvitamin D?).ab,kw,ti. (112459)
4 (paricalcitol or doxercalciferol or doxerocalciferol or calcifediol or calcitriol).ab,kw,ti. (8478)
5 hypovitaminosis D?.ab,kw,ti. (3012)
6 ((D or D2 or D3) adj3 supplement*).ab,kw,ti. (19177)
7 1 or 2 or 3 or 4 or 5 or 6 (163395)
8 betacoronavirus 1/ or betacoronavirus/ or human coronavirus oc43/ (696)
9 Middle East respiratory syndrome coronavirus/ (2028)
10 sars-related coronavirus/ or sars coronavirus/ (6354)
11 Coronavirinae/ (2231)
12 coronavirus infection/ or middle east respiratory syndrome/ or severe acute respiratory syndrome/ (11950)
13 (((corona* or corono*) adj1 (virus* or viral* or virinae*)) or coronavirus* or coronovirus* or coronavirinae* or Coronavirus* or Coronovirus* or Wuhan* or Hubei* or Huanan or "2019-nCoV" or 2019nCoV or nCoV2019 or "nCoV-2019" or "COVID-19" or COVID19 or "CORVID-19" or CORVID19 or "WN-CoV" or WNCoV or "HCoV-19" or HCoV19 or CoV or "2019 novel*" or Ncov or "n-cov" or "SARS-CoV-2" or "SARSCoV-2" or "SARSCoV2" or "SARS-CoV2" or SARSCov19 or "SARS-Cov19" or "SARSCov-19" or "SARS-Cov-19" or Ncovor or Ncorona* or Ncorono* or NcovWuhan* or NcovHubei* or NcovChina* or NcovChinese*).ab,kw,ti. (27686)
14 (severe acute respiratory syndrome or SARS or Middle East respiratory syndrome or MERS).ab,kw,ti. (17146)
15 (betacoronavirus* or betacoronavirinae*).ab,kw,ti. (275)
16 8 or 9 or 10 or 11 or 12 or 13 or 14 or 15 (40716)

17 7 and 16 (61)
18 exp animal/ (25459151)
19 exp human/ (20834835)
20 18 not 19 (4624316)
21 17 not 20 (58)
22 limit 21 to yr="2002 -Current" (58)

Update
Search date: 10/6/2020
Actual databases searched: Ovid Embase <1974 to 2020 June 09>
Search strategy:
Re-ran search above plus...
22 limit 21 to yr="2002 -Current" (123)
23 limit 22 to dd=20200506-20200610 (39)
24 limit 22 to em=202005-202006 (0)
25 limit 22 to dc=20200506-20200610 (62)
26 23 or 24 or 25 (62)

MedrXiv (searched via Medrxivr https://mcguinlu.shinyapps.io/medrxivr/)

Search date: 07/05/2020
Search Strategy:

Topic 1:

[Vv]itamin D
[Vv]itamin D2
[Vv]itamin D3
calciferol
25OHD
25OHD3
[Hh]ypovitaminosis D

Topic 2:

[Cc]oronavirus
[Cc]orona(\\s)([[:graph:]]+\\s){0,1}virus
[Cc]oronavirinae
[Cc]ovid
COVID
nCoV
NCOV
Ncov
[Nn]-cov
N-COV
2019ncov
2019-ncov
ncov2019
ncov-2019

For peer review only - http://bmjopen.bmj.com/site/about/guidelines.xhtml

SARS
[Ss]evere [Aa]cute [Rr]espiratory [Ss]yndrome
[Mm]iddle [Ee]ast [Rr]espiratory [Ss]yndrome
MERS

Earliest record date
20190101
Latest record date
20200507
Remove older versions of the same record

6 results

Update
Search date: 10/6/2020
Re-ran search above changing record dates as follows:
Earliest record date
20200507
Latest record date
20200610
Remove older versions of the same record

11 results

BioRxiv
https://www.biorxiv.org/

Search date: 07/05/2020

65 Results
for abstract or title "vitamin D" (match phrase words)

22 Results
for full text or abstract or title "ergocalciferol cholecalciferol colecalciferol" (match whole any)

41 Results
for full text or abstract or title "25OHD 25OHD3" (match whole any)

Imported into EndNote and de-duplicated
92 results after deduplication

Searched in Endnote using the following search strategy:
coronavirus or corona or covid or SARS or MERS or betacoronavirus or ncov
Any Field

5 results

Update
Search date: 10/6/2020

For peer review only

1 Results
for abstract or title "vitamin D" (match phrase words) and posted between "07 May, 2020 and 10 Jun, 2020" – *animal study (also in both results sets below) so not exported to EndNote*

3 Results
for full text or abstract or title "ergocalciferol cholecalciferol colecalciferol" (match whole any) and posted between "07 May, 2020 and 10 Jun, 2020" - *2 animal studies and 1 on sertraline in TB*

2 Results
for full text or abstract or title "25OHD 25OHD3" (match whole any) and posted between "07 May, 2020 and 10 Jun, 2020" - *1 animal study, 1 non-clinical / non-coronavirus*

0 results relevant to coronaviruses

Cochrane Library

Search date: 08/05/2020

ID Search Hits
#1 MeSH descriptor: [Vitamin D] explode all trees 5224
#2 MeSH descriptor: [Vitamin D Deficiency] this term only 1226
#3 ((vitamin NEXT D?) or (vit NEXT D?) or (D NEXT vitamin?) or ergocalciferol or cholecalciferol or colecalciferol or calciferol or dihydroergocalciferol or sitocalciferol or dihydrotachysterol or dihydroxycholecalciferol? or hydroxycholecalciferol? or "25(OH)D" or "25(OH)D3" or 25OHD? or calcidiol or hydroxyergocalciferol or alfacalcidol or alphacalcidol or "1,25(OH)2D" or (hydroxyvitamin NEXT D?) or (dihydroxyvitamin NEXT D?)):ti,ab,kw 12959
#4 (paricalcitol or doxercalciferol or doxerocalciferol or calcifediol or calcitriol):ti,ab,kw 2417
#5 hypovitaminosis NEXT D? 303
#6 ((D OR D2 OR D3) NEAR/3 supplement*):ti,ab,kw 5633
#7 #1 or #2 or #3 or #4 or #5 or #6 14461
#8 MeSH descriptor: [Coronavirus] this term only 2
#9 MeSH descriptor: [Betacoronavirus] this term only 2
#10 MeSH descriptor: [Betacoronavirus 1] this term only 0
#11 MeSH descriptor: [Coronavirus OC43, Human] this term only 0
#12 MeSH descriptor: [Middle East Respiratory Syndrome Coronavirus] explode all trees 1
#13 MeSH descriptor: [SARS Virus] this term only 9
#14 MeSH descriptor: [Coronavirus Infections] this term only 137
#15 MeSH descriptor: [Severe Acute Respiratory Syndrome] this term only 107
#16 (((corona* or corono*) near/1 (virus* or viral* or virinae*)) or coronavirus* or coronovirus* or coronavirinae* or Coronavirus* or Coronovirus* or Wuhan* or Hubei* or Huanan or "2019-nCoV" or 2019nCoV or nCoV2019 or "nCoV-2019" or "COVID-19" or COVID19 or "CORVID-19" or CORVID19 or "WN-CoV" or WNCoV or "HCoV-19" or HCoV19 or CoV or "2019 novel*" or Ncov or "n-cov" or "SARS-CoV-2" or "SARSCoV-2" or "SARSCoV2" or "SARS-CoV2" or SARSCov19 or "SARS-Cov19" or "SARSCov-19" or "SARS-Cov-19" or Ncovor or Ncorona* or Ncorono* or NcovWuhan* or NcovHubei* or NcovChina* or NcovChinese*):ti,ab,kw 614
#17 ("severe acute respiratory syndrome" or SARS or "Middle East respiratory syndrome" or MERS):ti,ab,kw 350
#18 (betacoronavirus* or betacoronavirinae*):ti,ab,kw 4

For peer review only - http://bmjopen.bmj.com/site/about/guidelines.xhtml

#19 #8 or #9 or #10 or #11 or #12 or #13 or #14 or #15 or #16 or #17 or #18 798
#20 #7 and #19 3

[all 3 results were from CENTRAL]

Update
Search date: 10/06/2020
Re-ran search exactly as above and retrieved **5** results, all from CENTRAL. All 5 results exported to EndNote for deduplication.

Database of publications (living map of evidence) on coronavirus disease (COVID-19) developed by the University of Bern

Living Evidence on COVID-19
Contributors: Michel Counotte, Hira Imeri, Mert Ipekci, Nicola Low

https://zika.ispm.unibe.ch/assets/data/pub/ncov/

Search date: 10/05/2020 (14,988 entries)

Search: Title, Abstract
Search:

vitamin D 13
vitamin D2 0
vitamin D3 0
ergocalciferol 0
cholecalciferol 0
colecalciferol 0
25(OH)D 0
25OHD 0
25(OH)D3 0
25OHD3 0
hypovitaminosis D 1
Vitamin D Deficiency 1

Oxford COVID-19 Evidence Service
https://www.cebm.net/oxford-covid-19-evidence-service/
The Centre for Evidence-Based Medicine (CEBM) The University of Oxford

Search date: 10/05/2020 (142 articles)
vitamin D 1
vitamin D2 0
vitamin D3 0
ergocalciferol 0
cholecalciferol 0
colecalciferol 0
25(OH)D 0
25OHD 0

For peer review only

25(OH)D3 0
25OHD3 0
hypovitaminosis D 0
Vitamin D Deficiency 0

Database of publications on coronavirus disease (COVID-19) developed by WHO
https://www.who.int/emergencies/diseases/novel-coronavirus-2019/global-research-on-novel-coronavirus-2019-ncov

Search date: 10/05/2020 (15,253 entries)

Search: Title, Abstract, Subject

vitamin D 19
vitamin D2 0
vitamin D3 2
ergocalciferol 0
cholecalciferol 1
colecalciferol 0
25(OH)D 0
25OHD 0
25(OH)D3 0
25OHD3 0
hypovitaminosis D 1
Vitamin D Deficiency 2

Total: 25
After de-duplication: 20

Searches for systematic reviews, for reference checking

Medline

Search date: 19/05/2020
Database: Ovid MEDLINE(R) ALL <1946 to May 18, 2020>
Search Strategy:
----
1 exp Vitamin D/ (58577)
2 Vitamin D Deficiency/ (15588)
3 (vitamin D? or vit D? or D vitamin? or ergocalciferol or cholecalciferol or colecalciferol or calciferol or dihydroergocalciferol or sitocalciferol or dihydrotachysterol or dihydroxycholecalciferol? or hydroxycholecalciferol? or "25(OH)D?" or 25OHD? or calcidiol or hydroxyergocalciferol or alfacalcidol or alphacalcidol or "1,25(OH)2D" or hydroxyvitamin D? or dihydroxyvitamin D?).ab,kf,ti. (78395)
4 (paricalcitol or doxercalciferol or doxerocalciferol or calcifediol or calcitriol).ab,kf,ti. (5588)
5 hypovitaminosis D?.ab,kf,ti. (1780)
6 ((D or D2 or D3) adj3 supplement*).ab,kf,ti. (12198)
7 1 or 2 or 3 or 4 or 5 or 6 (92747)

For peer review only

8 coronavirus/ or betacoronavirus/ or betacoronavirus 1/ or coronavirus oc43, human/ or middle east respiratory syndrome coronavirus/ or sars virus/ (8161)
9 coronavirus infections/ or severe acute respiratory syndrome/ (11614)
10 (((corona* or corono*) adj1 (virus* or viral* or virinae*)) or coronavirus* or coronovirus* or coronavirinae* or Coronavirus* or Coronovirus* or Wuhan* or Hubei* or Huanan or "2019-nCoV" or 2019nCoV or nCoV2019 or "nCoV-2019" or "COVID-19" or COVID19 or "CORVID-19" or CORVID19 or "WN-CoV" or WNCoV or "HCoV-19" or HCoV19 or CoV or "2019 novel*" or Ncov or "n-cov" or "SARS-CoV-2" or "SARSCoV-2" or "SARSCoV2" or "SARS-CoV2" or SARSCov19 or "SARS-Cov19" or "SARSCov-19" or "SARS-Cov-19" or Ncovor or Ncorona* or Ncorono* or NcovWuhan* or NcovHubei* or NcovChina* or NcovChinese*).ab,kf,ti. (31115)
11 (severe acute respiratory syndrome or SARS or Middle East respiratory syndrome or MERS).ab,kf,ti. (17795)
12 (betacoronavirus* or betacoronavirinae*).ab,kf,ti. (294)
13 exp Respiratory Tract Infections/ (356696)
14 (acute respiratory infection* or severe respiratory infection* or acute respiratory tract infection* or severe respiratory tract infection* or influenza or common cold or pneumonia or bronchitis).ab,kf,ti. (234266)
15 8 or 9 or 10 or 11 or 12 or 13 or 14 (503079)
16 7 and 15 (1062)
17 (metaanalys* or "meta analys*" or "meta-analys*").tw. (169008)
18 (systematic* adj3 review*).mp. (200684)
19 meta analysis.pt. (114746)
20 17 or 18 or 19 (301767)
21 16 and 20 (55)

Embase

Search date: 19/05/2020

Database: Embase Classic+Embase <1947 to 2020 Week 20>
Search Strategy:
----
1 exp vitamin D/ (147053)
2 vitamin D deficiency/ (30106)
3 (vitamin D? or vit D? or D vitamin? or ergocalciferol or cholecalciferol or colecalciferol or calciferol or dihydroergocalciferol or sitocalciferol or dihydrotachysterol or dihydroxycholecalciferol? or hydroxycholecalciferol? or "25(OH)D?" or 25OHD? or calcidiol or hydroxyergocalciferol or alfacalcidol or alphacalcidol or "1,25(OH)2D" or hydroxyvitamin D? or dihydroxyvitamin D?).ab,kw,ti. (118981)
4 (paricalcitol or doxercalciferol or doxerocalciferol or calcifediol or calcitriol).ab,kw,ti. (8485)
5 hypovitaminosis D?.ab,kw,ti. (3033)
6 ((D or D2 or D3) adj3 supplement*).ab,kw,ti. (19335)
7 1 or 2 or 3 or 4 or 5 or 6 (172654)
8 betacoronavirus 1/ or betacoronavirus/ or human coronavirus oc43/ (1085)
9 Middle East respiratory syndrome coronavirus/ (2082)
10 sars-related coronavirus/ or sars coronavirus/ (6062)
11 Coronavirinae/ (2060)
12 coronavirus infection/ or middle east respiratory syndrome/ or severe acute respiratory syndrome/ (12565)

For peer review only

13 (((corona* or corono*) adj1 (virus* or viral* or virinae*)) or coronavirus* or coronovirus* or coronavirinae* or Coronavirus* or Coronovirus* or Wuhan* or Hubei* or Huanan or "2019-nCoV" or 2019nCoV or nCoV2019 or "nCoV-2019" or "COVID-19" or COVID19 or "CORVID-19" or CORVID19 or "WN-CoV" or WNCoV or "HCoV-19" or HCoV19 or CoV or "2019 novel*" or Ncov or "n-cov" or "SARS-CoV-2" or "SARSCoV-2" or "SARSCoV2" or "SARS-CoV2" or SARSCov19 or "SARS-Cov19" or "SARSCov-19" or "SARS-Cov-19" or Ncovor or Ncorona* or Ncorono* or NcovWuhan* or NcovHubei* or NcovChina* or NcovChinese*).ab,kw,ti. (30532)
14 (severe acute respiratory syndrome or SARS or Middle East respiratory syndrome or MERS).ab,kw,ti. (17954)
15 (betacoronavirus* or betacoronavirinae*).ab,kw,ti. (286)
16 exp respiratory tract infection/ (460049)
17 (acute respiratory infection* or severe respiratory infection* or acute respiratory tract infection* or severe respiratory tract infection* or influenza or common cold or pneumonia or bronchitis).ab,kw,ti. (329779)
18 8 or 9 or 10 or 11 or 12 or 13 or 14 or 15 or 16 or 17 (674800)
19 7 and 18 (3315)
20 (metaanalys* or "meta analys*" or "meta-analys*").mp. (294469)
21 (systematic* adj2 review*).mp. (330720)
22 20 or 21 (475492)
23 19 and 22 (219)
24 limit 19 to (meta analysis or "systematic review") (145)
25 23 or 24 (219)
26 limit 25 to (conference abstract or conference paper or "conference review" or editorial or letter) (41)
27 25 not 26 (178)

Cochrane Database of Systematic Reviews (Cochrane Library)

Search Name: Vitamin D Covid and Acute Respiratory Infections SRs
Date Run: 20/05/2020 18:30:28
Comment:

ID	Search	Hits
#1	MeSH descriptor: [Vitamin D] explode all trees	5224
#2	MeSH descriptor: [Vitamin D Deficiency] this term only	1226
#3	((vitamin NEXT D?) or (vit NEXT D?) or (D NEXT vitamin?) or ergocalciferol or cholecalciferol or colecalciferol or calciferol or dihydroergocalciferol or sitocalciferol or dihydrotachysterol or dihydroxycholecalciferol? or hydroxycholecalciferol? or "25(OH)D" or "25(OH)D3" or 25OHD? or calcidiol or hydroxyergocalciferol or alfacalcidol or alphacalcidol or "1,25(OH)2D" or (hydroxyvitamin NEXT D?) or (dihydroxyvitamin NEXT D?)):ti,ab,kw	12959
#4	(paricalcitol or doxercalciferol or doxerocalciferol or calcifediol or calcitriol):ti,ab,kw	2417
#5	hypovitaminosis NEXT D?	303
#6	((D OR D2 OR D3) NEAR/3 supplement*):ti,ab,kw	5632
#7	#1 or #2 or #3 or #4 or #5 or #6	14461
#8	MeSH descriptor: [Coronavirus] this term only	2
#9	MeSH descriptor: [Betacoronavirus] this term only	2
#10	MeSH descriptor: [Betacoronavirus 1] this term only	0
#11	MeSH descriptor: [Coronavirus OC43, Human] this term only	0
#12	MeSH descriptor: [Middle East Respiratory Syndrome Coronavirus] explode all trees	1

For peer review only - http://bmjopen.bmj.com/site/about/guidelines.xhtml

#13 MeSH descriptor: [SARS Virus] this term only 9
#14 MeSH descriptor: [Coronavirus Infections] this term only 137
#15 MeSH descriptor: [Severe Acute Respiratory Syndrome] this term only 107
#16 (((corona* or corono*) near/1 (virus* or viral* or virinae*)) or coronavirus* or coronovirus* or coronavirinae* or Coronavirus* or Coronovirus* or Wuhan* or Hubei* or Huanan or "2019-nCoV" or 2019nCoV or nCoV2019 or "nCoV-2019" or "COVID-19" or COVID19 or "CORVID-19" or CORVID19 or "WN-CoV" or WNCoV or "HCoV-19" or HCoV19 or CoV or "2019 novel*" or Ncov or "n-cov" or "SARS-CoV-2" or "SARSCoV-2" or "SARSCoV2" or "SARS-CoV2" or SARSCov19 or "SARS-Cov19" or "SARSCov-19" or "SARS-Cov-19" or Ncovor or Ncorona* or Ncorono* or NcovWuhan* or NcovHubei* or NcovChina* or NcovChinese*):ti,ab,kw 616
#17 ("severe acute respiratory syndrome" or SARS or "Middle East respiratory syndrome" or MERS):ti,ab,kw 351
#18 (betacoronavirus* or betacoronavirinae*):ti,ab,kw 4
#19 MeSH descriptor: [Respiratory Tract Infections] explode all trees 14360
#20 (("acute respiratory" NEXT infection*) or ("severe respiratory" NEXT infection*) or ("acute respiratory tract" NEXT infection*) or ("severe respiratory tract" NEXT infection*) or influenza or "common cold" or pneumonia or bronchitis):ti,ab,kw 25944
#21 #8 or #9 or #10 or #11 or #12 or #13 or #14 or #15 or #16 or #17 or #18 or #19 or #20 32554
#22 #7 and #21 329

CDSR: 3

Expert consultation

One additional study identified:

Martineau AR, Jolliffe DA, Hooper RL, et al., (2017) Vitamin D supplementation to prevent acute respiratory tract infections: systematic review and meta-analysis of individual participant data. BMJ. 2017;356:i6583. doi:10.1136/bmj.i6583

For peer review only - http://bmjopen.bmj.com/site/about/guidelines.xhtml

2. Full details of the study eligibility criteria

Include	Exclude
**P- Population**<br><br>1) Patients acutely ill with Betacoronavirus infection [SARS-CoV, MERS-CoV, SARS-CoV-2]<br>2) or at risk of acute illness with Betacoronavirus infection	Animals studies, modelling studies
**I – Intervention/exposure**<br><br>1) Vitamin D supplementation<br>2) Low Serum Vitamin D	
**O - Outcomes**<br><br>1) Betacoronavirus infection (to include serological evidence of infection or clinically confirmed symptomatic infection);<br>2) severity of Betacoronavirus infection (to include patients admitted to hospital or admitted to intensive care); mortality due to Betacoronavirus.<br>3) Mortality due to Betacoronavirus	
**C – Comparator**<br><br>1) No Vitamin D supplementation<br>2) high or normal Serum Vitamin D	
**S - Study design**<br>Randomised controlled trials and non-randomized studies will be eligible for inclusion in the review including, non randomized controlled trials, interrupted time series, controlled before-and-after studies, cohort studies, ecological studies, case reports and case series.	Qualitative studies, Non-primary research- reviews, editorials etc, guidelines and non-systematic reviews.
**Subgroups**<br><br>1. Ethnicity characteristics (White British, All Other White, Mixed, Asian, Black, Other)<br>2. Age characteristics (population by five-year age groups)	Non-English language. Non peer reviewed publication.

For peer review only

3. List of studies excluded at full text review

Excluded studies		Reason<br><br>*Non-eligible study design- e.g. review*<br>*Non-eligible population*<br>*Non-eligible intervention*<br>*No relevant outcome*<br>*No comparator group*
1	Adams, K. K., et al. (2020). "Myth Busters: Dietary Supplements and COVID-19." Annals of Pharmacotherapy: 1060028020928052.	Study design – commentary
2	Ahmed, I., et al. (2020). "First Covid-19 maternal mortality in the UK associated with thrombotic complications." British Journal of Haematology. **18**.	Study design – letter
3	Alpalhao, M. and P. Filipe (2020). "SARS-CoV-2 pandemic and Vitamin D deficiency - a double trouble." Photodermatology, Photoimmunology & Photomedicine **01**: 01.	Study design – letter
4	Annweiler, C., et al. (2020). COvid-19 and Vitamin D Supplementation: a Multicenter Randomized Controlled Trial of High Dose Versus Standard Dose Vitamin D3 in High-risk COVID-19 Patients (CoVitTrial). https://clinicaltrials.gov/show/NCT04344041.	Study design – on-going RCT completion date. July 2020
5	Arya, A. and V. D. Dwivedi (2020). "Synergistic effect of vitamin D and remdesivir can fight COVID-19." Journal of Biomolecular Structure & Dynamics: 1-2	Study design – letter
6	Banerjee, D., et al. (2020). "COVID-19 infection in kidney transplant recipients." Kidney International **97**(6): 1076-1082.	Study design – commentary
7	Caccialanza, R., et al. (2020). Early nutritional supplementation in non-critically ill patients hospitalized for the 2019 novel coronavirus disease (COVID-19): Rationale and feasibility of a shared pragmatic protocol. Nutrition: 110835.	Study design - protocol
8	Calder, P. C., et al. (2020). Optimal Nutritional Status for a Well-Functioning Immune System Is an Important Factor to Protect against Viral Infections. Nutrients: 12(4), 1181.	Study design -narrative review
9	Cao, Z., et al. (2020). SARS-CoV-2 & Covid-19: Key-Roles of the 'Renin-Angiotensin' System / Vitamin D Impacting Drug and Vaccine Developments. Infectious Disorders - Drug Targets. *(E-pub Ahead of Print)* DOI : 10.2174/1871526520999200505174704	Study design – editorial
10	Carter, S. J., et al. (2020). Considerations for obesity, vitamin D, and physical activity amidst the COVID-19 pandemic. Obesity **16**: 16.	Study design -narrative review

For peer review only

11	Daneshkhah, A., et al. (2020). The Possible Role of Vitamin D in Suppressing Cytokine Storm and Associated Mortality in COVID-19 Patients. medRxiv. DOI: https://doi.org/10.1101/2020.04.08.20058578	Study design – modelling
12	Davies G, Garami AR, Byers JC. Evidence Supports a Causal Model for Vitamin D in COVID-19 Outcomes. medRxiv, 2020. DOR: https://doi.org/10.1101/2020.05.01.20087965v3	Study design – modelling
13	de Lucena, T. M. C., et al. (2020). "Mechanism of inflammatory response in associated comorbidities in COVID-19." Diabetes & Metabolic Syndrome **14**(4): 597-600.	Study design -narrative review
14	Eroglu, C., et al. (2019). The relation between serum vitamin D levels, viral infections and severity of attacks in children with recurrent wheezing. Allergologia et Immunopathologia **47**(6): 591-597.	Population - Not COVID-19/SARs/MERs
15	Faul, J. L., et al. (2020). "Vitamin d deficiency and ards after sars-cov-2 infection." Irish Medical Journal **113**(5).	Study design – letter
16	Ghasemian, R., et al. (2020). "The Role of Vitamin D in The Age of COVID-19: A Systematic Review and Meta-Analysis Along with an Ecological Approach." MedRxiv : the Preprint Server for Health Sciences. DOI: 10.1101/2020.06.05.20123554	Study design -narrative review
17	Grant, W. B., et al. (2020). Evidence that Vitamin D Supplementation Could Reduce Risk of Influenza and COVID-19 Infections and Deaths. Nutrients **12**(4): 02.	Study design -narrative review
18	Heiser, K., et al. (2020). Identification of potential treatments for COVID-19 through artificial intelligence-enabled phenomic analysis of human cells infected with SARS-CoV-2. bioRxiv: 2020.2004.2021.054387.	Study design – modelling
19	Hribar, C. A., et al. (2020). "Potential Role of Vitamin D in the Elderly to Resist COVID-19 and to Slow Progression of Parkinson's Disease." Brain Sciences **10**(5): 08.	Study design -narrative review
20	Jakovac, H. (2020). COVID-19 and vitamin D-Is there a link and an opportunity for intervention? American Journal of Physiology - Endocrinology & Metabolism **318**(5): E589-E589.	Study design – letter
21	Jamaati, H., et al. (2020). A fourteen-day experience with coronavirus disease 2019 (COVID-19) induced acute respiratory distress syndrome (ARDS): An Iranian treatment protocol. Iranian Journal of Pharmaceutical Research **19**(1): 31-36.	Study design – treatment protocol/recommendation
22	Kalippurayil Moozhipurath, R., et al. (2020). "Evidence of Protective Role of Ultraviolet-B (UVB) Radiation in	Exposure – not vitamin D(UVB)

For peer review only - http://bmjopen.bmj.com/site/about/guidelines.xhtml

	Reducing COVID-19 Deaths." MedRxiv : the Preprint Server for Health Sciences. DOI 10.1101/2020.05.06.20093419	
23	Kara, M., et al. (2020). "'Scientific Strabismus' or Two Related Pandemics: COVID-19 & Vitamin D Deficiency." British Journal of Nutrition: 1-20.	Study design -narrative review
24	Koivisto, O., et al. (2020). Key Vitamin D Target Genes with Functions in the Immune System. Nutrients, **12**(4):1140.	Population - Outcomes - target genes
25	Kow, C. S., et al. (2020). "Vitamin D Supplementation in Influenza and COVID-19 Infections Comment on: "Evidence that Vitamin D Supplementation Could Reduce Risk of Influenza and COVID-19 Infections and Deaths" Nutrients 2020, 12(4), 988." Nutrients **12**(6): 01.	Study design – commentary
26	Kumar, V. and A. Srivastava (2020). "Spurious Correlation? A review of the relationship between Vitamin D and Covid-19 infection and mortality." MedRxiv : the Preprint Server for Health Sciences. DOI: 10.1101/2020.05.25.20110338	Study design -narrative review
27	Laird, E., et al. (2020). "Vitamin D and inflammation: Potential implications for severity of Covid-19." Irish Medical Journal **113**(5).	Study design -narrative review
28	La Vignera, S., et al. (2020). Sex-Specific SARS-CoV-2 Mortality: Among Hormone-Modulated ACE2 Expression, Risk of Venous Thromboembolism and Hypovitaminosis D. International journal of molecular sciences **21**(8):2948.	Study design – editorial
29	Li, A. Y., et al. (2020). Multivariate Analysis of Factors Affecting COVID-19 Case and Death Rate in U.S. Counties: The Significant Effects of Black Race and Temperature. medRxiv. DOI: https://doi.org/10.1101/2020.04.17.20069708	Intervention – not Vitamin D
30	Maestri, E., et al. (2020). Vitamin D and coronavirus: a new field of use?. Recenti Progressi in Medicina **111**(4): 253-256.	Study design -narrative review, non-English
31	Mansbach, J. M. and C. A. Camargo Jr., (2009). Respiratory Viruses in Bronchiolitis and Their Link to Recurrent Wheezing and Asthma. Clinics in Laboratory Medicine **29**(4): 741-755.	Population - Not COVID-19/SARs/MERs
32	Marik, P. E., et al. (2020). Does vitamin D status impact mortality from SARS-CoV-2 infection? Medicine in Drug Discovery: 100041-100041.	Study design - commentary
33	McKenna, M. J. and M. A. T. Flynn (2020). "Covid-19, cocooning and vitamin d intake requirements." Irish Medical Journal **113**(5).	Study design -narrative review

For peer review only

34	Mitchell, F. (2020). "Vitamin-D and COVID-19: do deficient risk a poorer outcome?" The Lancet Diabetes & Endocrinology **20**: 20.	Study design -narrative review
35	Molloy, E. J. and N. Murphy (2020). Vitamin D, Covid-19 and Children. Irish Medical Journal **113**(4): 64.	Study design -narrative review
36	McCartney, D. M. and D. G. Byrne (2020). Optimisation of Vitamin D Status for Enhanced Immuno-protection Against Covid-19. Irish Medical Journal **113**(4): 58.	Study design -narrative review
37	Rabbitt, L. and E. Slattery (2020). "Vitamin d and covid-19: A note of caution." Irish Medical Journal **113**(5).	Study design - letter
38	Ribeiro, H., et al. (2020). "Does Vitamin D play a role in the management of Covid-19 in Brazil?" Revista de Saude Publica **54**: 53.	Study design -narrative review
39	Romano, L., et al. (2020). "Short Report - Medical nutrition therapy for critically ill patients with COVID-19." European Review for Medical and Pharmacological Sciences **24**(7): 4035-4039.	Study design -narrative review
40	Silberstein, M. (2020). Vitamin D: A simpler alternative to tocilizumab for trial in COVID-19? Medical Hypotheses **140**: 109767-109767.	Study design -narrative review
41	Speeckaert, M. M. and J. R. Delanghe (2020). "Association between low vitamin D and COVID-19: don't forget the vitamin D binding protein." Aging Clinical & Experimental Research **28**: 28.	Study design - letter
42	Skutsch, M., et al. (2020). "The association of UV with rates of COVID-19 transmission and deaths in Mexico: the possible mediating role of vitamin D." MedRxiv : the Preprint Server for Health Sciences. DOI: 10.1101/2020.05.25.20112805	Study design – modelling
43	Suresh, P. S. (2020). "Hypovitaminosis D and COVID-19: Matter of Concern in India?" Indian Journal of Clinical Biochemistry.	Study design - letter
44	Taghizadieh, A., et al. (2020). "Acute kidney injury in pregnant women following SARS-CoV-2 infection: A case report from Iran." Respiratory Medicine Case Reports 30	Exposure – not vitamin D
45	Tan, S. H. S., et al. (2020). "Medications in COVID-19 patients: summarizing the current literature from an orthopaedic perspective." International Orthopaedics **22**: 22.	Study design -narrative review
46	Yalaki, Z., et al. (2019). Comparison of viral agents and vitamin D levels in children with acute bronchiolitis infection. Cocuk Enfeksiyon Dergisi **13**(1): e14-e20.	Population - COVID-19/SARs/MERs not specified
47	Zabetakis, I., et al. (2020). "COVID-19: The Inflammation Link and the Role of Nutrition in Potential Mitigation." Nutrients **12**(5): 19.	Study design -narrative review

For peer review only - http://bmjopen.bmj.com/site/about/guidelines.xhtml

48	Zemb, P., et al. (2020). "Vitamin D deficiency and COVID-19 pandemic." Journal of Global Antimicrobial Resistance **28**: 28.	Study design - commentary

4. Articles included at full text, but later excluded at time of narrative synthesis

	Citation record	Exclusion reason	Update performed 8th October 2020
49	Darling, A. L., et al. (2020). Vitamin D status, body mass index, ethnicity and COVID-19: Initial analysis of the first-reported UK Biobank COVID-19 positive cases (n 580) compared with negative controls (n 723). medRxiv.<br><br>https://www.medrxiv.org/content/10.1101/2020.04.29.20084277v1?versioned=TRUE	Not peer reviewed publication at time of narrative synthesis	No update available
50	De Smet, D., et al. (2020). Vitamin D deficiency as risk factor for severe COVID-19: a convergence of two pandemics. medRxiv.<br><br>https://www.medrxiv.org/content/10.1101/2020.05.01.20079376v2	Not peer reviewed publication at time of narrative synthesis	No update available
51	Lau, F. H., et al. (2020). Vitamin D Insufficiency is Prevalent in Severe COVID-19. medRxiv.<br><br>https://www.medrxiv.org/content/10.1101/2020.04.24.20075838v1?versioned=TRUE	Not peer reviewed publication at time of narrative synthesis	No update available
52	Meltzer, D. O., et al. (2020). "Association of Vitamin D Deficiency and Treatment with COVID-19 Incidence." MedRxiv : the Preprint Server for Health Sciences **13**: 13.	Not peer reviewed public	An updated publication is available at https://jamanetwork.com/journals/jamanetworkopen/fullarticle/2770157<br><br>Citation

For peer review only

	https://www.medrxiv.org/content/10.1101/2020.05.08.20095893v1	ation at time of narrative synthesis	Meltzer DO, Best TJ, Zhang H, Vokes T, Arora V, Solway J. Association of Vitamin D Status and Other Clinical Characteristics With COVID-19 Test Results. *JAMA Netw Open.* 2020;3(9):e2019722. doi:10.1001/jamanetworkopen.2020.19722
53	Notari, A. and G. Torrieri (2020). "COVID-19 transmission risk factors." MedRxiv : the Preprint Server for Health Sciences. <br>https://www.medrxiv.org/content/10.1101/2020.05.08.20095083v1?versioned=TRUE	Not peer reviewed publication at time of narrative synthesis	No update available
54	Raisi-Estabragh, Z., et al. (2020). "Greater risk of severe COVID-19 in non-White ethnicities is not explained by cardiometabolic, socioeconomic, or behavioural factors, or by 25(OH)-vitamin D status: study of 1,326 cases from the UK Biobank." MedRxiv : the Preprint Server for Health Sciences. <br>https://www.medrxiv.org/content/10.1101/2020.06.01.20118943v1?versioned=TRUE	Not peer reviewed publication at time of narrative synthesis	An updated publication is available at https://academic.oup.com/jpubhealth/article/42/3/451/5859581 <br>Citation Zahra Raisi-Estabragh, Celeste McCracken, Mae S Bethell, Jackie Cooper, Cyrus Cooper, Mark J Caulfield, Patricia B Munroe, Nicholas C Harvey, Steffen E Petersen, Greater risk of severe COVID-19 in Black, Asian and Minority Ethnic populations is not explained by cardiometabolic, socioeconomic or behavioural factors, or by 25(OH)-vitamin D status: study of 1326 cases from the UK Biobank, *Journal of Public Health*, Volume 42, Issue 3, September 2020, Pages 451–460, https://doi.org/10.1093/pubmed/fdaa095
55	Tan, C. W., et al. (2020). "A cohort study to evaluate the effect of combination Vitamin D, Magnesium and Vitamin B12 (DMB) on progression to severe outcome in older COVID-19 patients." MedRxiv : the Preprint Server for Health Sciences. <br>https://www.medrxiv.org/content/10.1101/2020.06.01.20112334v2	Not peer reviewed publication at time of narrative synthesis	No update available

For peer review only - http://bmjopen.bmj.com/site/about/guidelines.xhtml

Study details of the seven articles included at full text, but excluded at time of narrative synthesis						
**Study**	**Design/ setting**	**Population**	**Exposure/Intervention**	**Outcomes**	**Results**	**Limitations**
Darling, A. L., et al. (2020)[1]	Retrospective cohort study<br><br>UK Biobank England cohort only	COVID-19 positive cases (n 580) Mean age 57.5 (SD 9.7)<br><br>COVID-19 negative controls (n 723) Mean age 57.9 (SD 8.7)	Serum 25(OH)D status Median (IQR) nmol/L<br><br>by gender (Male/Female), body mass index (Normal/underweight, overweight, obesity), ethnicity (Asian, Black, Mixed and Other, White)	COVID-19 test result	Serum 25(OH)D status similar in both groups:<br><br>COVID-19 positive cases (median IQR) = 43.3 (32.1) nmol/L)<br><br>COVID-19 negative controls (median (IQR) 44.1 (31.2) nmol/L) for COVID-19.<br><br>A logistic regression model suggests that being overweight (OR 1.51 CI 1.13-2.02) or obese (OR 1.67 CI 1.24-2.26); living in London (OR 1.45 CI 1.05-2.00); being male (OR 1.28 CI 1.01-1.61) and being of Asian, Black or Mixed ethnicity (OR 1.66 CI 1.08-2.54) is associated with a higher odds of testing positive for COVID-19	UK Biobank baseline samples collected in 2006-2010.
De Smet, D., et al. (2020)[2]	Retrospective observational study<br><br>Central network hospital, West Flanders, Belgium	186 SARS-CoV-2 infected patients hospitalised from March 1, 2020 to April 7, 2020<br><br>(109 males [median age 68 years, IQR 53-79] 77 females [median age 71 years, IQR 65-74])<br><br>25(OH)D in COVID-19 patients was compared a control group of 2717 patients with similar age distribution, sampled from March 1, 2019 to April 30, 2019. (999 males [median age 69 years, IQR 53-81] and 1718 females [median age 68 years, IQR 43-83]).	25(OH)D levels	SARS-CoV-2 infection	COVID-19 patients had a lower median 25(OH)D on admission (18.6 ng/mL, IQR 12.6-25.3) than controls (21.5 ng/mL, IQR 13.9-20.8, P=0.0016) and a higher percentage of vitamin D deficiency (defined as 25(OH)D < 20ng/mL): 58.6% versus 45.2% (P=0.0005).<br><br>In male COVID-19 patients, vitamin D deficiency was lower median 25(OH)D (17.6 ng/mL, IQR 12.7-24.0 versus 20.3 ng/mL, IQR 13.7-28.3, P=0.0234) and a higher deficiency rate (67.0% versus 49.2%, P=0.0006) than male controls.	The prevalence and age/sex/ seasonal-distribution of vitamin D status was derived from the general population sampled from 16274 consecutive, unselected and unique patients from January 1, 2019 to December 31, 2019.

For peer review only - http://bmjopen.bmj.com/site/about/guidelines.xhtml

Lau, F. H., et al. (2020)[3]	Retrospective observational study<br><br>A single, tertiary care academic (university) medical centre, Louisiana, New Orleans, USA	COVID-19 ICU patients (n 13) Mean age 61.5 (SD 15.7)<br><br>COVID-19 floor patients (n 7) Mean age 72.0 (SD 14.8)<br><br>Medical records of COVID-19 patients between March 27, 2020 and April 21, 2020<br><br>Vitamin D insufficiency (VDI) and COVID-19 metrics in ICU vs. floor patients	VDI: defined as serum 25(OH) D < 30 ng/mL)<br><br>Serum 25(OH) D status Mean (SD) ng/mL<br><br>by gender (Male/Female), body mass index (Normal, obesity), race (African American), age (elderly>65 years), hypertension	COVID-19 metrics	Overall, few significant differences were identified between ICU and floor patients: Lactate dehydrogenase was significantly higher among ICU patients (441.8 vs. 223.0, P=0.001). Also, body mass index was significantly higher among ICU patients (35.2 vs. 24.5, P=0.02).<br><br>Among ICU subjects, 11 (84.6%) had VDI, vs. 4 (57.1%) of floor subjects. 100% of ICU patients less than 75 years old had VDI (n=11). Among these, 64.6% (n=7) had critically low 25(OH) D (<20 ng/mL) and 3 had <10 ng/mL.<br><br>VDI is highly prevalent in dark-skinned people (82.1% of African Americans vs. 41.6% overall).<br><br>Male/Female ratio was 1.24 and 1.44 for COVID-19 and VDI respectively.	Statistical analysis was limited by the small number of subjects.
Meltzer, D. O., et al. (2020)[4]	Retrospective cohort study<br><br>University of Chicago Medicine, USA	4,314 patients tested for COVID-19 from 3/3/2020 to 4/10/2020. Among these, 499 had a vitamin D level in the year before testing.<br><br>COVID-19 positive cases with vitamin D deficient (n 178) Mean age 45.6<br><br>COVID-19 positive cases with not vitamin D deficient (n 321) Mean age 50.7	Vitamin D deficiency: defined by the most recent 25(OH) D <20ng/ml or 1,25-dihydroxycholecalciferol <18pg/ml within 1 year before COVID-19 testing. Treatment: defined by the most recent vitamin D type and dose, and treatment changes between the time of the most recent vitamin D level and time of COVID-19 testing<br><br>Vitamin D deficiency and treatment changes were combined to categorize vitamin D status at the time of COVID-19 testing as:<br>1)Likely deficient (last-level-deficient/treatment-not-increased)	Testing positive for COVID-19	In multivariable analysis, testing positive for COVID-19 was associated with increasing age (RR (age<50)=1.05, P<0.021; RR (age≥50)=1.02, P<0.064)), non-white race (RR=2.54, P<0.01) and being likely vitamin D deficient (deficient/treatment-not-increased: RR=1.77, P<0.02) as compared to likely vitamin D sufficient (not-deficient/treatment-not-decreased), with predicted COVID-19 rates in the vitamin D deficient group of 21.6% (95%CI [14.0%-29.2%]) vs 12.2% (95%CI [8.9%-15.4%]) in the vitamin D sufficient group.<br><br>Vitamin D deficiency declined with increasing vitamin D dose (especially of vitamin D3). Vitamin D dose was not significantly associated with testing positive for COVID-19 (P=0.18).	The associations observed might not reflect causal effects of vitamin D deficiency on COVID-19. This is because vitamin D deficiency can reflect a range of chronic health conditions or behavioural factors which plausibly decrease the likelihood of treatment of vitamin D

For peer review only - http://bmjopen.bmj.com/site/about/guidelines.xhtml

			2)Likely sufficient (last-level-not-deficient/treatment-not-decreased)<br>3)Uncertain deficiency (last-level-deficient/treatment-increased or last-level-not-deficient/treatment-decreased)<br><br>by age (<50, ≥50), gender (Male/Female), race (White, other than White), ethnicity (Hispanic, not Hispanic), body mass index, employee status, comorbidity indicators (e.g. hypertension)			deficiency and increase COVID-19 risk.
Notari, A. and G. Torrieri (2020)[5]	Correlational study<br><br>126 countries, Only 50 countries for vitamin D	The number of cases follows in its early stages an almost exponential expansion. A starting point in each country was chosen: the first day di with 30 cases and fitted for 12 days. Thus, capturing the early exponential growth.<br><br>Countries with too small total population (less than 300 thousands inhabitants) were excluded.	They analysed risk factors correlated with the initial transmission growth rate of COVID-19<br><br>Average annual level of serum Vitamin D and the seasonal level<br><br>The seasonal level is defined as: the amount during March or during winter for northern hemisphere, or during summer for southern hemisphere or the annual level for countries with little seasonal variation.	Growth rate of COVID-19	They looked for linear correlations of the exponents with other variables, for a sample of 126 countries.<br><br>They found a positive correlation, i.e. faster spread of COVID-19, with high confidence level with the following variables, with respective p-value: low Temperature (4.10^{-7}), high ratio of old vs. working-age people (3.10^{-6}), life expectancy (8.10^{-6}), number of international tourists (1.10^{-5}), earlier epidemic starting date di (2.10^{-5}), high level of physical contact in greeting habits (6.10^{-5}), lung cancer prevalence (6.10^{-5}), obesity in males (1.10^{-4}), share of population in urban areas (2.10^{-4}), cancer prevalence (3.10^{-4}), alcohol consumption (0.0019), daily smoking prevalence (0.0036), UV index (0.004, smaller sample, 73 countries), low Vitamin D serum levels (0.002-0.006, smaller sample, 50 countries). There is highly significant correlation also with blood type. Also, positive correlation with moderate CI (p-value of 0.02-0.03) with: CO2/SO emissions, type-1 diabetes in children, and low vaccination coverage for Tuberculosis (BCG).	The dataset for the annual vitamin D was built with the available literature, which is quite inhomogeneous.<br><br>The dataset for the seasonal levels is more restricted. This is because the relative literature is less complete. So, for this the authors have included only 42 countries.

For peer review only - http://bmjopen.bmj.com/site/about/guidelines.xhtml

					Vitamin D is not highly correlated with UV index due to different food consumption in different countries.	
Raisi-Estabragh, Z., et al. (2020)[6]	Retrospective cohort study<br><br>UK Biobank	4,510 UK participants tested for COVID-19.<br><br>Latest data release (29/05/2020) includes test results from 16/03/2020 to 18/05/2020.<br><br>COVID-19 positive cases (n 1,326) Mean age 68.11 (SD 9.23)<br><br>COVID-19 negative controls (n 3,184) Mean age 68.91 (SD 8.72)	Serum 25(OH) D levels nmol/L<br><br>Multivariate logistic regression models by age, gender (Male/Female), ethnicity (Caucasian (any White background) and non-Caucasian: Black, Asian, Chinese) to test whether addition of: 1)cardio metabolic factors (e.g. hypertension, body mass index); 2) 25(OH)-vitamin D; 3) poor diet; 4) Townsend deprivation score; 5) housing; or 6)behavioural factors attenuated sex/ethnicity associations with COVID-19 status	COVID-19 test result<br><br>Greater risk of severe COVID-19	Over-representation of men and non-White ethnicities in the COVID-19 positive group.<br><br>Non-Whites had, on average, poorer cardio metabolic profile, lower 25(OH)-vitamin D, greater material deprivation, and were more likely to live in larger households and flats.<br><br>Male sex, non-White ethnicity, higher body mass index, Townsend deprivation score, and household overcrowding were independently associated with significantly greater odds of COVID-19. The pattern of association was consistent for men and women; cardio metabolic, socio-demographic and behavioural factors did not attenuate sex/ethnicity associations.	UK Biobank baseline samples collected in 2006-2010.<br><br>Aggregating all Black and Minority Ethnic (BAME) populations into one cohort might overlook important differences between non-Caucasian ethnicities.<br><br>The current dataset does not allow assessment of specific COVID-19 health outcomes.
Tan, C. W., et al. (2020)[7]	Cohort observational study<br><br>A tertiary academic hospital, Singapore	All 43 consecutive hospitalized COVID-19 patients aged 50 and above.<br><br>Between 15 January and 15 April 2020.	DMB = a single daily oral dose of vitamin D3 1000 IU, magnesium 150mg and vitamin B12 500mcg for up to 14 days<br><br>Adjusted for age, gender and comorbidities	Deterioration post-DMB administration leading to any form of oxygen therapy and/or intensive care	Duration of therapy: days, Median 5 (IQR 4-7)<br><br>Significantly fewer DMB patients than controls required initiation of oxygen therapy subsequently throughout their hospitalization (17.6% vs 61.5%, P=0.006).<br><br>On univariate analysis, increasing age and presence of comorbidities were associated	Small sample size, and the lack of systematic biologic measures to support their findings.

For peer review only - http://bmjopen.bmj.com/site/about/guidelines.xhtml

		17 patients received Vitamin D, Magnesium, Vitamin B12 (DMB): Mean age 58.4 (SD 7.0)<br><br>26 patients did not: Mean age 64.1 (SD 7.9)		support for COVID-19 patients	with significantly higher OR for oxygen therapy, while exposure to DMB therapy was associated with a significantly improved OR 0.13 (95% CI: 0.03 – 0.59, P=0.008) .<br><br>On multivariate analysis, increasing age was associated with significantly higher OR for oxygen therapy, while exposure to DMB therapy was associated with a significantly improved OR 0.15 (95% CI: 0.025 – 0.93, P=0.041).	

For peer review only

5. Risk of bias of included studies

Risk of bias assessment using the Downs and Black Checklist[8]

Study	Quality score	Reviewer notes
Hastie et al., 2020[9]	14/20<br><br>Seven domains were not applicable and therefore not assessed, 2 reporting, 1 external validity 3 internal validity (bias) and 1 internal validity (confounding).	The study could not be scored for 3 questions as we were unable to determine; 1) the representativeness of the subjects who were prepared to participate from entire population from which they were recruited, 2) whether losses to follow-up were taken into account as patients lost to follow-up were not reported and 3) whether the study had sufficient power to detect a clinically important effect.<br><br>The study did not score a point for 3 questions; 1) providing the number and a description of the characteristics of patients lost to follow-up, 2) stating whether study subjects in different intervention groups we recruited over the same period of time and 3) for assignment concealment as it was a non-randomised study. The study scored partially (only 1 point not two) for clearly described distributions of principal confounders in each group of subjects to be compared.
D'Avolio et al, 2020[10]	13/15<br><br>Twelve domains were not applicable therefore not assessed, 3 reporting, 1 external validity, 4 internal validity (bias) and 4 internal validity (confounding).	The study could not be scored for the 'power' domain as we were unable to determine from the article whether the study had sufficient power to detect a clinically important effect where the probability value for a difference being due to chance is less than 5%.<br><br>The study did not score 1 point in the 'external validity' domain as those subjects who were prepared to participate were not representative of the entire population from which they were recruited.
Fasano et al., 2020[11]	12/17<br>Ten domains were not applicable therefore not assessed, 3 reporting, 1 external validity, 4 internal validity (bias) and 2 internal validity (confounding).	The study could not be scored for 4 items, the 'power' domain and one question in the 'Internal validity - confounding (selection bias)' as the study did not specify the time period over which patients were recruited. It could also not be scored for 2 questions in the 'External validity domain', 1) the representativeness of the subjects asked to participate in the study compared to the entire population from which they were recruited and 2) the representativeness of those subjects who were prepared to participate compared to the entire population from which they were recruited.<br><br>The study did not score 1 point as the effect of the main confounders was not investigated or confounding was demonstrated but no adjustment was made in the final analyses. The study scored two points for presentation of potential confounders.

For peer review only

Ilie et al., 2020[12]	4/20<br>Seven domains were not applicable and therefore not assessed, 1 reporting, 1 external validity, 3 internal validity (bias), 1 internal validity (confounding) and 1 for power.	The study could not be scored for 9 questions. Two in the 'reporting' domain, 1) interventions of interest not clearly described, 2) the main findings of the study are not clearly described. Two 'External validity' questions 1) the representativeness of the subjects asked to participate in the study compared to the entire population from which they were recruited and 2) the representativeness of those subjects who were prepared to participate compared to the entire population from which they were recruited. Two 'Internal validity – bias' domain questions 1) all analyses that had not been planned at the outset of the study were not clearly indicated (results of the study based on "data dredging", were not made clear), and 2) it was not clear is the statistical techniques used were appropriate to the data. Three 'Internal validity - confounding (selection bias)' domain questions, 1) no information provided concerning the source of patients included in the study 2) does not specify the time period over which patients were recruited, and 3) the numbers of patients lost to follow-up are not reported.<br><br>The study did not score 7 points for the following; 3 reporting issues 1) no description of the characteristics of participants included in the study 2) no description of the distributions of principal confounders in each group of subjects to be compared, and 3) no description of the characteristics of patients lost to follow-up. Two internal validity bias issues 1) differences in follow-up were ignored and 2) no evidence that the main measure used were accurate (valid and reliable).
**Note:** For each included study, the maximum possible quality score was dependent on which domains could be assessed based on the study design. The higher the score assigned to a study, the lower the risk of bias. For example, Hastie et al. 2020[9] was assigned a score of 14 out of a maximum possible score of 20, suggesting good quality and therefore low risk of bias compared to the other studies.		

For peer review only

References

1. Darling AL, Ahmadi KR, Ward KA, et al. Vitamin D status, body mass index, ethnicity and COVID-19: initial analysis of the first-reported UK Biobank COVID-19 positive cases (n 580) compared with negative controls (n 723). *medRxiv* 2020. doi: 10.1101/2020.04.29.20084277
2. De Smet D, De Smet K, Herroelen P, Gryspeerdt S, Martens GA. Vitamin D deficiency as risk factor for severe COVID-19: a convergence of two pandemics. *medRxiv* 2020. doi: 10.1101/2020.05.01.20079376
3. Lau FH, Majumder R, Torabi R, et al. Vitamin D insufficiency is prevalent in severe COVID-19. *medRxiv* 2020. doi: 10.1101/2020.04.24.20075838

4. Meltzer DO, Best TJ, Zhang H, Vokes T, Arora V, Solway J. Association of vitamin D deficiency and treatment with COVID-19 incidence. *medRxiv* 2020. doi: 10.1101/2020.05.08.20095893
5. Notari A, Torrieri G. COVID-19 transmission risk factors. *medRxiv* 2020. doi: 10.1101/2020.05.08.20095083
6. Raisi-Estabragh Z, McCracken C, Bethell MS, et al. Greater risk of severe COVID-19 in non-White ethnicities is not explained by cardiometabolic, socioeconomic, or behavioural factors, or by 25(OH)-vitamin D status: study of 1,326 cases from the UK Biobank. *medRxiv* 2020. doi: 10.1101/2020.06.01.20118943
7. Tan CW, Ho LP, Kalimuddin S, et al. A cohort study to evaluate the effect of combination vitamin D, magnesium and vitamin B12 (DMB) on progression to severe outcome in older COVID-19 patients. *medRxiv* 2020. doi: 10.1101/2020.06.01.20112334
8. Downs SH, Black N. The feasibility of creating a checklist for the assessment of the methodological quality both of randomised and non-randomised studies of health care interventions. *J Epidemiol Community Health* 1998;52(6):377-84. doi: 10.1136/jech.52.6.377
9. Hastie CE, Mackay DF, Ho F, et al. Vitamin D concentrations and COVID-19 infection in UK Biobank. *Diabetes Metab Syndr* 2020;14(4):561-65. doi: 10.1016/j.dsx.2020.04.050
10. D'Avolio A, Avataneo V, Manca A, et al. 25-hydroxyvitamin D concentrations are lower in patients with positive PCR for SARS-CoV-2. *Nutrients* 2020;12(5):1359. doi: 10.3390/nu12051359
11. Fasano A, Cereda E, Barichella M, et al. COVID-19 in Parkinson's Disease Patients Living in Lombardy, Italy. *Mov Disord* 2020. doi: 10.1002/mds.28176
12. Ilie PC, Stefanescu S, Smith L. The role of vitamin D in the prevention of coronavirus disease 2019 infection and mortality. *Aging Clin Exp Res* 2020. doi: 10.1007/s40520-020-01570-8

For peer review only

For peer review only - http://bmjopen.bmj.com/site/about/guidelines.xhtml

PRISMA 2009 Checklist

Section/topic	#	Checklist item	Reported on page #
**TITLE**			
Title	1	Identify the report as a systematic review, meta-analysis, or both.	1
**ABSTRACT**			
Structured summary	2	Provide a structured summary including, as applicable: background; objectives; data sources; study eligibility criteria, participants, and interventions; study appraisal and synthesis methods; results; limitations; conclusions and implications of key findings; systematic review registration number.	3
**INTRODUCTION**			
Rationale	3	Describe the rationale for the review in the context of what is already known.	5
Objectives	4	Provide an explicit statement of questions being addressed with reference to participants, interventions, comparisons, outcomes, and study design (PICOS).	6
**METHODS**			
Protocol and registration	5	Indicate if a review protocol exists, if and where it can be accessed (e.g., Web address), and, if available, provide registration information including registration number.	6
Eligibility criteria	6	Specify study characteristics (e.g., PICOS, length of follow-up) and report characteristics (e.g., years considered, language, publication status) used as criteria for eligibility, giving rationale.	7
Information sources	7	Describe all information sources (e.g., databases with dates of coverage, contact with study authors to identify additional studies) in the search and date last searched.	6
Search	8	Present full electronic search strategy for at least one database, including any limits used, such that it could be repeated.	Supp appendix 1
Study selection	9	State the process for selecting studies (i.e., screening, eligibility, included in systematic review, and, if applicable, included in the meta-analysis).	7 and supp appendix 2
Data collection process	10	Describe method of data extraction from reports (e.g., piloted forms, independently, in duplicate) and any processes for obtaining and confirming data from investigators.	8
Data items	11	List and define all variables for which data were sought (e.g., PICOS, funding sources) and any assumptions and simplifications made.	8
Risk of bias in individual studies	12	Describe methods used for assessing risk of bias of individual studies (including specification of whether this was done at the study or outcome level), and how this information is to be used in any data synthesis.	8

For peer review only - http://bmjopen.bmj.com/site/about/guidelines.xhtml

PRISMA 2009 Checklist

Section/topic	#	Checklist item	Reported on page #
Summary measures	13	State the principal summary measures (e.g., risk ratio, difference in means).	8
Synthesis of results	14	Describe the methods of handling data and combining results of studies, if done, including measures of consistency (e.g., I^2) for each meta-analysis.	8

Section/topic	#	Checklist item	Reported on page #
Risk of bias across studies	15	Specify any assessment of risk of bias that may affect the cumulative evidence (e.g., publication bias, selective reporting within studies).	n/a
Additional analyses	16	Describe methods of additional analyses (e.g., sensitivity or subgroup analyses, meta-regression), if done, indicating which were pre-specified.	8
**RESULTS**			
Study selection	17	Give numbers of studies screened, assessed for eligibility, and included in the review, with reasons for exclusions at each stage, ideally with a flow diagram.	8
Study characteristics	18	For each study, present characteristics for which data were extracted (e.g., study size, PICOS, follow-up period) and provide the citations.	8 and supp appendix 3
Risk of bias within studies	19	Present data on risk of bias of each study and, if available, any outcome level assessment (see item 12).	8/9 supp appendix 4
Results of individual studies	20	For all outcomes considered (benefits or harms), present, for each study: (a) simple summary data for each intervention group (b) effect estimates and confidence intervals, ideally with a forest plot.	8/9/10 and supp appendix 3
Synthesis of results	21	Present results of each meta-analysis done, including confidence intervals and measures of consistency.	n/a
Risk of bias across studies	22	Present results of any assessment of risk of bias across studies (see Item 15).	n/a
Additional analysis	23	Give results of additional analyses, if done (e.g., sensitivity or subgroup analyses, meta-regression [see Item 16]).	10
**DISCUSSION**			
Summary of evidence	24	Summarize the main findings including the strength of evidence for each main outcome; consider their relevance to key groups (e.g., healthcare providers, users, and policy makers).	11/12/13
Limitations	25	Discuss limitations at study and outcome level (e.g., risk of bias), and at review-level (e.g., incomplete retrieval of identified research, reporting bias).	13

For peer review only

PRISMA 2009 Checklist

Conclusions	26	Provide a general interpretation of the results in the context of other evidence, and implications for future research.	13/14
**FUNDING**			
Funding	27	Describe sources of funding for the systematic review and other support (e.g., supply of data); role of funders for the systematic review.	15

From: Moher D, Liberati A, Tetzlaff J, Altman DG, The PRISMA Group (2009). Preferred Reporting Items for Systematic Reviews and Meta-Analyses: The PRISMA Statement. PLoS Med 6(7): e1000097. doi:10.1371/journal.pmed1000097

For more information, visit: **www.prisma-statement.org**.

For peer review only

For peer review only - http://bmjopen.bmj.com/site/about/guidelines.xhtml
